# Cationic cluster formation versus disproportionation of low-valent indium and gallium complexes of 2,2'-bipyridine

**DOI:** 10.1038/ncomms9288

**Published:** 2015-10-19

**Authors:** Martin R. Lichtenthaler, Florian Stahl, Daniel Kratzert, Lorenz Heidinger, Erik Schleicher, Julian Hamann, Daniel Himmel, Stefan Weber, Ingo Krossing

**Affiliations:** 1 Institut für Anorganische und Analytische Chemie and Freiburger Materialforschungszentrum (FMF), Albert-Ludwigs-Universität Freiburg, Albertstr. 21 and Stefan-Meier Str. 21, 79104 Freiburg, Germany; 2 Institut für Physikalische Chemie and Freiburg Institute of Advanced Studies (FRIAS), Albert-Ludwigs-Universität Freiburg, Albertstr. 21 and Albertstr. 19, 79104 Freiburg, Germany

## Abstract

Group 13 M^I^ compounds often disproportionate into M^0^ and M^III^. Here, however, we show that the reaction of the M^I^ salt of the weakly coordinating alkoxyaluminate [Ga^I^(C_6_H_5_F)_2_]^+^[Al(OR^F^)_4_]^−^ (R^F^=C(CF_3_)_3_) with 2,2'-bipyridine (bipy) yields the paramagnetic and distorted octahedral [Ga(bipy)_3_]^2+^^•^{[Al(OR^F^)_4_]^−^}_2_ complex salt. While the latter appears to be a Ga^II^ compound, both, EPR and DFT investigations assign a ligand-centred [Ga^III^{(bipy)_3_}^•^]^2+^ radical dication. Surprisingly, the application of the heavier homologue [^I^n^I^(C_6_H_5_F)_2_]^+^[Al(OR^F^)_4_]^−^ leads to aggregation and formation of the homonuclear cationic triangular and rhombic [In_3_(bipy)_6_]^3+^, [In_3_(bipy)_5_]^3+^ and [In_4_(bipy)_6_]^4+^ metal atom clusters. Typically, such clusters are formed under strongly reductive conditions. Analysing the unexpected redox-neutral cationic cluster formation, DFT studies suggest a stepwise formation of the clusters, possibly via their triplet state and further investigations attribute the overall driving force of the reactions to the strong In−In bonds and the high lattice enthalpies of the resultant ligand stabilized [M_3_]^3+^{[Al(OR^F^)_4_]^−^}_3_ and [M_4_]^4+^{[Al(OR^F^)_4_]^−^}_4_ salts.

## Abstract (web summary)

Group 13 metals such as gallium and indium with oxidation states of +I tend to disproportionate into more stable 0 and +III states. Here, the authors report that with a 2,2'-bipyridine ligand Ga(+I) forms a paramagnetic mononuclear Ga(+III) complex, whereas the indium analogue aggregates forming cationic clusters retaining the +I state.

## Graphical abstract

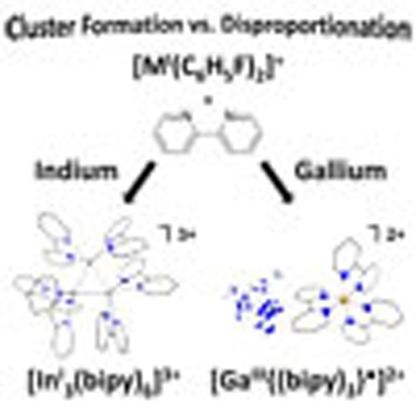

In 1966, F. A. Cotton defined the term metal atom cluster compound as ‘those containing a finite group of metal atoms which are held together entirely, mainly, or at least to a significant extent, by bonds directly between the metal atoms even though some non-metal atoms may be associated intimately with the cluster'^1^. Meanwhile, the term cluster has been expanded, describing various ensembles of bonded atoms (both metal and non-metal) or molecules, thus including compounds such as the boranes and carboranes^2^,^3^, Zintl-like phases^4^, salt-like clusters^5^ as well as metalloid clusters^6^. Among the many routes leading to metal atom cluster compounds, the reductive and anionic syntheses prevail: such clusters are typically electron deficient and have a strong demand for additional electrons that can be provided by reductants such as alkaline metals or through electron transfer reactions like disproportionations^6^,^7^. Alternatively, aggregation can be achieved by applying strong donor ligands: for example, the carbene-mediated formation of the neutral P_12_ non-metal cluster^8^. Though the different approaches have yielded a vast number of neutral and anionic cluster compounds, the redox-neutral synthesis of cationic clusters by low-valent cation aggregation has not been reported to our knowledge. Thus, univalent group 13 metal salts such as—partly hypothetic—M^+^A^–^ (M=Al, Ga, In, Tl; A=Cl, [AsF_6_], [Al(OR^F^)_4_] with R^F^=C(CF_3_)_3_) could in principle aggregate, yielding the electron precise [M_3_]^3+^(A^–^)_3_ cluster salt (Fig. 1, right).

The simple M^+^Cl^–^ salts however, are prone to disproportionate to elemental M^0^ and M^3+^(Cl^–^)_3_, due to the high and favourable lattice energies of MCl_3_ (Fig. 1, left). Thus, AlCl is only known as a gas phase molecule^9^, and a hypothetic salt Al^+^Cl^–^ would disproportionate to elemental Al^0^ and AlCl_3_ (solid-state reaction enthalpy Δ_r_*H*°(solid)=–396 kJ mol^–1^, cf. Born-Haber-Fajans Cycle (BHFC), Supplementary Fig. 9). For the heavier element indium on the other hand, the oxidation state +1 is more favourable due to inert pair effects^10^ and the known salt In^+^Cl^−^ (refs 11, 12) is stable towards disproportionation by +31 kJ mol^–1^ (BHFC, Supplementary Fig. 9). Yet, the alternative formation of the metal atom cluster [M_3_]^3+^ is hampered by the lower lattice energies of [M_3_]^3+^(A^–^)_3_ compared with M^3+^(A^–^)_3_ and, most of all, the very distinct Coulomb repulsion (Fig. 1, right). Thus, a gaseous triangular [M_3_]^3+^ cluster would Coulomb-explode, releasing +1,544 kJ mol^–1^ Coulomb energy at a typical M−M distance of 270 pm. The cluster formation, however, becomes favoured for larger anions A^−^: that is, the difference of the estimated lattice enthalpies (Δ_latt_*H*°) of the In^3+^(A^−^)_3_ and [In_3_]^3+^(A^−^)_3_ salts is +697 kJ mol^−1^ for Cl^−^ (*V*^–^=0.047 nm^3^), +281 kJ mol^−1^ for [AsF_6_]^−^ (*V*^–^=0.110 nm^3^) but only +26 kJ mol^−1^ for [Al(OR^F^)_4_]^−^ (*V*^–^=0.758 nm^3^; Supplementary Tables 10 and 11). Overall, salts with very large anions like [Al(OR^F^)_4_]^−^ and ligands, allowing for a delocalization and thus ‘dilution' of the positive charge on [M_3_]^3+^, may support cationic cluster formation. Yet, the question remains: what are suitable Ga^I^/In^I^ sources to perform such a chemistry?

Stabilizing gallium in its low oxidation states (<III) has been an objective since the 1,930 s. (ref. 13) Subvalent gallium halides Ga^I^[Ga^III^X_4_] (X=Cl, Br and I)^14^, ‘GaI' (refs 15, 16) and metastable, donor stabilized Ga^I^Cl solutions^9^ are important milestones and to this day used as starting material for further Ga^I^ chemistry: for example, arene-17, Cp^x^-complexes (Cp^x^: Cp=C_5_H_5_ and Cp*=C_5_Me_5_), (ref. 18) N-heterocyclic carbene (NHC) analogues^19^,^20^ of Ga^I^, or metalloidal gallium clusters^7^. In addition, the neutral Ga^I^-Cp/Cp* complexes as well as the *galla-*NHC analogues have been applied as ligands for transition-metals^21^. Due to the strongly coordinating halide anions, however, Ga^I^[Ga^III^X_4_] and ‘GaI' are prone to com- and disproportionation reactions^15^,^17^. Donor-free Ga^I^Cl and related compounds are only accessible at very low temperatures using elaborated matrix isolation techniques^9^. Simple indium halides In^I^X (X=Cl, Br and I)^11^,^12^,^22^ are stable at ambient conditions (*vide supra*). In contrast to the Ga^I^ systems, the halide anions can be replaced for weakly coordinating anions (WCAs) of different sizes including [BF_4_]^−^ (ref. 23), [OTf]^−^ (Tf=SO_2_CF_3_)^24^, [PnF_6_]^−^ (Pn=P, As, Sb)^25^ and [Al(OR^F^)_4_]^−^ (R^F^=C(CF_3_)_3_)^26^. Various In^I^-Cp complexes^22^ have also been used as catalysts in organic syntheses^27^. Our group introduced a simple route to univalent gallium and indium salts of the weakly coordinating [Al(OR^F^)_4_]^−^ anion, by oxidizing elemental gallium and indium with Ag^+^[Al(OR^F^)_4_]^−^ in fluorobenzene (C_6_H_5_F)^28^,^29^. Under inert conditions, the [M(C_6_H_5_F)_2_]^+^[Al(OR^F^)_4_]^−^ salts (M=Ga (**1**), In (**2**)) are stable, soluble in aromatic solvents (preferably fluorinated), and a potent starting material for further Ga^I^ and In^I^ chemistry: for example, phosphine^29^, crown ether^30^, carbene^31^ and N-heterocyclic arene^32^ complexes. In addition, the [Ga(arene)_1-2_]^+^ complexes (arene=C_6_H_5_F, mesitylene, diphenylethane, *m*-terphenyl) are highly efficient isobutylene polymerization catalysts^33^,^34^.

Herein we report on the surprising reactions of [M(C_6_H_5_F)_2_]^+^[Al(OR^F^)_4_]^−^ salts mainly with 2,2'-bipyridine (bipy), but also with the bipy-relative 1,10-phenanthroline (phen).

## Results

### Orienting quantum-chemical calculations

In contrast to the anionic chelating N-ligands for univalent gallium and indium, for example, guanidinates^35^, diazabutadienes^19^,^20^,^36^, *β*-diketiminates^20^,^37^ or tris(pyrazolyl)hydroborates (Tp)^38^, neutral N-ligands, like pyridine derivatives, were shown to only stabilize the +1 oxidation state of indium^39^, but not of gallium^40^,^41^. Applying [Ga^I^(C_6_H_5_F)_2_]^+^[Al(OR^F^)_4_]^−^ (**1**), however, we were able to isolate the first gallium(I) complexes with simple N-ligands, such as pyrazine and di-*tert*-butylmethylpyridine^32^. To expand the scope to neutral chelating N-ligands, we investigated the thermodynamics of potential ligand exchange reactions of the [M(C_6_H_5_F)_2_]^+^ complexes (M=Ga, In) and 1 to 3 equivalents bipy. All turned out to be exothermic/exergonic by at least −146 kJ  mol^–1^ (Supplementary Table 9). Thus, it appeared interesting to test the reactions.

### Reaction of [Ga^I^(C_6_H_5_F)_2_]^+^[Al(OR^F^)_4_]^−^ with bipy

**Figure 2:**
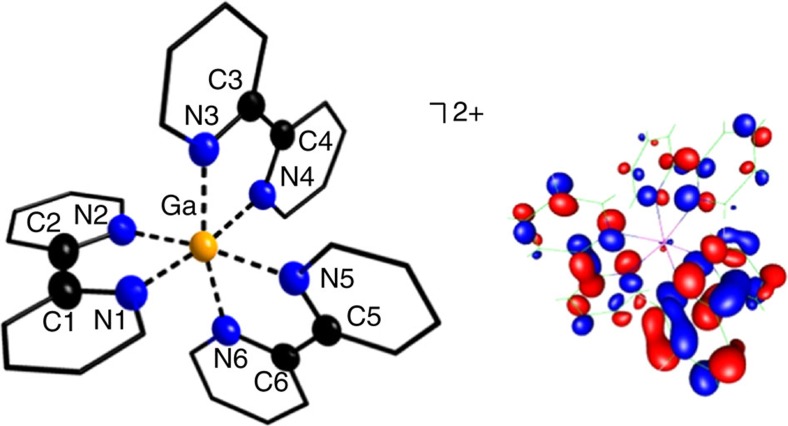
Molecular structure of [Ga(bipy)_3_]^2+•^ (**3**^**2+**^). Selected bond lengths are given in pm: Ga−N1=202.6(4), Ga−N2=202.6(4), Ga−N3=205.8(4), Ga−N4=208.2(4), Ga−N5=208.2(4), Ga−N6=205.8(4), C1−C2=149.2(12) and C3−C4=148.6(7), C5−C6=148.6(7). The earlier reported [Ga(bipy)_3_]^3+^ complex features a slightly longer average Ga−N bond length of 206.4 pm (cf. 205.5(4) pm for **3**^**2+**^) and a slightly shorter average C_1,3,5_−C_2,4,6_ bond length of 147.9 pm (cf. 148.8(9) pm for **3**^**2+**^)^40^. The [Al(OR^F^)_4_]^–^ anions and all of the hydrogen atoms were omitted for clarity. The thermal ellipsoids are set at 50% probability. An alternative presentation, featuring ellipsoids for all atoms, is included in Supplementary Fig. 6. As an inset, the figure also includes the B3LYP/SV(P) level frontier and singly occupied Kohn-Sham orbital (SOMO) of **3**^**2+**^, electron density cutoff at 0.04 a.u. The calculated distances of the latter are in good agreement with the experimental results (bond lengths in pm, similar label scheme): Ga−N1=206.4, Ga−N2=206.8, Ga−N3=211.6, Ga−N4=213.2, Ga−N5=211.1, Ga−N6=209.1, C1−C2=145.7, C3−C4=147.7 and C5−C6=146.9. Yet, and more importantly, the SOMO is primarily located on the bipy ligands, thus speaking for a [Ga^III^{(bipy)_3_}^•^]^2+^ rather than a [(Ga^II^)^•^(bipy)_3_]^2+^ complex. Applying the BHLYP/SV(P) level of theory, the SOMO is solely located on one bipy ligand, corresponding to a [Ga^3+^(bipy)_2_(bipy)^•^^−^]^2+^ complex (Supplementary Fig. 7). The calculated distances of the latter are in inferior agreement with the experimental results and therefore **3**^**2+**^ more likely corresponds to the [Ga^III^{(bipy)_3_}^•^]^2+^ formulation.

On mixing colourless solutions of **1** and bipy (2.00 eq) in *ortho*-difluorobenzene (*o*-C_6_H_4_F_2_), an unexpected distinct change in colour towards moss-green was observed (Supplementary Data 1–7). To promote crystallization, the reaction mixture was concentrated by slowly removing the volatiles under reduced pressure. During this process, the formation of a black precipitate was observed, while the colour of the solution remained green. Applying C_6_H_5_F as solvent, similar observations were made, though the precipitate formed without concentrating the solution. From the *o*-C_6_H_4_F_2_ solutions, green platelet single crystals were repeatedly isolated and surprisingly characterized as [Ga(bipy)_3_]^2+•^{[Al(OR^F^)_4_]^−^}_2_ (**3**) and not as the predicted [Ga(bipy)_2_]^+^ complex (cf. Supplementary Table 9). Though the [Al(OR^F^)_4_]^−^ anions are crucial in terms of stabilizing the low-valent oxidation states of gallium and indium, all structures within this manuscript feature no Ga−F or In−F contact shorter than the sum of the corresponding van der Waals radii^42^,^43^. The structural discussions are therefore focused on the obtained cationic complexes and clusters. To our knowledge, the dicationic, paramagnetic and distorted octahedral [Ga(bipy)_3_]^2+•^ complex (**3**^**2+**^) is the first structurally characterized one of its kind, thus differing from the [Ga(bipy)_3_]^3+^ complex obtained, if ‘GaI' is applied as gallium(I) starting material (Fig. 2)^40^. The synthesis of **3**^**2+**^ is the first example, where **1** did not act as a pure Ga^I^ source but disproportionated (*vide infra*). With a total charge of 2+, it is tempting to proclaim the successful stabilization of a monomeric, room-temperature stable Ga^II^ species, that is, a [(Ga^II^)^•^(bipy)_3_]^2+^ complex.

**Figure 3:**
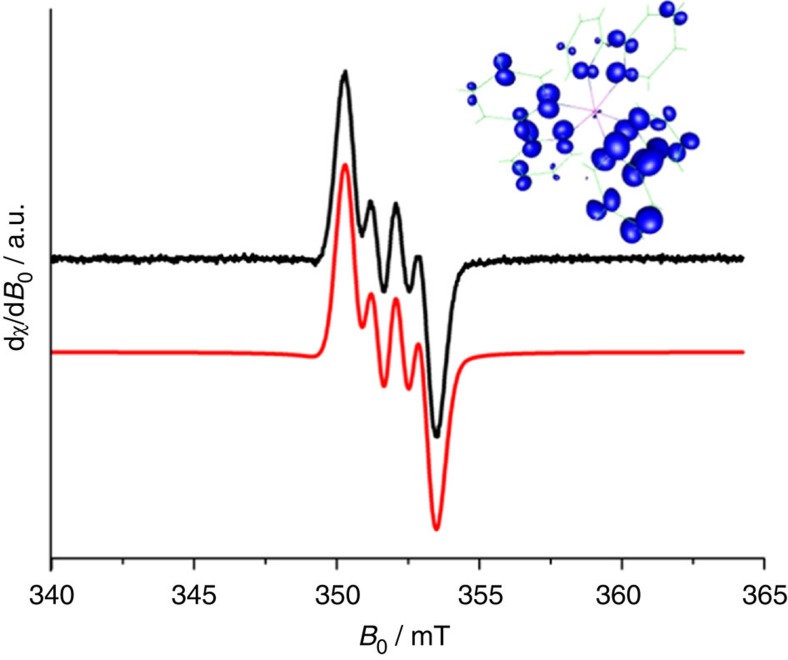
EPR investigations of [Ga(bipy)_3_]^2+•^{[Al(OR^F^)_4_]^−^}_2_ (**3**). X-band continuous wave EPR spectrum of a solution of **3** in *o*-C_6_H_4_F_2_ (1 mM) (black trace; recorded at room temperature, microwave frequency 9.861 GHz, magnetic-field modulation amplitude 0.1 mT, microwave power 2 mW) and the corresponding spectral simulation (red trace). The measurement and its spectral simulation exhibit an isotropic gallium dominated resonance with a *g*-value (*g*_iso_) of 2.0024 and a hyperfine coupling constant of *A*(^69^Ga)=21 MHz, thus supporting an isotropic spin density of <1.0% on the gallium atom and a ligand-centred spin system, while the exact whereabouts of the radical in the ligand backbone could not be identified. The *A*(^69^Ga) value strongly deviates from the theoretical hyperfine coupling constant of an isolated ^69^Ga atom (*A*(^69^Ga)=12210 MHz)^68^,^69^. In addition spectra were obtained from frozen solutions of **3** in C_6_H_5_F or *o*-C_6_H_4_F_2_ and both show a weak *g* anisotropy (Supplementary Fig. 4). As an inset, the figure also includes the DFT calculated spin density of **3**^**2+**^ (cutoff at 0.003 a.u., B3LYP/SV(P) level). With a distribution of 3.0% on the gallium atom and 97% on the bipy ligands, the latter corresponds to a [Ga^III^{(bipy)_3_}^•^]^2+^ complex, thus being in very good agreement with the EPR results. An alternative modelling at the BHLYP/SV(P) level yielded a spin density distribution of 1.3% on the gallium atom and 98% on one bipy ligand (Supplementary Fig. 7), suggesting a formulation as [Ga^3+^(bipy)_2_(bipy)^•^^−^]^2+^. Yet, a distribution of the spin density on all three ligands seems to be more likely as the calculated C−C and In−N bond lengths of the [Ga^III^{(bipy)_3_}^•^]^2+^ complex are in much better agreement with the experimental results.

While earlier reported Ga^II^ compounds have proven to be mixed-valent species^14^ or diamagnetic dimers^44^, Aldridge *et al.* recently reported on a thermally robust monomeric Ga^II^ compound: [Ga^II^{B(N(C_6_H_3_-2,3-*i*Pr)CH)_2_}_2_]^45^. In this Ga^II^ molecule, the metal atom is coordinated in a bent fashion by two boryl ligands and over 70% of the unpaired spin density is located on the metal. The related [Ga(dabab)_2_] complex^46^ (dabab=1,4-di-*tert*-butyl-1,4-diazabutadiene) on the other hand, is correctly described as a Ga^III^ cation coordinated by one singly and one doubly reduced dabab ligand^36^,^47^,^48^,^49^,^50^. A related description could account for **3**^**2+**^, that is, a [Ga^III^{(bipy)_3_}^•^]^2+^ complex, and therefore we conducted electron paramagnetic resonance (EPR) spectroscopy measurements of solutions of **3** in *o*-C_6_H_4_F_2_ (Fig. 3). Both, the isotropic *g*-value (*g*_iso_=2.0024) being close to that of the free electron and the low *g* anisotropy^[7a]^ speak in favour of a ligand-centred spin system. This assumption is further supported by the low hyperfine coupling constant *A*(^69^Ga)=21 MHz, thus being in good agreement with studies by Kaim *et al.*^47^ and clearly differing from the above mentioned metal-centred spin system [Ga^II^{B(N(C_6_H_3_-2,3-*i*Pr)CH)_2_}_2_] (cf. *A*(^69^Ga)=670 MHz)^45^.

Furthermore, we applied density functional theory (DFT) calculations to compute the singly occupied molecular orbitals (Fig. 2, inset) and the spin density of **3**^**2+**^ (Fig. 3, inset). We chose the hybrid B3LYP/SV(P) method, as it yielded good agreements between calculated and experimental bond lengths of **3**^**2+**^ (Fig. 2), and the main absorption maximum (*λ*_max_) in measured (302 nm) and simulated (296 nm) ultraviolet-visible spectra (Supplementary Fig. 5). These bands are reminiscent of the absorption of the related [Ru^3+^(bipy)_2_(bipy)^•^^−^)]^2+^ complex^51^ (cf. *λ*_max_=373 nm). Beyond featuring a similarly distorted octahedral coordination mode as its single-crystal congener, the singly occupied molecular orbitals of the geometry-optimized **3**^**2+**^ is solely located on the bipy ligands (Fig. 2) and only 3.0% of the spin is located at the gallium cation (Fig. 3). Overall, the experimental results (X-ray powder diffraction, EPR and ultraviolet-visible) and the DFT studies are in very good agreement and clearly assign a [Ga^III^{(bipy)_3_}^•^]^2+^ complex as correct formulation of **3**^**2+**^. To our knowledge, **3** is the first reported single-crystal structure of a p-block metal complex, featuring a bipy radical anion as ligand (cf. the few examples of alkali-metal salts of bipy radicals and dianions of Goicoechea *et al.* as well as Wieghardt's extensive work on transition-metal complexes of bipy)^52^,^53^.

### Reaction of [In^I^(C_6_H_5_F)_2_]^+^[Al(OR^F^)_4_]^−^ with bipy

**Figure 4:**
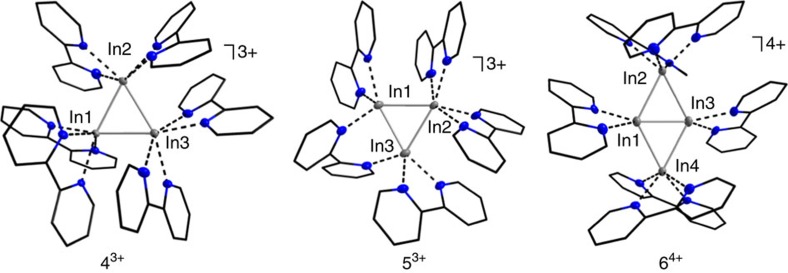
Molecular structures of indium clusters. Molecular structures of [In^I^_3_(bipy)_6_]^3+^ (**4**^**3+**^), [In^I^_3_(bipy)_5_]^3+^ (**5**^**3+**^) and [In^I^_3_(bipy)_6_]^4+^ (**6**^**4+**^). Selected bond lengths are given in pm and angles in °: **4**^**3+**^, In1−In2=266.07(4), In1−In3=266.98(5), In2−In3=278.12(5), av. In1−N=236.9(4), av. In2−N=249.0(4), av. In3−N=246.5(4) and ∢(In−In−In)=58.39(12)−62.90(12); **5**^**3+**^, In1−In2=267.89(5), In1−In3=266.80(5), In2−In3=269.84(5), av. In1−N=233.8(4), av. In2−N=234.0(4), av. In3−N=235.3(4) and ∢(In−In−In)=59.49(14)−60.62(14) (the asymmetric unit of **5** contains a second [In^I^_3_(bipy)_5_]^3+^ cluster featuring similar bonds lengths, Supplementary Table 4); **6**^**4+**^, In1−In2=275.93(14), In1−In3=259.65(12), In1−In4=276.62(14), In2−In3=280.83(14), In3−In4=278.56(14), av. In1−N=232.40(10), av. In2−N=236.33(10), av. In3−N=233.60(10), av. In4−N=236.10(10) and ∢(In−In−In)=55.59(3)−63.16(4) (the asymmetric unit of **6** contains four [In^I^_4_(bipy)_6_]^4+^ clusters, featuring similar In−In bonds lengths, Supplementary Table 5). The [Al(OR^F^)_4_]^–^ anions and all of the hydrogen atoms were omitted for clarity. The thermal ellipsoids are set at 50% probability. An alternative representation, featuring ellipsoids for all atoms, is included in Supplementary Fig. 6.

Due to the redox instability of **1**, we additionally reacted the heavier, more redox-stable homologue [In^I^(C_6_H_5_F)_2_]^+^[Al(OR^F^)_4_]^−^ (**2**) with bipy. While the isolation of single crystals from highly concentrated, yellowish solutions of **2** and bipy (2.00 eq or 1.63 eq) in C_6_H_5_F was straightforward, the composition of the obtained crystals depended on the amount of bipy employed as well as the crystallization procedure. Overall, we were surprised not to isolate any of the predicted [In(bipy)_1-3_]^+^ complexes (cf. Supplementary Table 9), but the very first homonuclear **cationic** triangular or rhombic In^I^ clusters: [In^I^_3_(bipy)_6_]^3+^{[Al(OR^F^)_4_]^−^}_3_ (**4**), [In^I^_3_(bipy)_5_]^3+^{[Al(OR^F^)_4_]^−^}_3_ (**5**) and [In^I^_4_(bipy)_6_]^4+^{[Al(OR^F^)_4_]^−^}_4_ (**6**). As above, the direct interaction between the cationic clusters and the [Al(OR^F^)_4_]^−^ anions is negligible and the latter are therefore not shown in Fig. 4. For the synthesis of **4**, 2.04 equivalents of bipy were applied. In **4**^**3+**^, each In^I^ cation is coordinated in a distorted octahedral fashion, or in other words, three tetragonal pyramidal N-coordinated [In^I^(bipy)_2_]^+^ fragments interact with each other, thus forming the observed triangular cationic In^I^ cluster. While the In1−In2 and In1−In3 bond lengths are very similar (266.07(4) and 266.98(5) pm, respectively), the In2−In3 distance is elongated by 11-12 pm. All three distances are well within the sum of the van der Waals radii^42^,^43^ (386 pm) and among the shortest compared to the manifold of reported organometallic and inorganic compounds that feature In−In bonds (Table 1 and Supplementary Table 15).

Reducing the amount of bipy from 2.00 to 1.63 equivalents, we obtained compound **5**. Though the molecular structure of **5**^**3+**^ resembles the one of **4**^**3+**^, one bipy ligand now acts as bridging N-ligand between two In^I^ cations, thus resulting in a more twisted arrangement of the two pyridine rings. These findings are likely attributable to the reduced amount of bipy employed and correspond well with the stoichiometry of the reaction (cf. ‘(5 bipy ligands)/(3 In^I^ cations)≈1.67'). Hence, only In2 is coordinated in a distorted octahedral fashion, while In1 and In3 are coordinated in distorted trigonal bipyramidal fashions. The very short In−In bond lengths (Table 1) are similar within 3 pm (av. In−In distance of 268.18(5) pm), thus resulting in an almost equilateral triangle. For **6**, 2.00 equivalents of bipy were applied. Yet, and different to the synthesis of **4**, the reaction mixture was additionally concentrated under reduced pressure, leading to the cationic, planar In^I^ rhomb **6**^**4+**^. While the coordination modes of In2 and In4 resemble the ones of the In^I^ cations in **4**^**3+**^ and In2 in **5**^**3+**^, In1 and In3 are pentacoordinated, interacting with only one bipy ligand and featuring three In−In contacts. The peripheral In−In bond lengths only deviate by 5 pm (av. In−In distance=277.99(14) pm) and are, with the exception of the In2−In3 bond of **4**^**3+**^, 10 pm longer than the In−In distances of their triangular congeners. The bridging In1−In3 distance on the other hand, is with 259.65(12) pm the shortest In−In bond that, to our knowledge, has been reported (the only shorter In−In bond derives from the structural relative seven, see below) (Table 1 and Supplementary Table 15).

Overall and despite the vast literature on compounds containing In−In bonds, the cationic molecular and univalent structures of **4**, **5** and **6** are unique. Somewhat related to **6** is the [In_4_{Cp_2_Mo_2_(CO)_4_P_2_}_8_]^4+^{[Al(OR^F^)_4_]^–^}_4_ salt reported by Scheer *et al.*^26^ Herein, the In^I^ cations form a similar rhombic arrangement, but with intermetallic distances that are at least 60 pm longer (shortest In−In distance: 348.2 pm). These findings must be due to the different ligand system, as the group used the same [Al(OR^F^)_4_]^–^ anion: that is, the interactions seem to be weakly dispersive rather than covalent. The remaining known cationic compounds are purely inorganic (cf. Supplementary Table 15) and while some of them feature similar In–In bond lengths, their chain-like substructures differ significantly. Though featuring a related geometry, the reported anionic triangular cyclogallanes differ in their electronic structures: that is, they are only accessible via reductive routes and feature delocalized electrons, resulting in a 2*π*-aromatic stabilization^54^,^55^. The dicationic rhombic tetraborane [B_4_H_2_(μ-hpp)_4_]^2+^ (hpp=1,3,4,6,7,8-hexahydro-2*H*-pyrimido[1,2-*a*]pyrimidinate) on the other hand, is structurally and electronically related to **6** (ref. 56). Finally, the cationic In^I^ clusters are not consistent with the Wade-Mingos rules (polyhedral skeletal electron pair theory), which are often used to rationalize clusters from group 13 (refs 2, 3, 57).

### Reaction of [In^I^(C_6_H_5_F)_2_]^+^[Al(OR^F^)_4_]^−^ with phen

**Figure 5:**
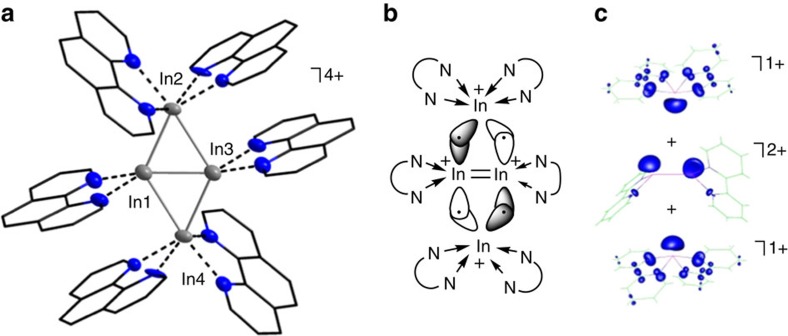
Molecular structure and bonding description of [In^I^_4_(phen)_6_]^4+^ (**7**^**4+**^) as well as spin density distributions of potential precursors. (**a**) Molecular structure of **7**^**4+**^. Selected bond lengths are given in pm, angles in ° and the values for **6**^**4+**^ are parenthesized: In1−In2=273.03(10) (275.93(14)), In1−In3=258.06(16) (259.65(12)), In1−In4=286.06(11) (275.93(14)), In2−In3=286.06(11) (280.83(14)), In3−In4=273.03(10) (278.56(14)), av. In1−N=231.2(9) (232.40(10)), av. In2−N=235.6(8) (236.33(10)), av. In3−N=231.2(9) (233.60(10)), av. In4−N=235.6(8) (236.10(10)) and ∢(In−In−In)=54.92(3)−65.11(3) (55.59(3)−63.16(4)). The [Al(OR^F^)_4_]^–^ anions and all of the hydrogen atoms were omitted for clarity. (**b**) A possible hypothetic description of the bonding situation in **6**^**4+**^ and **7**^**4+**^. The short bridging In1−In3 values could originate from interactions between the π*-orbitals of a dicationic, formally doubly bonded [(N-ligand)In=In(N-ligand)]^2+^ fragment (N-ligand=bipy, phen) and singly occupied orbitals of two [In(N-ligand)_2_]^+^ complexes, thus resulting in two two-electron three-centre bonds (2e3c), partially, but not fully, populating the antibonding π*-orbital. The remaining small In=In double-bonding contribution could account for the observed short In−In separations in **6**^**4+**^ and **7**^**4+**^ and also the relatively long 2e3c In−In bonds to the upper and lower [In(N-ligand)_2_]^+^ moieties. **c)** Calculated spin density distributions of triplet state fragments that could interact to form the observed cationic clusters (spin density cutoff at 0.010 a.u., B3LYP/SV(P) level). In the calculated [(bipy)In=In(bipy)]^3+^ fragment, the In−In distance is 291.4 pm and the average spin density on each indium atom 77% (a planar dicationic fragment did not converge, even if the conductor-like screening model (COSMO)^65^ was switched on and the permittivity was set to infinite *ɛ*_r_=∞). For triplet state of [In(bipy)_2_]^+^, the spin density on the indium atom is 35%.

While **3** and **4** reproduce the triangular [In_3_]^3+^ motif intrinsically, we additionally applied 1.49 equivalents of 1,10-phenanthroline (phen) to reproduce the rhombic [In_4_]^4+^ motif in **6**^**4+**^. In doing so, we were able to isolate a structural relative of **6**, that is, the [In^I^_4_(phen)_6_]^4+^{[Al(OR^F^)_4_]^−^}_4_ salt (**7**) (Fig. 5). The structural parameters of the [In^I^_4_(phen)_6_]^4+^ complex (**7**^**4+**^) resemble those of **6**^**4+**^, despite small *π*-*π*-interactions within the phen ligands (distances in pm, angles in °, values for **6**^**4+**^ are parenthesized): av. In−In=279.55(11) (277.99(14)), In1−In3=258.06(16) (259.65(12)), av. In−N=234.15(80) (235.14(10)), ∢(In−In−In)=54.92(3)−65.11(3) (55.59(3)−63.16(4)), no In−F contact to the [Al(OR^F^)_4_]^−^ anions. However, with 258.06(16) pm the bridging In1−In3 distance in **7**^**4+**^ is the shortest In−In bond length reported to this day. Both, the short bridging In1−In3 distances in **6**^**4+**^ and **7**^**4+**^, would be in agreement with the hypothetic interactions of a slightly trans-bent dicationic [(N-ligand)In=In(N-ligand)]^2+^ fragment and two [In(N-ligand)_2_]^+^ complexes (N-ligand=bipy, phen), thus resulting in two two-electron three-centre (2e3c) bonds (Fig. 5b).

**Figure 6:**
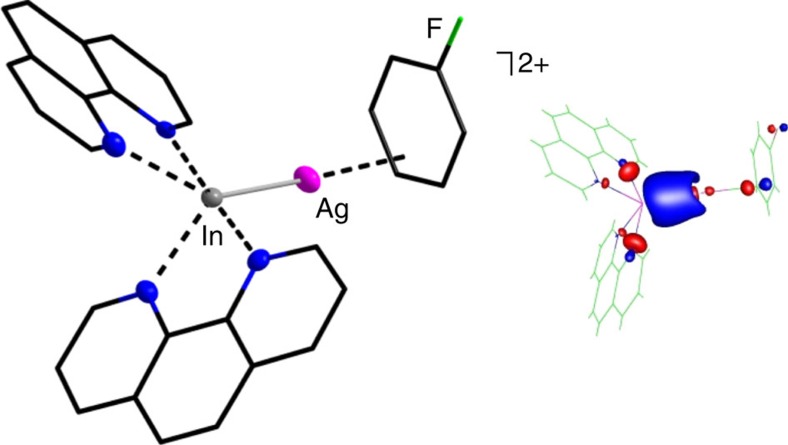
Molecular structure of [(phen)_2_In^I^−Ag^I^(C_6_H_5_F)]^2+^ (**8**^**2+**^). Selected bond lengths are given in pm and angles in °: av. In−N=233.3(3), In−Ag=256.37(5), Ag−cent=239.4 and In−Ag−cent=173.0 (cent=centroid of the *η*^3^-coordinating C_6_H_5_F; the linearity is in good agreement with earlier reported systems^70^). The [Al(OR^F^)_4_]^–^ anions and all of the hydrogen atoms were omitted for clarity. The thermal ellipsoids are set at 50% probability. An alternate formulation as [(phen)_2_In^I^−In^I^(C_6_H_5_F)]^2+^ diindane is not conceivable, as a [In^I^(C_6_H_5_F)]^+^ complex would be *η*^6^-coordinated. In addition, the X-ray diffraction refinement of the diindane formulation gave inferior R-factors and the DFT structure refinement did not converge. As an inset, the figure also contains the highest occupied molecular orbital (HOMO) of the converged **8**^**2+**^ structure. The latter features a constructive interaction between the occupied 5 s orbital of the [In^I^(phen)_2_]^+^ complex and the unoccupied 5 s/4d_z_2 hybrid orbital^70^ of the [Ag^I^(C_6_H_5_F)]^+^ complex. The calculated distances are in good agreement with experimental results (bond lengths in pm and angles in °): av. In−N=235.1, In−Ag=262.2, Ag−cent=253.4 and In−Ag−cent=175.0 (B3LYP/SV(P) level, electron density cutoff at 0.06 a.u.).

Apart from **7**, the reaction mixture also contained small amounts of a second type of single crystals, which, being colourless and not yellowish, surprisingly corresponded to the [(phen)_2_In^I^−Ag^I^(C_6_H_5_F)]^2+^{[Al(OR^F^)_4_]^−^}_2_ salt (**8**). To our knowledge, this is the first monomeric dicationic In−Ag adduct. While the Ag^I^ cation likely derives from the originally used Ag^+^[Al(OR^F^)_4_]^−^ salt (stemming from the synthesis of (**2**), the [(phen)_2_In^I^−Ag^I^(C_6_H_5_F)]^2+^ complex (**8**^**2+**^) can be considered as an addition product of the Lewis basic, tetragonal pyramidal coordinated [In^I^(phen)_2_]^+^ complex and the Lewis acidic, *η*^3^-coordinated [Ag^I^(C_6_H_5_F)]^+^ complex (cf. Δ_r_*H*°(gas)=−73/−84 kJ mol^−1^ and Δ_r_*G*°(gas)=−24/−36 kJ mol^−1^ for the formation of this gaseous dication (**!**) at 298.15 K, 1.0 bar, BHLYP/SV(P) and B3LYP/SV(P) level). Yet, multi-charged species in the gas phase are subject to strong repulsion and Coulomb explosion (v.s.). Thus, the surprisingly favourable Δ_r_*H*°(gas) and Δ_r_*G*°(gas) values for the formation of **8**^**2+**^ are probably attributable to the high Lewis basicity of the [In^I^(phen)_2_]^+^ complex. Fig. 6 contains the single-crystal structure as well as the highest occupied molecular orbital of the converged calculated **8**^**2+**^ structure.

### Multinuclear solution NMR spectroscopy

All obtained single crystals were dissolved in *o*-C_6_H_4_F_2_ and investigated by ^1^H, ^14^N, ^19^F, ^27^Al, ^71^Ga and ^115^In NMR spectroscopy. While the ^14^N, ^71^Ga and ^115^In NMR spectra featured no resonances, thus being in good agreement with earlier reported *σ*-coordinated complexes of Ga^I^ and In^I^ 28,29,31,32, one singlet in the ^19^F NMR and ^27^Al NMR spectra at −74.9 p.p.m. and +33.8 pm, respectively, revealed the intactness of the [Al(OR^F^)_4_]^−^ anions^58^. In the case of the mixed crystalline residue of **7** and **8**, the ^19^F NMR spectrum additionally featured the triplet of a triplet at −113 p.p.m. assigned to C_6_H_5_F, which likely derives from **8**^**2+**^. Finally, the ^1^H NMR spectra provided primary information on the stability of the obtained cationic complexes in solution: that is, the solution of **3** featured very weak and broad resonances due to the paramagnetic nature of **3**^**2+**^ (cf. EPR studies). Solutions of **4**, **5** and **6** featured multiplets attributable to solvated bipy, thus speaking for a fragmentation of the cationic indium clusters in solution. The solution of the crystalline residue of **7** and **8** yielded a complex multiplet pattern in the aromatic region, which likely is attributable to different fragments of both sets of single crystals. From the multinuclear NMR studies we suggest that the cationic In^I^ clusters are unstable in solution, while the [Al(OR^F^)_4_]^−^ anions stay intact. The dissociation of the cationic In^I^ clusters is probably attributable to the distinct Coulomb repulsion of the In−In bonded individual [In^I^(N-Ligand)_1,2_]^+^ units constituting **4**^**3+**^, **5**^**3+**^, **6**^**4+**^ and **7**^**4+**^ (cf. DFT studies below).

### Disproportionation versus cationic cluster formation

**Figure 7:**
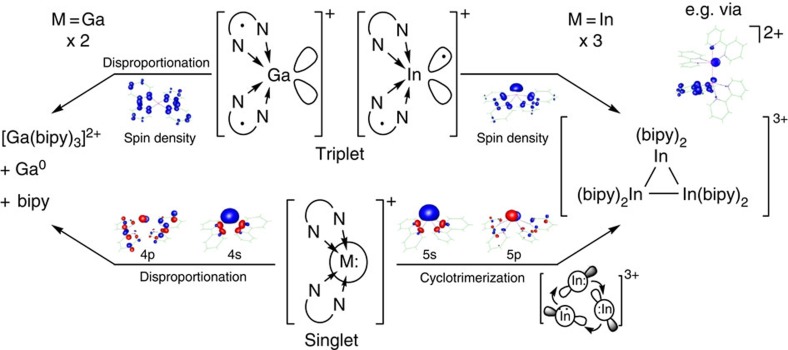
Metal-dependent behaviour of [M^I^(C_6_H_5_F)_2_]^+^[Al(OR^F^)_4_]^−^ (M=Ga, In) in the presence of bipy. Disproportionation (for M=Ga) and cluster formation (for M=In) can occur via (i) an ambiphilic singlet route (bottom) or (ii) a triplet route (top). Regarding (i), the [M^I^(bipy)_2_]^+^ complexes feature an occupied 4 s/5 s-orbital (HOMO) and empty 4p/5p-orbitals (LUMO+6), thus being able to react via a disproportionation or a cyclotrimerization, yielding **3**^**2+**^ and **4**^**3+**^, respectively. The conceivability of this reaction path directly correlates to the energy gap between the 4 s/5 s- and the 4p/5p-orbitals of the reactants (Table 2). Choosing reaction path (ii), the [M^I^(bipy)_2_]^+^ complexes react via their triplet state. Dependent on the metal, the latter significantly differ in terms of geometries and localization of spin densities. Hence, the [Ga(bipy)_2_]^+^ complex changes its coordination mode from tetragonal pyramidal in the singlet to tetrahedral in the triplet state and both unpaired electrons are equally distributed over the bipy ligands (spin density at the gallium atom=6.0%). These findings clearly speak for the tendency of the Ga^I^ cations to disproportionate and also explain the complex's inability to form a di-/oligo-gallane.^44^ The [In(bipy)_2_]^+^ complex on the other hand, retains its tetragonal pyramidal coordination mode and 35% of the spin density is located on the indium atom, making a stepwise cyclotrimerization feasible (cf. the dicationic [(bipy)_2_In−In(bipy)_2_]^2+^ cluster fragment shown). The conceivability of the reaction path directly correlates to the singlet-triplet gaps of the reactants (Table 2). The Kohn-Sham orbitals shown were selected on localization of the electron density on the metal cations and the lowest possible energy (electron and spin density cutoffs at 0.06 and 0.01 a.u., B3LYP/SV(P) level; as shown in Supplementary Fig. 10, the [(bipy)_2_In−In(bipy)_2_]^2+^ cluster fragment only featured a spin density at the indium atoms, if the BHLYP functional was applied).

To answer the question why **1** in the presence of bipy disproportionates and **2** functions as indium cluster source, as well as to elaborate a potential reaction pathway, we conducted further DFT calculations. From a retrosynthetic point of view, we chose the [M^I^(bipy)_2_]^+^ complex (M=Ga, In) as starting point, as the latter seems to be a crucial building block during the disproportionation and cluster formations: that is, in **4**^**3+**^, **5**^**3+**^ and **6**^**4+**^, six out of ten In^I^ cations are coordinated in such a manner. While this assumption is supported by the successful isolation of **7**^**4+**^ and **8**^**2+**^, the question remains, how the fragments interact with each other? (i) Via an ambiphilic route in which each [M(bipy)_2_]^+^ complex functions as a Lewis acid (empty 4p/5p orbitals) and as a Lewis base (occupied 4 s/5 s orbitals) (cf. the cyclopropane derivatives of the group 13 (ref. 59) and 14 homologues^60^) or ii) via a singlet-triplet route, that is, ligands such as *o*-quinones, N-hetero arenes and diazabutadienes have proven to be non-innocent^61^, thus promoting electron transfer reactions and a more easy access to the triplet states of the complexes^62^ (Fig. 7).

From an energetic point of view, the singlet-triplet route appears to be more conceivable, as the singlet-triplet gaps are distinctively smaller than the corresponding 4 s/5 s-4p/5p energy gaps (Table 2; cf. DFT studies by Macdonald *et al.*^63^). In addition and considering the distribution of spin densities, the triplet states of the [M(bipy)_2_]^+^ complexes offer important insights into the metal-dependent redox stabilities: that is, for gallium the tetrahedral [Ga^3+^{(bipy)^•−^}_2_]^+^ complex forms and for indium the tetragonal pyramidal [In^2+^(bipy)(bipy)^•−^]^+^ complex. Hence, only the latter should be able to stepwise cyclotrimerize, while the former is labile and disproportionates. In this context, the choice of the redox-active ligand is crucial and for indium, bipy seems to be the perfect match as it enables reversible, single electron transfers between the metal centre and the ligand, thus making way for the stepwise cationic metal atom cluster formation. For gallium, this is not the case and the bipy-located electrons are prone to intermolecular rather than intramolecular transfer reactions, resulting in a disproportionation of the [Ga(bipy)_2_]^+^ complex^64^.

Finally, we attempted to calculate the molecular structures of **4**^**3+**^, **5**^**3+**^ and **6**^**4+**^. Though we implemented the conductor-like screening model^65^ with an infinite permittivity and dispersive interactions (D3), the cationic In clusters fragmented due to the distinct Coulomb repulsion. However, we were able to calculate dicationic cluster fragments in their triplet state, such as [(bipy)_2_In−In(bipy)_2_]^2+^ (Fig. 7, inset). With an average spin-density distribution of 32% at each indium atom, the latter could be seen as a reaction intermediate of the univalent indium clusters, thus supporting a stepwise cluster formation (for further dicationic cluster fragments see Supplementary Fig. 10). Furthermore, we assessed the gas-phase thermodynamics (Δ_r_*H*°(gas), Table 3) of the formations of **4**^**3+**^, **5**^**3+**^ and **6**^**4+**^ from BHFCs and setting Δ_r_*H*°(solid) in a worst-case scenario to ±0 kJ mol^−1^ (Supplementary Fig. 8). The endothermic values are attributable to the above mentioned Coulomb repulsion and very well correspond to the large exothermicity of the Coulomb explosion of **4**^**3+**^ with formation of three [In(bipy)_2_]^+^ monocations in the gas phase and assessed via a suitable BHFC as −466 kJ mol^−1^ (Table 3 and Supplementary Fig. 8).

This is in agreement with single-point DFT calculations on the frozen conformation of solid **4**^**3+**^, Coulomb exploding to give three [In(bipy)_2_]^+^ monocations cut out of this cyclic trimer solid-state structure. B3LYP and BHLYP suggest this gas phase process to be favoured by −684 and −705 kJ mol^–1^, respectively. A non-ligand- supported triangular [In_3_]^3+^ cluster (*d*_In-In_=270 pm) was calculated to Coulomb explode at the same level with −1,447/–1,459 kJ mol^–1^, Supplementary Table 13). Overall, the formation of the ligand supported [In_3_]^3+^/[In_4_]^4+^ clusters seems to be only possible through the application of matching ligands and ultimately is a solid-state-driven phenomenon. Both, bipy and phen lead to a pronounced decrease of the Coulomb repulsion within the clusters by diluting the positive charges on the In^+^ cations to the ligand backbone, and contributing enough negative charge to yield ligand stabilized [In_3_]^3+^(A^−^)_3_ and [In_4_]^4+^(A^−^)_4_ salts with short In−In bonds. This corresponds to a ligand-to-metal charge transfer. The calculated high Δ_latt_*H*° values of −1,438 (**4**), −1,444 (**5**) and −2,266 kJ mol^−1^ (**6**) further stabilize the salts^66^. Together, the charge transfer leading to favourable metal-metal bond strengths, in combination with the lattice enthalpy gain are sufficient to overcome the strong Coulomb repulsion active in gaseous and solution phases. Last, it should be noted that for the central [In_4_]^4+^ cluster core with eight valence electrons deriving from four In^I^ cations, the stability of **6**^**4+**^ and **7**^**4+**^ would be in agreement with the Jellium model^67^.

## Discussion

The reaction of **1** and 2,2'-bipyridine resulted in a disproportionation of the former, thus yielding the monomeric and paramagnetic [Ga(bipy)_3_]^2+•^ complex. Herein, the gallium cation is coordinated in a distorted octahedral fashion, and EPR and DFT studies reveal a ligand-centred radical: that is, a [Ga^III^{(bipy)_3_}^•^]^2+^ complex. Applying the heavier homologue **2** on the other hand, we isolated the first homonuclear cationic triangular and rhombic clusters of univalent indium: [In^I^_3_(bipy)_6_]^3+^, [In^I^_3_(bipy)_5_]^3+^, [In^I^_4_(bipy)_6_]^4+^ and [In^I^_4_(phen)_6_]^4+^. Herein, the In^I^ cations are coordinated by one, 1.5 or two chelating bipy/phen ligands. To our knowledge, the In−In distances (258.1 and 259.7 pm) within the In−In bridges in the rhombic clusters are the shortest that have been reported so far. DFT studies suggest a stepwise formation of the clusters via their triplet state and an alternate ambiphilic route seems to be energetically less favourable. The general driving force for this cationic cluster formation is attributable to relatively strong In−In bonds, reduction of Coulomb repulsion by introduction of a suitable ligand and ligand-to-metal charge transfer in combination with the high lattice enthalpies of the resultant ligand stabilized [M_3_]^3+^(A^−^)_3_ and [M_4_]^4+^(A^−^)_4_ salts. We are convinced that this is a general phenomenon, which could be used as a pathway to cationic metal atom cluster formation of subvalent metal cations in combination with strong but sterically accessible (chelating?) ligands.

## Methods

### General experimental procedures

All manipulations were performed using Schlenk or glove box techniques in an argon atmosphere (H_2_O and O_2_<1 p.p.m.). *o*-C_6_H_4_F_2_ and C_6_H_5_F were dried over CaH_2_, distilled and had H_2_O contents below 5 p.p.m. (Karl-Fischer titrations). Because the obtained compounds contain large amounts of fluorine in chemically very stable CF_3_ groups, standard combustion analyses have proven to be incomplete. Characterizations of novel compounds were therefore done on the basis of single-crystal X-ray analysis and multinuclear NMR spectroscopy. As the highly symmetric and perfluorinated [Al(OR^F^)_4_]^−^ anions are usually heavily disordered, prone to crystallize in superstructures (cf. compound **3**) and, due to their bulkiness, able to add up to very large unit cells (the unit cell sizes of **6** and the protein Viscotoxin B are comparable, Supplementary Table 8), processing of the crystal structure data were everything else than trivial. In this context, however, the quality of the data which were collected using a completely up to date crystallography is clearly well within the limits of accepted standards, thus allowing us to unambiguously identify all structures. Moreover, all compounds were reproduced from independent syntheses (apart from **5** and **6**) and **4**, **5**, **6** and **7** intrinsically confirm the central structural triangular [In_3_]^3+^ and rhombic [In_4_]^4+^ motifs. Therefore, access to international facilities for better quality X-ray diffraction data were not sought for. Further details are included in the Supporting Information. Moreover, it was several times attempted to obtain ESI–MS data of the reported systems. As the ionic compounds are very sensitive however, no meaningful spectra were obtained—presumably due to oxidation and/or hydrolysis on the way to the ionization chamber (as very frequently encountered with our sensitive systems). Since the other investigations strongly suggested these multiply charged cations to only exist in the solid state, this method was not further pursued. Compound **3** was additionally characterized by EPR and ultraviolet-visible measurements. The DFT calculations were performed at the BHLYP/SV(P) and B3LYP/SV(P) level of theory.

## Additional information

**Accession codes**: The X-ray crystallographic coordinates for structures reported in this study have been deposited at the Cambridge Crystallographic Data Centre (CCDC), under deposition numbers CCDC 1032681 (**3**), CCDC 1032680 (**4**), CCDC 1033040 (**5**), CCDC 1032732 (**6**), CCDC 1034231 (**7**) and CCDC 1034089 (**8**). These data can be obtained free of charge from The Cambridge Crystallographic Data Centre via www.ccdc.cam.ac.uk/data_request/cif.

**How to cite this article:** Lichtenthaler, M. R. *et al.* Cationic cluster formation versus disproportionation of low-valent indium and gallium complexes of 2,2'-bipyridine. *Nat. Commun.* 6:8288 doi: 10.1038/ncomms9288 (2015).

## Supplementary Material

Supplementary Information Supplementary Figures 1-10, Supplementary Tables 1-15, Supplementary Methods and Supplementary References

Supplementary Data 1 Cif File for Structure 3

Supplementary Data 2 Cif File for Structure 4

Supplementary Data 3 Cif File for Structure 5

Supplementary Data 4 Cif File for Structure 6

Supplementary Data 5 Cif File for Structure 7

Supplementary Data 6 Cif File for Structure 8

Supplementary Data 7 Coordinates and Frequencies of all calculated Compounds

## Figures and Tables

**Figure 1:**
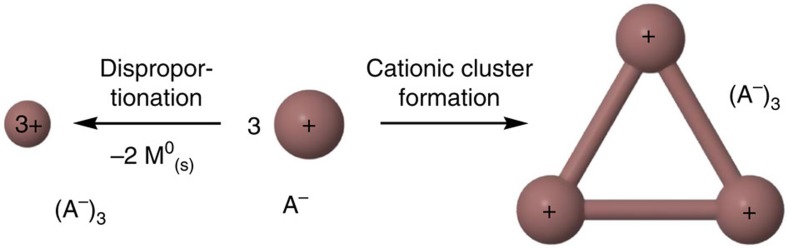
Disproportionation versus cationic cluster formation of univalent group 13 metal salts M^+^A^–^. Generally, the disproportionation is favoured over the cluster formation due to the much higher lattice energies of the M^3+^(A^−^)_3_ salt.

**Table 1:** Selection of organometallic and inorganic indium compounds, featuring at least one In−In bond (*d*(In−In) in pm).

**Compound**	***d*****(In−In) (pm)**	**Comment**
[In_4_(phen)_6_]^4+^{[Al(OR^F^)_4_]^–^}_4_ (**7**)	258.1–286.1*	this work
[In_4_(bipy)_6_]^4+^{[Al(OR^F^)_4_]^–^}_4_ (**6**)	259.7–280.8*	this work
In_5_Mo_18_O_28_ (In_5_-moieties)	261.6–266.5	—†
In_11_Mo_40_O_62_ (In_5_- and In_6_-moieties)	262.4–268.9	—†
In_3_(PO_4_)_2_ (In_2_-dimers)	263.0	—†
[In{C(Si(Me_3_)_3_}{{OC(C_6_H_5_)}_2_CH}]_2_	264.6–279.3	—†
[In{(O_2_CPh)C(SiMe_3_)_3_}]_2_	265.4	—†
[In{N(C_6_H_2_-2,4,6-Me_3_)CH}_2_O(SO_2_)(CF_3_)]_∞_	265.6, 266.5	—†
[In_3_(bipy)_6_]^3+^{[Al(OR^F^)_4_]^–^}_3_ (**4**)	266.1–278.1	this work
[In_3_(bipy)_5_]^3+^{[Al(OR^F^)_4_]^–^}_3_ (**5**)	266.8–269.8	this work
In_5_S_4_ (In_5_-moieties)	276.2–276.9	—†
In_4_Se_3_ (In_3_-moieties)	275.6–277.6	—†
InSe (In_2_-dimers)	281.8	—†
In_2_O(PO_4_) ([In_2_]^4+^[In_2_O_2_(PO_4_)_2_]^4−^)	286.2	—†
In−In (metal)	325.2, 337.7	—†
[In_4_{Cp_2_Mo_2_(CO)_4_P_2_}_8_]^4+^{[Al(OR^F^)_4_]^–^}_4_	348.2–396.0	—†
InCl (distorted In_4_-tetrahedra)	359.1–476.3	—†

The entries are ordered in terms of increasing *d*(In−In) values. Supplementary Table 15 contains a comprehensive compilation.

^*^The very short In−In bond lengths (258.1 and 259.7 pm) correspond to the bridging In1−In3 distances within the rhombic **6**^**4+**^ and **7**^**4+**^.

^†^The references are included with Supplementary Table 15.

**Table 2:** Energy gaps between the occupied 4 s/5 s- and unoccupied 4p/5p-orbitals as well as singlet-triplet gaps of [M(C_6_H_5_F)_2_]^+^, [M(bipy)]^+^ and [M(bipy)_2_]^+^ in kJ mol^−1^ (M=Ga, In; gas phase, 298.15 K, 1.0 bar, values at BHLYP/SV(P)/B3LYP/SV(P) level).

	**Δ*****E*** **(kJ** **mol**^**−1**^**) (M=Ga)**		**Δ*****E*** **[kJ** **mol**^**−1**^**] (M=In)**	
	**4 s/4p**	**singlet-triplet**	**5 s/5p**	**singlet-triplet**
[M(C_6_H_5_F)_2_]^+^	853/653	311/-*	797/618	-*/-†
[M(bipy)]^+^	582/386	196/-†	580/383	227/-†
[M(bipy)_2_]^+^	709/507	5‡/21‡	645/471	111§/96§

^*^Even after 500 iteration cycles, the geometry optimization of the triplet state did not converge.

^†^Though the geometry optimization converged, the electronic occupation of the triplet state is not correctly described.

^‡^The significant decrease of Δ*E* is accompanied by a geometry change of the [Ga(bipy)_2_]^+^ complex from a tetragonal pyramidal to a tetrahedral coordination mode in the singlet and triplet state, respectively (Supplementary Table 12).

^§^The [In(bipy)_2_]^+^ complex features a tetragonal pyramidal coordination mode both in the singlet and the triplet state (Supplementary Table 12).

**Table 3:** Estimated Δ_r_ *H*°(gas) values for the formation of **4** ^**3+**^, **5** ^**3+**^ and **6** ^**4+**^ as well as for the Coulomb explosion of **4** ^**3+**^ in kJ mol^−1^.

**Gas phase reaction**	**Δ**_**r**_***H*****°(gas) [kJ** **mol**^**–1**^**]**
3 [In(PhF)_2_]^+^+6 bipy → [In_3_(bipy)_6_]^3+^+6 PhF	+164
3 [In(PhF)_2_]^+^+5 bipy → [In_3_(bipy)_5_]^3+^+6 PhF	+252
4 [In(PhF)_2_]^+^+6 bipy → [In_4_(bipy)_6_]^4+^+8 PhF	+731
[In_3_(bipy)_6_]^3+^ → 3 [In(bipy)_2_]^+^	−466

The Δ_r_*H*°(solid) values for all BHFCs were deliberately set to ±0 kJ mol^−1^, the Δ_latt_*H*° values calculated by applying the Jenkins generalized Kapustinskii equation^66^ and all other enthalpies extrapolated from the given references (Supplementary Fig. 8).
