# Supplementary material for: Cationic cluster formation versus disproportionation of low-valent indium and gallium complexes of 2,2'-bipyridine
Source: Nat Commun. 2015 Oct 19;6:8288. doi: 10.1038/ncomms9288 (PMC4633986; doi:10.1038/ncomms9288)
Supplement: Supplementary Information — Supplementary Figures 1-10, Supplementary Tables 1-15, Supplementary Methods and Supplementary References [file ncomms9288-s1.pdf]

1. Supplementary Figures

1.1. Multinuclear NMR spectra of 3, 4, 5, 6, 7 and 8

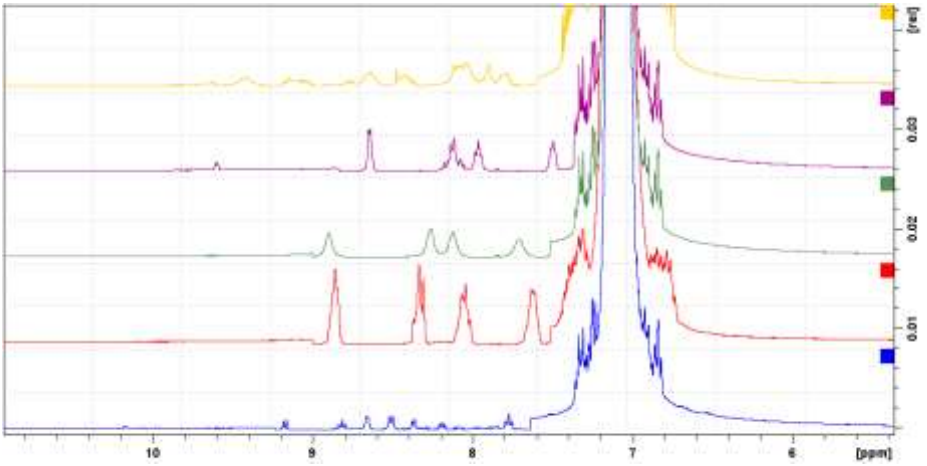

Supplementary Figure 1: ^{1}H NMR spectra (400 MHz, *o*-$C_6H_4F_2$, calibrated to *o*-$C_6H_4F_2$ = 7.12 ppm[1], 298 K) of **3** (blue), **4** (red), **5** (green), **6** (purple), **7** and **8** (yellow); 64 times magnified. The resonances in the aromatic region are attributable to the bipy, phen and C_6H_5F ligands. The different chemical shifts likely derive from various coordination modes to the gallium and indium cations.

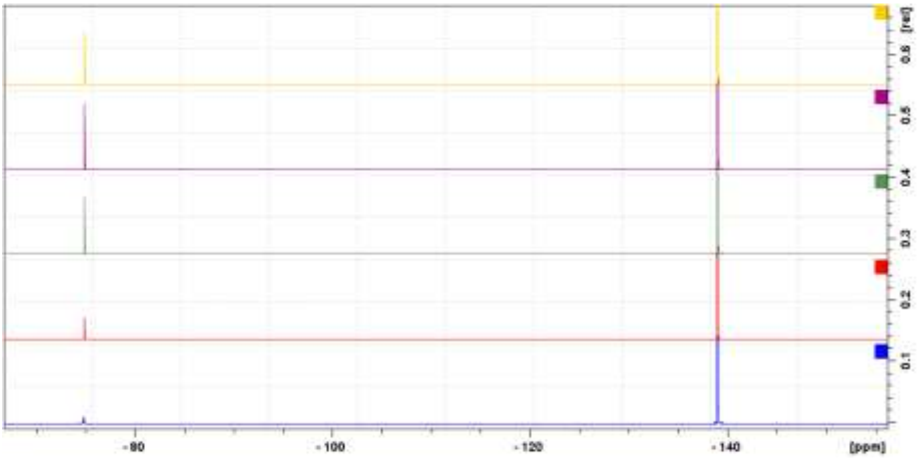

Supplementary Figure 2: ^{19}F NMR spectra (377 MHz, *o*-$C_6H_4F_2$, calibrated to *o*-$C_6H_4F_2$ = –139 ppm[2], 298 K) of **3** (blue), **4** (red), **5** (green), **6** (purple), **7** and **8** (yellow); eight times magnified. The resonances at −75 pm are attributable to intact $[Al(OR^F)_4]^-$ anions.

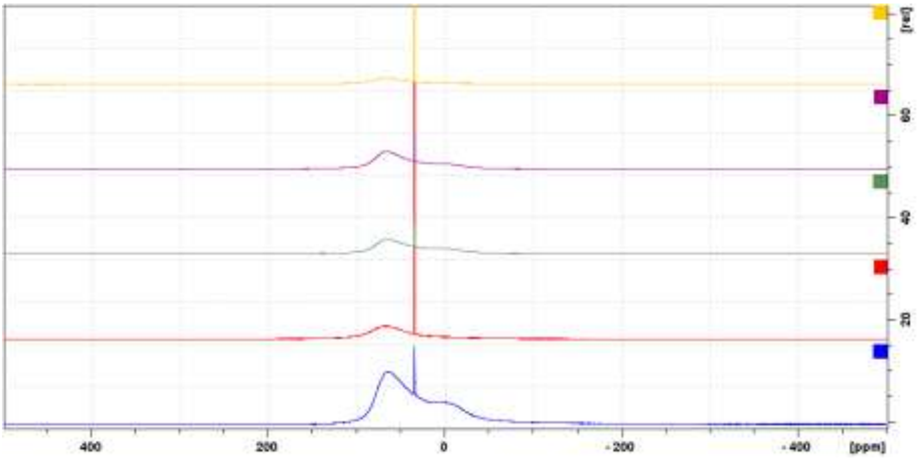

Supplementary Figure 3: ^{27}Al NMR spectra (104 MHz, *o*-$C_6H_4F_2$, calibrated to $[Al(OR^F)_4]^-$ = 33.8 ppm[3], 298 K) **3** (blue), **4** (red), **5** (green), **6** (purple), **7** and **8** (yellow). The resonances at 33.8 pm are attributable to intact $[Al(OR^F)_4]^-$ anions.

The ^{14}N, ^{71}Ga and ^{115}In NMR spectra did not show any resonance.

1.2. EPR Spectra of Frozen Solutions of 3 in C_6H_5F and *o*-$C_6H_4F_2$

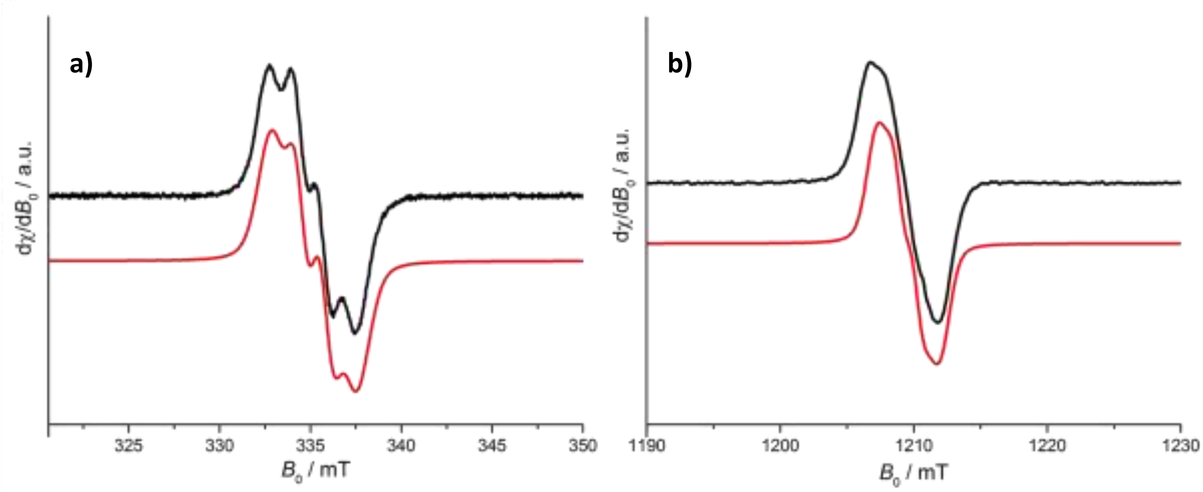

Supplementary Figure 4: a) X-band EPR measurement of a frozen solution of 3 in C_6H_5F (1 mM) at 120 K (black trace; microwave frequency 9.395 GHz, magnetic-field modulation 0.1 mT, microwave power 63 µW) and the corresponding simulation (red trace). b) Q-band EPR measurement of frozen solution of 3 in o-$C_6H_4F_2$ (0.5 mM) at 80K (black trace; microwave frequency 33.901 GHz, magnetic-field modulation 0.1 mT, microwave power 9.15 µW) and the corresponding simulation (red trace). The X-band EPR measurement showed a S = 1/2 structure coupled to a I = 3/2 gallium nucleus, which resulted in a pronounced quartet hyperfine structure. Neither the X-band nor the Q-band measurements showed a well resolved g-tensor. Both spectra were simulated with an isotropic g-value (g_{iso} = 2.0024) and an anisotropic gallium hyperfine coupling. The good agreement between experiment and simulations supports our assumption of a g-tensor of low anisotropy.

1.3. UV-VIS Spectrum of a Solution of 3 in *o*-$C_6H_4F_2$

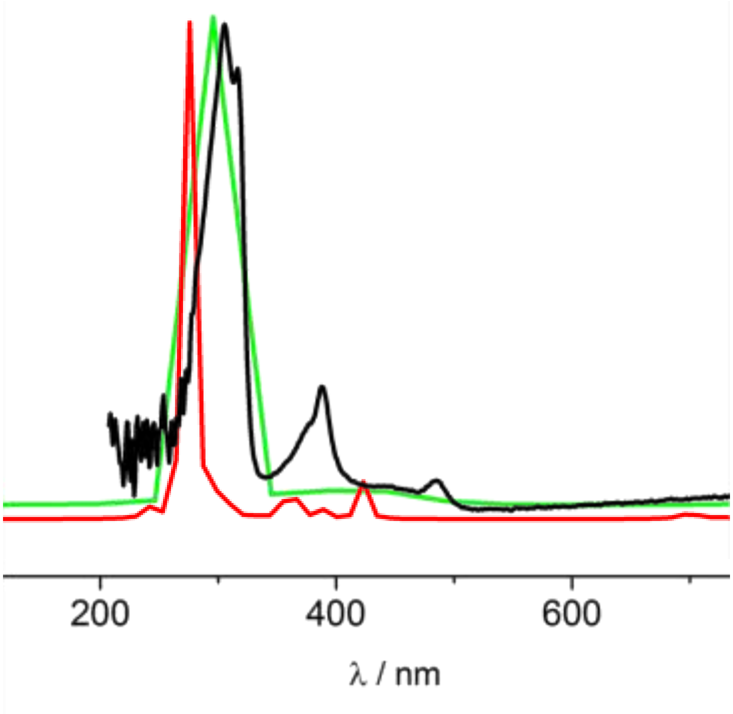

Supplementary Figure 5: UV-VIS spectrum of a solution of **3** in *o*-$C_6H_4F_2$ (333 µM) (black trace) and simulated excitations for the $[Ga(bipy)_3]^{2+}$ complex (gas phase, 298.15 K, 1.0 bar, B3LYP/SV(P) (green trace) and BHLYP/SV(P) level (red trace)). The absorption maxima (λ_{max}) are given in nm and the values of the simulations are parenthesized: 302 (296, 272), 313 (363), 384 (385), 481 (420).

1.4. Molecular Structures of 3^{2+}, 4^{3+}, 5^{3+}, 6^{4+}, 7^{4+}, 8^{2+}

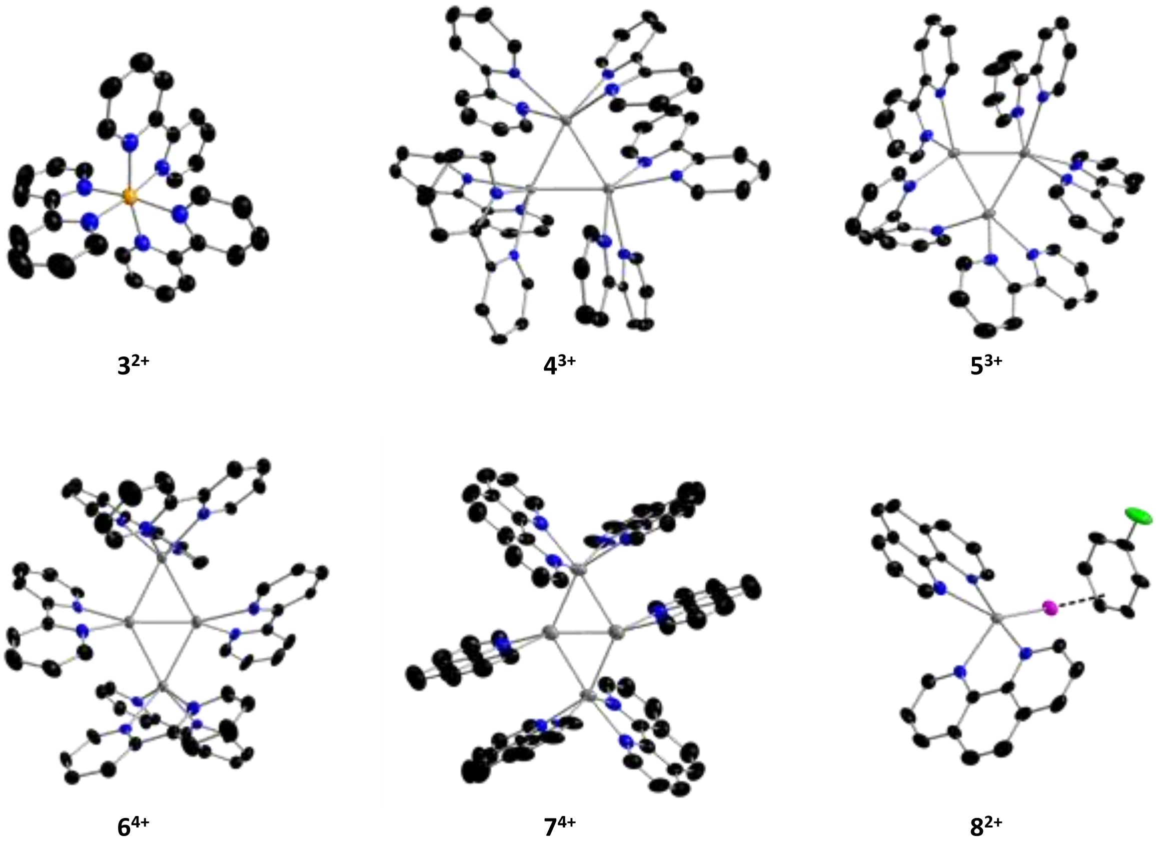

Supplementary Figure 6: Molecular structures of $\mathbf{3}^{2+}$, $\mathbf{4}^{3+}$, $\mathbf{5}^{3+}$, $\mathbf{6}^{4+}$, $\mathbf{7}^{4+}$, $\mathbf{8}^{2+}$. The $[Al(OR^F)_4]^-$ anions and all of the hydrogen atoms were omitted for clarity. The thermal ellipsoids are set at 50% probability.

1.5. Spin Density Distribution in $[Ga(bipy)_3]^{2+\bullet}$

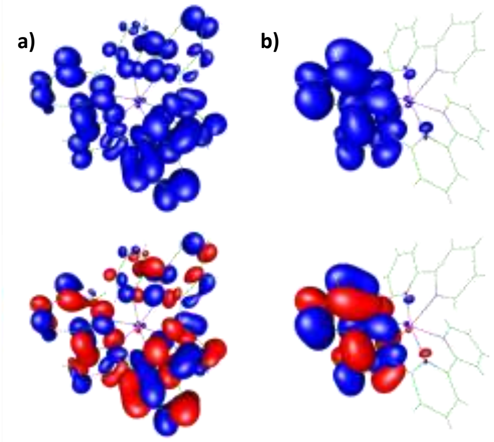

Supplementary Figure 7: Modelled spin density distribution and singly occupied molecular orbital of the $[Ga(bipy)_3]^{2+\bullet}$ complex (gas phase, 298.15 K, 1.0 bar, spin and electron density cut off at 0.001 and 0.03 a.u., respectively). The B3LYP/SV(P) level (a) corresponds to 3.0% spin density at the gallium atom and the BHLYP/SV(P) (b) level and 1.3%. As expected, a decrease in the functional's Hartree Fock exchange (BHLYP: 50% HF exchange, B3LYP: 20% HF exchange) led to a decreased localization of the spin density. In both cases however, the spin density is primarily ligand-centered.

1.6. Born-Haber-Fajans Cycles (BHFCs) concerning the Formation of 4^{3+}, 5^{3+} and 6^{4+}, the Coulomb Explosion of 4^{3+} and the Disproportionation of $Al^{+}Cl^{-}$ and $In^{+}Cl^{-}$

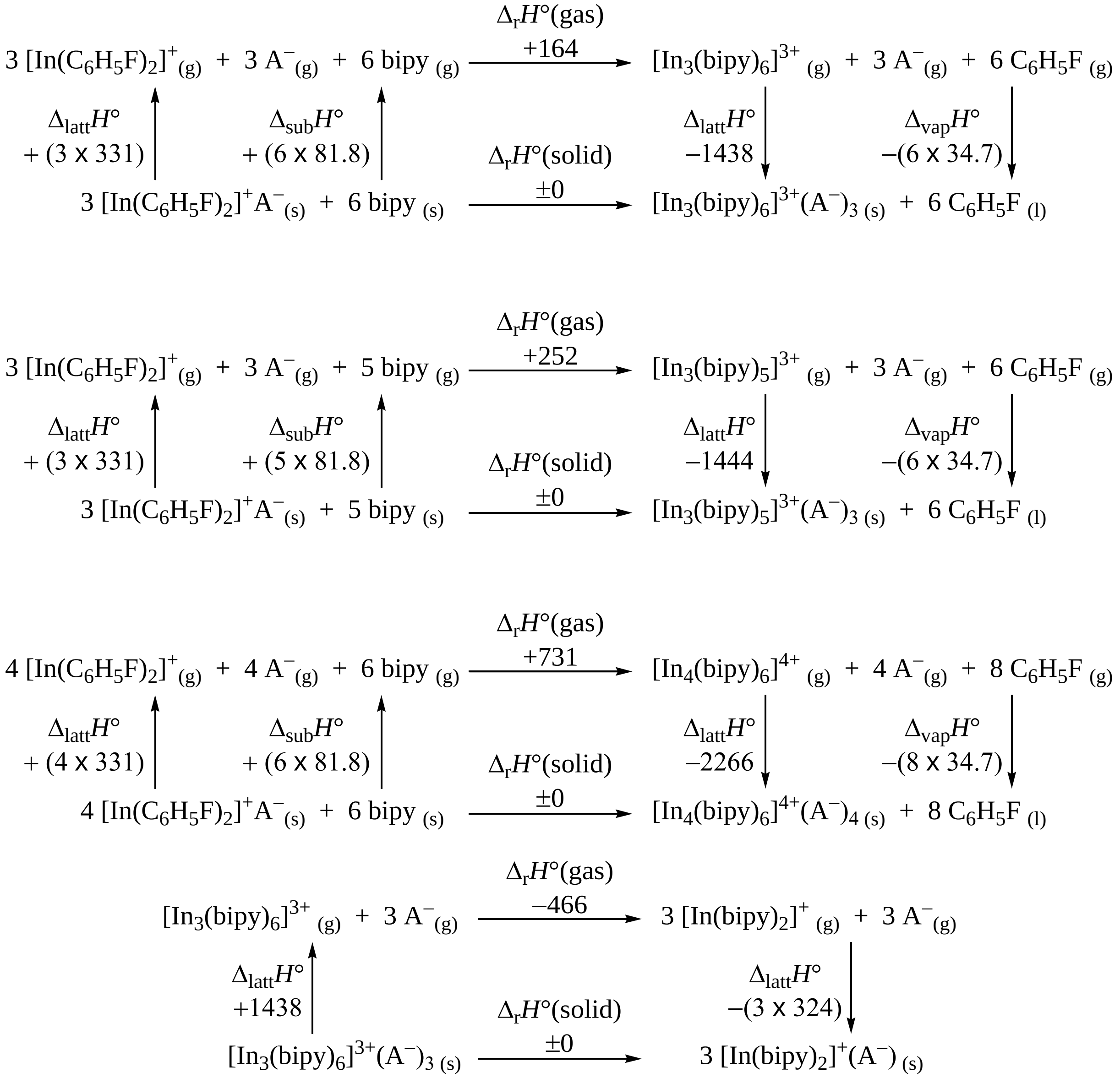

Supplementary Figure 8: Born-Haber-Fajans cycles to estimate the $\Delta_r H°$(gas) values for the formation of $\mathbf{4}^{3+}$, $\mathbf{5}^{3+}$ and $\mathbf{6}^{4+}$ as well as for the Coulomb explosion of $\mathbf{4}^{3+}$ (A^{-} = $[Al(OR^{F})_4]^{-}$). The $\Delta_r H°$(solid) values were deliberately set to ±0 kJ mol^{-1}. The lattice enthalpies ($\Delta_{latt}H°$) of the gallium(I) salts were calculated using the Jenkins generalized Kapustinskii equation[4] and the ion volumes derived from the single-crystal structures of **4**, **5** and **6**. $\Delta_{latt}H°$ of the theoretical $[In(bipy)_2]^{+}[Al(OR^{F})_4]^{-}$ salt was estimated by applying a third of the molecular volume of **4**. $\Delta_{sub}H°$ for bipy[5] and $\Delta_{vap}H°$ for C_6H_5F[6] were extrapolated from the given references. All enthalpies are given in kJ mol^{-1}.

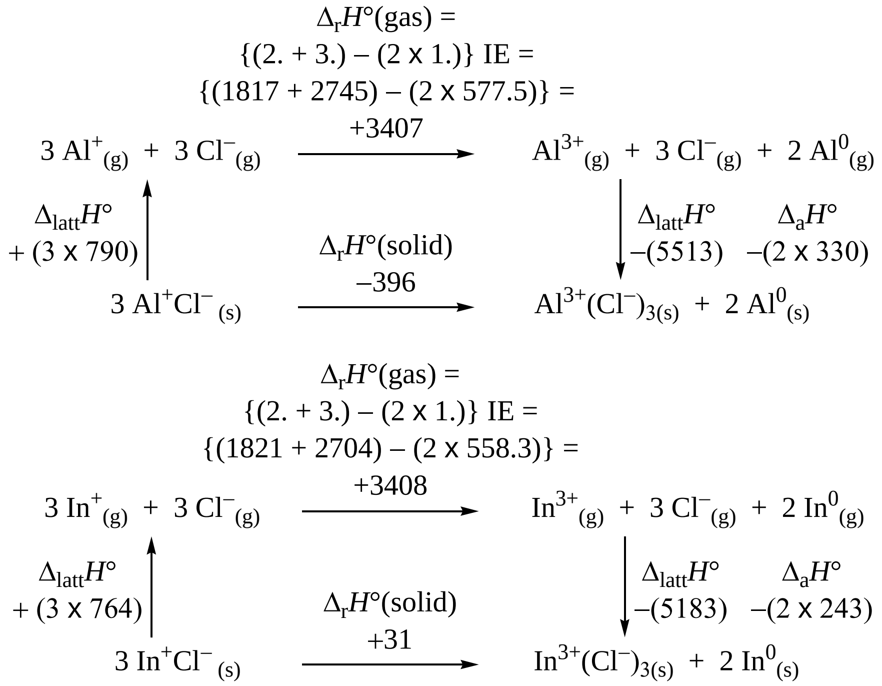

Supplementary Figure 9: Born-Haber-Fajans cycles to estimate the $\Delta_r H°$(solid) values for the disproportionation of AlCl and InCl, respectively. The $\Delta_{latt}H°$ values of, $AlCl_3$ (5513), InCl (764) and $InCl_3$ (5183) result from thermochemical cycles and were taken from the *CRC Handbook of Chemistry and Physics*, 87th Edition, **2006/2007** with the exception of unknown AlCl (790), for which we set: $\Delta_{latt}H°$(AlCl) ≈ $\Delta_{latt}H°$(NaCl). The $\Delta_r H°$(gas) values were calculated by applying a combination of the first, second and third ionization energies (IE). The IE values and atomization enthalpies ($\Delta_a H°$) were extrapolated from C. E. Housecroft, A. G. Sharpe, *Inorganic Chemistry*, 2nd Ed., **2005**. All enthalpies are given in kJ mol^{-1}.

1.7. Triplet States of Dicationic Cluster Fragments and their Spin Density Distributions

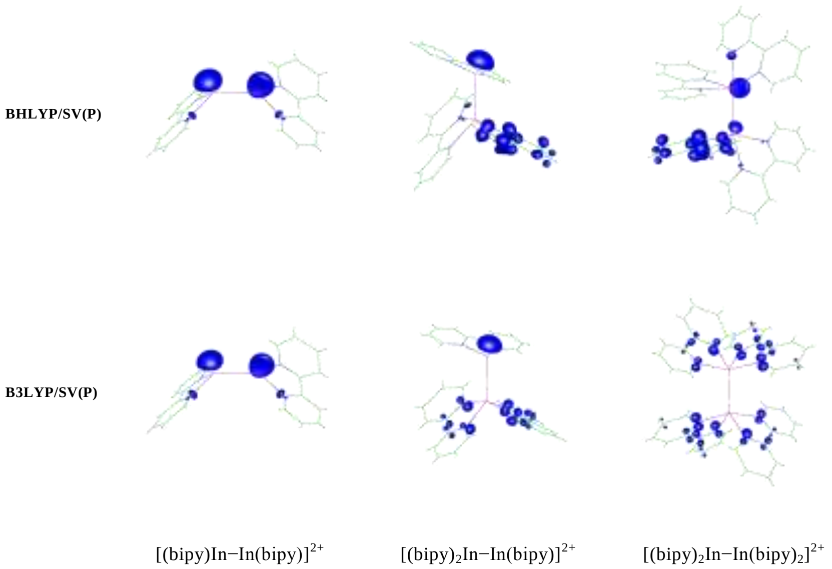

Supplementary Figure 10: Triplet states of dicationic univalent indium cluster fragments, average spin density distributions at the indium atoms and In–In bond distances. $[(bipy)In{-}In(bipy)]^{2+}$: 84% / 77% and 287.8 pm / 291.4 pm; $[(bipy)In{-}In(bipy)_2]^{2+}$: 43% / 42% and 295.2 pm / 296.2 pm; $[(bipy)_2In{-}In(bipy)_2]^{2+}$: 32% / 3% and 295.2 pm / 286.7 pm. The most notable differences between the BHLYP and B3LYP functional is the spin density distribution in the $[(bipy)_2In{-}In(bipy)_2]^{2+}$ complex: i.e., the BHLYP functional yields an average spin density distribution at the indium atoms of 32% and the B3LYP functional of only 3%. This might be a result of the decrease in the functional's Hartree Fock exchange (BHLYP: 50% HF exchange, B3LYP: 20% HF exchange) which generally leads to a decreased localization of spin density. Gas phase, 298.15 K, 1.0 bar, spin density cut off at 0.01 a.u., BHLYP/SV(P) and B3LYP/SV(P) level).

2. Supplementary Tables

2.1. Summary of the crystallographic data for 3, 4, 5 and 6.

Supplementary Table 1: Summary of the crystallographic data collections and refinement details for the bipy containing single-crystal structures: **3**, **4**, **5** and **6**.

	**3**	**4**	**5**	**6**
Formula	$C_{68}H_{27}Al_2F_{73.75}GaN_6O_8$	$C_{108}H_{48}Al_3F_{108}In_3N_{12}O_{12}$	$C_{422}H_{184}Al_{12}F_{437}In_{12}N_{40}O_{48}$	$C_{124}H_{48}Al_4F_{144}In_4N_{12}O_{16}$
M_w [g mol^{-1}]	2580.88	4182.98	16586.68	5264.94
T [K]	150(2)	100(2)	100(2)	100(2)
crystal system	monoclinic	monoclinic	triclinic	triclinic
space group	C2/c	$P2_1/c$	*P*1;¯	*P*1;¯
a [Å]	25.1999(16)	22.4009(11)	16.2891(6)	34.744(5)
b [Å]	15.4651(16)	30.7088(16)	25.6709(9)	34.792(5)
c [Å]	24.792(2)	20.4177(10)	33.2531(12)	37.210(5)
α [°]	90	90	89.470(2)	106.127(4)
β [°]	112.931(7)	95.459(2)	87.358(2)	101.609(4)
γ [°]	90	90	81.522(2)	118.938(3)
V [Å^3]	8898.3(14)	13981.7(12)	13738.4(9)	34671(8)
Z	4	4	1	8
ρ_{calc} [g cm^{-3}]	1.927	1.987	2.005	2.017
µ [mm^{-1}]	0.541	0.716	0.729	0.763
F(000)	5047	8136.0	8057.0	20384.0
crystal size [mm^3]	0.20 × 0.13 × 0.06	0.37 × 0.21 × 0.21	0.13 × 0.11 × 0.06	0.20 × 0.15 × 0.10
Θ range for data coll. [°]	3.164 to 50.282	1.826 to 55.56	2.016 to 55.22	1.888 to 49.008
index ranges	-30 ≤ h ≤ 30<br>-17 ≤ k ≤ 18<br>-29 ≤ l ≤ 29	-29 ≤ h ≤ 29<br>-39 ≤ k ≤ 39<br>-26 ≤ l ≤ 26	-21 ≤ h ≤ 21<br>-33 ≤ k ≤ 33<br>-43 ≤ l ≤ 43	-39 ≤ h ≤ 40<br>-40 ≤ k ≤ 39<br>-43 ≤ l ≤ 43
reflections collected	99261	206231	351052	566584
independent reflections	7923	32673	62800	111628
R_{int}	0.0422	0.0877	0.0492	0.0961
data/restraints/ parameters	7923/10335/ 1101	32673/82692/ 3337	62800/335118/ 5878	111628/927436/ 11257
absorption correction	multi-scan	multi-scan	multi-scan	multi-scan
T_{min}	0.6995	0.6307	0.7081	0.5947
T_{max}	0.7452	0.7456	0.7456	0.7451
GOF on F^2	1.017	1.204	1.024	1.078
final *R* indeces [*I* > = 2σ (*I*)]				
R_1	0.0752	0.0680	0.0655	0.0740
wR_2	0.1864	0.1492	0.1519	0.1691
final *R* indeces [all data]				
R_1	0.1021	0.0755	0.1043	0.1550
wR_2	0.2063	0.1530	0.1709	0.2157
largest diff. peak/hole [e Å^{-3}]	0.74/-0.69	1.63/-1.35	1.74/-1.43	2.64/-1.81

The low data/restraints/parameter ratios derive from the heavily disordered $[Al(OR^F)_4]^-$ anions within the structures. Though the ratios can be significantly improved by removing the anions and assessing the resultant voids with the program SQUEEZE[7] in PLATON[8], the procedure hardly influenced the *R* values and more importantly the geometry of the remaining cationic structures. We therefore left the anions within the structure refinement. The negligibility of the cation anion interaction is best shown by the shortest M−F contacts (M = Ga, In): i.e., for **3** (479.7 pm), **4** (467.0 pm), **5** (380.9 pm), **6** (449.1 pm), **7** (408.5 pm) and **8** (400.9 pm) the contacts are significantly longer than sum of the van der Waals radii (Ga−F = 334 pm; In−F = 340 pm).[9]

2.2. Crystal Structure Data for $[Ga(bipy)_3]^{2+}\{[Al(OR^F)_4]^-\}_2$ (3)

Supplementary Table 2: Crystal data and structure refinement for **3**.

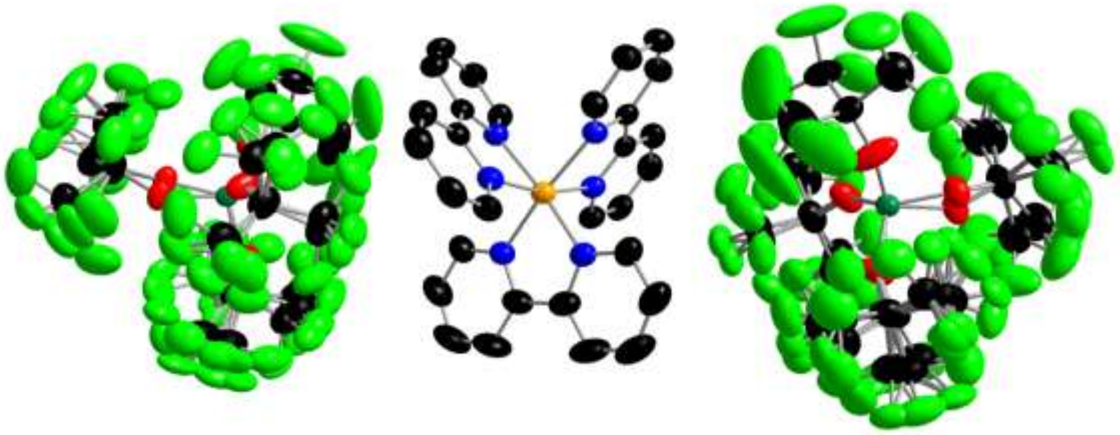

CCDC Deposition Number: 1032681

Identification code	**3**
Empirical formula	$C_{68}H_{27}Al_2F_{73.75}GaN_6O_8$
Formula weight	2580.88
Temperature/K	150(2)
Crystal system	monoclinic
Space group	C2/c
a/Å	25.1999(16)
b/Å	15.4651(16)
c/Å	24.792(2)
α/°	90
β/°	112.931(7)
γ/°	90
Volume/Å^3	8898.3(14)
Z	4
ρ_{calc}g/cm^3	1.927
μ/mm^{-1}	0.541
F(000)	5047.0
Crystal size/mm^3	0.200 × 0.130 × 0.060
Radiation	MoKα (λ = 0.71073)
2Θ range for data collection/°	3.164 to 50.282
Index ranges	-30 ≤ h ≤ 30, -17 ≤ k ≤ 18, -29 ≤ l ≤ 29
Reflections collected	99261
Independent reflections	7923 [R_{int} = 0.0422, R_{sigma} = 0.0266]

Data/restraints/parameters	7923/10335/1101
Goodness-of-fit on F^2	1.017
Final R indexes [I>=2σ (I)]	R_1 = 0.0752, wR_2 = 0.1864
Final R indexes [all data]	R_1 = 0.1021, wR_2 = 0.2063
Largest diff. peak/hole / e Å^{-3}	0.74/-0.69

The class of $[Al(OR^F)_4]^-$anions often tend to crystallize in a superstructure. Therefore, the obtained crystals of **3** had to be measured at a temperature of 150 K instead of 100 K. As the highly symmetric, perfluorinated aluminiumalkoxide anions are known to be heavily disordered at 100 K measurement temperature (very small rotational barriers of the -$C(CF_3)_3$ moieties and the CF_3-groups), they were even more disordered a 150 K. Additionally the displacement parameters became larger. As the scattering power inversely correlates to the exponent of the displacement parameter, the scattering power was much weaker at this temperature. A statement describing this very issue has been included as vrf in the cif file. Nevertheless, the structure was unambiguously proven. It should also be noted, that the synthesis of **3** was successfully reproduced multiple times by applying different crystallization techniques. An improvement of the scattering power of the single crystals could however not be observed. The chosen data set, was the most promising and in this context theta values of 25.1°, a residual density of 0.74/-0.69 eÅ^{-3}, R_1 = 7.5% and wR_2 = 20.6% are quite reasonable.

2.3. Crystal Structure Data for $[In_3(bipy)_6]^{3+}\{[Al(OR^F)_4]^-\}_3$ (4)

Supplementary Table 3: Crystal data and structure refinement for **4**.

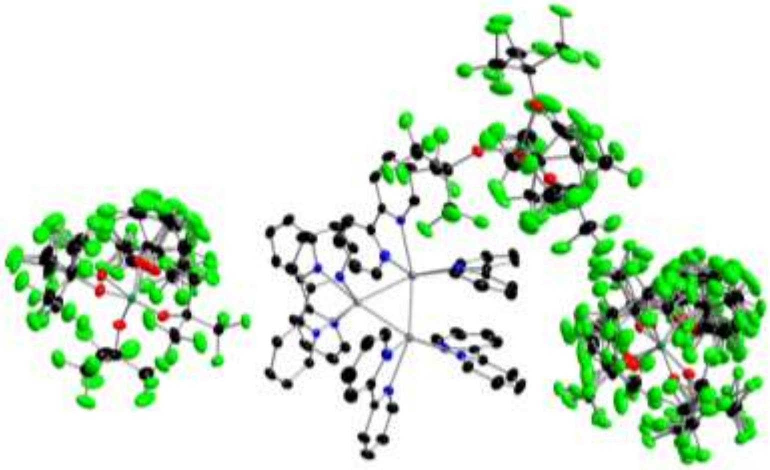

CCDC Deposition Number: 1032680

Identification code	**4**
Empirical formula	$C_{108}H_{48}Al_3F_{108}In_3N_{12}O_{12}$
Formula weight	4182.98
Temperature/K	100(2)
Crystal system	monoclinic
Space group	$P2_1/c$
a/Å	22.4009(11)
b/Å	30.7088(16)
c/Å	20.4177(10)
α/°	90
β/°	95.459(2)
γ/°	90
Volume/Å^3	13981.7(12)
Z	4
ρ_{calc}g/cm^3	1.987
μ/mm^{-1}	0.716
F(000)	8136.0
Crystal size/mm^3	0.370 × 0.210 × 0.210
Radiation	MoKα (λ = 0.71073)
2Θ range for data collection/°	1.826 to 55.56
Index ranges	-29 ≤ h ≤ 29, -39 ≤ k ≤ 39, -26 ≤ l ≤ 26
Reflections collected	206231
Independent reflections	32673 [R_{int} = 0.0877, R_{sigma} = 0.0429]
Data/restraints/parameters	32673/82692/3337
Goodness-of-fit on F^2	1.204
Final R indexes [I>=2σ (I)]	R_1 = 0.0680, wR_2 = 0.1492
Final R indexes [all data]	R_1 = 0.0755, wR_2 = 0.1530
Largest diff. peak/hole / e Å^{-3}	1.63/-1.35

2.4. Crystal Structure Data for $[In_3(bipy)_5]^{3+}\{[Al(OR^F)_4]^-\}_3$ (5)

Supplementary Table 4: Crystal data and structure refinement for **5**.

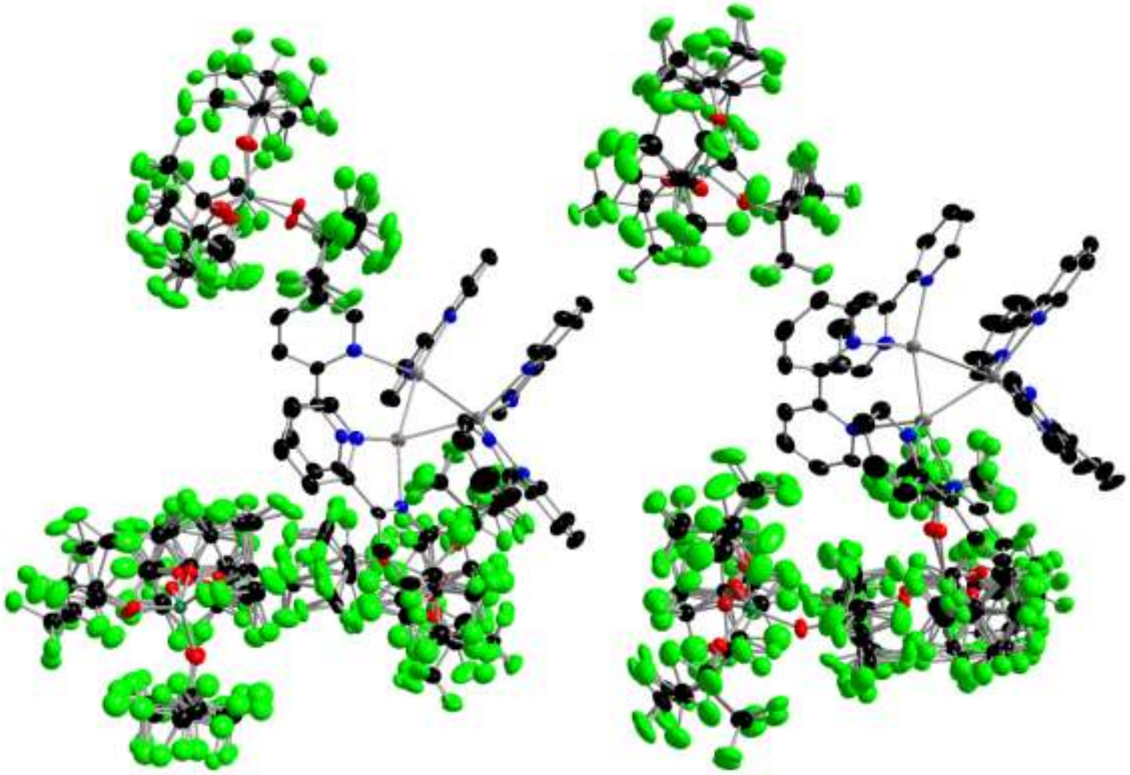

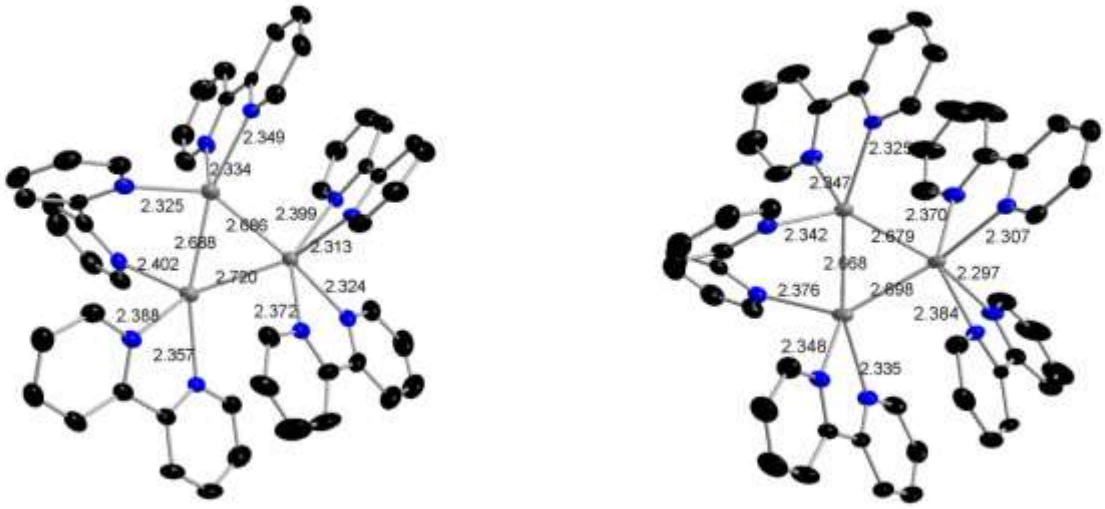

CCDC Deposition Number: 1033040

Identification code	**5**
Empirical formula	$C_{422}H_{184}Al_{12}F_{437}In_{12}N_{40}O_{48}$
Formula weight	16586.68
Temperature/K	100(2)
Crystal system	triclinic
Space group	P-1
a/Å	16.2891(6)
b/Å	25.6709(9)
c/Å	33.2531(12)

α/°	89.470(2)
β/°	87.358(2)
γ/°	81.522(2)
Volume/Å^3	13738.4(9)
Z	1
ρ_{calc}g/cm^3	2.005
μ/mm^{-1}	0.729
F(000)	8057.0
Crystal size/mm^3	0.130 × 0.110 × 0.060
Radiation	MoKα (λ = 0.71073)
2Θ range for data collection/°	2.016 to 55.22
Index ranges	-21 ≤ h ≤ 21, -33 ≤ k ≤ 33, -43 ≤ l ≤ 43
Reflections collected	351052
Independent reflections	62800 [R_{int} = 0.0492, R_{sigma} = 0.0664]
Data/restraints/parameters	62800/335118/5878
Goodness-of-fit on F^2	1.024
Final R indexes [I>=2σ (I)]	R_1 = 0.0655, wR_2 = 0.1519
Final R indexes [all data]	R_1 = 0.1043, wR_2 = 0.1709
Largest diff. peak/hole / e Å^{-3}	1.74/-1.43

2.5. Crystal Structure Data for $[In_4(bipy)_6]^{4+}\{[Al(OR^F)_4]^-\}_4$ (6)

Supplementary Table 5: Crystal data and structure refinement for **6**.

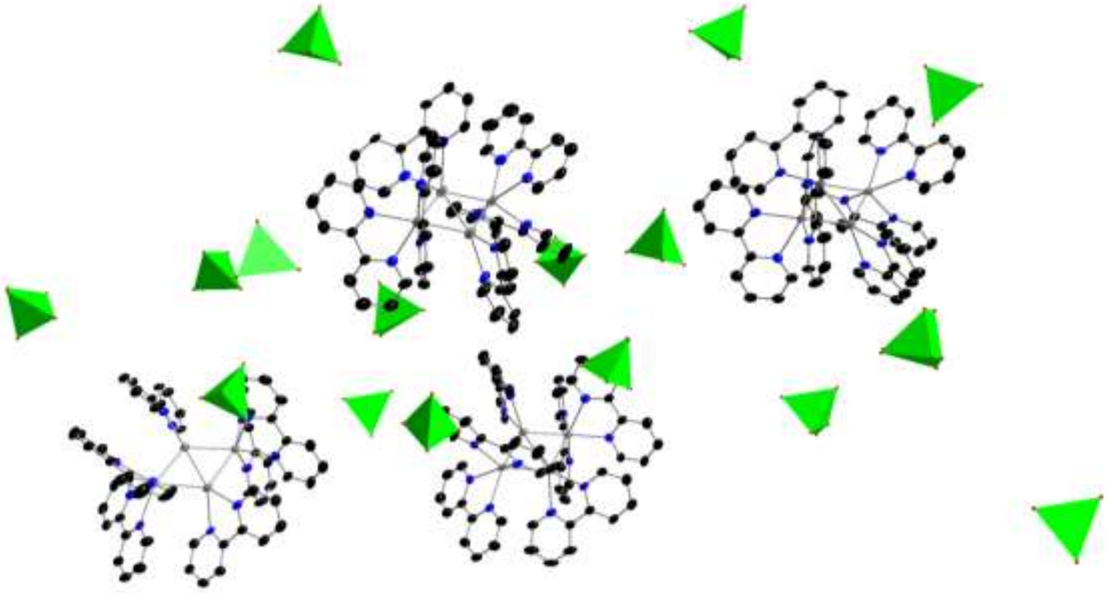

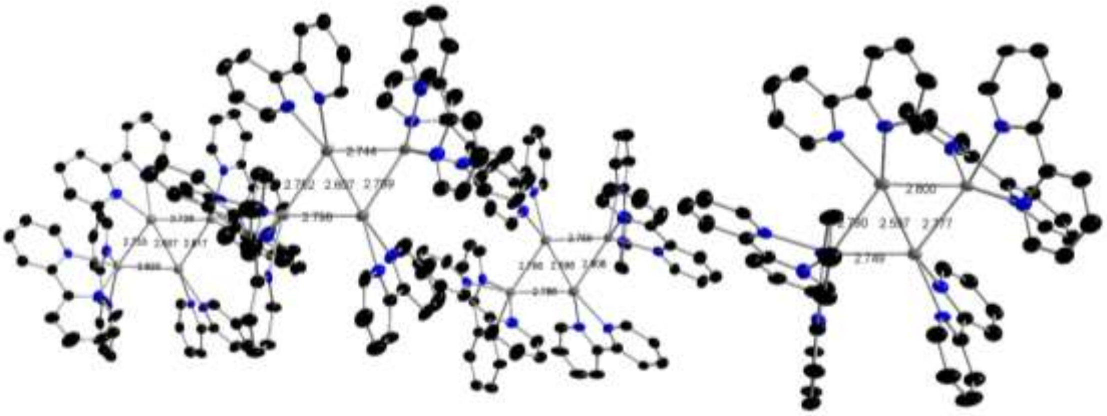

CCDC Deposition Number: 1032732

Identification code	**6**
Empirical formula	$C_{124}H_{48}Al_4F_{144}In_4N_{12}O_{16}$
Formula weight	5264.94
Temperature/K	100(2)
Crystal system	triclinic
Space group	*P*1;¯
a/Å	34.744(5)
b/Å	34.792(5)
c/Å	37.210(5)
α/°	106.127(4)
β/°	101.609(4)
γ/°	118.938(3)
Volume/Å^3	34671(8)
Z	8
ρ_{calc}g/cm^3	2.017

μ/mm^{-1}	0.763
F(000)	20384.0
Crystal size/mm^3	0.200 × 0.150 × 0.100
Radiation	MoKα (λ = 0.71073)
2Θ range for data collection/°	1.888 to 49.008
Index ranges	-39 ≤ h ≤ 40, -40 ≤ k ≤ 39, -43 ≤ l ≤ 43
Reflections collected	566584
Independent reflections	111628 [R_{int} = 0.0961, R_{sigma} = 0.1155]
Data/restraints/parameters	111628/927436/11257
Goodness-of-fit on F^2	1.078
Final R indexes [I>=2σ (I)]	R_1 = 0.0740, wR_2 = 0.1691
Final R indexes [all data]	R_1 = 0.1550, wR_2 = 0.2157
Largest diff. peak/hole / e Å^{-3}	2.64/-1.81

Due to clarity, the $[Al(OR^F)_4]^-$ anions are only depicted as AlO_4 polyhedra.

2.6. Crystal Structure Data for $[In_4(phen)_6]^{4+}\{[Al(OR^F)_4]^-\}_4$ (7)

Supplementary Table 6: Crystal data and structure refinement for **7**.

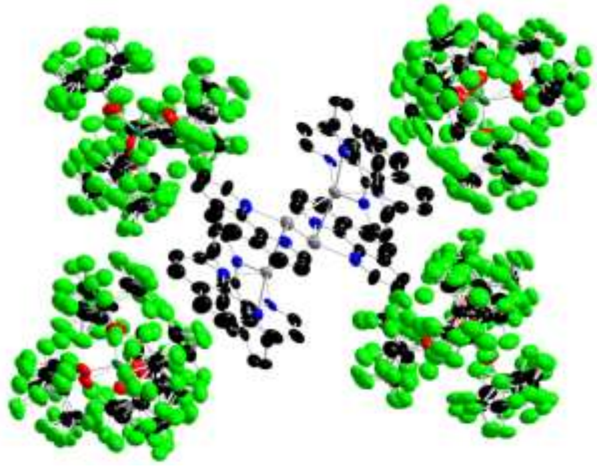

CCDC Deposition Number: 1034231

Identification code	**7**
Empirical formula	$C_{86}H_{39}Al_2F_{75}In_2N_6O_8$
Formula weight	2992.83
Temperature/K	100(2)
Crystal system	monoclinic
Space group	$P2_1/c$
a/Å	20.2872(5)
b/Å	28.7711(7)
c/Å	19.7149(6)
α/°	90
β/°	117.3030(13)
γ/°	90
Volume/Å^3	10225.3(5)
Z	4
ρ_{calc}g/cm^3	1.944
μ/mm^{-1}	0.664
F(000)	5840.0
Crystal size/mm^3	0.4 × 0.15 × 0.07
Radiation	MoKα (λ = 0.71073)
2Θ range for data collection/°	2.26 to 45.29
Index ranges	-21 ≤ h ≤ 21, -31 ≤ k ≤ 30, -21 ≤ l ≤ 21
Reflections collected	109764
Independent reflections	13548 [R_{int} = 0.0891, R_{sigma} = 0.0635]
Data/restraints/parameters	13548/63817/2285
Goodness-of-fit on F^2	1.272
Final R indexes [I>=2σ (I)]	R_1 = 0.0900, wR_2 = 0.2232
Final R indexes [all data]	R_1 = 0.1347, wR_2 = 0.2524
Largest diff. peak/hole / e Å^{-3}	1.44/-0.66

2.7. Crystal Structure Data for $[(phen)_2In{-}Ag(FB)]^{2+}\{[Al(OR^F)_4]^-\}_2$ (8)

Supplementary Table 7: Crystal data and structure refinement for **8**.

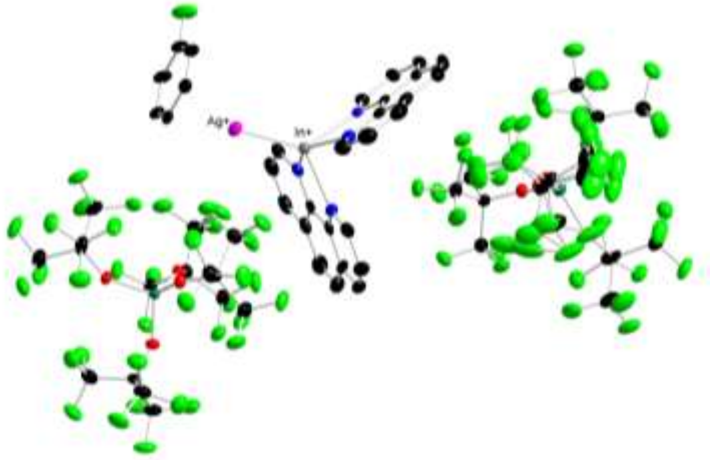

CCDC Deposition Number: 1034089

Identification code	**8**
Empirical formula	$C_{80}H_{36}AgAl_2F_{76}InN_4O_8$
Formula weight	2901.78
Temperature/K	100(2)
Crystal system	monoclinic
Space group	$P2_1/n$
a/Å	16.4785(8)
b/Å	23.7211(12)
c/Å	25.2293(13)
α/°	90
β/°	97.519(3)
γ/°	90
Volume/Å^3	9777.0(9)
Z	4
ρ_{calc}g/cm^3	1.971
μ/mm^{-1}	0.656
F(000)	5656.0
Crystal size/mm^3	0.2 × 0.1 × 0.1
Radiation	MoKα (λ = 0.71073)
2Θ range for data collection/°	2.366 to 46.116
Index ranges	-18 ≤ h ≤ 17, -25 ≤ k ≤ 26, -27 ≤ l ≤ 27
Reflections collected	75310
Independent reflections	13657 [R_{int} = 0.0498, R_{sigma} = 0.0401]
Data/restraints/parameters	13657/13320/1696
Goodness-of-fit on F^2	1.023
Final R indexes [I>=2σ (I)]	R_1 = 0.0363, wR_2 = 0.0796
Final R indexes [all data]	R_1 = 0.0580, wR_2 = 0.0898
Largest diff. peak/hole / e Å^{-3}	0.67/-0.52

2.8. Comparison of the structural parameters of 6 and the protein Viscotoxin B2

Supplementary Table 8: Comparison of the structural parameters of **6** and the protein Viscotoxin B2.[10]

	6	Viscotoxin B2
space group	*P*1;¯	$P2_12_12_1$
unit cell	a=34.74 Å	a=39.82 Å
	b=34.79 Å	b=40.39 Å
	c=37.21 Å	c=44.69 Å
refl. collected	566587	680367
uniq refl.	111631	34307
R-factor (all)	15.5%,	12.7%
resolution	0.857 Å	1.05 Å

Concerning structures **6**, **7** and **8**, the low theta values can be explained with the large unit cell sizes in combination with the heavy disorder of the highly symmetric, perfluorinated aluminumalkoxide anions. Structure **6** for example resembles the size of the protein Viscotoxin B2.[10] By contrast to Viscotoxin B2, we could not make so much use of symmetry and thus the number of unique reflections in our triclinic structure **6** is more than three times as high (111631 vs. 34307). Thus, the number of equivalent reflections that could be merged for accuracy is much smaller. Despite this, our resolution is unambiguously better (0.857 vs. 1.05 Å). In this context, the quality of our crystal structure data is clearly well within the limits of accepted standards.

2.9. Orienting DFT-Calculations of Reactions of $[M(PhF)_2]^+$ complexes and 1-3 bipy Molecules

Supplementary Table 9: Possibly occurring ligand exchange reactions of the ?[6]-coordinated $[M(PhF)_2]^+$ complexes (M = Ga, In, PhF = C_6H_5F) and bipy. All compounds correspond to a singlet state occupation and the $[M(bipy)]^+$ complexes are coordinated in a N-chelated, the $[M(bipy)_2]^+$ complexes in a tetragonal pyramidal and the $[M(bipy)_3]^+$ complexes in a distorted octahedral fashion (gas phase, 298.15 K, 1.0 bar, BHLYP/SV(P) and B3LYP/SV(P) level). The hybrid BHLYP and B3LYP functionals and the valence SV(P) basis set were chosen, as they describe the electronic occupations of the closed and open shell systems in a correct manner (see below).

Reaction	$\Delta_rH°$ [kJ mol^{-1}]		$\Delta_rG°$ [kJ mol^{-1}]	
	(M = Ga)	(M = In)	(M = Ga)	(M = In)
$[M(PhF)_2]^+$ + bipy → $[M(bipy)]^+$ + 2 PhF	−160 / −156	−146 / −147	−185 / −182	−173 / −174
$[M(PhF)_2]^+$ + 2 bipy → $[M(bipy)_2]^+$ + 2 PhF	−273 / −269	−266 / −267	−248 / −246	−245 / −244
$[M(PhF)_2]^+$ + 3 bipy → $[M(bipy)_3]^+$ + 2 PhF	−334 / −360	−299 / −364	−238 / −264	−210 / −273

2.10. Calculations of Lattice Enthalpies ($\Delta_{latt}H°$)

Supplementary Table 10: The $\Delta_{latt}H°$ values were calculated by applying the Jenkins generalized Kapustinskii equation.[4] The ionic volumes (V_{ion}) were extracted from the given references: $V_{ion}(Cl^-)$ = 0.047 nm[34], $V_{ion}([AsF_6]^-)$ = 0.110 nm[34], $V_{ion}([Al(OR^F)_4]^-)$ = 0.758 nm[34] (R^F = $C(CF_3)_3$), $V_{ion}(In^+)$ = 0.0122 nm[311], $V_{ion}(Al^+)$ ≈ $V_{ion}(Na^+)$ = 0.0039 nm[34], $V_{ion}(In^{3+})$ = 0.0035 nm[312], $V_{ion}(Al^{3+})$ = 0.0013 nm[312], $V_{ion}([In_3]^{3+})$ ≈ 3 x $V_{ion}(In^+)$ and $V_{ion}([Al_3]^{3+})$ ≈ 3 x $V_{ion}(Al^+)$.

	$\Delta_{latt}H°$ [kJ mol^{-1}]	$\Delta_{latt}H°$ [kJ mol^{-1}]
	M = Al	M = In
**MCl**	808	772
**$M[AsF_6]$**	632	619
**$M[Al(OR^F)_4]$**	364	363
**$M(Cl)_3$**	4912	484
**$M[AsF_6]_3$**	3801	3779
**$M[Al(OR^F)_4]_3$**	2166	2164
**$[M_3]Cl_3$**	4621	4148
**$[M_3][AsF_6]_3$**	3699	3498
**$[M_3][Al(OR^F)_4]_3$**	2157	2138

Supplementary Table 11: Difference of $\Delta_{latt}H°$ of the $M^{3+}(A^-)_3$ and $(M)_3(A^-)_3$ salts.

	$\Delta_{latt}H°$ (MA_3–$[M_3]A_3$) [kJ mol^{-1}]	
**A^-**	**M = Al**	**M = In**
**Cl^-**	291	697
**$[AsF_6]^-$**	103	281
**$[Al(OR^F)_4]^-$**	8	26

2.11. Structural Relaxations of the $[M(bipy)_{1\text{-}2}]^+$ Complexes

Supplementary Table 12: Structural relaxations upon changing the occupation of the $[M(bipy)]^+$ and $[M(bipy)_2]^+$ complexes from singlet to triplet as well as the spin densities of the triplet states (M = Ga, In; gas phase, 298.15 K, 1.0 bar, spin density cut off at 0.01 a.u., BHLYP/SV(P) and B3LYP/SV(P) level).

		M = Ga				M = In			
		singlet		triplet	spin density	singlet		triplet	spin density
**$[M(bipy)]^{+[a]}$**	**BHLYP/ SV(P)**	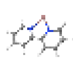	→	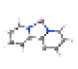	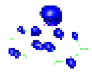	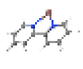	→	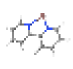	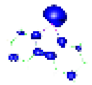
	**B3LYP/ SV(P)**	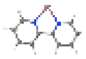	→	–[c]	–[c]	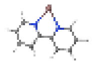	→	–[c]	–[c]
**$[M(bipy)_2]^{+[b]}$**	**BHLYP/ SV(P)**	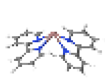	→	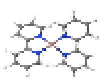	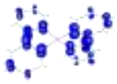	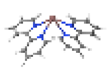	→	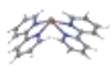	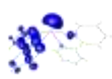
	**B3LYP/ SV(P)**	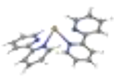	→	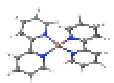	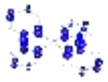	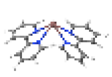	→	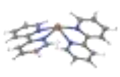	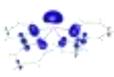

[a] While the $[M(bipy)]^+$ complexes retain their original coordination mode in the triplet state, the average M–N distances shorten from 223 pm to 191 pm for gallium and from 242 pm to 210 pm for indium. 57% of the spin density is located at the gallium atom and 60% at the indium atom. [b] The $[Ga(bipy)_2]^+$ complex changes its coordination mode from tetragonal pyramidal in the singlet to tetrahedral in the triplet state and the average Ga–N bond length shortens from 243/244 pm to 191/193 pm. Only 5.4/6.0% of the spin density is located at the gallium atom, corresponding to a primarily ligand-centered triplet state. The $[In(bipy)_2]^+$ complex retains its original tetragonal pyramidal coordination mode in the triplet state, though the average In–N bond length shortens from 257 pm to 226/223 pm. 38/35% of the spin density is located at the indium atom. [c] Though the geometry optimization converged, the electronic occupation of the triplet state is not correctly described.

2.12. Single Point DFT calculated Coulomb Explosions

Supplementary Table 13: Single Point DFT calculated Coulomb explosions of a non-ligand-supported $[In_3]^{3+}$ cluster (In–In bond = 270 pm) and the frozen conformation of $\mathbf{4}^{3+}$ as observed in the solid state XRD-structure; the three resultant $[In(bipy)_2]^+$ monocations are cut out of the $\mathbf{4}^{3+}$ solid state structure (values at the BHLYP/SV(P) and B3LYP/SV(P) level).

**gas phase reaction**	**$\Delta_rH°$(gas) [kJ mol^{-1}]**
$[In_3]^{3+}$ → 3 In^+	−1459 / −1447
$[In_3(bipy)_6]^{3+}$ → 3 $[In(bipy)_2]^+$	−705 / −684

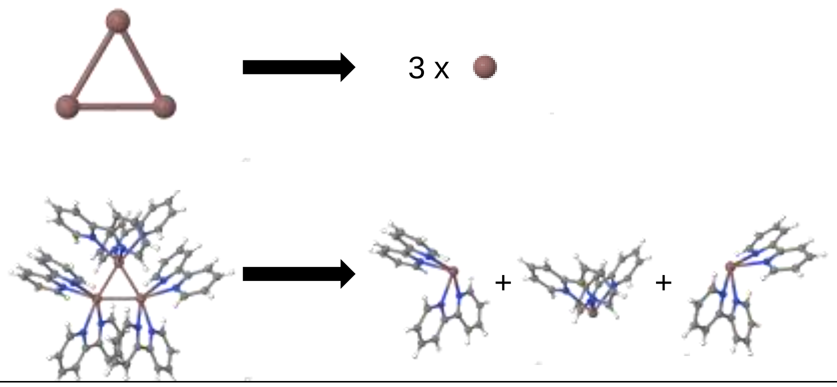

2.13. Summary of Calculated Energies of All Compounds

Supplementary Table 14: Summary of the energies that were used to calculate the thermodynamics of the ligand exchange reactions, the singlet-triplet and 4s/5s-4p/5p gaps and the spin densities. All values were calculated at the BHLYP/SV(P) and B3LYP/SV(P) level and are given in hartree.

	E_{SCF}		E_{Vib}	
	**BHLYP/SV(P)**	**B3LYP/SV(P)**	**BHLYP/SV(P)**	**B3LYP/SV(P)**
C_6H_5F	–331.0482226	–331.0482034	0.0959598	0.0925214
bipy	–494.6946108	–494.6986743	0.1638802	0.1577100
$[Ga(C_6H_5F)_2]^+$	–2586.4767170	–2586.4304398	0.1935492	0.1867277
$[Ga(C_6H_5F)_2]^+$ (triplet)	–2586.3584475	-[a]	0.1909426	-[a]
$[In(C_6H_5F)_2]^+$	–663.7806972	-663.7923391	0.1934490	0.1865888
$[In(C_6H_5F)_2]^+$ (triplet)	-[a]	-[b]	-[a]	-[b]
$[Ga(bipy)]^+$	–2419.1348973	–2419.0913809	0.1663402	0.1602839
$[Ga(bipy)]^+$ (triplet)	–2419.0603050	-[b]	0.1657081	-[b]
$[In(bipy)]^+$	–496.4333115	–496.4497574	0.1660663	0.1600393
$[In(bipy)]^+$ (triplet)	–496.3469001	-[b]	0.1649878	-[b]
$[Ga(bipy)_2]^+$	–2913.8749759	–2913.8356733	0.3319074	0.3196552
$[Ga(bipy)_2]^+$ (triplet)	–2913.8730703	–2913.8276538	0.3308638	0.3188726
$[In(bipy)_2]^+$	–991.1765158	–991.1965063	0.3316817	0.3194634
$[In(bipy)_2]^+$ (triplet)	–991.1343055	–991.1598009	0.3307375	0.3178135
$[Ga(bipy)_3]^+$	–3408.5920055	–3408.5691839	0.4956653	0.4783648
$[Ga(bipy)_3]^{2+}$	–3408.3707502	–3408.3230555	0.5005471	0.4803650
$[In(phen)_2]^+$	–1143.4502823	–1143.4682946	0.3589391	0.3457572
$[Ag(C_6H_5F)]^+$	–477.6674975	–477.7393909	0.0967027	0.0933027
$[(phen)_2In–Ag(C_6H_5F)]^{2+}$	–1621.1485501	–1621.2429546	0.4580223	0.4415444
$[(bipy)In–In(bipy)]^{2+}$ (triplet)	–992.7513250	–992.7878410	0.3334579	0.3209590
$[(bipy)_2In–In(bipy)_2]^{2+}$ (triplet)	–1982.2828853	–1982.3410737	0.6638210	0.6368196
$[(bipy)_2In–In(bipy)]^{2+}$ (triplet)	–1487.5298771	–1487.5691675	0.4981230	0.4786379
$(In_3)^{3+}$ (In–In = 270 pm)	–4.3440794	–4.3778325	-[c]	-[c]
In^+	–1.6332369	–1.6429803	-[c]	-[c]
$[In_3(bipy)_6]^{3+}$ (from XRD: $\mathbf{4^{3+}}$)	–2972.4334328	–2972.4199255	-[c]	-[c]
$[In(bipy)_2]^+$ (1st cut out of $\mathbf{4^{3+}}$)	–990.8949831	–990.8875109	-[c]	-[c]
$[In(bipy)_2]^+$ (2nd cut out of $\mathbf{4^{3+}}$)	–990.9051044	–990.8986476	-[c]	-[c]
$[In(bipy)_2]^+$ (3rd cut out of $\mathbf{4^{3+}}$)	–990.9017348	–990.8943664	-[c]	-[c]

[a] Even after 500 iteration cycles, the geometry optimization of the triplet state did not converge. [b] Though the geometry optimization converged, the electronic occupation of the triplet state is not correctly described. [c] Single point DFT calculations do not included a geometry optimization and therefore no vibrational analysis.

2.14. Representatitve Compilation of Compounds containing In–In bonds

Supplementary Table 15: Compilation of organometallic and inorganic indium compounds, featuring at least one In–In bond (*d*(In–In) in pm). The entries are ordered in terms of increasing *d*(In–In) values.

**compound**	***d*(In–In) [pm]**	**reference**
$[In_4(phen)_6]^{4+}\{[Al(OR^F)_4]^-\}_4$ (**7**)	258.1–286.1[a]	this work
$[In_4(bipy)_6]^{4+}\{[Al(OR^F)_4]^-\}_4$ (**6**)	259.6–280.8[a]	this work
$In_5Mo_{18}O_{28}$ (In_5-moieties)	261.6–266.5	13
$In_{11}Mo_{40}O_{62}$ (In_5- and In_6-moieties)	262.4–268.9	14
$In_3(PO_4)_2$ (In_2-dimers)	263.0	15,16
$[In\{C(Si(Me_3)_3\}\{\{OC(C_6H_5)\}_2CH\}]_2$	264.6–279.3	17
$[In\{(O_2CPh)C(SiMe_3)_3\}]_2$	265.4	18
$[In\{N(C_6H_2\text{-}2,4,6\text{-}Me_3)CH\}_2O(SO_2)(CF_3)]_\infty$	265.6, 266.5	19
$[In_3(bipy)_6]^{3+}\{[Al(OR^F)_4]^-\}_3$ (**4**)	266.1–278.1	this work

[In$_3$(bipy)$_5$]$^{3+}${[Al(OR^F)$_4$]$^-$}$_3$ (**5**)	266.8–269.8	this work
KInBr$_3$ ({K$^+$}$_2$[In$_2$Br$_6$]$^{2-}$)	267.(1)	20
[In$_2$X$_4$([18]crown-6)] (X = Cl, Br, I)	267.3–272.5	21-23
In$_2$Br$_3$ ({In$^+$}$_2$[In$_2$Br$_6$]$^{2-}$)	268.8, 271.6	24
[In{In(C$_6$H$_2$-2,4,6-*i*Pr$_3$)$_2$}$_3$]	269.6	25,26
In$_5$Br$_7$ ({In$^+$}$_3$[In$_2$Br$_6$]$^{2-}$Br$^-$)	270.7	27-29
[InCl{N(C$_6$H$_3$-2,6-*i*Pr$_2$)}$_2${CN(C$_5$H$_8$-2,6-Me$_2$)$_2$}]$_2$	270.9	30
[InCl$_2$(OC$_4$H$_8$)$_2$]$_2$	271.5	31
[InCl{C$_6$H$_3$-2,6-(CH$_2$NMe$_2$)$_2$}]$_2$	271.6	32
{[CH(NMe$_2$)$_2$]$^+$}$_2$[In$_2$Cl$_6$]$^{2-}$	271.9	33
[InCl{N(C$_6$H$_3$-2,6-*i*Pr$_2$)}$_2${CN(*i*Pr)$_2$}]$_2$	272.0	30
In$_5$E$_5$X (E = S, Se; X = Cl, Br)	272.3–274.8	34
[In(OC$_4$H$_8$){C$_{10}$H$_6$(NSiMe$_3$)$_2$}]$_2$	272.4	35,36
{[Ph$_4$P]$^+$}$_2$[In$_2$Cl$_6$]$^{2-}$	272.7	37
[InBr{N(SiMe$_3$)C(Ph)C(SiMe$_3$)$_2$}]$_2$	272.8	38
[InCl{N(C$_6$H$_3$-2,6-*i*Pr$_3$)CH}$_2$]$_2$	272.8	39
MIn$_7$E$_9$ (M = Rb, Cs; E = S, Se)	273.1–273.6	40
[In$_3$(In$_2$)$_3$(PhP)$_4$(Ph$_2$P$_2$)$_3$Cl$_7$(PEt$_3$)$_3$]	273.5–275.1	41,42
In$_6$S$_7$ (two cluster systems with one In−In contact)	274.1	43
Na$_{24}$In$_5$O$_{15}$ (indium centered [In(In$_4$)]$^{6+}$ tetrahedra)	274.0	44
[In{C$_6$H$_2$-2,4,6-(CF$_3$)$_3$}$_2$]$_2$	274.4	45
[InBr$_2${C{N(C$_6$H$_2$-2,4,6-Me$_3$)CH}$_2$}]$_2$	274.4	39
[InI$_2${P(*n*Pr)$_3$}]$_2$	274.5	46
[In$_2$I$_3${HB(N$_2$C$_3$(*t*Bu)H$_2$)$_3$}(N$_2$C$_3$(*t*Bu)H$_2$)]	274.8	47
[H(NC$_7$H$_{13}$)$_2$]$^+$[In$_5$Br$_8$(NC$_7$H$_{13}$)$_4$]$^-$	274.5–274.8	48
[In$_6$I$_8${(NMe$_2$CH$_2$)$_2$}$_4$]	275.6–283.5	49,50
[InCl{N(C$_6$H$_3$-2,6-*i*Pr$_2$)C(H)}$_2$CPh]$_2$	275.8	51
[In{In(3,6-*t*Bu-carbazol-9-yl)$_3$}]$_2$	275.9	52
In$_6$Se$_7$ (two cluster systems with one In−In contact)	276.0	53
[In(PhP(CH$_2$SiMe$_2$NSiMe$_2$CH$_2$)$_2$PPh)]$_2$	276.2	54
In$_5$S$_4$ (In$_5$-moieties)	276.2–276.9	55
[In(SiMe)$_2${N(*t*Bu)}$_4$]$_2$	276.8	56
In$_4$Se$_3$ (In$_3$-moieties)	275.6–277.6	57
[In$_8$(Si*t*Bu$_3$)$_6$]	277.0–330.3	58,59
[In(P)$_2${N(*t*Bu)}$_4$]$_2$	277.2	60
[In$_2$Br$_3$I{(NMe$_2$CH$_2$)$_2$}$_4$]	277.5	61
[In(C$_6$H$_2$-2,4,6-*i*Pr$_3$)$_2$]$_2$	277.5	25,26
[In$_2${B{N(C$_6$H$_3$-2,6-(*i*Pr)$_2$CH}$_2$}$_3$]	277.7	62
[In$_2$ClI{C$_6$H$_3$-2,6-(C$_6$H$_2$-2,4,6-Me$_3$)$_2$}$_2$]$_2$	277.8	63
[In{[OC(CH$_3$)CH(CH$_3$)CO]C(SiMe$_3$)$_3$}]$_2$	278.0	64
[InCl{C$_6$H$_3$-2,6-(C$_6$H$_2$-2,4,6-Me$_3$)$_2$}]$_4$	279.0	63
[InI{C$_6$H$_3$-2,6-(C$_6$H$_2$-2,4,6-Me$_3$)$_2$}]$_4$	279.5	63
[In$_2$(SiMePh$_2$)$_3$(OH)$_3$Li$_2$(OC$_4$H$_8$)$_3$]	279.6	65
[In{η^3-[Me$_3$SiNCH$_2$]$_3$(μ-In)CMe}]$_2$	280.7	66,67
In$_3$I$_2$[C(SiMe$_3$)$_3$]$_3$	281.0–298.8	68
[In$_{12}$(Si*t*Bu$_3$)$_8$]	281.4–330.7	69,70
[RIIn{μ-InR}$_4$InIR] (R = {N(C$_6$H$_3$-2,6-Me$_2$)C(Me)}$_2$CH)	281.2–285.4	71
[In$_3$Br$_3${C(SiMe$_3$)$_3$}$_3$][Li(OC$_4$H$_8$)$_3$]	281.6–282.7	72
InSe (In$_2$-dimers)	281.8	73
[InX{C(SiMe$_3$)$_3$}]$_4$ (X = Cl, Br)	282.3 (Cl), 283.5 (Br)	74
[Na(OC$_4$H$_8$)$_6$]$^+$[In$_3$(SiPh$_3$)$_6$]$^-$ (linear triindane)	282.7	65
[In{CH(SiMe$_3$)$_2$}$_2$]$_2$	282.8	75
[InCl{N(C$_6$H$_3$-2,6-*i*Pr$_2$)C(Me)}$_2$CH]$_2$	283.4	76

$BaIn_{0.60}Ga_{0.40}$ (Ga/In parallelograms)	283.5–284.9	[77]
$Na_{15}In_{27.4}$ (four different cluster systems)	284.1–415.2	[78]
$Na_7In_{11.8}$ (three different cluster systems)	284.5–347.3	[79]
$[In\{CH(SiMe_3)_2\}_2CNR]_2$ (R = *t*Bu, Ph)	284.7 (*t*Bu), 284.8 (Ph)	[80]
$Cs_{22}In_6(SiO_4)_4$ ($[In_6]^{6-}$ octahedra)	284.8–377.7	[81]
$[\{(SiMe_3)_2HC\}_2In{-}In(C_2C_6H_5)\{CH(SiMe_3)_2\}_2]^-$	285.2	[82]
$In_2O(PO_4)$ ($[In_2]^{4+}[In_2O_2(PO_4)_2]^{4-}$)	286.2	[16]
$K_{39}In_{80}$ (five different cluster systems)	286.8–335.9	[83]
$[In_8(C_6H_2\text{-}2,4,6\text{-}Me_3)_4]$	287.5–299.6	[84,85]
Rb_2In_3 ($[In_6]^{4-}$ octahedra)	287.5–309.4	[86]
$[In\{Si(SiMe_3)_3\}]_2$	286.8	[87]
$[InSi(SiMe_3)_3]_4$	288.7–290.1	[88]
$Na_{96}In_{97}Z_2$ (In_{74} fullerene; Z = Ni, Pd, Pt)	292.0–305.0	[89,90]
$[In\{Si(tBu)_3\}_2]_2$	292.2	[91,92]
$[In\{Si(tBu)_2Ph\}_2]_2$	293.8	[93]
$Ba_5In_4Bi_5$ (tetragonal In_4Bi pyramids)	295.8	[94]
K_8In_{11} ($[In_{11}]^{7-}$ cluster and one delocalized e^-)	296.3–328.4	[95]
$[In\{C_6H_3\text{-}2,6\text{-}(C_6H_3\text{-}2,6\text{-}iPr_2)_2\}]_2$	297.9	[96]
$[In\{C(SiMe_3)_3\}]_4$	298.0–318.0	[97,98]
La_3In_5 (square $[In_5]^{9-}$ pyramids)	301.0–357.5	[99]
$[\{(Me_3Si)_3C\}In(\mu\text{-}Cl)_2\{\mu\text{-}Fe(CO)_4\}In\{C(SiMe_3)_3\}]$	304.8	[100]
$NaIn_2$ ($[In_4]^{2-}$ tetrahedra)	306.6–315.2	[78]
$[In\{N(C_6H_2\text{-}2,4,6\text{-}Me_3)C(Me)\}_2CH]_2$	319.7	[101,102]
In–In (metal)	325.2, 337.7	[103]
$C_{44}H_{68}In_4N$ (In_4-tetrahedra)	326.8	[104]
$[In_4\{Cp_2Mo_2(CO)_4P_2\}_8]^{4+}\{[Al(OR^F)_4]^-\}_4$	348.2–396.0	[105]
InCl (In_4-tetrahedra)	359.1–476.3	[11,106]
$Pt_2In_{14}Ga_3O_8F_{15}$($[PtIn_6]^{10+}$ octahedrons)	359.7–364.4	[107]
In_7Cl_9 ($\{In^+\}_6InCl_9$)	361.0–387.0	[108,109]
$[In\{\eta^5\text{-}C_5(CH_2Ph)_5\}]_2$	363.1	[110]
$[InBr\{(NMe_2CH_2)_2\}]_2$	367.8	[111]
$[In(\eta^5\text{-}C_5Me_5)]_6$	394.2–396.3	[112,113]
$[In(\eta^5\text{-}C_5H_5)]_\infty$ / $[In(\eta^5\text{-}C_5H_4Me)]_\infty$	398.6	[114]

3. Supplementary Methods

3.1. Genereal Procedures

All manipulations were performed using Schlenk or glove box techniques in an argon atmosphere (H_2O and O_2 < 1 ppm). *o*-$C_6H_4F_2$ and C_6H_5F were dried over CaH_2, distilled and had H_2O contents below 5 ppm (Karl-Fischer titrations). Because the obtained compounds contain large amounts of fluorine in chemically very stable CF_3 groups, standard combustion analyses have proven to be incomplete. Characterizations of novel compounds were therefore done on the basis of single-crystal X-ray analysis and multinuclear NMR spectroscopy. It was several times attempted, to obtain ESI-MS-data of these systems. However, these ions are very sensitive and in no cases, we could obtain meaningful spectra – presumably due to oxidation and/or hydrolysis on the way to the ionization chamber (as very frequently encountered with our sensitive systems). Since the other investigations strongly suggested these multiply charged cations to only exist in the solid state, we did not pursue this method further. The single-crystal structures of all compounds within this manuscript have been reproduced from independent syntheses, apart from **5** and **6**. Overall, longer crystallization times seem to yield single crystals of slightly better quality. Obtained single crystals were coated with perfluoroether oil and mounted on 0.1 mm micromounts at the respective crystallization temperature. The crystal structure data were collected from the shock-cooled crystals at 100 K (150 K for structure **3** due to a superstructure), on a Bruker SMART APEX2 CCD Quazar diffractometer equipped with a CCD area detector using Mo-K_α radiation. Data reduction was done with SAINT[115] and scaling and absorption correction of the data was performed by SADABS-2012/1[116] and 2014/3[117]. The structures were solved by intrinsic phasing using SHELXT[118] and were refined by full matrix least-squares minimization on F^2 using all reflections with SHELXL[119] in the ShelXle[120] GUI. For **6** the twin law 0 1 0 1 0 0 −1 −1 −1 was additionally applied, with twin fractions of 43% and 57%. In addition, a riding model was used to attribute idealized positions to all hydrogen atoms. The disorder of the $[Al(OR^F)_4]^-$ anions was treated using DSR.[121] The graphical representations were prepared using the software Diamond 3.2i.[122] CCDC 1032681 (**3**), CCDC 1032680 (**4**), CCDC 1033040 (**5**), CCDC 1032732 (**6**), CCDC 1034231 (**7**) and CCDC 1034089 (**8**) contain the supplementary crystallographic data for this chapter. Solution NMR spectra of **3**, **4**, **5**, **6**, **7** and **8** in *o*-$C_6H_4F_2$ were recorded on a BRUKER AVANCE III HD 300 MHz and a BRUKER AVANCE II 400 MHz WB spectrometer, at room temperature, using flame-sealed NMR tubes. For measuring and processing of the data the software Bruker Topspin 2.1 and 3.2 were used. Resonances are given in ppm and were referenced to $SiMe_4$ for the ^1H NMR spectra, to $CFCl_3$ for the ^{19}F NMR spectra, to a 1.1 M solution of $Al(NO_3)_3$ in D_2O for the ^{27}Al NMR spectra and to a 1.1 M solution $Ga(NO_3)_3$ in D_2O for the ^{71}Ga NMR spectra.[123] UV-VIS spectra were recorded on a Thermo Scientific Evolution 600 spectrometer. X-Band continuous wave EPR measurements were performed with a BRUKER EMX spectrometer and a BRUKER 4122-SHQ-E resonator (both from Bruker BioSpin GmbH, Rheinstetten, Germany). For the low-temperature measurements, an Oxford Instruments ESR900 cryostat was used. The Q-Band EPR spectra were recorded using a Bruker ELEXSYS E580 spectrometer in combination with an ER-5106QT-W1 resonator, which was immersed into an Oxford Instruments CF935O helium gas-flow cryostat. All magnetic-field calibrations were done by using a LiF:Li standard (g_{LiFLi} = 2.002293).[124] EPR simulations for an S = 1/2 spin system coupled to a single nucleus (I = 3/2) with the natural abundances of ^{69}Ga and ^{71}Ga were performed using the Easyspin toolbox function “pepper”.[125] The DFT calculations were performed using the program package TURBOMOLE 6.6[126-135] and at the BHLYP/SV(P) level of theory.[136-140]

3.2. Preparation of $[Ga(bipy)_3]^{2+}\{[Al(OR^F)_4]^-\}_2$ (3)

A moss-green solution of **1** (200 mg, 163 µmol, 1.00 eq) and bipy (50.8 mg, 326 µmol, 2.00 eq) in *o*-$C_6H_4F_2$ (1.0 mL) was slowly concentrated under reduced pressure and stored at +25 °C. Doing so, the formation of a black precipitate was observed, while the solution remained green. After five months green, platelet-shaped single crystals were isolated from the solution and characterized using single-crystal X-ray analysis and multinuclear NMR spectroscopy. The synthesis of **3**, was reproduced by storing a highly concentrated solution of **1** (100 mg, 81.4 µmol, 1.00 eq) and bipy (25.0 mg, 160 µmol, 1.97 eq) in C_6H_5F (200 µL) at −20 °C. Choosing this way, crystal growth was observed after one month. Yet, the obtained single crystals were of poorer quality (e.g. weaker scattering power). ^{1}H NMR (400 MHz, *o*-$C_6H_4F_2$, calibrated to *o*-$C_6H_4F_2$ = 7.12 ppm[1], 298 K): δ = 7.77 (m, CH, bipy), 8.19 (m, CH, bipy), 8.37 (m, CH, bipy), 8.50 (m, CH, bipy), 8.66 (m, CH, bipy), 8.82 (m, CH, bipy), 9.17 (m, CH, bipy); ^{14}N NMR (28.9 MHz, *o*-$C_6H_4F_2$, 298 K) δ = no signal; ^{19}F NMR (377 MHz, *o*-$C_6H_4F_2$, calibrated to *o*-$C_6H_4F_2$ = –139 ppm[2], 298 K) δ = –74.8 (s, CF_3, $[Al(OR^F)_4]^-$ anion); ^{27}Al NMR (104 MHz, *o*-$C_6H_4F_2$, calibrated to $[Al(OR^F)_4]^-$ = 33.8 ppm[3], 298 K) δ = 33.8 (s, $[Al(OR^F)_4]^-$ anion); ^{71}Ga NMR (122 MHz, *o*-$C_6H_4F_2$, 298 K) δ = no signal. UV-VIS (298 K, 333 µM in *o*-$C_6H_4F_2$) λ_{max} = 302 nm, 313 nm, 384 nm, 481 nm.

3.3. Preparation of $[In_3(bipy)_6]^{3+}\{[Al(OR^F)_4]^-\}_3$ (4)

A highly concentrated, yellow solution of **2** (100 mg, 78.5 µmol, 1.00 eq) and bipy (25.0 mg, 160 µmol, 2.04 eq) in C_6H_5F (200 µL) was stored at +25 °C. After eight months, yellow, platelet-shaped single crystals were isolated from the solution and characterized using single-crystal X-ray analysis and multinuclear NMR spectroscopy. Storing a more diluted solution in C_6H_5F (1.5 mL) at −40 °C, single crystals of identical composition were obtained after two days. ^{1}H NMR (300 MHz, *o*-$C_6H_4F_2$, calibrated to *o*-$C_6H_4F_2$ = 7.12 ppm[1], 298 K): δ = 7.62 (m, 2H, CH, bipy), 8.04 (m, 2H, CH, bipy), 8.34 (m, 2H, CH, bipy), 8.86 (m, 2H, CH, bipy); ^{14}N NMR (21.7 MHz, *o*-$C_6H_4F_2$, 298 K) δ = no signal; ^{19}F NMR (282 MHz, *o*-$C_6H_4F_2$, calibrated to *o*-$C_6H_4F_2$ = –139 ppm[2], 298 K) δ = –74.9 (s, CF_3, $[Al(OR^F)_4]^-$ anion); ^{27}Al NMR (78.2 MHz, *o*-$C_6H_4F_2$, calibrated to $[Al(OR^F)_4]^-$ = 33.8 ppm[3], 298 K) δ = 33.8 (s, $[Al(OR^F)_4]^-$ anion); ^{115}In NMR (65.7 MHz, *o*-$C_6H_4F_2$, 298 K) δ = no signal.

3.4. Preparation of $[In_3(bipy)_5]^{3+}\{[Al(OR^F)_4]^-\}_3$ (5)

A yellow solution of **2** (100 mg, 78.5 µmol, 1.00 eq) and bipy (20.0 mg, 128 µmol, 1.63 eq) in C_6H_5F (1.5 mL) was stored at −28 °C. After eight months, yellow, platelet-shaped single crystals were isolated from the solution and characterized using single-crystal X-ray analysis and multinuclear NMR spectroscopy. ^{1}H NMR (400 MHz, *o*-$C_6H_4F_2$, calibrated to *o*-$C_6H_4F_2$ = 7.12 ppm[1], 298 K): δ = 7.71 (m, 2H, CH, bipy), 8.12 (m, 2H, CH, bipy), 8.26 (m, 2H, CH, bipy), 8.90 (m, 2H, CH, bipy); ^{14}N NMR (28.9 MHz, *o*-$C_6H_4F_2$, 298 K) δ = no signal; ^{19}F NMR (377 MHz, *o*-$C_6H_4F_2$, calibrated to *o*-$C_6H_4F_2$ = –139 ppm[2], 298 K) δ = –74.9 (s, CF_3, $[Al(OR^F)_4]^-$ anion); ^{27}Al NMR (104 MHz, *o*-$C_6H_4F_2$, calibrated to $[Al(OR^F)_4]^-$ = 33.8 ppm[3], 298 K) δ = 33.8 (s, $[Al(OR^F)_4]^-$ anion).

3.5. Preparation of $[In_4(bipy)_6]^{4+}\{[Al(OR^F)_4]^-\}_4$ (6)

A yellow solution of **2** (100 mg, 78.5 µmol, 1.00 eq) and bipy (24.5 mg, 157 µmol, 2.00 eq) in C_6H_5F (0.5 mL) was slowly concentrated under reduced pressure and stored at +25 °C. After three days, yellow, platelet-shaped single crystals were isolated from the solution and characterized using single-crystal X-ray analysis and multinuclear NMR spectroscopy. ^{1}H NMR (400 MHz, *o*-$C_6H_4F_2$, calibrated to *o*-$C_6H_4F_2$ = 7.12 ppm[1], 298 K): δ = 7.50 (m, 2H, CH, bipy), 7.96 (m, 2H, CH, bipy), 8.05–8.19 (m_c, 2H, CH, bipy), 8.64 (m, 2H, CH, bipy); ^{14}N NMR (28.9 MHz, *o*-$C_6H_4F_2$, 298 K) δ = no signal; ^{19}F NMR (377 MHz, *o*-$C_6H_4F_2$, calibrated to *o*-$C_6H_4F_2$ = –139 ppm[2], 298 K) δ = –74.9 (s, CF_3,

[Al(OR^F)$_4$]$^-$ anion); ^{27}Al NMR (104 MHz, *o*-$C_6H_4F_2$, calibrated to [Al(OR^F)$_4$]$^-$ = 33.8 ppm[3], 298 K) δ = 33.8 (s, [Al(OR^F)$_4$]$^-$ anion); ^{115}In NMR (87.6 MHz, *o*-$C_6H_4F_2$, 298 K) δ = no signal.

3.6. Preparation of [In$_4$(phen)$_6$]$^{4+}${[Al(OR^F)$_4$]$^-$}$_4$ (7) and [(phen)$_2$In–Ag(FB)]$^{2+}${[Al(OR^F)$_4$]$^-$}$_2$ (8)

A yellow solution of **2** (100 mg, 78.5 µmol, 1.00 eq) and bipy (21.0 mg, 117 µmol, 1.49 eq) in C_6H_5F (1.5 mL) was stored at −28 °C. After eight months, yellow and colorless, platelet-shaped single crystals were isolated from the solution and characterized using single-crystal X-ray analysis and multinuclear NMR spectroscopy. The synthesis of **7** was reproduced by storing a solution of like composition at −40 °C for two months and the synthesis of **8** by storing a solution of **2** (200 mg, 157 µmol, 1.00 eq) and bipy (28.3 mg, 157 µmol, 1.00 eq) in C_6H_5F (1.5 mL) at −40 °C for 16 days. ^{1}H NMR (300 MHz, *o*-$C_6H_4F_2$, calibrated to *o*-$C_6H_4F_2$ = 7.12 ppm[1], 298 K): δ = 7.79 (m$_c$, CH, phen), 7.89 (m, CH, phen), 8.01 (m, CH, phen), 8.09 (m$_c$, CH, phen), 8.40 (m$_c$, CH, phen), 8.48 (m, CH, phen), 8.63 (m, CH, phen), 9.08 (m$_c$, CH, phen), 9.17 (m, CH, phen), 9.41 (m, CH, phen); ^{14}N NMR (21.7 MHz, *o*-$C_6H_4F_2$, 298 K) δ = no signal; ^{19}F NMR (282 MHz, *o*-$C_6H_4F_2$, calibrated to *o*-$C_6H_4F_2$ = –139 ppm[2], 298 K) δ = –74.9 (s, CF_3, [Al(OR^F)$_4$]$^-$ anion), −113 (tt, $^3J(^{19}F, ^1H)$ = 9.2 Hz, $^4J(^{19}F, ^1H)$ = 5.7 Hz, CF, C_6H_5F); ^{27}Al NMR (78.2 MHz, *o*-$C_6H_4F_2$, calibrated to [Al(OR^F)$_4$]$^-$ = 33.8 ppm[3], 298 K) δ = 33.8 (s, [Al(OR^F)$_4$]$^-$ anion); ^{115}In NMR (65.7 MHz, *o*-$C_6H_4F_2$, 298 K) δ = no signal.

3.7. Crystal Structure Data

Crystal structure data for **3** (CCDC 1032681): $C_{68}H_{27}Al_2F_{73.75}GaN_6O_8$, M_w = 2580.88 g mol^{-1}, monoclinic, space group *C2/c*, *a* = 25.1999(16) Å, *b* = 15.4651(16) Å, *c* = 24.792(2) Å, α = 90 ° β = 112.931(7)°, γ = 90 °, *V* = 8898.3(14) Å^3, *Z* = 4, ρ_{calc} = 1.927 g cm^{-3}, *F*(000) = 5047, λ = 0.71073 Å, *T* = 150(2) K, absorption coefficient = 0.541 mm^{-1}, absorption correction: multi-scan, T_{min} = 0.6995, T_{max} = 0.7452, GooF = 1.017, R_1 = 7.52 and wR_2 = 18.64 for reflections $I > 2\sigma(I)$, R_1 = 10.21 and wR_2 = 20.63 for all reflections.

Crystal structure data for **4** (CCDC 1032680): $C_{108}H_{48}Al_3F_{108}In_3N_{12}O_{12}$, M_w = 4182.98 g mol^{-1}, monoclinic, space group *P*2$_1$/*c*, *a* = 22.4009(11) Å, *b* = 30.7088(16) Å, *c* = 20.4177(10) Å, α = 90 °, β = 95.459(2)°, γ = 90 °, *V* = 13981.7(12) Å^3, *Z* = 4, ρ_{calc} = 1.987 g cm^{-3}, *F*(000) = 8136, λ = 0.71073 Å, *T* = 100(2) K, absorption coefficient = 0.716 mm^{-1}, absorption correction: multi-scan, T_{min} = 0.6307, T_{max} = 0.7456, GooF = 1.204, R_1 = 6.80 and wR_2 = 14.92 for reflections $I > 2\sigma(I)$, R_1 = 7.55 and wR_2 = 15.30 for all reflections.

Crystal structure data for **5** (CCDC 1033040): $C_{422}H_{184}Al_{12}F_{437}In_{12}N_{40}O_{48}$, M_w = 16586.68 g mol^{-1}, triclinic, space group *P*1;¯, *a* = 16.2891(6) Å, *b* = 25.6709(9) Å, *c* = 33.2531(12) Å, α = 89.470(2)°, β = 87.358(2)°, γ = 81.522(2)°, *V* = 13738.4(9) Å^3, *Z* = 1, ρ_{calc} = 2.005 g cm^{-3}, *F*(000) = 8057, λ = 0.71073 Å, *T* = 100(2) K, absorption coefficient = 0.729 mm^{-1}, absorption correction: multi-scan, T_{min} = 0.7081, T_{max} = 0.7456, GooF = 1.024, R_1 = 6.55 and wR_2 = 15.19 for reflections $I > 2\sigma(I)$, R_1 = 10.43 and wR_2 = 17.09 for all reflections.

Crystal structure data for **6** (CCDC 1032732): $C_{124}H_{48}Al_4F_{144}In_4N_{12}O_{16}$, M_w = 5264.94 g mol^{-1}, triclinic, space group *P*1;¯, *a* = 34.744(5) Å, *b* = 34.792(5) Å, *c* = 37.210(5) Å, α = 106.127(4)°, β = 101.609(4)°, γ = 118.938(3)°, *V* = 34671(8) Å^3, *Z* = 8, ρ_{calc} = 2.017 g cm^{-3}, *F*(000) = 20384, λ = 0.71073 Å, *T* = 100(2) K, absorption coefficient = 0.763 mm^{-1}, absorption correction: multi-scan, T_{min} = 0.5947, T_{max} = 0.7451, GooF = 1.078, R_1 = 7.40 and wR_2 = 16.91 for reflections $I > 2\sigma(I)$, R_1 = 15.50 and wR_2 = 21.57 for all reflections.

Crystal structure data for **7** (CCDC 1034231): $C_{86}H_{39}Al_2F_{75}In_2N_6O_8$, M_w =2992.83 g mol^{-1}, monoclinic, $P2_1/c$, a = 20.2872(5) Å, b = 28.7711(7) Å, c = 19.7149(6) Å, α = 90 °, β = 117.3030(13)°, γ = 90 °, V = 10225.3(5) Å^3, Z = 4, ρ_{calc} = 1.944 g cm^{-3}, F(000) = 5840, λ = 0.71073 Å, T = 100(2) K, absorption coefficient = 0.664 mm^{-1}, T_{min} = 0.6156, T_{max} = 0.7448, GooF = 1.272, R_1 = 9.00 and wR_2 = 22.32 for reflections $I > 2\sigma(I)$, R_1 = 13.47 and wR_2 = 25.24 for all reflections.

Crystal structure data for **8** (CCDC 1034089): $C_{80}H_{36}AgAl_2F_{76}InN_4O_8$, M_w =2901.78 g mol^{-1}, monoclinic, $P2_1/n$, a = 16.4785(8) Å, b = 23.7211(12) Å, c = 25.2293(13) Å, α = 90 °, β = 97.519(3)°, γ = 90 °, V = 9777.0(9) Å^3, Z = 4, ρ_{calc} = 1.971 g cm^{-3}, F(000) = 5656, λ = 0.71073 Å, T = 100(2) K, absorption coefficient = 0.656 mm^{-1}, T_{min} = 0.6266, T_{max} = 0.7449, GooF = 1.023, R_1 = 3.63 and wR_2 = 7.96 for reflections $I > 2\sigma(I)$, R_1 = 5.80 and wR_2 = 8.98 for all reflections.

4. Supplementary References

1 *^{1}H NMR Spectrum of o-$C_6H_4F_2$ (0.05 mL) in $CDCl_3$ (5 mL); JEOL AL-400 (399.65 MHz); SDBSWeb: http://sdbs.db.aist.go.jp/sdbs/cgi-bin/direct_frame_disp.cgi?sdbsno=4698 (National Institute of Advanced Industrial Science and Technology, 01.12.2014)* (2014).

2 Hesse, M., Meier, H. & Zeeh, B. *Spektroskopische Methoden in der Organischen Chemie*., Vol. 7 (Thieme Verlag, **2005**).

3 Krossing, I. The facile preparation of weakly coordinating anions: structure and characterisation of silver polyfluoroalkoxyaluminates $AgAl(OR^F)_4$, calculation of the alkoxide ion affinity. *Chem. Eur. J.* **7**, 490-502 (2001).

4 Jenkins, H. D. B., Roobottom, H. K., Passmore, J. & Glasser, L. Relationships among Ionic Lattice Energies, Molecular (Formula Unit) Volumes, and Thermochemical Radii. *Inorg. Chem.* **38**, 3609-3620 (1999).

5 Ribeiro da Silva, M. A. V., Morais, V. M. F., Matos, M. A. R. & Rio, C. M. A. Thermochemical and Theoretical Studies of Some Bipyridines. *J. Org. Chem.* **60**, 5291-5294 (1995).

6 Majer, V. & Václav, S. *Enthalpies of vaporization of organic compounds : a critical review and data compilation*. 300 (Blackwell Scientific, 1985).

7 van der Sluis, P. & Spek, A. L. BYPASS: an effective method for the refinement of crystal structures containing disordered solvent regions. *Acta Cryst., Sect. A: Found. Crystallogr.* **46**, 194-201 (1990).

8 Spek, A. PLATON, An Integrated Tool for the Analysis of the Results of a Single Crystal Structure Determination. *Acta Cryst., Sect. A: Found. Crystallogr.* **46**, c34 (1990).

9 Bondi, A. van der Waals Volumes and Radii. *J. Phys. Chem.* **68**, 441-451 (1964).

10 Pal, A. *et al.* Structures of viscotoxins A1 and B2 from European mistletoe solved using native data alone. *Acta Crystallogr. Sect. D* **64**, 985-992 (2008).

11 van der Vorst, C. P. J. M., Verschoor, G. C. & Maaskant, W. J. A. The structures of yellow and red indium monochloride. *Acta Crystallogr., Sect. B: Struct. Sci.* **34**, 3333-3335 (1978).

12 Shannon, R. D. & Prewitt, C. T. Effective ionic radii in oxides and fluorides. *Acta Crystallogr., Sect. B: Struct. Sci.* **25**, 925-946 (1969).

13 Fais, E., Borrmann, H., Mattausch, H. & Simon, A. Kristallstrukturen zweier Modifikationen von $In_5Mo_{18}O_{28}$ und deren Zwillingsbildung. *Z. Anorg Allg. Chem.* **621**, 1178-1184 (1995).

14 Mattausch, H., Simon, A. & Peters, E. M. Indium molybdate, $In_{11}Mo_{40}O_{62}$: a molybdate with units of four and five condensed Mo_6 octahedra and linear In_5^{7+} and In_6^{8+} ions. *Inorg. Chem.* **25**, 3428-3433 (1986).

15 Peltier, V., L'Haridon, P., Marchand, R. & Laurent, Y. Synthese et caracterisation structurale d'un phosphate d'indium $In_3P_2O_8$ presentant des paires In-In. *Acta Crystallogr., Sect. B: Struct. Sci.* **52**, 905-908 (1996).

16 Thauern, H. & Glaum, R. Synthesis, Crystal Structure Determination and Vibrational Spectra of Indium(II) Indium(III) Oxidephosphate, $(In_2)^{4+}(In^{3+})_2O_2(PO_4)_2$. *Z. Anorg Allg. Chem.* **630**, 2463-2467 (2004).

17 Uhl, W., Lawerenz, A. & Zemke, S. Terminale und verbrückende Koordination von Indium-Indium-Bindungen – bemerkenswerte Polymorphie an der Verbindung $In_2R_2[(OCC_6H_5)_2CH]_2$ [R = $C(SiMe_3)_3$]. *Z. Anorg Allg. Chem.* **633**, 979-985 (2007).

18 Uhl, W. & El-Hamdan, A. Reactivity of Organoelement Subhalides of Gallium and Indium — Ga–Ga and In−In Bonds Bridged by Carboxylato Ligands. *Eur. J. Inorg. Chem.* **2004**, 969-972 (2004).
19 Allan, C. J., Cooper, B. F. T., Cowley, H. J., Rawson, J. M. & Macdonald, C. L. B. Non-Innocent Ligand Effects on Low-Oxidation-State Indium Complexes. *Chem. Eur. J.* **19**, 14470-14483 (2013).
20 Scholten, M., Dronskowski, R., Staffel, T. & Meyer, G. Synthesis and Crystal Structure of Potassium Indium Tribromide, $KInBr_3$. *Z. Anorg Allg. Chem.* **624**, 1741-1745 (1998).
21 Andrews, C. G. & Macdonald, C. L. B. Crown Ether Ligation: An Approach to Low-Oxidation-State Indium Compounds. *Angew. Chem.* **117**, 7619-7622 (2005).
22 Andrews, C. G. & Macdonald, C. L. B. Crown Ether Ligation: An Approach to Low-Oxidation-State Indium Compounds. *Angew. Chem. Int. Ed.* **44**, 7453-7456 (2005).
23 Cooper, B. F. T. *et al.* “Crowned” Univalent Indium Complexes as Donors? Experimental and Computational Insights on the Valence Isomers of $EE'X_4$ Species. *Chem. Eur. J.* **17**, 6148-6161 (2011).
24 Staffel, T. & Meyer, G. The mono-, sesqui-, and dibromides of indium: InBr, In_2Br_3, and $InBr_2$. *Z. Anorg Allg. Chem.* **552**, 113-122 (1987).
25 Brothers, P. J. *et al.* $In(InTrip_2)_3$: ein neuartiger In_4-Cluster mit kurzen In-In-Bindungen und trigonal-planarer Geometrie. *Angew. Chem.* **108**, 2528-2530 (1996).
26 Brothers, P. J. *et al.* A New In_4 Cluster with Short In−In Bonds in Trigonal-Planar $In(InTrip_2)_3$. *Angew. Chem. Int. Ed. Engl.* **35**, 2355-2357 (1996).
27 Staffel, T. & Meyer, G. In_5Br_7, the second mixed-valence In(I)−In(II) bromide: $In_3^{I}[In_2^{II}Br_6]Br$. With an appendix on a structure refinement of $InBr_3$. *Z. Anorg Allg. Chem.* **563**, 27-37 (1988).
28 Ruck, M. & Bärnighausen, H. Zur Polymorphie von In_5Br_7. *Z. Anorg Allg. Chem.* **625**, 577-585 (1999).
29 Scholten, M., Kölle, P. & Dronskowski, R. Temperature-dependent diffraction studies on the phase evolution of tetraindium heptabromide. *J. Solid State Chem.* **174**, 349-356 (2003).
30 Jin, G., Jones, C., Junk, P. C., Stasch, A. & Woodul, W. D. Group 13 metal(I) and (II) guanidinate complexes: effect of ligand backbone on metal oxidation state and coordination sphere. *New J. Chem.* **32**, 835-842 (2008).
31 Gabbai, F. P., Schier, A., Riede, J. & Schmidbaur, H. Different Pathways of the Reaction of InCl with Ph_3PAuCl: Isolation of the First Mixed-Valent Mixed-Metal Gold/Indium Cluster. *Inorg. Chem.* **34**, 3855-3856 (1995).
32 Lomelí, V., McBurnett, B. G. & Cowley, A. H. An indium(II)-indium(II) compound with intramolecular donor-acceptor bonds. *J. Organomet. Chem.* **562**, 123-125 (1998).
33 Tian, X., Pape, T. & Mitzel, N. W. Formamidinium salts of low valent metal halide anions MX_3^- (M = Ge, Sn) and $M_2X_6^{2-}$ (M = Ga, In). *Z. Naturforsch., B: Chem. Sci.* **59**, 1524-1531 (2004).
34 Deiseroth, H.-J., Reiner, C., Xhaxhiu, K., Schlosser, M. & Kienle, L. X-Ray and Transmission Electron Microscopy Investigations of the New Solids In_5S_5Cl, In_5Se_5Cl, In_5S_5Br, and In_5Se_5Br. *Z. Anorg Allg. Chem.* **630**, 2319-2328 (2004).
35 Hellmann, K. W. *et al.* Ligandenoxidation statt Redoxdisproportionierung: Thallium(I)-induzierte Synthese von 4,9-Diaminoperylen-3,10-chinondiiminen. *Angew. Chem.* **110**, 2053-2057 (1998).

36 Hellmann, K. W. *et al.* Metal–Ligand versus Metal–Metal Redox Chemistry: Thallium(I)-Induced Synthesis of 4,9-Diaminoperylenequinone-3,10-diimine Derivatives. *Angew. Chem. Int. Ed.* **37**, 1948-1952 (1998).

37 Bubenheim, W., Frenzen, G. & Muller, U. Die Chloroindate $[PPh_4]_2[In_2Cl_6]$ und $[PPh_4]_2[InCl_5].CH_3CN$. *Acta Crystallogr., Sect. C: Cryst. Struct. Commun.* **51**, 1120-1124 (1995).

38 Klimek, K. S., Cui, C., Roesky, H. W., Noltemeyer, M. & Schmidt, H.-G. Synthesis and Characterization of 1-Aza-allyl Complexes with Al−Al, Ga−Ga, and In−In Bonds. *Organometallics* **19**, 3085-3090 (2000).

39 Baker, R. J., Farley, R. D., Jones, C., Kloth, M. & Murphy, D. M. Synthesis and characterisation of the first carbene and diazabutadiene-indium(II) complexes. *Chem. Commun.*, 1196-1197 (2002).

40 Reiner, C., Deiseroth, H.-J., Schlosser, M. & Kienle, L. Die neuen gemischtvalenten Chalkogenoindate MIn_7X_9 (M = Rb, Cs; X = S, Se): Strukturchemie, Röntgenund HRTEM-Untersuchungen. *Z. Anorg Allg. Chem.* **628**, 249-257 (2002).

41 von Hänisch, C., Fenske, D., Kattannek, M. & Ahlrichs, R. $[In_3(In_2)_3(PhP)_4(Ph_2P_2)_3Cl_7(PEt_3)_3]$ – eine neue molekulare III/V-Verbindung mit einem ungewöhnlichen 19atomigen Polyedergerüst. *Angew. Chem.* **111**, 2900-2902 (1999).

42 von Hänisch, C., Fenske, D., Kattannek, M. & Ahlrichs, R. $[In_3(In_2)_3(PhP)_4(Ph_2P_2)_3Cl_7(PEt_3)_3]$ – A New Molecular III/V Compound Featuring an Unusual 19-Atom Cage. *Angew. Chem. Int. Ed.* **38**, 2736-2738 (1999).

43 Hogg, J. H. C. & Duffin, W. J. The crystal structure of In_6S_7. *Acta Crystallogr.* **23**, 111-118 (1967).

44 Wagner, G. & Hoppe, R. A new cluster type: In_5^{6+} in $Na_{24}In_5O_{15}$. *J. Less Common Met.* **116**, 129-136 (1986).

45 Schluter, R. D. *et al.* Use of the 2,4,6-tris(trifluoromethyl)phenyl ligand to stabilize indium-indium and gallium-gallium bonds. *J. Am. Chem. Soc.* **115**, 2070-2071 (1993).

46 M. Godfrey, S., J. Kelly, K., Kramkowski, P., A. McAuliffe, C. & G. Pritchard, R. Reaction of indium metal with R_3PI_2 (R = Ph, Pr^i, Pr^n); structural characterisation of the novel 'subvalent' indium(II) complex $In_2I_4(PPr^n{}_3)_2$ which contains an In-In bond, the four- and five-coordinate indium(III) complex $InI_3(PPh_3)_2{\cdot}InI_3(PPh_3)$, and the tetrahedral indium(III) complex $InI_3(PPr^i{}_3)$. *Chem. Commun.*, 1001-1002 (1997).

47 Frazer, A., Hodge, P. & Piggott, B. Novel redox properties of $HB(3\text{-}Bu^tpz)_3^-$ as shown by its reaction with In and Sn. *Chem. Commun.*, 1727-1728 (1996).

48 Cole, M. L., Jones, C. & Kloth, M. Controlled Decomposition of an Indium Trihydride Adduct: Synthesis and Characterization of the First Mixed-Oxidation-State Indium Sub-halide Complex Anion, $[In_5Br_8(quinuclidine)_4]$. *Inorg. Chem.* **44**, 4909-4911 (2005).

49 Green, S. P., Jones, C. & Stasch, A. "Dissolution" of Indium(I) Iodide: Synthesis and Structural Characterization of the Neutral Indium Sub-Halide Cluster Complex $[In_6I_8(tmeda)_4]$. *Angew. Chem.* **119**, 8772-8775 (2007).

50 Green, S. P., Jones, C. & Stasch, A. "Dissolution" of Indium(I) Iodide: Synthesis and Structural Characterization of the Neutral Indium Sub-Halide Cluster Complex $[In_6I_8(tmeda)_4]$. *Angew. Chem. Int. Ed.* **46**, 8618-8621 (2007).

51 Cheng, Y., Doyle, D. J., Hitchcock, P. B. & Lappert, M. F. The β-dialdiminato ligand $[\{N(C_6H_3Pr^i2\text{-}2,6)C(H)\}_2CPh]^-$: the conjugate acid and Li, Al, Ga and In derivatives. *Dalton Trans.*, 4449-4460 (2006).

52 Mansaray, H. B., Kelly, M., Vidovic, D. & Aldridge, S. Tuning Main Group Redox Chemistry through Steric Loading: Subvalent Group 13 Metal Complexes of Carbazolyl Ligands. *Chem. Eur. J.* **17**, 5381-5386 (2011).

53 Hogg, J. The crystal structure of In_6Se_7. *Acta Crystallogr., Sect. B: Struct. Sci.* **27**, 1630-1634 (1971).

54 Fryzuk, M. D., Giesbrecht, G. R., Rettig, S. J. & Yap, G. P. A. Synthesis and characterization of Group 13 hydrides and metal−metal bonded dimers stabilized by the macrocyclic bis(amidophosphine) ligand $[P_2N_2]$ ($[P_2N_2]$ = $[PhP(CH_2SiMe_2NSiMe_2CH_2)_2PPh]$). *J. Organomet. Chem.* **591**, 63-70 (1999).

55 Wadsten, T., Arnberg, L. & Berg, J. E. The structure of pentaindium tetrasulfide. *Acta Crystallogr., Sect. B: Struct. Sci.* **36**, 2220-2223 (1980).

56 Veith, M., Goffing, F., Becker, S. & Huch, V. Spezielle Silazanderivate von Digallan(4) and Diindan(4) mit Ga−Ga- bzw. In−In-Bindung. *J. Organomet. Chem.* **406**, 105-118 (1991).

57 Hogg, J. H. C., Sutherland, H. H. & Williams, D. J. The crystal structure of tetraindium triselenide. *Acta Crystallogr., Sect. B: Struct. Sci.* **29**, 1590-1593 (1973).

58 Wiberg, N., Blank, T., Purath, A., Stößer, G. & Schnöckel, H. Hexasupersilyloctaindan $(t\mathrm{Bu}_3\mathrm{Si})_6\mathrm{In}_8$ – eine Verbindung mit einem neuartigen In_8-Clustergerüst. *Angew. Chem.* **111**, 2745-2748 (1999).

59 Wiberg, N., Blank, T., Purath, A., Stößer, G. & Schnöckel, H. Hexasupersilyloctaindane $(t\mathrm{Bu}_3\mathrm{Si})_6\mathrm{In}_8$ – A Compound with a Novel In_8-Cluster Framework. *Angew. Chem. Int. Ed.* **38**, 2563-2565 (1999).

60 Grocholl, L., Schranz, I., Stahl, L. & Staples, R. J. Syntheses and Molecular Structures of Bis(tert-butylamido)cyclodiphosph(III)azane Cage Complexes of Thallium(I) and Indium(II). *Inorg. Chem.* **37**, 2496-2499 (1998).

61 Khan, M. A., Peppe, C. & Tuck, D. G. The crystal and molecular structure of the bis(N,N,N',N'-tetramethylethanediamine) adduct of $I(Br)InInBr_2$, $In_2Br_3I\bullet 2C_6H_{16}N_2$, an indium–indium bonded molecule. *Can. J. Chem.* **62**, 601-605 (1984).

62 Protchenko, A. V. *et al.* Stable GaX_2, InX_2 and TlX_2 radicals. *Nat. Chem.* **6**, 315-319 (2014).

63 Serrano, O., Fettinger, J. C. & Power, P. P. Synthesis and molecular structures of the 1,2-dihalogen derivatives of Ga(II) and In(II), $[\{GaI(ArMe_6)\}_2]$, $[\{InCl(ArMe_6)\}_2]_2$, $[\{InI(ArMe_6)\}_2]_2$, and $[\{In_4Cl_2I_2(ArMe_6)_4\}]$, $ArMe_6C_6H_3$-2,6$(C_6H_2$-2,4,6-$Me_3)_2$. *Polyhedron* **58**, 144-150 (2013).

64 Uhl, W., El-Hamdan, A. & Lawerenz, A. Investigations into the Reactivity of Organoelement Gallium and Indium Subhalides – Syntheses of Digallium and Diindium Acetylacetonates. *Eur. J. Inorg. Chem.* **2005**, 1056-1062 (2005).

65 Linti, G., Buhler, M., Monakhov, K. Y. & Zessin, T. Synthesis and structure of Tri- and octaindium compounds. *Dalton Trans.*, 8071-8078 (2009).

66 Hellmann, K. W., Gade, L. H., Steiner, A., Stalke, D. & Möller, F. Aggregation und Redoxdisproportionierungen in tripodalen In- und Tl-Amiden: erstmalige Charakterisierung von gemischtvalenten M^{I}/M^{II}-Verbindungen (M = In, Tl). *Angew. Chem.* **109**, 99-102 (1997).

67 Hellmann, K. W., Gade, L. H., Steiner, A., Stalke, D. & Möller, F. Aggregation and Redox-Disproportionation in Tripodal Indium and Thallium Amides: First Characterization of Mixed-Valent M^{I}/M^{II} Compounds (M = In, Tl). *Angew. Chem. Int. Ed. Engl.* **36**, 160-163 (1997).

68 Uhl, W., Melle, S., Geiseler, G. & Harms, K. $In_3I_2[C(SiMe_3)_3]_3$: Synthesis of a Diiodotrialkyltriindane(5) Containing Two In−In Single Bonds. *Organometallics* **20**, 3355-3357 (2001).
69 Wiberg, N., Blank, T., Nöth, H. & Ponikwar, W. Dodecaindan $(t\mathrm{Bu_3Si})_8In_{12}$ – eine Verbindung mit einem In_{12}-Deltapolyeder-Gerüst. *Angew. Chem.* **111**, 887-890 (1999).
70 Wiberg, N., Blank, T., Nöth, H. & Ponikwar, W. Dodecaindane $(t\mathrm{Bu_3Si})_8In_{12}$ – A Compound with an In_{12} Deltapolyhedron Framework. *Angew. Chem. Int. Ed.* **38**, 839-841 (1999).
71 Hill, M. S., Hitchcock, P. B. & Pongtavornpinyo, R. A Linear Homocatenated Compound Containing Six Indium Centers. *Science* **311**, 1904-1907 (2006).
72 Uhl, W., Schmock, F. & Geiseler, G. $[In_3Br_3\{C(SiMe_3)_3\}_3][Li(THF)_3]$ mit einer Kette aus drei Indiumatomen — ein bemerkenswertes Nebenprodukt aus der Synthese des Tetraindiumclusters $In_4[C(SiMe_3)_3]_4$. *Z. Anorg Allg. Chem.* **628**, 1963-1966 (2002).
73 Likforman, A., Carre, D., Etienne, J. & Bachet, B. Structure cristalline du monoseleniure d'indium InSe. *Acta Crystallogr., Sect. B: Struct. Sci.* **31**, 1252-1254 (1975).
74 Uhl, W. & Melle, S. Alkylindium Subhalides Derived from the Tetrahedral Indium(I) Cluster Compound $[In_4\{C(SiMe_3)_3\}_4]$. *Chem. Eur. J.* **7**, 4216-4221 (2001).
75 Uhl, W., Layh, M. & Hiller, W. Tetrakis[bis(trimethylsilyl)methyl]diindan(4) mit Indium−Indium-Bindung. *J. Organomet. Chem.* **368**, 139-154 (1989).
76 Stender, M. & Power, P. P. Reaction of $\{HC(CMeNAr)_2\}Li$ (Ar=2,6-*i*-$Pr_2C_6H_3$) with indium monochloride to yield the In−In bonded dimer $[\{HC(CMeNAr)_2\}InCl]_2$ and the hydroxide $[\{HC(CMeNAr)_2\}InCl(\mu\text{-}OH)]_2$. *Polyhedron* **21**, 525-529 (2002).
77 Wendorff, M. & Röhr, C. New Mixed Sr/Ba Trielides $A^{II}In_xM^{III}{}_{1-x}$ (M = Ga, Al). *Z. Naturforsch.* **65b**, 1-15 (2008).
78 Sevov, S. C. & Corbett, J. D. Synthesis, Characterization, and Bonding of Indium Cluster Phases: $Na_{15}In_{27.4}$, a Network of In_{16} and In_{11} Clusters; Na_2In with Isolated Indium Tetrahedra. *J. Solid State Chem.* **103**, 114-130 (1993).
79 Sevov, S. C. & Corbett, J. D. Synthesis, characterization, and bonding of indium clusters: $Na_7In_{11.8}$, a novel network structure containing closo-In_{16} and nido-In_{11} clusters. *Inorg. Chem.* **31**, 1895-1901 (1992).
80 Uhl, W., Hannemann, F. & Wartchow, R. Reactions of Tetraalkyldiindane(4), $R_2In{-}InR_2$ (R = $CH(SiMe_3)_2$), with tert-Butyl and Phenyl Isonitriles. Formation of Adducts with Retention of the In−In Bond. *Organometallics* **17**, 3822-3825 (1998).
81 Saltykov, V., Nuss, J., Wedig, U., Prasad, Dasari L. V. K. & Jansen, M. First Isolated "Hypoelectronic" $[In_6]^{6-}$ Cluster in Insulating $Cs_{22}In_6(SiO_4)_4$. *Z. Anorg. Allg. Chem.* **637**, 834-839 (2011).
82 Uhl, W. & Spies, T. Umsetzungen der Dielementverbindungen $R_2E{-}ER_2$ [E = Ga, In; R = $CH(SiMe_3)_2$] mit Lithium-phenylethinid – Bildung von Addukten unter Erhalt der E–E-Bindungen. *Z. Anorg Allg. Chem.* **626**, 1059-1064 (2000).
83 Li, B. & Corbett, J. D. Synthesis and Characterization of the New Cluster Phase K39In80. Three K−In Compounds with Remarkably Specific and Transferable Cation Dispositions. *Inorg. Chem.* **42**, 8768-8772 (2003).
84 Eichler, B. E., Hardman, N. J. & Power, P. P. $In_8(C_6H_3\text{-}2,6\text{-}Mes_2)_4$ (Mes=C_6H_2-2,4,6-Me_3): A Metal-Rich Main-Group Cluster with a Distorted Cubane Structure. *Angew. Chem.* **112**, 391-393 (2000).

85 Eichler, B. E., Hardman, N. J. & Power, P. P. $In_8(C_6H_3$-2,6-$Mes_2)_4$ (Mes=C_6H_2-2,4,6-Me_3): A Metal-Rich Main-Group Cluster with a Distorted Cubane Structure. *Angew. Chem. Int. Ed.* **39**, 383-385 (2000).
86 Slavi Sevov, C. & Corbett, J. D. Synthesis, characterization, and bonding of indium clusters. Rb_2In_3, a zintl phase with layers of closo-indium octahedra. *Z. Anorg Allg. Chem.* **619**, 128-132 (1993).
87 Wochele, R., Schwarz, W., Klinkhammer, K. W., Locke, K. & Weidlein, J. Neue Hypersilanide der Erdmetalle Aluminium, Gallium und Indium. *Z. Anorg Allg. Chem.* **626**, 1963-1973 (2000).
88 Bühler, M. & Linti, G. Synthese und Struktur des tetrameren Tris(trimethylsilyl)silylindium(I) und neuer silylsubstituierter Indiumverbindungen. *Z. Anorg Allg. Chem.* **632**, 2453-2460 (2006).
89 Sevov, S. C. & Corbett, J. D. Carbon-Free Fullerenes: Condensed and Stuffed Anionic Examples in Indium Systems. *Science* **262**, 880-883 (1993).
90 Sevov, S. C. & Corbett, J. D. A New Indium Phase with Three Stuffed and Condensed Fullerane-Like Cages: $Na_{172}In_{197}Z_2$ (*Z*= Ni, Pd, Pt). *J. Solid State Chem.* **123**, 344-370 (1996).
91 Wiberg, N., Amelunxen, K., Nöth, H., Schmidt, M. & Schwenk, H. Tetrasupersilyldiindium(In-In) und -dithallium(*Tl-Tl*): $(tBu_3Si)_2M$—$M(SitBu_3)_2$ (M = In, Tl). *Angew. Chem.* **108**, 110-112 (1996).
92 Wiberg, N., Amelunxen, K., Nöth, H., Schmidt, M. & Schwenk, H. Tetrasupersilyldiindium(In–In) and Tetrasupersilyldithallium(*Tl-Tl*): $(tBu_3Si)_2M$—$M(SitBu_3)_2$ (M = In, Tl). *Angew. Chem. Int. Ed. Engl.* **35**, 65-67 (1996).
93 Wiberg, N. *et al.* Ditrielanes $(R_3Si)_2E$–$E(SiR_3)_2$ and Heterocubanes $(R_3Si)_4E_4Y_4$ (R_3Si = tBu_3Si, tBu_2PhSi; E = Al, Ga, In, Tl; Y = O, Se). *Eur. J. Inorg. Chem.* **2002**, 341-350 (2002).
94 Ponou, S. *et al.* Synthesis, Characterization, and Electronic Structure of $Ba_5In_4Bi_5$: An Acentric and One-Electron Deficient Phase. *Chem. Eur. J.* **10**, 3615-3621 (2004).
95 Sevov, S. C. & Corbett, J. D. A remarkable hypoelectronic indium cluster in K_8In_{11}. *Inorg. Chem.* **30**, 4875-4877 (1991).
96 Wright, R. J., Phillips, A. D., Hardman, N. J. & Power, P. P. The "Diindene" ArInInAr (Ar = C_6H_3-2,6-$Dipp_2$, Dipp = C_6H_3-2,6-Pr^i_2). Dimeric versus Monomeric In(I) Aryls: para-Substituent Effects in Terphenyl Ligands. *J. Am. Chem. Soc.* **124**, 8538-8539 (2002).
97 Schluter, R. D., Cowley, A. H., Atwood, D. A., Jones, R. A. & Atwood, J. L. AN ALKYL-SUBSTITUTED INDIUM(I) TETRAMER. *J. Coord. Chem.* **30**, 25-28 (1993).
98 Uhl, W., Graupner, R., Layh, M. & Schütz, U. $In_4\{C(SiMe_3)_3\}_4$ mit In_4-tetraeder und $In_4Se_4\{C(SiMe_3)_3\}_4$ mit In_4Se_4-heterocubanstruktur. *J. Organomet. Chem.* **493**, C1-C5 (1995).
99 Zhao, J.-T. & Corbett, J. D. Square pyramidal clusters in La_3In_5 and Y_3In_5. La_3In_5 as a metallic Zintl phase. *Inorg. Chem.* **34**, 378-383 (1995).
100 Atwood, J. L. *et al.* The chemistry of trichloro[tris(trimethylsilyl)methyl] and trichloro[tris(dimethylphenylsilyl)methyl] complexes of gallium, indium, and thallium. Crystal and molecular structures of $[Li(thf)_2(\mu\text{-}Cl)_2GaCl\{C(SiMe_2Ph)_3\}]\cdot thf$, $[Li(thf)_3(\mu\text{-}Cl)InCl_2\{C(SiMe_3)_3\}]$, and $[\{(Me_3Si)_3C\}In(\mu\text{-}Cl)_2\{\mu\text{-}Fe(CO)_4\}In\{C(SiMe_3)_3\}]$ (thf = tetrahydrofuran). *J. Chem. Soc., Dalton Trans.*, 747-755 (1987).
101 Hill, M. S., Hitchcock, P. B. & Pongtavornpinyo, R. Dimerization of Indanediyl Fragments: An Alkene Analogue for Group 13? *Angew. Chem.* **117**, 4303-4307 (2005).

102 Hill, M. S., Hitchcock, P. B. & Pongtavornpinyo, R. Dimerization of Indanediyl Fragments: An Alkene Analogue for Group 13? *Angew. Chem. Int. Ed.* **44**, 4231-4235 (2005).

103 Dean, J. A. *Lange's Handbook of Chemistry, Fourteenth Edition*. (McGraw-Hill, 1992).

104 Rahbarnoohi, H., Wells, R. L. & Rheingold, A. L. Synthesis of a novel indium-nitrogen cage compound; molecular structure of $[[(Np)In\{\mu\text{-}(NH)_2C_6H_4\}]_4$ (Np = CH_2CMe_3). *Chem. Commun.*, 2661-2662 (1996).

105 Welsch, S., Bodensteiner, M., Dušek, M., Sierka, M. & Scheer, M. A Novel Soluble InI Precursor for Pn Ligand Coordination Chemistry. *Chem. Eur. J.* **16**, 13041-13045 (2010).

106 Van Den Berg, J. M. The crystal structure of the room temperature modification of indium chloride, InCl. *Acta Crystallogr.* **20**, 905-910 (1966).

107 Köhler, J., Chang, J.-H. & Whangbo, M.-H. Bonding and Oxidation State of a Transition Metal Atom Encapsulated in an Isolated Octahedral Cluster Cation of Main Group Elements: Synthesis, Crystal Structure, and Electronic Structure of Pt2In14Ga3O8F15 Containing Highly Positive 18-Electron Complex $[PtIn_6]^{10+}$ and Low-Valent In+ Ions. *J. Am. Chem. Soc.* **127**, 2277-2284 (2005).

108 Beck, H. P. & Wilhelm, D. In_7Cl_9 - eine neue „alte" Verbindung im System In-Cl. *Angew. Chem.* **103**, 897-898 (1991).

109 Beck, H. P. & Wilhelm, D. In_7Cl_9—A New "Old" Compound in the System In-Cl. *Angew. Chem. Int. Ed. Engl.* **30**, 824-825 (1991).

110 Schumann, H., Janiak, C., Görlitz, F., Loebel, J. & Dietrich, A. Synthesis and crystal structure of pentabenzylcyclopentadienylindium(I). *J. Organomet. Chem.* **363**, 243-251 (1989).

111 Green, S. P., Jones, C. & Stasch, A. Synthesis and structural characterisation of a soluble, metastable indium(I) halide complex, [InBr(tmeda)]. *Chem. Commun.*, 6285-6287 (2008).

112 Beachley, O. T., Churchill, M. R., Fettinger, J. C., Pazik, J. C. & Victoriano, L. Synthesis and crystal and molecular structure of $In(C_5Me_5)$ - an apparent octahedral cluster. *J. Am. Chem. Soc.* **108**, 4666-4668 (1986).

113 Beachley, O. T. *et al.* (Pentamethylcyclopentadienyl)indium(I) and -indium(III) compounds. Syntheses, reactivities, and x-ray diffraction and electron diffraction studies of $In(C_5Me_5)$. *Organometallics* **8**, 346-356 (1989).

114 Beachley, O. T. *et al.* Synthesis, characterization and structural studies of $In(C_5H_4Me)$ by x-ray diffraction and electron diffraction techniques and a reinvestigation of the crystalline state of $In(C_5H_5)$ by x-ray diffraction studies. *Organometallics* **7**, 1051-1059 (1988).

115 SAINT, Bruker AXS, V8.34A (Madison, Wisconsin, USA, 2013).

116 SADABS 2012/1 (University of Goettingen, Germany, 2012).

117 SADABS 2014/3 (University of Goettingen, Germany, 2014).

118 SHELXT - Crystal Structure Solution, 2013/6 (BETA TEST) (University of Goettingen, Germany, 2013).

119 Sheldrick, G. M. A short history of SHELX. *Acta Cryst., Sect. A: Found. Crystallogr.* **64**, 112-122 (2008).

120 Hubschle, C. B., Sheldrick, G. M. & Dittrich, B. ShelXle: a Qt graphical user interface for SHELXL. *J. Appl. Cryst.* **44**, 1281-128 (2011).

121 Disordered Solvent Refinement (DSR), www.xs3.uni-freiburg.de/research/dsr, 1.3.2 (University of Freiburg, Germany, 2014).

122 Diamond - Crystal and Molecular Structure Visualization, 3.2i (Crystal Impact GbR, Bonn Germany, **2012**).

123 Harris, R. K., Becker, E. D., Cabral de Menezes, S. M., Goodfellow, R. & Granger, P. NMR nomenclature. Nuclear spin properties and conventions for chemical shifts (IUPAC Recommendations 2001). *Pure Appl. Chem.* **73**, 1795-1818 (2001).

124 Stesmans, A. & Van Gorp, G. Improved measurement of the g factor of conduction electrons in Li particles embedded in LiF:Li. *Phys. Lett. A* **139**, 95-98 (1989).

125 Stoll, S. & Schweiger, A. EasySpin, a comprehensive software package for spectral simulation and analysis in EPR. *J. Magn. Reson.* **178**, 42-55 (2006).

126 Ahlrichs, R., Bär, M., Häser, M., Horn, H. & Kömel, C. Electronic structure calculations on workstation computers: The program system turbomole. *Chem. Phys. Lett.* **162**, 165-169 (1989).

127 Eichkorn, K., Treutler, O., Öhm, H., Häser, M. & Ahlrichs, R. Auxiliary basis sets to approximate Coulomb potentials. *Chem. Phys. Lett.* **240**, 283-290 (1995).

128 Eichkorn, K., Treutler, O., Öhm, H., Häser, M. & Ahlrichs, R. Auxiliary basis sets to approximate Coulomb potentials (Chem. Phys. Letters 240 (1995) 283-290). *Chem. Phys. Lett.* **242**, 652-660 (1995).

129 Treutler, O. & Ahlrichs, R. Efficient molecular numerical integration schemes. *J. Chem. Phys.* **102**, 346-354 (1995).

130 Eichkorn, K., Weigend, F., Treutler, O. & Ahlrichs, R. Auxiliary basis sets for main row atoms and transition metals and their use to approximate Coulomb potentials. *Theor. Chem. Acc.* **97**, 119-124 (1997).

131 von Arnim, M. & Ahlrichs, R. Performance of parallel TURBOMOLE for density functional calculations. *J. Comput. Chem.* **19**, 1746-1757 (1998).

132 Deglmann, P., Furche, F. & Ahlrichs, R. An efficient implementation of second analytical derivatives for density functional methods. *Chem. Phys. Lett.* **362**, 511-518 (2002).

133 Deglmann, P. & Furche, F. Efficient characterization of stationary points on potential energy surfaces. *J. Chem. Phys.* **117**, 9535-9538 (2002).

134 Deglmann, P., May, K., Furche, F. & Ahlrichs, R. Nuclear second analytical derivative calculations using auxiliary basis set expansions. *Chem. Phys. Lett.* **384**, 103-107 (2004).

135 Weigend, F. Accurate Coulomb-fitting basis sets for H to Rn. *Phys. Chem. Chem. Phys.* **8**, 1057-1065 (2006).

136 Dirac, P. A. M. Quantum Mechanics of Many-Electron Systems. *Proceedings A* **123**, 714-733 (1929).

137 Slater, J. C. A Simplification of the Hartree-Fock Method. *Phys. Rev.* **81**, 385-390 (1951).

138 Lee, C., Yang, W. & Parr, R. G. Development of the Colle-Salvetti correlation-energy formula into a functional of the electron density. *Phys. Rev. B* **37**, 785-789 (1988).

139 Becke, A. D. Density-functional exchange-energy approximation with correct asymptotic behavior. *Phys. Rev. A* **38**, 3098-3100 (1988).

140 Becke, A. D. A new mixing of Hartree‐Fock and local density ‐ functional theories. *J. Chem. Phys.* **98**, 1372-1377 (1993).
