# Supplementary material for: Cationic cluster formation versus disproportionation of low-valent indium and gallium complexes of 2,2'-bipyridine
Source: Nat Commun. 2015 Oct 19;6:8288. doi: 10.1038/ncomms9288 (PMC4633986; doi:10.1038/ncomms9288)
Supplement: Supplementary Data 7 — Coordinates and Frequencies of all calculated Compounds [file ncomms9288-s8.doc]

# Atomic Coordinates and Frequencies of All Calculated Compounds

## C_6_H_5_F

Optimized atomic coordinates from the DFT calculations (BHLYP/SV(P) level) (Bohr Units):

| -2.28474555401362 1.09946356466841 0.00324586218163 C  -2.27099571358981 -1.52456555207549 -0.00479334716112 C  0.00000470951320 -2.84252781960176 -0.01246555596145 C  2.27101848606227 -1.52456263794245 -0.01208447228287 C  2.28478425378303 1.09946810946875 -0.00444198335175 C  0.00002535223705 2.36879563592773 0.00321894567004 C  0.00003536984371 4.87743474742093 0.01090989360524 F  -4.03009025506180 2.17300003233958 0.00952102107749 H  -4.05141777781358 -2.54286681157603 -0.00516285101760 H  -0.00000083832740 -4.89267059998886 -0.01919428476531 H  4.05143136558417 -2.54286255922266 -0.01804135204442 H  4.03013957439525 2.17300618047639 -0.00438009789967 H |
| --- |

List of calculated frequencies (BHLYP/SV(P) level):

| mode symmetry wave number IR intensity selection rules  cm**(-1) km/mol IR RAMAN |
| --- |
| 1 -0.00 0.00000 - -  2 -0.00 0.00000 - -  3 -0.00 0.00000 - -  4 0.00 0.00000 - -  5 0.00 0.00000 - -  6 0.00 0.00000 - -  7 a 255.47 0.15028 YES YES  8 a 428.26 2.53564 YES YES  9 a 446.57 0.00000 YES YES  10 a 541.17 6.38344 YES YES  11 a 543.88 4.02738 YES YES  12 a 648.14 0.10762 YES YES  13 a 734.89 17.97906 YES YES  14 a 812.70 62.68355 YES YES  15 a 861.98 22.80734 YES YES  16 a 883.17 0.00000 YES YES  17 a 971.53 9.86174 YES YES  18 a 1038.58 0.00000 YES YES  19 a 1054.90 0.08547 YES YES  20 a 1064.16 0.31806 YES YES  21 a 1078.97 3.54710 YES YES  22 a 1124.75 7.81543 YES YES  23 a 1202.22 0.25573 YES YES  24 a 1206.81 5.43082 YES YES  25 a 1341.55 91.43304 YES YES  26 a 1365.28 0.08159 YES YES  27 a 1393.38 1.07133 YES YES  28 a 1552.72 1.44396 YES YES  29 a 1605.13 97.39917 YES YES  30 a 1737.91 11.57386 YES YES  31 a 1746.58 63.47337 YES YES  32 a 3277.87 0.08495 YES YES  33 a 3287.55 6.85118 YES YES  34 a 3298.64 12.58320 YES YES  35 a 3307.29 3.61224 YES YES  36 a 3309.46 0.29883 YES YES |

Optimized atomic coordinates from the DFT calculations (B3LYP/SV(P) level) (Bohr Units):

| -2.30271735566905 0.34779699333752 -0.00228399886848 C  -2.28825070085603 -2.29538930858678 -0.00145872022100 C  -0.00005529415971 -3.62248783977189 -0.00062579635010 C  2.28820283477224 -2.29544462706630 -0.00131366219773 C  2.30270720069033 0.34778355500497 -0.00165182253085 C  -0.00001997134608 1.62795469693506 -0.00152270129010 C  0.00003078975037 4.16397055548000 -0.00030928594212 F  -4.06272990857513 1.42985127330025 -0.00285471587195 H  -4.08371146443332 -3.32220017396205 -0.00138152805580 H  -0.00002931457663 -5.68980392706074 0.00019322035044 H  4.08369947421484 -3.32226639600120 -0.00134712611895 H  4.06278145171794 1.42980939521514 -0.00204089509948 H |
| --- |

List of calculated frequencies (B3LYP/SV(P) level):

| mode symmetry wave number IR intensity selection rules  cm**(-1) km/mol IR RAMAN |
| --- |
| 1 -0.00 0.00000 - -  2 0.00 0.00000 - -  3 0.00 0.00000 - -  4 0.00 0.00000 - -  5 0.00 0.00000 - -  6 0.00 0.00000 - -  7 a 244.30 0.10048 YES YES  8 a 412.72 2.07749 YES YES  9 a 429.16 0.00002 YES YES  10 a 519.69 6.12206 YES YES  11 a 525.25 3.94534 YES YES  12 a 626.42 0.11418 YES YES  13 a 707.21 16.10693 YES YES  14 a 778.27 54.82389 YES YES  15 a 830.29 21.27351 YES YES  16 a 843.08 0.00004 YES YES  17 a 926.68 7.94842 YES YES  18 a 988.33 0.00000 YES YES  19 a 1015.29 0.26455 YES YES  20 a 1017.53 0.01261 YES YES  21 a 1040.98 3.04477 YES YES  22 a 1087.09 7.72786 YES YES  23 a 1166.65 6.26156 YES YES  24 a 1167.33 0.21438 YES YES  25 a 1283.29 89.28750 YES YES  26 a 1324.63 0.38671 YES YES  27 a 1371.58 0.33538 YES YES  28 a 1493.37 0.84615 YES YES  29 a 1539.70 83.23606 YES YES  30 a 1659.98 9.95301 YES YES  31 a 1666.10 54.36740 YES YES  32 a 3170.91 0.16546 YES YES  33 a 3180.04 8.79133 YES YES  34 a 3191.90 16.62908 YES YES  35 a 3201.26 5.47257 YES YES  36 a 3203.19 0.08139 YES YES |

### bipy

Optimized atomic coordinates from the DFT calculations (BHLYP/SV(P) level) (Bohr Units):

| -2.18254270410604 5.47145139325816 0.61218788150177 C  -2.24419205898079 2.85298579256121 0.64377074399313 C  -0.06752964449437 1.51346053344718 -0.01213974595367 C  2.05733404505777 2.67802542621588 -0.65932714229051 N  2.10659976644584 5.16955267423865 -0.69049318237791 C  0.04141362395625 6.67155026957937 -0.07343945459955 C  -0.03701037057136 -1.30232171826797 -0.00227768380716 C  -2.18420267563822 -2.68904436480461 -0.65713828884185 C  -2.06632729481231 -5.30553083061407 -0.62233746066367 C  0.18271712812862 -6.45674576219507 0.06532538719587 C  2.21521247097897 -4.91001445579222 0.68038443979436 C  2.11244172468481 -2.42017917988624 0.64608427973884 N  -3.84588247667383 6.55462474001386 1.13135203346130 H  -3.94487642817395 1.86420367701919 1.21380877058101 H  3.88851805869840 6.03795478742132 -1.24000081475841 H  0.18551966022409 8.71562129315837 -0.12519769434370 H  -3.90575875726828 -1.73765286664065 -1.22808655896861 H  -3.70597315874338 -6.42486109317269 -1.14016553189794 H  0.37058400182136 -8.49720094528998 0.12000410517223 H  4.01533883709027 -5.73930597688156 1.23091639100241 H |
| --- |

List of calculated frequencies (BHLYP/SV(P) level):

| mode symmetry wave number IR intensity selection rules  cm**(-1) km/mol IR RAMAN |
| --- |
| 1 -0.00 0.00000 - -  2 0.00 0.00000 - -  3 0.00 0.00000 - -  4 0.00 0.00000 - -  5 0.00 0.00000 - -  6 0.00 0.00000 - -  7 a 55.13 0.06590 YES YES  8 a 98.99 3.30511 YES YES  9 a 138.80 2.11389 YES YES  10 a 264.86 0.19557 YES YES  11 a 335.61 0.00063 YES YES  12 a 397.86 1.14975 YES YES  13 a 442.51 5.36454 YES YES  14 a 444.19 0.49451 YES YES  15 a 521.96 1.55388 YES YES  16 a 596.52 0.70878 YES YES  17 a 641.46 5.51346 YES YES  18 a 659.67 5.52403 YES YES  19 a 695.53 11.87554 YES YES  20 a 797.19 4.74103 YES YES  21 a 802.58 29.08347 YES YES  22 a 811.46 56.64152 YES YES  23 a 815.47 0.13494 YES YES  24 a 882.78 2.65946 YES YES  25 a 955.09 0.67684 YES YES  26 a 961.13 0.45153 YES YES  27 a 1036.76 0.05665 YES YES  28 a 1040.78 0.19627 YES YES  29 a 1052.27 4.45777 YES YES  30 a 1054.31 7.73385 YES YES  31 a 1072.55 0.21928 YES YES  32 a 1073.09 0.18231 YES YES  33 a 1095.73 4.23626 YES YES  34 a 1114.94 5.39480 YES YES  35 a 1139.79 0.31458 YES YES  36 a 1153.80 2.72927 YES YES  37 a 1169.15 6.52021 YES YES  38 a 1196.63 7.42950 YES YES  39 a 1204.81 3.59128 YES YES  40 a 1333.69 0.13880 YES YES  41 a 1342.90 6.36803 YES YES  42 a 1355.50 0.00122 YES YES  43 a 1382.25 4.02437 YES YES  44 a 1395.92 0.00002 YES YES  45 a 1519.41 8.06934 YES YES  46 a 1531.41 63.84934 YES YES  47 a 1572.32 31.70422 YES YES  48 a 1599.29 4.17446 YES YES  49 a 1703.96 55.01626 YES YES  50 a 1719.53 5.76065 YES YES  51 a 1731.93 101.93574 YES YES  52 a 1733.94 0.42883 YES YES  53 a 3257.48 24.46298 YES YES  54 a 3257.90 23.12865 YES YES  55 a 3280.01 4.10139 YES YES  56 a 3281.15 10.74625 YES YES  57 a 3298.03 16.54872 YES YES  58 a 3301.61 2.42617 YES YES  59 a 3304.93 11.93163 YES YES  60 a 3308.52 4.16066 YES YES |

Optimized atomic coordinates from the DFT calculations (B3LYP/SV(P) level) (Bohr Units):

| -2.20321545528318 5.51330072301401 0.59225061771527 C  -2.26884096730295 2.87594301523799 0.61206113039351 C  -0.06843428873446 1.52089039734401 -0.02133102351557 C  2.09089473276099 2.69484660456032 -0.63956628646808 N  2.13719210738397 5.20896277004181 -0.65803379844418 C  0.04913983687593 6.72228466332332 -0.05993230277041 C  -0.03703521894825 -1.30938169759642 -0.01349689700753 C  -2.20829084054678 -2.71480310859106 -0.63756650169952 C  -2.08803884724887 -5.34995862818245 -0.59574499117512 C  0.18886282873330 -6.50666243965131 0.06626313386626 C  2.24653647541925 -4.94563335459081 0.64655262503400 C  2.14780575340802 -2.43327524546739 0.60809768663671 N  -3.88664585035235 6.60528233376751 1.09659284969165 H  -3.99536620125558 1.88410135146181 1.16176207531972 H  3.94373963601556 6.08729066323961 -1.18247535336083 H  0.19686708531722 8.78410931573996 -0.09982181118436 H  -3.95527824331481 -1.76326754668351 -1.19375391010108 H  -3.74877756790041 -6.48092007222553 -1.08926190208969 H  0.37833074230364 -8.56460899086313 0.12631592972299 H  4.07193803029349 -5.78192736051081 1.17431920337408 H |
| --- |

List of calculated frequencies (B3LYP/SV(P) level):

| mode symmetry wave number IR intensity selection rules  cm**(-1) km/mol IR RAMAN |
| --- |
| 1 0.00 0.00000 - -  2 0.00 0.00000 - -  3 0.00 0.00000 - -  4 0.00 0.00000 - -  5 0.00 0.00000 - -  6 0.00 0.00000 - -  7 a 52.73 0.05044 YES YES  8 a 93.41 2.95703 YES YES  9 a 134.23 1.83735 YES YES  10 a 248.90 0.25876 YES YES  11 a 324.55 0.00049 YES YES  12 a 382.41 1.31158 YES YES  13 a 422.48 4.63060 YES YES  14 a 423.21 0.43659 YES YES  15 a 497.70 1.14876 YES YES  16 a 573.25 0.58374 YES YES  17 a 619.40 5.23362 YES YES  18 a 637.34 4.94824 YES YES  19 a 671.32 11.44559 YES YES  20 a 763.29 4.09667 YES YES  21 a 768.65 33.43815 YES YES  22 a 779.73 41.19998 YES YES  23 a 785.49 0.19473 YES YES  24 a 848.16 1.96426 YES YES  25 a 915.17 0.45317 YES YES  26 a 919.95 0.34061 YES YES  27 a 986.18 0.06351 YES YES  28 a 990.16 0.20961 YES YES  29 a 1009.56 3.64170 YES YES  30 a 1010.82 7.13187 YES YES  31 a 1023.27 0.24460 YES YES  32 a 1023.90 0.07970 YES YES  33 a 1056.01 4.74071 YES YES  34 a 1074.50 5.03892 YES YES  35 a 1097.62 0.32365 YES YES  36 a 1112.95 2.98968 YES YES  37 a 1126.54 7.94389 YES YES  38 a 1162.16 3.27933 YES YES  39 a 1171.16 2.86949 YES YES  40 a 1289.15 0.14167 YES YES  41 a 1317.99 0.11886 YES YES  42 a 1324.00 1.55073 YES YES  43 a 1332.13 3.11097 YES YES  44 a 1351.44 0.02699 YES YES  45 a 1457.44 6.33750 YES YES  46 a 1470.60 52.88142 YES YES  47 a 1505.53 28.31240 YES YES  48 a 1523.07 4.59056 YES YES  49 a 1624.21 42.05277 YES YES  50 a 1634.56 5.06615 YES YES  51 a 1645.89 0.49961 YES YES  52 a 1649.41 82.91378 YES YES  53 a 3138.94 29.19850 YES YES  54 a 3139.43 31.09688 YES YES  55 a 3171.31 4.13483 YES YES  56 a 3172.58 10.29479 YES YES  57 a 3187.70 22.56689 YES YES  58 a 3191.25 2.65067 YES YES  59 a 3195.02 16.27669 YES YES  60 a 3198.82 8.31857 YES YES |

### [Ga(C_6_H_5_F)_2_]^+^

Optimized atomic coordinates from the DFT calculations (BHLYP/SV(P) level) (Bohr Units):

| 20.20224916502128 0.68749756869832 20.12494766348030 Ga  18.66799963645974 5.46123195687578 23.27660928923859 C  18.87641503423385 3.55549883989128 25.07006990016131 C  17.19853806150899 1.52943145131163 24.97462235035002 C  15.35722608913694 1.40768150247559 23.09266304196895 C  15.18966118992898 3.33736184491769 21.30501641664977 C  16.85515329625001 5.37383582998858 21.38014754938671 C  24.80275865334465 -0.95826315993120 16.77644761680963 C  25.03857707685133 -2.43069150858575 18.93704752167677 C  23.17466494188968 -4.21009413372908 19.47374203032205 C  21.09494389856367 -4.48888858600168 17.87852172459255 C  20.89147832340309 -2.98235261102008 15.72526178520084 C  22.74228370802523 -1.19608346618940 15.16731317927845 C  20.23356175488939 7.38350501084656 23.36663854576988 F  20.29937973796127 3.71079221575976 26.53705224409669 H  17.31360510026474 0.05722758881103 26.39736735721662 H  14.02630479292337 -0.15059748771449 23.05342469105687 H  13.73647709607783 3.27644262730924 19.85963474838901 H  16.73886348904869 6.91395540836231 20.03279201976329 H  26.56312353825381 0.70808391937883 16.24981000238547 F  26.69119732687596 -2.20528648687410 20.12823680779336 H  23.36765400932311 -5.40379710565458 21.12981221505703 H  19.67247926852913 -5.90902421358769 18.27969348591081 H  19.30037164796452 -3.21657034728659 14.45318376000470 H  22.64437950068473 -0.02919797261045 13.48513088598721 H |
| --- |

List of calculated frequencies (BHLYP/SV(P) level):

| mode symmetry wave number IR intensity selection rules  cm**(-1) km/mol IR RAMAN |
| --- |
| 1 -0.00 0.00000 - -  2 -0.00 0.00000 - -  3 -0.00 0.00000 - -  4 -0.00 0.00000 - -  5 0.00 0.00000 - -  6 0.00 0.00000 - -  7 a 5.32 0.34609 YES YES  8 a 6.88 0.36499 YES YES  9 a 12.46 0.00618 YES YES  10 a 64.48 0.00900 YES YES  11 a 68.22 4.75598 YES YES  12 a 100.89 2.24686 YES YES  13 a 103.15 0.09015 YES YES  14 a 104.59 9.19758 YES YES  15 a 105.05 80.96032 YES YES  16 a 255.93 0.00053 YES YES  17 a 258.41 0.47709 YES YES  18 a 426.52 0.08975 YES YES  19 a 427.06 2.93515 YES YES  20 a 432.77 0.05072 YES YES  21 a 433.18 3.36286 YES YES  22 a 534.95 14.76672 YES YES  23 a 535.66 1.62998 YES YES  24 a 543.71 6.02415 YES YES  25 a 543.89 5.95516 YES YES  26 a 641.18 0.00146 YES YES  27 a 641.53 0.13777 YES YES  28 a 715.80 18.28257 YES YES  29 a 715.99 2.92336 YES YES  30 a 835.03 223.72903 YES YES  31 a 837.12 1.78919 YES YES  32 a 864.51 3.44564 YES YES  33 a 864.90 33.29486 YES YES  34 a 914.07 0.38076 YES YES  35 a 914.62 0.01086 YES YES  36 a 984.70 16.19939 YES YES  37 a 985.32 0.03093 YES YES  38 a 1049.05 7.76657 YES YES  39 a 1049.87 0.25717 YES YES  40 a 1054.98 0.00605 YES YES  41 a 1055.93 0.03005 YES YES  42 a 1071.20 2.98124 YES YES  43 a 1071.45 1.72484 YES YES  44 a 1073.81 2.49178 YES YES  45 a 1074.32 0.61892 YES YES  46 a 1122.74 7.87350 YES YES  47 a 1122.88 0.16000 YES YES  48 a 1207.37 0.01452 YES YES  49 a 1207.95 1.03620 YES YES  50 a 1208.89 1.88564 YES YES  51 a 1209.72 7.30148 YES YES  52 a 1360.25 48.63149 YES YES  53 a 1363.44 127.17298 YES YES  54 a 1369.10 0.00058 YES YES  55 a 1369.30 0.03075 YES YES  56 a 1404.16 0.03088 YES YES  57 a 1404.48 1.10192 YES YES  58 a 1542.21 17.57932 YES YES  59 a 1543.15 0.08644 YES YES  60 a 1599.33 18.74057 YES YES  61 a 1599.98 239.56776 YES YES  62 a 1708.41 0.26300 YES YES  63 a 1709.48 18.17717 YES YES  64 a 1726.56 16.21208 YES YES  65 a 1728.36 143.92198 YES YES  66 a 3293.84 0.02179 YES YES  67 a 3293.91 0.00977 YES YES  68 a 3301.37 0.06486 YES YES  69 a 3301.45 0.49133 YES YES  70 a 3309.26 0.31044 YES YES  71 a 3309.35 1.65840 YES YES  72 a 3314.42 2.96324 YES YES  73 a 3314.51 12.79707 YES YES  74 a 3316.90 0.49717 YES YES  75 a 3316.99 0.03219 YES YES |

Optimized atomic coordinates from the DFT calculations (B3LYP/SV(P) level) (Bohr Units):

| 20.17227284673306 0.64365463021257 20.14361036274461 Ga  18.65182589784256 5.51439930919977 23.17615049597569 C  18.95986311568418 3.65149823044442 25.03132459579784 C  17.33102045208836 1.55922252890007 25.02936549715588 C  15.43919793912103 1.32763548019705 23.17981649368019 C  15.17339369590539 3.21194255545343 21.32712038111394 C  16.78816341329548 5.31439062127444 21.30734175246174 c  24.83881742919839 -0.95233041094912 16.88732245047704 C  24.98101978380521 -2.50919791860062 19.02348526108166 C  23.05054766097855 -4.27540607974091 19.45372379356703 C  20.99797587399862 -4.46107577632196 17.77845741173342 C  20.88869533320547 -2.87486705049283 15.65104658274345 C  22.80617697203084 -1.10054037727609 15.19772002182023 C  20.16365679094383 7.51210706202105 23.18507985807365 F  20.42161449386205 3.89202960682722 26.47175705210520 H  17.52441389147774 0.12091609808733 26.50173745808465 H  14.14506752683182 -0.28282759954757 23.21534724693809 H  13.68067132367035 3.06428638002235 19.90434533723080 H  16.59483866340539 6.82224340222577 19.90750047252208 H  26.66533467541787 0.70921188427759 16.46105964293062 F  26.61443294729218 -2.35438192483244 20.27912736463307 H  23.17070343414186 -5.53236052181458 21.09052000595442 H  19.52221070614553 -5.87167325341096 18.09813168598665 H  19.31764138909610 -3.03629625169261 14.31691059851289 H  22.77979008124220 0.12911806097799 13.53718500922221 H |
| --- |

List of calculated frequencies (B3LYP/SV(P) level):

| mode symmetry wave number IR intensity selection rules  cm**(-1) km/mol IR RAMAN |
| --- |
| 1 0.00 0.00000 - -  2 0.00 0.00000 - -  3 0.00 0.00000 - -  4 0.00 0.00000 - -  5 0.00 0.00000 - -  6 0.00 0.00000 - -  7 a 7.95 0.21765 YES YES  8 a 8.98 0.23771 YES YES  9 a 9.79 0.01517 YES YES  10 a 63.12 0.02197 YES YES  11 a 66.41 4.26605 YES YES  12 a 97.85 0.02345 YES YES  13 a 101.84 0.24322 YES YES  14 a 103.04 1.66825 YES YES  15 a 109.97 89.04623 YES YES  16 a 242.94 0.03293 YES YES  17 a 246.30 0.40406 YES YES  18 a 408.14 0.03287 YES YES  19 a 409.15 0.97362 YES YES  20 a 417.13 1.56437 YES YES  21 a 417.79 2.89056 YES YES  22 a 513.20 14.14570 YES YES  23 a 514.50 3.61457 YES YES  24 a 524.51 4.53299 YES YES  25 a 524.97 4.67532 YES YES  26 a 619.69 0.00277 YES YES  27 a 620.10 0.16594 YES YES  28 a 687.75 11.48360 YES YES  29 a 688.59 7.89321 YES YES  30 a 802.41 201.90446 YES YES  31 a 804.34 1.86073 YES YES  32 a 833.03 2.88709 YES YES  33 a 833.42 28.65758 YES YES  34 a 875.21 0.26203 YES YES  35 a 876.48 0.13565 YES YES  36 a 942.30 12.26822 YES YES  37 a 942.97 0.20558 YES YES  38 a 1006.67 0.01194 YES YES  39 a 1007.85 0.03838 YES YES  40 a 1011.15 7.47160 YES YES  41 a 1012.08 0.96679 YES YES  42 a 1025.30 0.22800 YES YES  43 a 1026.59 0.16049 YES YES  44 a 1033.94 3.51414 YES YES  45 a 1034.12 3.43057 YES YES  46 a 1084.50 6.08499 YES YES  47 a 1084.75 2.17098 YES YES  48 a 1168.62 2.44994 YES YES  49 a 1169.59 8.11026 YES YES  50 a 1171.26 0.04245 YES YES  51 a 1172.03 0.81441 YES YES  52 a 1304.56 51.32991 YES YES  53 a 1308.02 124.28365 YES YES  54 a 1323.82 0.05248 YES YES  55 a 1324.23 0.10557 YES YES  56 a 1377.53 0.16542 YES YES  57 a 1378.44 0.38674 YES YES  58 a 1482.69 14.29551 YES YES  59 a 1483.70 0.11364 YES YES  60 a 1533.87 49.31911 YES YES  61 a 1534.64 178.93493 YES YES  62 a 1630.73 0.84545 YES YES  63 a 1632.16 15.26203 YES YES  64 a 1647.22 18.38688 YES YES  65 a 1649.30 126.12948 YES YES  66 a 3189.69 0.01900 YES YES  67 a 3190.06 0.01657 YES YES  68 a 3196.62 0.27223 YES YES  69 a 3197.15 0.34143 YES YES  70 a 3205.27 0.38657 YES YES  71 a 3205.55 0.54016 YES YES  72 a 3210.15 4.95937 YES YES  73 a 3210.65 5.83853 YES YES  74 a 3212.53 0.47547 YES YES  75 a 3213.06 0.69808 YES YES |

### [Ga(C_6_H_5_F)_2_]^+^ (triplet)

Optimized atomic coordinates from the DFT calculations (BHLYP/SV(P) level) (Bohr Units):

| 20.72958930132388 0.69334643943949 20.35549182612639 Ga  17.34643183921428 5.92914702094761 23.90792279649113 C  17.84583376689484 3.89127218296436 25.48958236234161 C  17.37855892425333 1.47311297363933 24.61219921338854 C  16.45833503457069 1.09191903284349 22.14252087481615 C  15.92429913510372 3.20020259400616 20.60788106098838 C  16.39239124857403 5.62199907345160 21.47956209645269 C  25.01217750480404 -2.06495695491705 15.85237109835920 C  25.53347597932084 -3.66523707625841 17.97409856409614 C  23.62360140325903 -4.27723678589175 19.59422243644778 C  21.14221879311527 -3.29789647853180 19.25260698733227 C  20.62115025555678 -1.63981648171198 17.06727180325061 C  22.68771245475754 -1.11603132223006 15.36041787705842 C  17.77924827886451 8.21156600941871 24.73553120618963 F  18.55717610039525 4.23832187422645 27.37897980082255 H  17.69751666582156 -0.13492408826848 25.84410818902572 H  15.84530036714710 -0.78051170688594 21.55914745715566 H  15.10870335467823 2.93176160502989 18.74555313507636 H  16.00185504445805 7.27735109214269 20.33776033910580 H  26.90509861754520 -1.58147171337966 14.33081467188008 F  27.43248947494896 -4.39673460671759 18.20405235674389 H  23.97726075313564 -5.55167508543644 21.16111652440060 H  19.57746486612628 -4.24726958302605 20.18732419055554 H  18.71458670877219 -1.59847505606034 16.29624991803835 H  22.38687046477301 0.01393572664733 13.67840004640332 H |
| --- |

List of calculated frequencies (BHLYP/SV(P) level):

| mode symmetry wave number IR intensity selection rules  cm**(-1) km/mol IR RAMAN |
| --- |
| 1 -0.00 0.00000 - -  2 -0.00 0.00000 - -  3 -0.00 0.00000 - -  4 -0.00 0.00000 - -  5 0.00 0.00000 - -  6 0.00 0.00000 - -  7 a 11.05 0.24071 YES YES  8 a 28.52 0.68764 YES YES  9 a 32.19 0.25410 YES YES  10 a 58.20 0.10868 YES YES  11 a 62.61 0.25546 YES YES  12 a 90.21 2.00099 YES YES  13 a 109.96 0.20442 YES YES  14 a 163.47 39.06417 YES YES  15 a 218.16 2.03226 YES YES  16 a 259.98 36.22421 YES YES  17 a 299.37 13.72713 YES YES  18 a 425.99 3.51152 YES YES  19 a 434.64 4.21584 YES YES  20 a 436.63 0.94541 YES YES  21 a 450.12 4.61656 YES YES  22 a 463.23 8.54278 YES YES  23 a 513.15 3.88701 YES YES  24 a 530.54 25.63900 YES YES  25 a 543.64 5.14675 YES YES  26 a 608.84 5.97553 YES YES  27 a 637.13 0.60460 YES YES  28 a 662.94 3.75843 YES YES  29 a 723.18 3.25076 YES YES  30 a 746.62 57.28210 YES YES  31 a 817.24 34.00966 YES YES  32 a 850.56 28.31636 YES YES  33 a 852.14 164.99978 YES YES  34 a 865.87 5.04681 YES YES  35 a 871.51 40.66309 YES YES  36 a 897.31 0.09163 YES YES  37 a 929.29 22.14664 YES YES  38 a 935.49 9.93292 YES YES  39 a 968.93 19.01991 YES YES  40 a 1017.92 1.01602 YES YES  41 a 1033.54 43.85253 YES YES  42 a 1038.82 1.08267 YES YES  43 a 1054.58 0.02458 YES YES  44 a 1058.35 11.27748 YES YES  45 a 1063.66 5.21695 YES YES  46 a 1080.22 3.27551 YES YES  47 a 1121.31 2.45988 YES YES  48 a 1161.18 1.57080 YES YES  49 a 1195.97 5.37658 YES YES  50 a 1200.81 1.11841 YES YES  51 a 1209.90 26.09775 YES YES  52 a 1334.46 92.54158 YES YES  53 a 1357.09 28.61203 YES YES  54 a 1368.50 6.00097 YES YES  55 a 1377.66 137.34408 YES YES  56 a 1415.26 4.42711 YES YES  57 a 1423.88 0.54949 YES YES  58 a 1448.41 10.77726 YES YES  59 a 1533.29 55.92625 YES YES  60 a 1544.62 14.53905 YES YES  61 a 1591.39 101.95975 YES YES  62 a 1601.57 20.85981 YES YES  63 a 1689.38 17.05095 YES YES  64 a 1709.91 304.33331 YES YES  65 a 1723.85 275.43327 YES YES  66 a 3243.12 1.73489 YES YES  67 a 3262.63 0.93308 YES YES  68 a 3266.84 2.85451 YES YES  69 a 3302.03 0.40734 YES YES  70 a 3302.46 2.42181 YES YES  71 a 3303.29 0.88303 YES YES  72 a 3315.12 5.30197 YES YES  73 a 3320.52 10.03400 YES YES  74 a 3321.08 3.27867 YES YES  75 a 3322.80 5.12125 YES YES |

### [In(C_6_H_5_F)_2_]^+^

Optimized atomic coordinates from the DFT calculations (BHLYP/SV(P) level) (Bohr Units):

| 28.76867080125745 10.62359217852608 35.20102695298937 C  24.85856755105130 10.62363098590088 23.26345326413851 C  28.04215701073413 8.21435170643194 34.45412304288700 C  25.58541838342987 8.21454251338426 24.01055754482771 C  29.45472091522335 6.97564172267935 32.60854936640069 C  24.17314908538362 6.97587205599154 25.85638283236571 C  31.54125731938908 8.15204093588183 31.51142263124431 C  22.08662005546684 8.15218878124753 26.95362075424426 C  32.22580161182860 10.57750182552445 32.28232813294669 C  21.40209417737265 10.57773254578947 26.18296647651136 C  30.83551927452020 11.83267398471987 34.12872280576192 C  22.79208983470722 11.83286495804360 24.33632516465828 C  27.45596538212985 11.79188002396226 36.95662084635173 F  26.16999123346772 11.79124134906194 21.50648989103694 F  26.81392865392341 11.26319489239194 29.23242305254081 In  26.44246331459055 7.31996307471653 35.37219125451038 H  27.18481068815606 7.32006877197157 23.09204181826371 H  28.94274833240336 5.06715682378707 32.05914174803324 H  24.68500749808594 5.06732404135751 26.40568320637940 H  32.66420687610599 7.16290631747402 30.11049060243498 H  20.96369130537415 7.16293175289387 28.35448059658024 H  33.87230653687033 11.48820372487780 31.46715298327787 H  19.75565565892253 11.48840416409922 26.99830976176214 H  31.35701775433710 13.70226968447558 34.78728935694295 H  22.27033312597348 13.70234438106706 23.67762496433195 H |
| --- |

List of calculated frequencies (BHLYP/SV(P) level):

| mode symmetry wave number IR intensity selection rules  cm**(-1) km/mol IR RAMAN |
| --- |
| 1 -0.00 0.00000 - -  2 0.00 0.00000 - -  3 0.00 0.00000 - -  4 0.00 0.00000 - -  5 0.00 0.00000 - -  6 0.00 0.00000 - -  7 a 7.74 0.85476 YES YES  8 a 10.14 1.07873 YES YES  9 a 14.07 0.06723 YES YES  10 a 54.24 0.43011 YES YES  11 a 66.98 2.53461 YES YES  12 a 84.60 0.14706 YES YES  13 a 87.80 26.80783 YES YES  14 a 92.60 16.17690 YES YES  15 a 98.77 0.91920 YES YES  16 a 256.87 0.36589 YES YES  17 a 258.04 0.00005 YES YES  18 a 428.85 2.02887 YES YES  19 a 428.91 3.08169 YES YES  20 a 436.39 0.30126 YES YES  21 a 436.75 0.66544 YES YES  22 a 538.44 15.13189 YES YES  23 a 538.83 0.10939 YES YES  24 a 544.77 9.06702 YES YES  25 a 545.12 4.59923 YES YES  26 a 642.71 0.02694 YES YES  27 a 642.92 0.19972 YES YES  28 a 725.14 21.56209 YES YES  29 a 725.67 3.97531 YES YES  30 a 832.73 206.22640 YES YES  31 a 836.75 28.44844 YES YES  32 a 864.16 25.58765 YES YES  33 a 864.50 10.67782 YES YES  34 a 911.89 0.67868 YES YES  35 a 912.22 1.28253 YES YES  36 a 985.31 1.54242 YES YES  37 a 985.83 17.78478 YES YES  38 a 1048.64 6.73562 YES YES  39 a 1049.63 0.00880 YES YES  40 a 1053.67 1.87480 YES YES  41 a 1055.64 0.23697 YES YES  42 a 1071.72 6.11937 YES YES  43 a 1072.23 0.10573 YES YES  44 a 1074.73 1.73307 YES YES  45 a 1075.33 0.00324 YES YES  46 a 1122.44 3.62606 YES YES  47 a 1122.65 3.97746 YES YES  48 a 1205.94 0.12010 YES YES  49 a 1207.13 0.23157 YES YES  50 a 1209.21 2.41380 YES YES  51 a 1209.65 7.09425 YES YES  52 a 1357.76 161.32301 YES YES  53 a 1359.53 18.99222 YES YES  54 a 1367.39 0.00055 YES YES  55 a 1367.74 0.06387 YES YES  56 a 1400.07 0.38988 YES YES  57 a 1400.53 0.85991 YES YES  58 a 1542.97 10.99247 YES YES  59 a 1543.11 6.62685 YES YES  60 a 1599.28 209.82841 YES YES  61 a 1599.44 48.19227 YES YES  62 a 1711.86 6.77674 YES YES  63 a 1712.29 10.80432 YES YES  64 a 1729.35 24.09777 YES YES  65 a 1729.42 127.74954 YES YES  66 a 3291.34 0.01820 YES YES  67 a 3291.53 0.07446 YES YES  68 a 3299.75 0.31433 YES YES  69 a 3299.90 0.56084 YES YES  70 a 3307.68 0.02844 YES YES  71 a 3307.76 1.18012 YES YES  72 a 3312.57 4.01984 YES YES  73 a 3312.72 5.94456 YES YES  74 a 3315.95 0.57237 YES YES  75 a 3316.03 2.02188 YES YES |

Optimized atomic coordinates from the DFT calculations (B3LYP/SV(P) level) (Bohr Units):

| 28.76884883740351 10.71263323293745 35.21320497192445 C  24.85877145968401 10.71265592816425 23.25161341266315 C  28.10373232924389 8.22379797545568 34.60584921127634 C  25.52387841808697 8.22382754676285 23.85900397430853 C  29.55796415487450 6.90952110294989 32.81752360694240 C  24.06957173687979 6.90953752234721 25.64726213745234 C  31.62471290900420 8.08615259287200 31.63759942810541 C  22.00275879360916 8.08615223676313 26.82708955022632 C  32.24645986749312 10.59010968973465 32.26526046697607 C  21.38102382845314 10.59010676664163 26.19939717721662 C  30.81487539253553 11.92304865549201 34.05262001626443 C  22.81268314583285 11.92305796470678 24.41210597835696 C  27.41748940837585 11.95659358457860 36.92366571305285 F  26.21016844453732 11.95661063534703 21.54118252052790 F  26.81368271276177 10.92552951489689 29.23245186075119 In  26.51936728036362 7.33214604683558 35.58823508984813 H  27.10827653470252 7.33217691309898 22.87667243901141 H  29.09537271275221 4.94222083712348 32.37976613028209 H  24.53216723122092 4.94224011752036 26.08502599740029 H  32.78199131572262 7.03879242916320 30.28293563849842 H  20.84541872378649 7.03878282474172 28.18169474968823 H  33.87866378753257 11.50472711541616 31.38522243403114 H  19.74876297180656 11.50470605712527 27.07935111240506 H  31.28997801706700 13.85769995510084 34.60192327786667 H  22.33757236697488 13.85769595048144 23.86276215634597 H |
| --- |

List of calculated frequencies (B3LYP/SV(P) level):

| mode symmetry wave number IR intensity selection rules  cm**(-1) km/mol IR RAMAN |
| --- |
| 1 -0.00 0.00000 - -  2 -0.00 0.00000 - -  3 -0.00 0.00000 - -  4 0.00 0.00000 - -  5 0.00 0.00000 - -  6 0.00 0.00000 - -  7 a 6.79 0.61495 YES YES  8 a 8.70 0.88994 YES YES  9 a 13.13 0.01999 YES YES  10 a 53.54 0.26582 YES YES  11 a 65.48 1.60783 YES YES  12 a 82.12 0.06245 YES YES  13 a 85.70 12.51383 YES YES  14 a 93.45 35.19847 YES YES  15 a 97.21 0.48494 YES YES  16 a 244.36 0.53805 YES YES  17 a 245.83 0.00003 YES YES  18 a 412.29 1.16846 YES YES  19 a 412.43 2.08234 YES YES  20 a 418.04 0.65487 YES YES  21 a 418.29 1.26531 YES YES  22 a 517.34 18.19531 YES YES  23 a 517.86 0.16861 YES YES  24 a 525.22 6.29428 YES YES  25 a 525.30 3.55377 YES YES  26 a 620.89 0.02805 YES YES  27 a 621.07 0.19608 YES YES  28 a 697.87 20.94277 YES YES  29 a 698.31 2.69880 YES YES  30 a 800.50 193.57980 YES YES  31 a 804.03 18.47043 YES YES  32 a 831.95 22.79378 YES YES  33 a 832.21 9.44184 YES YES  34 a 873.85 0.72395 YES YES  35 a 873.88 1.01253 YES YES  36 a 942.93 0.81410 YES YES  37 a 943.23 14.85606 YES YES  38 a 1005.22 0.33588 YES YES  39 a 1006.85 0.15995 YES YES  40 a 1011.86 9.29835 YES YES  41 a 1012.61 0.01608 YES YES  42 a 1027.83 0.24376 YES YES  43 a 1028.34 0.01428 YES YES  44 a 1034.09 6.92401 YES YES  45 a 1034.45 0.13293 YES YES  46 a 1084.60 3.41861 YES YES  47 a 1084.76 4.25169 YES YES  48 a 1168.84 1.08411 YES YES  49 a 1169.94 8.47771 YES YES  50 a 1172.15 1.75530 YES YES  51 a 1172.26 0.23282 YES YES  52 a 1299.32 159.37881 YES YES  53 a 1301.17 23.10850 YES YES  54 a 1324.34 0.00811 YES YES  55 a 1324.43 0.07533 YES YES  56 a 1373.23 0.20354 YES YES  57 a 1373.59 0.33786 YES YES  58 a 1483.36 9.52727 YES YES  59 a 1483.57 5.41112 YES YES  60 a 1533.20 178.71861 YES YES  61 a 1533.43 48.31286 YES YES  62 a 1633.37 5.35329 YES YES  63 a 1633.86 10.03181 YES YES  64 a 1648.35 24.43821 YES YES  65 a 1648.51 108.37237 YES YES  66 a 3187.16 0.00119 YES YES  67 a 3187.27 0.07802 YES YES  68 a 3194.87 0.24741 YES YES  69 a 3194.98 0.57194 YES YES  70 a 3203.55 0.00421 YES YES  71 a 3203.60 0.41465 YES YES  72 a 3208.81 3.23595 YES YES  73 a 3208.88 4.62214 YES YES  74 a 3211.25 0.21293 YES YES  75 a 3211.31 1.30642 YES YES |

### [Ga(bipy)]^+^

Optimized atomic coordinates from the DFT calculations (BHLYP/SV(P) level) (Bohr Units):

| 1.48326973523205 1.58498324010621 -4.23949934740332 Ga  -4.02250308081362 3.53052675727889 -3.85602319557121 C  -6.09653909014327 5.11679977070260 -4.09373448222022 C  -5.78069657634831 7.46729529333766 -5.22007399167946 C  -3.41127488592331 8.17824574992144 -6.07936405082761 C  -1.43382294992147 6.48962649139951 -5.77291784889199 C  -1.74408765655391 4.24526688388887 -4.69604133228209 N  -4.12883016912425 0.98502679195533 -2.69312431286521 C  -1.93454403453160 -0.27614727043223 -2.64029160781935 N  -1.81483012508400 -2.57531445420073 -1.64449675960321 C  -3.91174177016685 -3.76944710785769 -0.62776820388049 C  -6.19575817652251 -2.48927397925795 -0.66620494909823 C  -6.31209165936398 -0.08272146285407 -1.70968950443893 C  -7.94148020421043 4.54936059839206 -3.41955491262942 H  -7.38376794756828 8.72930492503125 -5.42111140378922 H  -3.09003681285732 9.99526668804137 -6.96747274664585 H  0.46200774141493 6.95207835518578 -6.41195161088608 H  0.02152567478132 -3.49399414586366 -1.66061148221713 H  -3.74418235756018 -5.64853708057749 0.16874223289512 H  -7.88659943328140 -3.35050259469934 0.10983511425041 H  -8.08921365642227 0.92767541959247 -1.74564294989888 H |
| --- |

List of calculated frequencies (BHLYP/SV(P) level):

| mode symmetry wave number IR intensity selection rules  cm**(-1) km/mol IR RAMAN |
| --- |
| 1 -0.00 0.00000 - -  2 -0.00 0.00000 - -  3 -0.00 0.00000 - -  4 0.00 0.00000 - -  5 0.00 0.00000 - -  6 0.00 0.00000 - -  7 a 71.56 0.00021 YES YES  8 a 73.41 0.22351 YES YES  9 a 121.02 2.59965 YES YES  10 a 155.13 5.29105 YES YES  11 a 160.10 17.50990 YES YES  12 a 247.46 0.05280 YES YES  13 a 253.66 32.21158 YES YES  14 a 366.62 3.67490 YES YES  15 a 439.51 0.00045 YES YES  16 a 440.84 2.94239 YES YES  17 a 471.19 1.57834 YES YES  18 a 473.82 5.16615 YES YES  19 a 588.35 0.00007 YES YES  20 a 666.87 20.68351 YES YES  21 a 684.90 33.45041 YES YES  22 a 688.33 3.83145 YES YES  23 a 789.98 14.83786 YES YES  24 a 799.16 0.00018 YES YES  25 a 810.67 4.76528 YES YES  26 a 820.57 91.16328 YES YES  27 a 883.31 0.00059 YES YES  28 a 966.05 0.00016 YES YES  29 a 966.53 0.39415 YES YES  30 a 1052.27 0.16622 YES YES  31 a 1053.04 0.00005 YES YES  32 a 1069.86 42.28215 YES YES  33 a 1077.27 74.53150 YES YES  34 a 1092.48 0.00014 YES YES  35 a 1094.97 0.50203 YES YES  36 a 1095.58 0.11636 YES YES  37 a 1125.28 5.39113 YES YES  38 a 1134.67 8.85213 YES YES  39 a 1166.32 11.64654 YES YES  40 a 1181.32 0.34377 YES YES  41 a 1209.07 16.23956 YES YES  42 a 1224.63 8.02707 YES YES  43 a 1343.34 29.33960 YES YES  44 a 1349.13 0.34475 YES YES  45 a 1366.33 0.02964 YES YES  46 a 1390.13 41.84848 YES YES  47 a 1403.20 3.93166 YES YES  48 a 1524.89 1.58612 YES YES  49 a 1541.13 87.25689 YES YES  50 a 1571.66 33.27766 YES YES  51 a 1607.80 45.17189 YES YES  52 a 1705.32 44.02142 YES YES  53 a 1716.67 14.69165 YES YES  54 a 1729.99 62.86571 YES YES  55 a 1741.34 55.45568 YES YES  56 a 3290.52 0.88622 YES YES  57 a 3290.74 5.47160 YES YES  58 a 3306.53 0.43771 YES YES  59 a 3307.48 0.27122 YES YES  60 a 3323.02 0.36144 YES YES  61 a 3325.07 0.00346 YES YES  62 a 3326.85 4.14751 YES YES  63 a 3337.97 0.82146 YES YES |

Optimized atomic coordinates from the DFT calculations (B3LYP/SV(P) level) (Bohr Units):

| 1.54964101738182 1.57444907842444 -4.25602208733586 Ga  -4.01479979911825 3.53390875366970 -3.86179066645802 C  -6.10839765241110 5.13325609506194 -4.10389430332751 C  -5.80032374553862 7.50211623493471 -5.23399683630456 C  -3.41487466785231 8.22733131402492 -6.09793786140646 C  -1.41532797161991 6.53433899192402 -5.79289966538472 C  -1.71650091377057 4.26635904952475 -4.71023728793332 N  -4.12160035300842 0.97788742206176 -2.69426063468503 C  -1.90906104052068 -0.30281611756836 -2.63345519920500 N  -1.80031722006299 -2.62147005807324 -1.62418772586368 C  -3.91899801203552 -3.81472929505568 -0.60189618126203 C  -6.21745449202251 -2.51968456020545 -0.64669588198007 C  -6.32496187397869 -0.09945458925086 -1.70238482536680 C  -7.96840888064550 4.55724866317459 -3.42611098586275 H  -7.42152429577120 8.76860720594088 -5.43479902293314 H  -3.09735429160519 10.06194941522040 -6.99089991517133 H  0.49491872783459 7.00777235983448 -6.43711835632015 H  0.04950735521496 -3.55224295077941 -1.63635885658666 H  -3.75715335016999 -5.70844837347337 0.20527829544684 H  -7.92597923815823 -3.38151296014603 0.13469390152298 H  -8.11622673711026 0.92065318984625 -1.74202324908524 H |
| --- |

List of calculated frequencies (B3LYP/SV(P) level):

| mode symmetry wave number IR intensity selection rules  cm**(-1) km/mol IR RAMAN |
| --- |
| 1 0.00 0.00000 - -  2 0.00 0.00000 - -  3 0.00 0.00000 - -  4 0.00 0.00000 - -  5 0.00 0.00000 - -  6 0.00 0.00000 - -  7 a 68.58 0.00692 YES YES  8 a 68.77 0.13917 YES YES  9 a 115.38 2.69812 YES YES  10 a 149.89 4.28842 YES YES  11 a 156.01 14.86020 YES YES  12 a 234.46 0.02328 YES YES  13 a 244.08 27.65236 YES YES  14 a 353.10 3.51668 YES YES  15 a 420.19 0.00057 YES YES  16 a 421.22 3.04272 YES YES  17 a 451.28 1.26109 YES YES  18 a 457.77 3.51703 YES YES  19 a 567.76 0.00006 YES YES  20 a 644.61 17.25270 YES YES  21 a 662.06 28.50009 YES YES  22 a 664.67 3.74258 YES YES  23 a 760.21 7.12158 YES YES  24 a 764.86 0.00034 YES YES  25 a 781.55 4.45931 YES YES  26 a 786.23 88.86010 YES YES  27 a 850.43 0.00040 YES YES  28 a 925.77 0.00008 YES YES  29 a 925.89 0.17669 YES YES  30 a 1001.67 0.21842 YES YES  31 a 1002.80 0.00006 YES YES  32 a 1027.81 33.92453 YES YES  33 a 1034.90 63.37539 YES YES  34 a 1044.45 0.00013 YES YES  35 a 1047.08 0.17330 YES YES  36 a 1055.95 0.13364 YES YES  37 a 1084.64 3.55439 YES YES  38 a 1093.27 6.46605 YES YES  39 a 1125.31 12.12063 YES YES  40 a 1140.46 0.12346 YES YES  41 a 1175.15 7.40914 YES YES  42 a 1188.69 4.97612 YES YES  43 a 1301.03 0.57100 YES YES  44 a 1318.81 1.91127 YES YES  45 a 1323.14 1.20376 YES YES  46 a 1346.63 40.57711 YES YES  47 a 1363.74 2.02254 YES YES  48 a 1464.19 0.87578 YES YES  49 a 1480.78 75.91822 YES YES  50 a 1507.34 34.53754 YES YES  51 a 1535.24 36.06282 YES YES  52 a 1625.45 18.59598 YES YES  53 a 1635.38 8.59794 YES YES  54 a 1647.19 60.37368 YES YES  55 a 1659.30 39.40007 YES YES  56 a 3181.76 0.99947 YES YES  57 a 3182.05 6.15554 YES YES  58 a 3201.01 0.58399 YES YES  59 a 3202.00 0.10005 YES YES  60 a 3216.37 0.00950 YES YES  61 a 3218.79 0.01176 YES YES  62 a 3220.36 2.59145 YES YES  63 a 3229.01 0.09066 YES YES |

### [Ga(bipy)]^+^ (triplet)

Optimized atomic coordinates from the DFT calculations (BHLYP/SV(P) level) (Bohr Units):

| 0.78512249115123 1.66318616218381 -4.13206757635125 Ga  -4.02591522325600 3.49002425856048 -3.84153471501763 C  -6.08469221726206 5.13898577963919 -4.10256935912323 C  -5.75751715365814 7.45949801586976 -5.21308530027995 C  -3.34158675096706 8.17167945121182 -6.08834585348152 C  -1.38235356400487 6.52903779789482 -5.80743538499333 C  -1.69314534459310 4.22550692079292 -4.70765049277988 N  -4.13006499928603 1.02206475371765 -2.71536783644750 C  -1.88296984796883 -0.27223790255159 -2.65130237847550 N  -1.76670252726836 -2.62542248150290 -1.62395010626465 C  -3.84364934629650 -3.77548450776616 -0.63304352845429 C  -6.17592146544840 -2.48465294561794 -0.67778735963527 C  -6.30662280613307 -0.10984562698316 -1.71102341208633 C  -7.92982013367678 4.57486597956443 -3.42499690347220 H  -7.34664803532106 8.73647655298749 -5.41581417306572 H  -3.02588008212841 9.99066784145784 -6.97279231535341 H  0.50997736741751 6.98557302107688 -6.44779756309625 H  0.06887181259376 -3.53654980797997 -1.63643508393002 H  -3.68263489795113 -5.65137838862850 0.17054203017810 H  -7.85626121377485 -3.36209724964612 0.09938098476297 H  -8.08678349713652 0.89562124480949 -1.75392101813551 H |
| --- |

List of calculated frequencies (BHLYP/SV(P) level):

| mode symmetry wave number IR intensity selection rules  cm**(-1) km/mol IR RAMAN |
| --- |
| 1 -0.00 0.00000 - -  2 -0.00 0.00000 - -  3 -0.00 0.00000 - -  4 0.00 0.00000 - -  5 0.00 0.00000 - -  6 0.00 0.00000 - -  7 a 99.76 0.66630 YES YES  8 a 111.09 0.00002 YES YES  9 a 164.52 6.23182 YES YES  10 a 210.56 0.03681 YES YES  11 a 250.10 0.00004 YES YES  12 a 285.80 0.37611 YES YES  13 a 325.24 5.33441 YES YES  14 a 409.15 0.47960 YES YES  15 a 436.20 0.01127 YES YES  16 a 445.77 19.12481 YES YES  17 a 462.01 0.91900 YES YES  18 a 481.00 14.52925 YES YES  19 a 576.27 0.00006 YES YES  20 a 665.10 14.03714 YES YES  21 a 685.09 4.87209 YES YES  22 a 708.29 13.15163 YES YES  23 a 734.70 20.43519 YES YES  24 a 784.89 0.00767 YES YES  25 a 797.58 0.93867 YES YES  26 a 803.81 91.58384 YES YES  27 a 838.14 0.00191 YES YES  28 a 922.12 0.52579 YES YES  29 a 923.50 0.02262 YES YES  30 a 1011.31 137.98841 YES YES  31 a 1030.58 0.00260 YES YES  32 a 1031.32 0.03192 YES YES  33 a 1061.10 0.00276 YES YES  34 a 1065.19 0.40511 YES YES  35 a 1077.39 1.17058 YES YES  36 a 1086.80 0.02631 YES YES  37 a 1095.10 1.94989 YES YES  38 a 1096.72 3.42447 YES YES  39 a 1176.85 1.68344 YES YES  40 a 1199.74 0.26002 YES YES  41 a 1214.51 26.26923 YES YES  42 a 1221.91 13.13065 YES YES  43 a 1308.91 8.58018 YES YES  44 a 1345.10 3.26448 YES YES  45 a 1379.18 26.14011 YES YES  46 a 1394.72 0.00054 YES YES  47 a 1422.89 12.19185 YES YES  48 a 1518.24 0.55389 YES YES  49 a 1524.49 0.26804 YES YES  50 a 1546.19 133.57428 YES YES  51 a 1551.09 167.85262 YES YES  52 a 1632.59 4.48773 YES YES  53 a 1642.51 239.30321 YES YES  54 a 1708.56 73.92121 YES YES  55 a 1716.25 75.59841 YES YES  56 a 3307.03 0.80061 YES YES  57 a 3307.88 2.04171 YES YES  58 a 3311.91 9.54818 YES YES  59 a 3313.66 0.89074 YES YES  60 a 3323.29 0.01913 YES YES  61 a 3328.13 0.29696 YES YES  62 a 3329.35 6.40796 YES YES  63 a 3336.31 1.01572 YES YES |

### [In(bipy)]^+^

Optimized atomic coordinates from the DFT calculations (BHLYP/SV(P) level) (Bohr Units):

| 14.60434477053291 -5.61882840638651 15.61763795142356 In  14.83491077154608 -1.37561418122782 17.31947878617999 N  17.51205710862791 -2.94236936779524 13.30710254601519 N  13.46285108862363 -0.74965780954215 19.32455089616998 C  16.36858344397014 0.32750758892471 16.24759198494817 C  17.83266541836917 -0.53313791979660 14.01030445211035 C  18.75878498132476 -3.85424703321208 11.33007008637313 C  13.53606820871711 1.63566684221465 20.39987257249657 C  16.54875700964473 2.76154422674806 17.21793636331647 C  19.46239746610646 1.04128517367852 12.68225934326632 C  20.41778969016343 -2.40723490996744 9.91395655828839 C  15.11706287965852 3.41821616149490 19.31543498990199 C  20.76592857733592 0.09117679461751 10.61378247953843 C  12.25239887405203 -2.21159621390131 20.11037868178132 H  18.42186686889145 -5.82338234179747 10.85162035904861 H  12.38558306172639 2.06909429202033 22.03763534800706 H  17.78674139100756 4.14799048953981 16.36854732330268 H  19.72841769144329 2.99144614180815 13.23219446497450 H  21.39865771872267 -3.22513781593117 8.31311470556639 H  15.24374818906279 5.31219167358814 20.08969377560960 H  22.04228107851329 1.30314973505148 9.56271673440551 H |
| --- |

List of calculated frequencies (BHLYP/SV(P) level):

| mode symmetry wave number IR intensity selection rules  cm**(-1) km/mol IR RAMAN |
| --- |
| 1 -0.00 0.00000 - -  2 -0.00 0.00000 - -  3 0.00 0.00000 - -  4 0.00 0.00000 - -  5 0.00 0.00000 - -  6 0.00 0.00000 - -  7 a 56.87 0.00198 YES YES  8 a 65.69 0.01670 YES YES  9 a 118.32 2.86077 YES YES  10 a 134.71 2.45308 YES YES  11 a 143.82 16.49280 YES YES  12 a 230.75 17.33993 YES YES  13 a 244.75 0.16199 YES YES  14 a 363.30 3.16552 YES YES  15 a 438.31 0.01032 YES YES  16 a 442.16 2.72477 YES YES  17 a 467.91 3.32618 YES YES  18 a 472.55 2.11797 YES YES  19 a 590.16 0.00163 YES YES  20 a 664.03 18.02639 YES YES  21 a 681.96 28.99775 YES YES  22 a 689.18 3.16573 YES YES  23 a 793.48 11.84854 YES YES  24 a 796.12 0.00469 YES YES  25 a 809.49 4.90785 YES YES  26 a 818.82 90.38019 YES YES  27 a 885.68 0.00809 YES YES  28 a 964.16 0.00155 YES YES  29 a 966.00 0.51060 YES YES  30 a 1049.95 0.06289 YES YES  31 a 1050.93 0.00054 YES YES  32 a 1068.84 33.76323 YES YES  33 a 1074.65 67.91276 YES YES  34 a 1091.40 0.00157 YES YES  35 a 1094.20 0.46587 YES YES  36 a 1096.72 0.08472 YES YES  37 a 1128.24 3.30471 YES YES  38 a 1136.72 7.86535 YES YES  39 a 1167.14 10.47515 YES YES  40 a 1181.31 0.22875 YES YES  41 a 1209.31 14.74736 YES YES  42 a 1228.44 8.38821 YES YES  43 a 1342.04 28.98368 YES YES  44 a 1349.79 0.42401 YES YES  45 a 1366.43 0.03965 YES YES  46 a 1391.33 37.12428 YES YES  47 a 1395.79 3.96853 YES YES  48 a 1524.14 1.63580 YES YES  49 a 1538.90 86.76535 YES YES  50 a 1574.78 35.88106 YES YES  51 a 1606.62 37.84671 YES YES  52 a 1704.81 50.50971 YES YES  53 a 1717.79 12.11172 YES YES  54 a 1729.72 55.07380 YES YES  55 a 1740.71 49.95168 YES YES  56 a 3281.69 0.21955 YES YES  57 a 3281.95 7.16527 YES YES  58 a 3305.84 0.61651 YES YES  59 a 3306.67 0.04968 YES YES  60 a 3323.28 0.96610 YES YES  61 a 3324.07 0.06833 YES YES  62 a 3328.43 2.11882 YES YES  63 a 3343.81 0.39913 YES YES |

Optimized atomic coordinates from the DFT calculations (B3LYP/SV(P) level) (Bohr Units):

| 14.58844245156217 -5.63935988940931 15.61521003772041 In  14.81710396987473 -1.39254029510614 17.33945376170291 N  17.51161394109845 -2.97016271070615 13.29242126107694 N  13.43503073756840 -0.75543750475743 19.36331740590465 C  16.36484009815318 0.32742283788421 16.25194683176233 C  17.83480990986875 -0.53648162067669 14.00625791737192 C  18.77275489088894 -3.88532429745322 11.29635553145078 C  13.51054836750466 1.65034449087358 20.44042488868747 C  16.54033733837167 2.78257568992032 17.23207146465588 C  19.48156359904459 1.04711131386825 12.66479991819435 C  20.44450814266937 -2.42320098215930 9.87468552018339 C  15.10085633526420 3.44661368993302 19.34397275581280 C  20.79553024768115 0.09354460366526 10.58218839533410 C  12.21643886692359 -2.23049480949890 20.15782131861556 H  18.43191703470845 -5.87074646702097 10.81267606598704 H  12.35186041975038 2.08961257967397 22.09239766321373 H  17.78576024525848 4.18257688814799 16.37399848582887 H  19.75186589943889 3.01343715459091 13.21963662347239 H  21.43491826692226 -3.24576048303555 8.26006470515097 H  15.22786599557259 5.35766340268978 20.12138325890735 H  22.08332952991525 1.31666952870499 9.52479659169060 H |
| --- |

List of calculated frequencies (B3LYP/SV(P) level):

| mode symmetry wave number IR intensity selection rules  cm**(-1) km/mol IR RAMAN |
| --- |
| 1 -0.00 0.00000 - -  2 0.00 0.00000 - -  3 0.00 0.00000 - -  4 0.00 0.00000 - -  5 0.00 0.00000 - -  6 0.00 0.00000 - -  7 a 56.96 0.00144 YES YES  8 a 62.68 0.03145 YES YES  9 a 113.84 2.67254 YES YES  10 a 131.93 1.92012 YES YES  11 a 140.67 14.14425 YES YES  12 a 223.74 15.33869 YES YES  13 a 232.37 0.30529 YES YES  14 a 350.88 2.80238 YES YES  15 a 419.33 0.00756 YES YES  16 a 422.54 2.64234 YES YES  17 a 450.68 1.37188 YES YES  18 a 454.91 2.78122 YES YES  19 a 569.21 0.00114 YES YES  20 a 642.42 15.68793 YES YES  21 a 659.64 25.31325 YES YES  22 a 665.38 3.17262 YES YES  23 a 762.60 0.00372 YES YES  24 a 763.12 4.78591 YES YES  25 a 780.38 4.73867 YES YES  26 a 785.09 87.60914 YES YES  27 a 852.31 0.00543 YES YES  28 a 924.18 0.00111 YES YES  29 a 925.38 0.25834 YES YES  30 a 999.98 0.09545 YES YES  31 a 1001.27 0.00061 YES YES  32 a 1027.03 29.00238 YES YES  33 a 1032.52 59.48732 YES YES  34 a 1043.72 0.00095 YES YES  35 a 1046.62 0.16468 YES YES  36 a 1056.90 0.18842 YES YES  37 a 1087.20 2.53935 YES YES  38 a 1094.99 6.23104 YES YES  39 a 1126.08 11.31732 YES YES  40 a 1140.28 0.13358 YES YES  41 a 1175.51 6.61585 YES YES  42 a 1191.59 5.59971 YES YES  43 a 1302.59 0.55295 YES YES  44 a 1319.11 2.68152 YES YES  45 a 1321.59 2.29901 YES YES  46 a 1345.55 37.80140 YES YES  47 a 1357.03 1.59811 YES YES  48 a 1463.45 0.89785 YES YES  49 a 1478.74 75.71697 YES YES  50 a 1509.81 36.24446 YES YES  51 a 1533.73 32.07334 YES YES  52 a 1625.31 23.10180 YES YES  53 a 1635.96 7.14534 YES YES  54 a 1646.89 55.97846 YES YES  55 a 1658.94 36.15695 YES YES  56 a 3173.19 0.03274 YES YES  57 a 3173.50 8.46813 YES YES  58 a 3200.21 0.70615 YES YES  59 a 3201.04 0.00810 YES YES  60 a 3216.60 0.16902 YES YES  61 a 3217.58 0.04059 YES YES  62 a 3221.06 1.67137 YES YES  63 a 3233.32 0.00313 YES YES |

### [In(bipy)]^+^ (triplet)

Optimized atomic coordinates from the DFT calculations (BHLYP/SV(P) level) (Bohr Units):

| 14.89970994504973 -4.98267251500219 15.56492842025341 In  14.81063891884094 -1.44446932042879 17.35074766637859 N  17.47563670067167 -3.01037020499575 13.28819387075184 N  13.42289609198163 -0.76972254163586 19.39944456876438 C  16.37503656191234 0.27934611825250 16.22067164087487 C  17.79864565456065 -0.55748305471259 14.04698852027797 C  18.76246776543258 -3.90625923777285 11.25806555206421 C  13.51215565782114 1.59588126873912 20.41286005619491 C  16.51777196383169 2.73941079452056 17.21330718687620 C  19.47149948640709 1.00300025271606 12.69659713210652 C  20.39869213448164 -2.44995789467852 9.90456023402014 C  15.10833458365631 3.40257027999151 19.28470952768242 C  20.76225824136218 0.08020377201543 10.64737568711623 C  12.22013584649733 -2.22115215140212 20.20744467403048 H  18.42421056166508 -5.86499465589585 10.75318705162629 H  12.37083086290612 2.05103478180474 22.05034655018960 H  17.74559025811129 4.12090664390034 16.34175638615451 H  19.74477984163612 2.94428812714831 13.27321375383074 H  21.38549089445685 -3.24471316773419 8.29652579132046 H  15.22513628197682 5.30213456445322 20.04350097814035 H  22.04997803478102 1.29108126084540 9.61145515407004 H |
| --- |

List of calculated frequencies (BHLYP/SV(P) level):

| mode symmetry wave number IR intensity selection rules  cm**(-1) km/mol IR RAMAN |
| --- |
| 1 -0.00 0.00000 - -  2 -0.00 0.00000 - -  3 -0.00 0.00000 - -  4 -0.00 0.00000 - -  5 -0.00 0.00000 - -  6 0.00 0.00000 - -  7 a 93.77 0.10339 YES YES  8 a 97.21 0.00000 YES YES  9 a 146.94 6.76907 YES YES  10 a 193.39 0.48662 YES YES  11 a 220.98 0.20813 YES YES  12 a 245.51 0.00002 YES YES  13 a 262.53 0.14290 YES YES  14 a 393.44 0.08936 YES YES  15 a 435.69 0.00234 YES YES  16 a 445.90 15.72678 YES YES  17 a 461.03 2.22133 YES YES  18 a 470.61 11.71628 YES YES  19 a 577.69 0.00010 YES YES  20 a 656.41 11.97669 YES YES  21 a 680.11 0.22633 YES YES  22 a 693.40 8.39779 YES YES  23 a 742.01 15.48323 YES YES  24 a 781.38 0.00106 YES YES  25 a 793.59 2.58599 YES YES  26 a 802.98 93.50098 YES YES  27 a 843.85 0.00012 YES YES  28 a 922.48 0.00171 YES YES  29 a 923.62 0.33238 YES YES  30 a 1015.44 100.62837 YES YES  31 a 1030.40 0.00045 YES YES  32 a 1031.14 0.08414 YES YES  33 a 1047.95 76.08559 YES YES  34 a 1060.58 0.00020 YES YES  35 a 1065.55 0.30868 YES YES  36 a 1082.94 1.23193 YES YES  37 a 1100.73 3.93122 YES YES  38 a 1100.94 3.61361 YES YES  39 a 1171.85 0.65446 YES YES  40 a 1197.38 0.80985 YES YES  41 a 1214.36 29.29740 YES YES  42 a 1223.98 62.64401 YES YES  43 a 1313.83 30.38587 YES YES  44 a 1347.84 1.33484 YES YES  45 a 1360.45 143.20703 YES YES  46 a 1390.85 0.00705 YES YES  47 a 1415.88 65.20731 YES YES  48 a 1511.74 75.17230 YES YES  49 a 1523.08 5.37678 YES YES  50 a 1529.15 312.63682 YES YES  51 a 1553.87 147.22464 YES YES  52 a 1634.43 18.67342 YES YES  53 a 1647.45 265.77869 YES YES  54 a 1711.36 46.73196 YES YES  55 a 1712.80 58.70688 YES YES  56 a 3297.54 2.78066 YES YES  57 a 3297.73 1.49371 YES YES  58 a 3309.26 8.01051 YES YES  59 a 3310.85 0.02459 YES YES  60 a 3324.65 2.10692 YES YES  61 a 3326.07 2.23098 YES YES  62 a 3329.61 2.06013 YES YES  63 a 3345.08 0.38405 YES YES |

### [Ga(bipy)_2_]^+^

Optimized atomic coordinates from the DFT calculations (BHLYP/SV(P) level) (Bohr Units):

| 1.96823951425786 2.28969543781168 -3.41218410105485 Ga  -4.01518610591294 3.67561817964635 -4.00025706203177 C  -6.11071748943377 5.25375135314467 -3.77388509289306 C  -5.97171684646205 7.69380912147499 -4.71694965816557 C  -3.74841081884012 8.50435054740721 -5.84295815973832 C  -1.74392293579882 6.82240432468944 -5.95239344910170 C  -1.88511161065365 4.48303694693494 -5.07185079259927 N  -3.99253501563869 1.03832444620604 -3.04550657197808 C  -1.73747993619557 -0.08505454773122 -2.84039465461017 N  -1.60494852255624 -2.44942163073465 -2.01298263414498 C  -3.71381258806403 -3.84271362360998 -1.33394023701238 C  -6.06215854660441 -2.70101028750149 -1.53251248969920 C  -6.20336797656448 -0.23461032698431 -2.39842020726153 C  -7.81968202142405 4.61679996594065 -2.84916956781926 H  -7.58215085321101 8.95225143445328 -4.55540398209496 H  -3.55697168993604 10.39792458551034 -6.59993716136120 H  0.05482810852951 7.38484422803482 -6.77006414567087 H  0.27416335206598 -3.25771375407714 -1.89533012574630 H  -3.50789518814196 -5.77099704530473 -0.67516410552984 H  -7.77072570803277 -3.71753010317786 -1.03171582235702 H  -8.02530056275346 0.67080488422477 -2.59937927323646 H  5.47892387211874 -2.46981309733376 -5.19449053798443 C  7.71922193432204 -3.85036342151550 -5.28645538015463 C  8.93490781512734 -4.45034484685486 -3.04482812138442 C  7.90019411349235 -3.64725074339288 -0.77498567222799 C  5.68683696676191 -2.24979832304678 -0.86129296800456 C  4.51350604984957 -1.69602746359697 -3.00231921631301 N  4.05298598262854 -1.73987556870428 -7.49711971493702 C  2.29305527276192 0.05549701943765 -7.23388092385994 N  0.95298355840595 0.77456639653818 -9.22889510339568 C  1.28343967068626 -0.25121775881555 -11.61383054338845 C  3.10010296703299 -2.11333343064646 -11.91403050289557 C  4.49939899855431 -2.86608992618060 -9.83368865829216 C  8.53426005695668 -4.42677008875906 -7.07110264871921 H  10.68291880148286 -5.52117226043375 -3.07736084697662 H  8.79141382932274 -4.06997870060878 1.02010102632353 H  4.82973463625337 -1.53393662718890 0.86304499018420 H  -0.44736472773343 2.23257577219298 -8.89467684161794 H  0.13682223634581 0.39518202274737 -13.18293318003277 H  3.42169432416438 -2.98273187241150 -13.74270380302963 H  5.89975425372130 -4.34275664598882 -10.02895149061263 H |
| --- |

List of calculated frequencies (BHLYP/SV(P) level):

| mode symmetry wave number IR intensity selection rules  cm**(-1) km/mol IR RAMAN |
| --- |
| 1 -0.00 0.00000 - -  2 -0.00 0.00000 - -  3 0.00 0.00000 - -  4 0.00 0.00000 - -  5 0.00 0.00000 - -  6 0.00 0.00000 - -  7 a 25.40 0.00724 YES YES  8 a 27.52 4.33978 YES YES  9 a 29.11 1.17580 YES YES  10 a 69.57 0.07854 YES YES  11 a 74.24 0.11831 YES YES  12 a 76.27 2.71589 YES YES  13 a 84.37 0.86606 YES YES  14 a 89.64 0.09609 YES YES  15 a 109.85 16.94766 YES YES  16 a 117.74 10.88466 YES YES  17 a 118.32 0.37714 YES YES  18 a 128.20 5.10404 YES YES  19 a 186.14 24.43217 YES YES  20 a 207.67 4.50364 YES YES  21 a 211.41 20.21771 YES YES  22 a 260.51 3.38062 YES YES  23 a 263.11 1.45085 YES YES  24 a 354.71 1.15596 YES YES  25 a 354.94 1.15280 YES YES  26 a 436.30 5.54610 YES YES  27 a 437.08 2.04496 YES YES  28 a 443.92 2.99657 YES YES  29 a 449.96 8.17565 YES YES  30 a 451.37 0.03811 YES YES  31 a 466.31 11.00220 YES YES  32 a 493.19 2.95761 YES YES  33 a 493.68 1.35561 YES YES  34 a 591.10 0.34768 YES YES  35 a 593.46 0.07694 YES YES  36 a 651.93 16.23432 YES YES  37 a 655.10 5.55677 YES YES  38 a 671.35 10.07065 YES YES  39 a 674.62 27.01378 YES YES  40 a 690.27 2.41414 YES YES  41 a 691.86 5.07121 YES YES  42 a 796.78 7.38451 YES YES  43 a 797.36 0.70682 YES YES  44 a 798.43 1.71034 YES YES  45 a 799.75 29.68668 YES YES  46 a 811.45 2.57529 YES YES  47 a 811.62 6.17632 YES YES  48 a 816.06 68.26712 YES YES  49 a 816.60 64.03775 YES YES  50 a 883.04 1.61654 YES YES  51 a 884.05 0.13743 YES YES  52 a 960.95 1.84122 YES YES  53 a 961.01 0.51567 YES YES  54 a 963.62 0.56367 YES YES  55 a 963.80 0.23286 YES YES  56 a 1046.59 0.13259 YES YES  57 a 1046.83 0.12686 YES YES  58 a 1057.20 0.18748 YES YES  59 a 1057.56 0.17184 YES YES  60 a 1059.21 42.72820 YES YES  61 a 1061.56 0.48637 YES YES  62 a 1066.45 23.03695 YES YES  63 a 1066.87 50.31874 YES YES  64 a 1085.11 0.19320 YES YES  65 a 1085.25 0.17319 YES YES  66 a 1088.08 0.31931 YES YES  67 a 1088.25 0.23762 YES YES  68 a 1096.74 1.09110 YES YES  69 a 1097.71 0.24873 YES YES  70 a 1123.96 8.49762 YES YES  71 a 1124.21 0.65838 YES YES  72 a 1136.97 5.20905 YES YES  73 a 1137.43 3.58497 YES YES  74 a 1164.27 12.38785 YES YES  75 a 1164.80 2.18153 YES YES  76 a 1178.53 0.22250 YES YES  77 a 1180.17 0.57098 YES YES  78 a 1205.32 4.65421 YES YES  79 a 1205.68 22.02679 YES YES  80 a 1221.33 4.50574 YES YES  81 a 1221.39 8.75942 YES YES  82 a 1338.95 8.18097 YES YES  83 a 1339.16 11.51285 YES YES  84 a 1344.68 33.32540 YES YES  85 a 1347.59 1.45942 YES YES  86 a 1363.86 0.00356 YES YES  87 a 1364.04 0.04653 YES YES  88 a 1386.09 12.24477 YES YES  89 a 1387.17 33.24150 YES YES  90 a 1391.45 5.36899 YES YES  91 a 1392.47 0.54456 YES YES  92 a 1520.73 0.67670 YES YES  93 a 1521.44 5.79267 YES YES  94 a 1536.83 30.61717 YES YES  95 a 1537.38 110.52757 YES YES  96 a 1571.05 36.45606 YES YES  97 a 1574.25 15.36831 YES YES  98 a 1598.40 1.68232 YES YES  99 a 1600.37 20.57329 YES YES  100 a 1702.49 81.43317 YES YES  101 a 1703.05 6.31006 YES YES  102 a 1719.38 6.14122 YES YES  103 a 1720.57 14.74368 YES YES  104 a 1729.91 9.75794 YES YES  105 a 1729.98 62.98682 YES YES  106 a 1737.97 57.25097 YES YES  107 a 1739.18 43.95902 YES YES  108 a 3279.81 8.42044 YES YES  109 a 3279.83 2.37566 YES YES  110 a 3299.63 1.77305 YES YES  111 a 3299.66 1.87038 YES YES  112 a 3300.68 1.11105 YES YES  113 a 3300.73 0.29658 YES YES  114 a 3312.01 1.16393 YES YES  115 a 3312.21 1.22413 YES YES  116 a 3317.55 1.11206 YES YES  117 a 3317.61 0.98702 YES YES  118 a 3321.44 1.37354 YES YES  119 a 3321.54 1.32680 YES YES  120 a 3323.82 1.34263 YES YES  121 a 3324.00 1.45380 YES YES  122 a 3334.57 0.19400 YES YES  123 a 3334.70 0.17622 YES YES |

Optimized atomic coordinates from the DFT calculations (B3LYP/SV(P) level) (Bohr Units):

| 1.92865806304418 2.22917118465740 -3.45230310560553 Ga  -4.07380254322430 3.69241324491156 -3.96214576523927 C  -6.15839132604588 5.31796525644233 -3.70718146536346 C  -6.00743211307809 7.76664897962802 -4.67549679270404 C  -3.77826476100444 8.54759531419222 -5.85591555537155 C  -1.77978390437826 6.83057667562486 -5.99004311054681 C  -1.92867281546883 4.47546979632087 -5.08622815959832 N  -4.07387579729975 1.05009033573481 -2.98849592999950 C  -1.81185544825957 -0.12554900655436 -2.82960154246443 N  -1.70238339762942 -2.50799926380666 -1.98123012210924 C  -3.83509029781656 -3.86744538711414 -1.23778010155884 C  -6.18529243283310 -2.67628645283669 -1.38621697291564 C  -6.30477317710734 -0.19560700286953 -2.26833545371684 C  -7.87285068106886 4.70678317366415 -2.73782704800984 H  -7.61286728146008 9.05587111086741 -4.49207431124039 H  -3.57757780630745 10.44850545189068 -6.63859894939893 H  0.02637088964460 7.37077577231205 -6.85010002135744 H  0.18249524002697 -3.35264163503648 -1.90385199293890 H  -3.64717346252992 -5.81209617329435 -0.56797127581521 H  -7.91285351346441 -3.66805814111895 -0.83386961240254 H  -8.13200256268081 0.74551642114960 -2.42957867582979 H  5.54273864970686 -2.48138845020027 -5.23382033558214 C  7.82444993728301 -3.83870696364574 -5.30106386978336 C  9.02844739354198 -4.44588964359856 -3.03431356895379 C  7.94474909407878 -3.67036294715602 -0.75470207886356 C  5.69891742152327 -2.28978066590924 -0.85650229204945 C  4.52872443800317 -1.72736146001745 -3.02441728389606 N  4.13087755506053 -1.75834813153826 -7.56052034018260 C  2.29998476348076 0.00408724257453 -7.30343768923822 N  0.96030411623791 0.71418198244445 -9.33052960816526 C  1.35900292296794 -0.28409205991997 -11.73753234765243 C  3.24809382019437 -2.10282374923888 -12.03415754173811 C  4.64642681132631 -2.84510087193231 -9.92706639661048 C  8.68100041215011 -4.39341842991985 -7.09305903598340 H  10.80633587559105 -5.50037290527596 -3.05369595483071 H  8.82422401900278 -4.10323320276876 1.06339594278467 H  4.80355163095413 -1.59234825418010 0.87716616015397 H  -0.49509434301631 2.14291399802387 -8.99498255152899 H  0.20673067320107 0.34979660333473 -13.33003996546744 H  3.62518753772019 -2.94736929644902 -13.88286812720667 H  6.10269357081844 -4.29115787759629 -10.12380658244487 H |
| --- |

List of calculated frequencies (B3LYP/SV(P) level):

| mode symmetry wave number IR intensity selection rules  cm**(-1) km/mol IR RAMAN |
| --- |
| 1 0.00 0.00000 - -  2 0.00 0.00000 - -  3 0.00 0.00000 - -  4 0.00 0.00000 - -  5 0.00 0.00000 - -  6 0.00 0.00000 - -  7 a 24.03 0.02237 YES YES  8 a 28.81 2.40886 YES YES  9 a 29.01 1.92758 YES YES  10 a 67.50 0.07497 YES YES  11 a 70.07 0.86154 YES YES  12 a 73.29 1.61097 YES YES  13 a 79.48 0.61769 YES YES  14 a 87.37 0.02044 YES YES  15 a 106.75 6.07637 YES YES  16 a 110.89 0.09987 YES YES  17 a 115.27 17.60667 YES YES  18 a 127.75 4.42608 YES YES  19 a 179.63 18.98960 YES YES  20 a 199.47 1.78739 YES YES  21 a 204.03 17.41724 YES YES  22 a 246.57 4.20050 YES YES  23 a 249.41 1.29590 YES YES  24 a 342.65 0.63612 YES YES  25 a 342.74 0.52166 YES YES  26 a 418.52 6.65600 YES YES  27 a 419.13 4.66744 YES YES  28 a 424.39 1.65646 YES YES  29 a 431.67 0.10727 YES YES  30 a 432.31 6.68768 YES YES  31 a 447.73 9.37807 YES YES  32 a 472.95 2.93335 YES YES  33 a 473.17 0.09016 YES YES  34 a 569.58 0.25145 YES YES  35 a 571.74 0.08601 YES YES  36 a 630.11 12.98592 YES YES  37 a 632.85 4.89479 YES YES  38 a 648.33 6.32719 YES YES  39 a 651.65 19.25384 YES YES  40 a 666.27 2.13578 YES YES  41 a 667.70 4.67332 YES YES  42 a 762.97 0.85196 YES YES  43 a 764.33 1.16614 YES YES  44 a 765.59 2.85520 YES YES  45 a 767.84 15.28561 YES YES  46 a 780.51 43.26204 YES YES  47 a 780.87 31.12072 YES YES  48 a 783.16 34.99236 YES YES  49 a 783.81 29.67902 YES YES  50 a 849.07 0.92905 YES YES  51 a 849.96 0.06989 YES YES  52 a 919.76 0.53333 YES YES  53 a 919.87 1.45987 YES YES  54 a 923.06 0.63907 YES YES  55 a 923.33 0.12431 YES YES  56 a 995.97 0.04787 YES YES  57 a 996.24 0.02392 YES YES  58 a 1006.27 0.15384 YES YES  59 a 1006.47 0.06029 YES YES  60 a 1016.06 32.98105 YES YES  61 a 1017.81 0.17745 YES YES  62 a 1023.04 15.38652 YES YES  63 a 1023.66 31.07677 YES YES  64 a 1035.91 0.15046 YES YES  65 a 1036.07 0.08031 YES YES  66 a 1039.03 0.11382 YES YES  67 a 1039.20 0.24096 YES YES  68 a 1056.25 0.93638 YES YES  69 a 1057.40 0.36683 YES YES  70 a 1082.76 6.31439 YES YES  71 a 1083.03 0.41911 YES YES  72 a 1095.08 4.28848 YES YES  73 a 1095.16 2.99477 YES YES  74 a 1123.15 12.57332 YES YES  75 a 1123.47 1.56644 YES YES  76 a 1136.86 0.65054 YES YES  77 a 1138.57 1.84041 YES YES  78 a 1171.24 1.79761 YES YES  79 a 1171.41 10.75042 YES YES  80 a 1185.12 7.95073 YES YES  81 a 1185.26 0.23779 YES YES  82 a 1294.93 6.36976 YES YES  83 a 1297.85 0.01533 YES YES  84 a 1318.54 0.50903 YES YES  85 a 1319.21 7.03784 YES YES  86 a 1324.43 3.80729 YES YES  87 a 1325.34 0.00641 YES YES  88 a 1340.14 12.75406 YES YES  89 a 1340.75 32.49488 YES YES  90 a 1349.27 0.16923 YES YES  91 a 1349.58 1.22006 YES YES  92 a 1458.95 0.74097 YES YES  93 a 1460.05 5.90019 YES YES  94 a 1476.25 25.55699 YES YES  95 a 1476.68 90.24730 YES YES  96 a 1505.33 39.69363 YES YES  97 a 1508.45 15.65761 YES YES  98 a 1523.26 0.19489 YES YES  99 a 1525.40 8.78239 YES YES  100 a 1621.89 31.61469 YES YES  101 a 1622.60 1.80905 YES YES  102 a 1636.19 2.71954 YES YES  103 a 1637.24 8.26942 YES YES  104 a 1645.84 1.72821 YES YES  105 a 1645.93 61.95129 YES YES  106 a 1655.29 41.16367 YES YES  107 a 1656.21 30.89925 YES YES  108 a 3168.74 10.97202 YES YES  109 a 3168.80 2.88119 YES YES  110 a 3193.15 3.03834 YES YES  111 a 3193.16 2.12321 YES YES  112 a 3194.34 1.21010 YES YES  113 a 3194.38 0.42603 YES YES  114 a 3201.07 1.29209 YES YES  115 a 3201.17 1.34297 YES YES  116 a 3209.73 2.05840 YES YES  117 a 3209.77 2.79080 YES YES  118 a 3212.70 1.52351 YES YES  119 a 3212.76 0.72341 YES YES  120 a 3214.92 0.28989 YES YES  121 a 3214.96 0.39872 YES YES  122 a 3224.83 1.60977 YES YES  123 a 3224.90 1.00586 YES YES |

### [Ga(bipy)_2_]^+^ (triplet)

Optimized atomic coordinates from the DFT calculations (BHLYP/SV(P) level) (Bohr Units):

| 0.38635432909954 0.12707898362536 -4.92749241474237 Ga  -3.45852775656748 3.11859719286358 -3.19283492999206 C  -4.84188114228502 5.32722030394309 -2.62312084275338 C  -3.87384580979987 7.65039381404311 -3.19739723115765 C  -1.47003651676388 7.84241378693943 -4.36984654907145 C  -0.18772757850566 5.68010804803045 -4.89716331715239 C  -1.12765325737774 3.35925461299252 -4.33982039601494 N  -4.25068292465275 0.60162288226210 -2.68775482772568 C  -2.58320245377407 -1.27137113066000 -3.40626553246783 N  -3.16121267467490 -3.74128895662542 -3.02565367169018 C  -5.36616565707131 -4.48203349963632 -1.93575408395760 C  -7.11211511641018 -2.59585232343441 -1.17201654807729 C  -6.55557317633961 -0.10266705623491 -1.54409618244210 C  -6.67376341082994 5.16911092208065 -1.72730987887905 H  -4.94002551386866 9.34403605553836 -2.75746600204322 H  -0.65816165698999 9.66028075282592 -4.84259208026134 H  1.65678738313480 5.71669519763795 -5.79312496323716 H  -1.75810442405498 -5.10332926326392 -3.64408029605620 H  -5.76161726231473 -6.47138231204938 -1.66485308132372 H  -8.88493717634737 -3.13212772891106 -0.29552347825743 H  -7.88081537768108 1.34428044481555 -0.96676076733831 H  5.22257452056258 -1.51255176515064 -5.74098076839596 C  7.76708680907265 -2.13397455854558 -5.23567696894163 C  8.73138613638636 -1.82961605728118 -2.86051562858930 C  7.16806249412555 -0.88942526401776 -0.89532703037293 C  4.72557754293728 -0.31252540556674 -1.44234972952414 C  3.74262860284307 -0.60363092028036 -3.79393847844459 N  4.00254104722571 -1.72843777355483 -8.12481933222440 C  1.50275796414039 -0.98555736775583 -8.18378294853943 N  0.15853434953479 -1.11973369600612 -10.36417956804315 C  1.18439486210581 -1.97688584904744 -12.55766408706709 C  3.75057255185712 -2.75396246936286 -12.55132568057193 C  5.12681056147781 -2.62714261293235 -10.37094553463824 C  8.95100276727002 -2.84822446421902 -6.74280129894102 H  10.68763640483736 -2.30459219170704 -2.47821257614213 H  7.88502093583011 -0.63003778990473 1.00360578658454 H  3.44703421532230 0.41259154853241 -0.01202949456854 H  -1.79453120792012 -0.50232697814354 -10.26105018491493 H  0.05164124942672 -2.05734636847144 -14.25991353987371 H  4.61754111368801 -3.44781019990166 -14.27343885149140 H  7.08456142423644 -3.21892597167045 -10.35452644208457 H |
| --- |

List of calculated frequencies (BHLYP/SV(P) level):

| mode symmetry wave number IR intensity selection rules  cm**(-1) km/mol IR RAMAN |
| --- |
| 1 -0.00 0.00000 - -  2 0.00 0.00000 - -  3 0.00 0.00000 - -  4 0.00 0.00000 - -  5 0.00 0.00000 - -  6 0.00 0.00000 - -  7 a 22.62 0.00001 YES YES  8 a 37.82 0.30070 YES YES  9 a 37.99 0.31324 YES YES  10 a 85.84 0.18313 YES YES  11 a 86.13 0.29858 YES YES  12 a 113.93 0.00071 YES YES  13 a 117.66 0.00012 YES YES  14 a 153.77 1.07975 YES YES  15 a 154.15 1.08892 YES YES  16 a 154.37 2.49879 YES YES  17 a 220.47 0.00072 YES YES  18 a 227.59 0.00213 YES YES  19 a 243.37 0.00034 YES YES  20 a 270.28 0.00002 YES YES  21 a 286.51 0.07284 YES YES  22 a 291.44 0.18622 YES YES  23 a 367.25 68.02564 YES YES  24 a 404.69 0.00002 YES YES  25 a 426.77 3.56665 YES YES  26 a 427.83 0.49538 YES YES  27 a 428.14 2.35369 YES YES  28 a 428.73 30.08211 YES YES  29 a 437.75 0.00826 YES YES  30 a 447.69 9.04748 YES YES  31 a 447.91 6.93394 YES YES  32 a 493.21 23.69580 YES YES  33 a 494.54 24.42946 YES YES  34 a 565.30 0.00120 YES YES  35 a 566.13 0.00069 YES YES  36 a 661.17 21.65712 YES YES  37 a 661.56 23.11276 YES YES  38 a 688.53 3.76958 YES YES  39 a 688.98 4.28595 YES YES  40 a 704.71 0.00023 YES YES  41 a 717.02 47.62782 YES YES  42 a 718.32 7.97803 YES YES  43 a 718.38 9.64173 YES YES  44 a 775.69 0.00018 YES YES  45 a 776.32 0.00406 YES YES  46 a 795.06 86.62660 YES YES  47 a 795.08 78.43221 YES YES  48 a 795.51 49.44973 YES YES  49 a 798.08 0.00722 YES YES  50 a 820.95 0.02386 YES YES  51 a 821.83 0.00200 YES YES  52 a 906.14 1.31634 YES YES  53 a 906.22 1.53383 YES YES  54 a 907.02 0.00247 YES YES  55 a 907.18 0.00158 YES YES  56 a 1009.12 286.22058 YES YES  57 a 1010.72 299.62896 YES YES  58 a 1025.23 0.17054 YES YES  59 a 1025.25 0.24619 YES YES  60 a 1025.34 1.10291 YES YES  61 a 1025.35 0.88434 YES YES  62 a 1052.59 0.00015 YES YES  63 a 1052.61 0.00043 YES YES  64 a 1057.34 0.28905 YES YES  65 a 1057.37 0.28175 YES YES  66 a 1079.41 0.37565 YES YES  67 a 1079.77 0.32567 YES YES  68 a 1081.19 0.00047 YES YES  69 a 1081.30 2.28439 YES YES  70 a 1092.90 0.00132 YES YES  71 a 1094.66 4.64780 YES YES  72 a 1094.70 4.60774 YES YES  73 a 1096.42 113.55858 YES YES  74 a 1182.90 138.32809 YES YES  75 a 1184.30 0.00114 YES YES  76 a 1204.59 1.17577 YES YES  77 a 1204.66 1.05728 YES YES  78 a 1211.57 47.99811 YES YES  79 a 1211.64 51.64356 YES YES  80 a 1226.68 0.00085 YES YES  81 a 1226.83 5.34198 YES YES  82 a 1292.74 93.37771 YES YES  83 a 1302.34 0.00177 YES YES  84 a 1345.21 0.06311 YES YES  85 a 1345.36 0.12365 YES YES  86 a 1391.07 26.11212 YES YES  87 a 1391.44 0.08014 YES YES  88 a 1395.82 13.65619 YES YES  89 a 1395.91 14.59273 YES YES  90 a 1442.94 4.85281 YES YES  91 a 1443.86 0.00035 YES YES  92 a 1521.80 0.01059 YES YES  93 a 1521.96 26.13754 YES YES  94 a 1522.68 0.00670 YES YES  95 a 1522.80 0.32387 YES YES  96 a 1553.19 98.08134 YES YES  97 a 1553.24 104.15185 YES YES  98 a 1610.87 0.04373 YES YES  99 a 1610.93 0.13356 YES YES  100 a 1625.26 627.78921 YES YES  101 a 1626.23 668.78733 YES YES  102 a 1643.05 0.00798 YES YES  103 a 1645.01 7.90483 YES YES  104 a 1697.71 465.84680 YES YES  105 a 1701.10 0.02182 YES YES  106 a 1707.42 18.00674 YES YES  107 a 1707.44 17.97899 YES YES  108 a 3297.57 0.63449 YES YES  109 a 3297.61 1.48130 YES YES  110 a 3297.65 1.72914 YES YES  111 a 3297.95 0.00411 YES YES  112 a 3303.63 12.93259 YES YES  113 a 3303.66 13.43897 YES YES  114 a 3306.11 5.47760 YES YES  115 a 3306.17 0.09299 YES YES  116 a 3318.70 0.02459 YES YES  117 a 3318.72 0.02365 YES YES  118 a 3326.01 3.07315 YES YES  119 a 3326.06 0.10762 YES YES  120 a 3327.30 0.62925 YES YES  121 a 3327.32 0.65950 YES YES  122 a 3331.25 0.01858 YES YES  123 a 3331.27 0.00778 YES YES |

Optimized atomic coordinates from the DFT calculations (B3LYP/SV(P) level) (Bohr Units):

| 0.36390066982091 0.10116622405910 -4.95292803328326 Ga  -3.51871208017129 3.11038810893033 -3.18064631060508 C  -4.90729163217876 5.32846342275923 -2.61093660245688 C  -3.96094304304127 7.67604972943066 -3.23703206364345 C  -1.57171280674564 7.86808824225089 -4.46423809801686 C  -0.26587074160435 5.68960222232834 -4.99166133703677 C  -1.18384874165073 3.35514968101549 -4.37943532953636 N  -4.29164202850989 0.57003129003730 -2.63559124324212 C  -2.60618129334774 -1.31627414422115 -3.38016602396516 N  -3.15621382635583 -3.80363011908804 -2.95471113385124 C  -5.35136303583000 -4.55285964696160 -1.79359328078777 C  -7.10662907391752 -2.66689645345194 -1.00959452267702 C  -6.57250250480681 -0.14982302131152 -1.43095393077189 C  -6.73674760180805 5.16895107159163 -1.67170291609635 H  -5.04044716149810 9.38058634347697 -2.79348398339952 H  -0.77527744358039 9.69932885120513 -4.98376699000611 H  1.57648238735949 5.72623709148305 -5.93031459832290 H  -1.74241428558797 -5.17099192383592 -3.59363822397110 H  -5.72284095943492 -6.55927218832864 -1.48702863994684 H  -8.86858902430570 -3.21083516515584 -0.07845224053525 H  -7.90941297627464 1.30368366756972 -0.83551942073801 H  5.26032890813602 -1.52875909084634 -5.75343417109572 C  7.80290679550464 -2.17639137662252 -5.21639142136858 C  8.73362533358872 -1.97035599420157 -2.78945726161793 C  7.12544042636234 -1.10044703693136 -0.81252954898455 C  4.67186129037394 -0.48382638426836 -1.38585185686662 C  3.73017843518016 -0.67934523937154 -3.78213491071077 N  4.06754431539246 -1.66207316927316 -8.18401312753364 C  1.53613447769970 -0.92662181166272 -8.25050735898769 N  0.21613609023164 -0.98191863235284 -10.46973692748040 C  1.29687621068998 -1.74789714153687 -12.69984910863015 C  3.88051008575225 -2.50753920868529 -12.68915843092191 C  5.23175585150086 -2.46063469181197 -10.45970852582858 C  9.02506913757073 -2.84266145135120 -6.73852615399265 H  10.69624330161931 -2.47314246185786 -2.38599993298410 H  7.80825584440429 -0.91941476802223 1.12618040229003 H  3.35534519215177 0.19477914532354 0.05737010108535 H  -1.75930426202123 -0.37832422477859 -10.36945758528247 H  0.17947291940480 -1.76608505744939 -14.43440721297381 H  4.78864086671859 -3.12516829601333 -14.43878099055716 H  7.21116315409323 -3.04039182027392 -10.43901048609417 H |
| --- |

List of calculated frequencies (B3LYP/SV(P) level):

| mode symmetry wave number IR intensity selection rules  cm**(-1) km/mol IR RAMAN |
| --- |
| 1 -0.00 0.00000 - -  2 -0.00 0.00000 - -  3 -0.00 0.00000 - -  4 -0.00 0.00000 - -  5 -0.00 0.00000 - -  6 0.00 0.00000 - -  7 a 22.10 0.00000 YES YES  8 a 36.94 0.13382 YES YES  9 a 37.03 0.13434 YES YES  10 a 82.96 0.21922 YES YES  11 a 83.03 0.23713 YES YES  12 a 108.10 0.00018 YES YES  13 a 111.95 0.00046 YES YES  14 a 146.87 2.68456 YES YES  15 a 147.02 3.11110 YES YES  16 a 148.03 0.50983 YES YES  17 a 213.16 0.02920 YES YES  18 a 222.34 0.00040 YES YES  19 a 236.60 0.00004 YES YES  20 a 260.76 0.00028 YES YES  21 a 278.97 0.65931 YES YES  22 a 279.89 0.63280 YES YES  23 a 354.82 66.57687 YES YES  24 a 389.61 0.00002 YES YES  25 a 407.16 0.01859 YES YES  26 a 410.18 3.94337 YES YES  27 a 410.24 3.94310 YES YES  28 a 412.03 32.79080 YES YES  29 a 416.64 0.00105 YES YES  30 a 431.50 6.73496 YES YES  31 a 431.54 6.50420 YES YES  32 a 477.87 10.44697 YES YES  33 a 478.11 10.62659 YES YES  34 a 546.56 0.00037 YES YES  35 a 547.09 0.00016 YES YES  36 a 642.32 5.85072 YES YES  37 a 642.38 5.95098 YES YES  38 a 665.72 1.09867 YES YES  39 a 665.81 1.13714 YES YES  40 a 679.56 0.00155 YES YES  41 a 691.27 29.40018 YES YES  42 a 691.51 6.43624 YES YES  43 a 691.61 17.13777 YES YES  44 a 741.81 0.00129 YES YES  45 a 742.03 0.00362 YES YES  46 a 760.23 78.66984 YES YES  47 a 760.23 77.12107 YES YES  48 a 767.84 44.73400 YES YES  49 a 770.08 0.01534 YES YES  50 a 789.51 0.00110 YES YES  51 a 790.15 0.00124 YES YES  52 a 869.48 1.05580 YES YES  53 a 869.52 1.05580 YES YES  54 a 870.93 0.02584 YES YES  55 a 871.16 0.00828 YES YES  56 a 970.35 0.62285 YES YES  57 a 970.37 0.58303 YES YES  58 a 970.63 0.02439 YES YES  59 a 970.74 0.00787 YES YES  60 a 989.51 107.85414 YES YES  61 a 989.73 108.94901 YES YES  62 a 1002.03 0.00038 YES YES  63 a 1002.06 0.00042 YES YES  64 a 1006.71 0.18346 YES YES  65 a 1006.76 0.18386 YES YES  66 a 1042.78 2.94257 YES YES  67 a 1042.84 2.97442 YES YES  68 a 1045.99 0.00567 YES YES  69 a 1046.42 28.71502 YES YES  70 a 1050.08 0.00079 YES YES  71 a 1054.14 87.09119 YES YES  72 a 1055.39 3.80626 YES YES  73 a 1055.44 3.78505 YES YES  74 a 1136.34 115.57284 YES YES  75 a 1137.24 0.00848 YES YES  76 a 1159.68 6.10131 YES YES  77 a 1159.70 6.16968 YES YES  78 a 1170.96 29.98613 YES YES  79 a 1171.00 30.02171 YES YES  80 a 1183.36 0.00274 YES YES  81 a 1183.63 3.39127 YES YES  82 a 1247.77 56.69923 YES YES  83 a 1255.15 0.00140 YES YES  84 a 1302.18 2.10311 YES YES  85 a 1302.21 2.17669 YES YES  86 a 1346.13 22.71921 YES YES  87 a 1346.56 0.44088 YES YES  88 a 1348.24 24.75875 YES YES  89 a 1348.30 24.79016 YES YES  90 a 1385.12 4.32626 YES YES  91 a 1385.71 0.00657 YES YES  92 a 1460.75 0.00395 YES YES  93 a 1461.49 15.56664 YES YES  94 a 1465.14 0.01044 YES YES  95 a 1465.17 0.00290 YES YES  96 a 1498.71 50.58281 YES YES  97 a 1498.73 50.82940 YES YES  98 a 1540.06 0.02860 YES YES  99 a 1540.42 4.90331 YES YES  100 a 1563.22 328.44843 YES YES  101 a 1563.34 332.57427 YES YES  102 a 1566.46 0.62162 YES YES  103 a 1567.55 1.46028 YES YES  104 a 1625.35 322.41082 YES YES  105 a 1627.47 0.11165 YES YES  106 a 1634.00 26.75129 YES YES  107 a 1634.04 26.82766 YES YES  108 a 3190.97 0.53503 YES YES  109 a 3191.10 2.74497 YES YES  110 a 3191.15 2.34276 YES YES  111 a 3191.45 0.11287 YES YES  112 a 3198.44 5.56679 YES YES  113 a 3198.48 5.55976 YES YES  114 a 3201.10 3.76917 YES YES  115 a 3201.16 0.95731 YES YES  116 a 3211.77 0.32094 YES YES  117 a 3211.80 0.32422 YES YES  118 a 3218.96 3.65863 YES YES  119 a 3219.01 0.88803 YES YES  120 a 3221.18 0.14216 YES YES  121 a 3221.24 0.14135 YES YES  122 a 3222.82 0.10631 YES YES  123 a 3222.85 0.03782 YES YES |

### [In(bipy)_2_]^+^

Optimized atomic coordinates from the DFT calculations (BHLYP/SV(P) level) (Bohr Units):

| 8.34695407773022 9.09433398722493 43.04781985745032 In  12.02925615658460 6.94963396198713 40.52787718944533 N  8.14622262281469 4.32829716837255 42.61615191576563 N  10.79821146327704 7.84972002624187 46.95886995833274 N  5.71746422314627 8.43753655610032 47.16174418874606 N  13.71532400577865 8.41014655974153 39.38960960810416 C  11.61560943012923 4.61696133296188 39.67316680265110 C  9.68368092491165 3.09809709413501 41.03409527577380 C  6.38250229037776 3.05256878787934 43.85957454428202 C  13.24898694786896 7.36069601999473 46.76349357481956 C  9.70907888788632 7.84941982308235 49.23805226178987 C  6.96204802870031 8.42841635118807 49.35146968737057 C  3.26981773247937 8.96505502008338 47.12162100436753 C  15.12468340611725 7.62189001265780 37.32798245746367 C  13.94174127656147 10.30733523831633 40.14639312838145 H  12.91964573522224 3.68667219137706 37.58227679274168 C  9.47147100982340 0.49635549081324 40.68804781374315 C  6.03991564581948 0.46877630807277 43.61399513104007 C  5.19120665658863 4.14670621901800 45.11902363712059 H  14.02810194631840 7.38115849992331 44.86729127318732 H  14.77431121025383 6.83498105685581 48.82528002790111 C  11.10862020644233 7.32753999738252 51.40646074774435 C  5.72697600957964 8.97278607691501 51.61334388521763 C  1.88938480897327 9.48945090311409 49.28425065661734 C  2.36884206259540 8.98590485110362 45.27462316977645 H  16.49225114216394 8.87775493887463 36.46332153965901 H  14.69872123051201 5.20718432011864 36.40556471630748 C  12.53988723075556 1.81763561235493 36.84600118852378 H  10.74652458003739 -0.50039885479009 39.43939651188636 H  7.62983684880294 -0.83171673646512 41.99109917243264 C  4.57096179217078 -0.48526046705536 44.67557917735631 H  16.76827652610197 6.44638361602053 48.56463951849437 H  13.66780209638956 6.81671846045911 51.19756162191275 C  10.21704570950162 7.29047783815259 53.24526732813129 H  6.74164230929384 9.02283638468047 53.38721955618490 H  3.16105564533369 9.49875643244036 51.57456473097912 C  -0.11376270242371 9.89922736965273 49.16425983706684 H  15.72622161479967 4.51292475437319 34.77287152671190 H  7.45039395678708 -2.85896489491030 41.74301689328824 H  14.77619662209061 6.40317360532856 52.87200900590112 H  2.17373936878296 9.92887126377205 53.31913814146734 H |
| --- |

List of calculated frequencies (BHLYP/SV(P) level):

| mode symmetry wave number IR intensity selection rules  cm**(-1) km/mol IR RAMAN |
| --- |
| 1 -0.00 0.00000 - -  2 -0.00 0.00000 - -  3 -0.00 0.00000 - -  4 0.00 0.00000 - -  5 0.00 0.00000 - -  6 0.00 0.00000 - -  7 a 20.99 0.33350 YES YES  8 a 24.71 0.94332 YES YES  9 a 28.61 1.68205 YES YES  10 a 63.72 0.25747 YES YES  11 a 70.80 0.04208 YES YES  12 a 73.11 0.41108 YES YES  13 a 81.71 0.32721 YES YES  14 a 83.59 0.02547 YES YES  15 a 105.63 14.66720 YES YES  16 a 114.85 0.30474 YES YES  17 a 116.30 2.12948 YES YES  18 a 117.72 5.74791 YES YES  19 a 158.61 22.24178 YES YES  20 a 204.42 8.83825 YES YES  21 a 207.71 10.30193 YES YES  22 a 256.24 1.54947 YES YES  23 a 259.26 1.01949 YES YES  24 a 354.80 1.04164 YES YES  25 a 355.74 1.05650 YES YES  26 a 435.31 2.83774 YES YES  27 a 438.41 3.93341 YES YES  28 a 443.40 1.83025 YES YES  29 a 448.43 0.10593 YES YES  30 a 450.70 6.60709 YES YES  31 a 462.48 8.75397 YES YES  32 a 489.13 2.33087 YES YES  33 a 492.71 2.39135 YES YES  34 a 591.00 0.64742 YES YES  35 a 593.51 0.13961 YES YES  36 a 653.18 17.75421 YES YES  37 a 655.56 4.52922 YES YES  38 a 672.40 12.27883 YES YES  39 a 674.51 25.27668 YES YES  40 a 690.64 2.27775 YES YES  41 a 692.22 5.28604 YES YES  42 a 795.94 1.54270 YES YES  43 a 797.40 2.41099 YES YES  44 a 797.56 3.95422 YES YES  45 a 799.85 24.34450 YES YES  46 a 811.04 2.62124 YES YES  47 a 811.20 8.78481 YES YES  48 a 815.34 71.92971 YES YES  49 a 815.48 68.07843 YES YES  50 a 883.83 1.85037 YES YES  51 a 885.06 0.62142 YES YES  52 a 960.90 0.18929 YES YES  53 a 961.34 2.31324 YES YES  54 a 962.39 0.13695 YES YES  55 a 963.02 0.19650 YES YES  56 a 1046.64 0.19483 YES YES  57 a 1047.37 0.53826 YES YES  58 a 1051.90 0.84542 YES YES  59 a 1052.53 0.34228 YES YES  60 a 1060.81 45.93600 YES YES  61 a 1063.00 0.79573 YES YES  62 a 1066.65 27.24255 YES YES  63 a 1067.10 45.71480 YES YES  64 a 1085.18 0.13194 YES YES  65 a 1085.47 0.07472 YES YES  66 a 1087.58 0.27625 YES YES  67 a 1087.87 0.14071 YES YES  68 a 1097.63 1.21856 YES YES  69 a 1098.22 0.15356 YES YES  70 a 1125.47 7.47667 YES YES  71 a 1126.27 1.06116 YES YES  72 a 1137.87 5.53364 YES YES  73 a 1138.40 2.54384 YES YES  74 a 1164.71 11.16674 YES YES  75 a 1165.18 3.77677 YES YES  76 a 1178.55 0.37166 YES YES  77 a 1179.92 0.54424 YES YES  78 a 1205.65 4.44649 YES YES  79 a 1205.89 21.47638 YES YES  80 a 1223.39 6.73245 YES YES  81 a 1224.21 7.23532 YES YES  82 a 1339.03 7.51179 YES YES  83 a 1339.24 12.57696 YES YES  84 a 1344.55 31.82453 YES YES  85 a 1347.46 1.39760 YES YES  86 a 1364.18 0.03798 YES YES  87 a 1364.63 0.12655 YES YES  88 a 1387.32 8.30551 YES YES  89 a 1388.12 33.03466 YES YES  90 a 1391.55 8.74066 YES YES  91 a 1392.36 1.78497 YES YES  92 a 1521.33 1.23841 YES YES  93 a 1521.78 4.35270 YES YES  94 a 1536.04 32.08706 YES YES  95 a 1536.68 109.11066 YES YES  96 a 1573.60 40.93344 YES YES  97 a 1576.14 14.80385 YES YES  98 a 1600.19 5.32109 YES YES  99 a 1601.86 20.93745 YES YES  100 a 1703.20 79.92526 YES YES  101 a 1703.75 19.08027 YES YES  102 a 1720.00 6.33128 YES YES  103 a 1721.04 13.55240 YES YES  104 a 1730.32 7.85133 YES YES  105 a 1730.46 61.06039 YES YES  106 a 1738.18 59.27505 YES YES  107 a 1739.24 40.21434 YES YES  108 a 3274.43 4.34075 YES YES  109 a 3274.62 5.53553 YES YES  110 a 3298.88 2.42799 YES YES  111 a 3299.05 2.62206 YES YES  112 a 3300.47 0.69593 YES YES  113 a 3300.69 0.48590 YES YES  114 a 3307.77 1.19339 YES YES  115 a 3308.07 1.33541 YES YES  116 a 3317.87 0.87496 YES YES  117 a 3318.18 0.75950 YES YES  118 a 3320.27 1.41126 YES YES  119 a 3320.36 1.36002 YES YES  120 a 3323.43 0.16746 YES YES  121 a 3323.99 0.23964 YES YES  122 a 3336.84 0.16845 YES YES  123 a 3338.32 0.17935 YES YES |

Optimized atomic coordinates from the DFT calculations (B3LYP/SV(P) level) (Bohr Units):

| 8.37134025995677 8.95861730980252 43.11763104726865 In  12.05794996277598 6.90356034620157 40.53826741350937 N  8.17128664765964 4.21346845266066 42.61472992306400 N  10.82138420825405 7.78531433501834 47.04084945341476 N  5.69874077311868 8.35906630820986 47.21888049615266 N  13.76105424231566 8.39796944561224 39.41645666920532 C  11.60976909003356 4.58064369295281 39.59605261771025 C  9.67890936353827 3.02120739513789 40.93394615710978 C  6.40555903950039 2.88972930375421 43.85357286964253 C  13.29368539686240 7.27146493195031 46.87322798860171 C  9.72011381335923 7.90136526633639 49.34151238271648 C  6.96337174007851 8.48764992227769 49.42557104530877 C  3.22451623258077 8.87634612337547 47.16528974033027 C  15.14817190930795 7.66177257432483 37.29721435239600 C  14.01450842887288 10.28168437041067 40.24329468336619 H  12.88975676530026 3.71459074167338 37.43530196008804 C  9.41848197552545 0.41699891394980 40.48305666222718 C  6.02969703832636 0.30336213583313 43.50540418039448 C  5.24632658490594 3.95566822307872 45.19372797004666 H  14.07667659698599 7.19629014407016 44.96080263878705 H  14.82427751924257 6.83738492129568 48.97579857252103 C  11.13659573321608 7.48222372222977 51.54847676486720 C  5.72156431976295 9.17883544453208 51.66999382989234 C  1.84351036800212 9.52757108412360 49.31603189580413 C  2.30769950733998 8.78151778128140 45.30871198197665 H  16.53311038991251 8.94320695496619 36.45701212376355 H  14.68230485759190 5.26898139964282 36.28396195788739 C  12.47832002308300 1.86350066265272 36.62583517942350 H  10.66351250548914 -0.55120380063112 39.15564668490981 H  7.57861745944068 -0.95433513463881 41.77939480618097 C  4.56464212707796 -0.68898911766167 44.57062402208731 H  16.83230400371280 6.41880572427381 48.73393381021550 H  13.71048770007685 6.94568403182152 51.36489222651594 C  10.23874119023953 7.53835809786789 53.40318031775645 H  6.75297961058634 9.35232237934214 53.44673854764406 H  3.13550785798945 9.69255865437503 51.61228278717752 C  -0.18051114002194 9.91643081832116 49.18126635742532 H  15.69024243566339 4.61943634844916 34.60007262590561 H  7.36871653963015 -2.98404850961862 41.44853388171430 H  14.82626386251973 6.60705220001816 53.07182848103131 H  2.14666379726628 10.23397957827605 53.34501795009705 H |
| --- |

List of calculated frequencies (B3LYP/SV(P) level):

| mode symmetry wave number IR intensity selection rules  cm**(-1) km/mol IR RAMAN |
| --- |
| 1 -0.00 0.00000 - -  2 -0.00 0.00000 - -  3 -0.00 0.00000 - -  4 0.00 0.00000 - -  5 0.00 0.00000 - -  6 0.00 0.00000 - -  7 a 21.18 0.03953 YES YES  8 a 24.96 1.30258 YES YES  9 a 28.81 1.59333 YES YES  10 a 63.59 0.30610 YES YES  11 a 69.05 0.13904 YES YES  12 a 69.86 0.75383 YES YES  13 a 77.45 0.13580 YES YES  14 a 82.78 0.03432 YES YES  15 a 103.26 8.23110 YES YES  16 a 108.47 0.01430 YES YES  17 a 113.54 8.49718 YES YES  18 a 116.21 2.34216 YES YES  19 a 155.24 18.55142 YES YES  20 a 197.57 3.77831 YES YES  21 a 199.43 11.03512 YES YES  22 a 244.11 2.32928 YES YES  23 a 246.49 0.94763 YES YES  24 a 342.97 0.53340 YES YES  25 a 343.38 0.46903 YES YES  26 a 417.45 4.08511 YES YES  27 a 418.65 5.77472 YES YES  28 a 424.50 1.34548 YES YES  29 a 429.55 0.08606 YES YES  30 a 433.13 5.87767 YES YES  31 a 445.03 8.53257 YES YES  32 a 471.12 2.18268 YES YES  33 a 472.53 1.45795 YES YES  34 a 569.15 0.58109 YES YES  35 a 571.47 0.15410 YES YES  36 a 631.31 14.26557 YES YES  37 a 633.49 4.14157 YES YES  38 a 649.25 7.60500 YES YES  39 a 651.83 18.50008 YES YES  40 a 666.58 2.05376 YES YES  41 a 668.01 4.55257 YES YES  42 a 762.50 1.10884 YES YES  43 a 763.76 1.02643 YES YES  44 a 766.38 1.87328 YES YES  45 a 768.47 12.02658 YES YES  46 a 780.05 46.78980 YES YES  47 a 780.68 34.30778 YES YES  48 a 782.55 44.25597 YES YES  49 a 782.93 19.38851 YES YES  50 a 849.52 1.25560 YES YES  51 a 850.39 0.30513 YES YES  52 a 919.79 0.28441 YES YES  53 a 920.07 1.97218 YES YES  54 a 922.02 0.49803 YES YES  55 a 922.50 0.13864 YES YES  56 a 995.97 0.05775 YES YES  57 a 996.54 0.30071 YES YES  58 a 1002.36 0.72857 YES YES  59 a 1002.94 0.24924 YES YES  60 a 1017.49 36.77580 YES YES  61 a 1019.02 0.40637 YES YES  62 a 1023.40 16.77815 YES YES  63 a 1024.11 28.71024 YES YES  64 a 1036.15 0.12755 YES YES  65 a 1036.41 0.05874 YES YES  66 a 1038.90 0.10398 YES YES  67 a 1039.16 0.18418 YES YES  68 a 1057.18 1.31451 YES YES  69 a 1058.01 0.32122 YES YES  70 a 1084.09 6.04125 YES YES  71 a 1084.75 0.60395 YES YES  72 a 1095.79 4.14067 YES YES  73 a 1096.05 2.84388 YES YES  74 a 1123.70 12.08216 YES YES  75 a 1124.00 2.85494 YES YES  76 a 1136.96 0.80778 YES YES  77 a 1138.48 1.61071 YES YES  78 a 1171.75 1.87405 YES YES  79 a 1171.89 10.56471 YES YES  80 a 1186.77 6.34236 YES YES  81 a 1186.93 1.80578 YES YES  82 a 1295.82 6.88363 YES YES  83 a 1298.52 0.00792 YES YES  84 a 1319.07 0.79839 YES YES  85 a 1319.63 5.77307 YES YES  86 a 1323.45 5.89170 YES YES  87 a 1324.65 0.05498 YES YES  88 a 1340.24 10.85134 YES YES  89 a 1340.90 34.65407 YES YES  90 a 1348.28 0.25539 YES YES  91 a 1348.93 1.68482 YES YES  92 a 1459.60 1.07914 YES YES  93 a 1460.42 4.68802 YES YES  94 a 1475.51 25.28211 YES YES  95 a 1476.01 93.06495 YES YES  96 a 1507.41 42.57466 YES YES  97 a 1510.06 14.97246 YES YES  98 a 1524.16 0.87982 YES YES  99 a 1526.29 7.56251 YES YES  100 a 1622.29 31.13396 YES YES  101 a 1622.90 4.66100 YES YES  102 a 1636.45 2.91003 YES YES  103 a 1637.41 7.93072 YES YES  104 a 1646.22 7.07236 YES YES  105 a 1646.36 59.12721 YES YES  106 a 1655.67 41.54128 YES YES  107 a 1656.48 30.52430 YES YES  108 a 3164.09 6.42603 YES YES  109 a 3164.22 6.56799 YES YES  110 a 3191.66 4.96415 YES YES  111 a 3191.67 2.89341 YES YES  112 a 3194.23 0.82731 YES YES  113 a 3194.29 0.38038 YES YES  114 a 3197.43 0.09425 YES YES  115 a 3197.45 0.90561 YES YES  116 a 3210.19 2.14711 YES YES  117 a 3210.28 2.70032 YES YES  118 a 3212.01 1.13756 YES YES  119 a 3212.05 0.34964 YES YES  120 a 3215.36 0.03933 YES YES  121 a 3215.49 0.03444 YES YES  122 a 3226.46 1.32809 YES YES  123 a 3226.84 1.26786 YES YES |

### [In(bipy)_2_]^+^ (triplet)

Optimized atomic coordinates from the DFT calculations (BHLYP/SV(P) level) (Bohr Units):

| 8.79538791835667 8.45440078751793 43.67978172489164 In  11.78276766463115 6.87527774295013 40.72054345127356 N  7.96748905516390 4.25050531566462 42.82961698885258 N  11.22065677510166 8.00749484859672 46.91472911870694 N  6.15750457766155 8.64409574180048 46.83688559146371 N  13.53051630563350 8.34165426594060 39.67352246106224 C  11.35055622067387 4.56269568649109 39.80353952734794 C  9.35607050614314 3.05746903538862 41.08204422758418 C  6.16420263054428 3.01429668324782 44.06756229790597 C  13.75187970865314 7.69811350888673 46.87313871810328 C  9.96141152830156 8.00673885838511 49.19652140249828 C  7.29072621288222 8.33015456611548 49.15543268879994 C  3.65311865854897 9.09545158174518 46.68734220674182 C  14.98555343494686 7.56878760552461 37.64271367720772 C  13.76947318140827 10.21316620005702 40.48419536262509 H  12.71008793260445 3.66212851153879 37.74054720023739 C  8.92362700680629 0.52610776303493 40.52954192476179 C  5.62577675276055 0.49374521192781 43.61977271523124 C  5.12343138383648 4.08767279668310 45.47089057980452 H  14.62691705832843 7.70201306670951 45.01730633166510 H  15.18830692576075 7.38881057766036 48.98803920638895 C  11.38443746322993 7.69409867232607 51.44720374661118 C  5.74487925236168 8.37147099118739 51.34805949601761 C  2.10559596785368 9.18601745843039 48.74615952626337 C  2.90307717785061 9.39134817709788 44.79729170023223 H  16.40357471990349 8.81738030101950 36.85285703157480 H  14.54980680399253 5.18022896665973 36.65676993990937 C  12.34128695203650 1.80863906944851 36.96258792919284 H  10.05326575543019 -0.45236821091833 39.13592067334149 H  7.03758598074165 -0.76634080588051 41.81069297605295 C  4.14236260614141 -0.44412568269455 44.67466863970630 H  17.21589146389531 7.15415322293538 48.84046196616791 H  13.94205624396942 7.39488572094100 51.35829129242576 C  10.43004124222347 7.70197502823405 53.25479879452975 H  6.58493454364142 8.08526496080539 53.18966780549454 H  3.20820355272100 8.78699104111546 51.15525961665546 C  0.10658119748299 9.56618502511039 48.52874726067895 H  15.62303993377559 4.50143140630330 35.04748135809576 H  6.68865560488688 -2.74426837905761 41.39989242335426 H  15.01182263269220 7.16685029173157 53.09153869580219 H  2.04429020350264 8.82544556688770 52.84200678087650 H |
| --- |

List of calculated frequencies (BHLYP/SV(P) level):

| mode symmetry wave number IR intensity selection rules  cm**(-1) km/mol IR RAMAN |
| --- |
| 1 0.00 0.00000 - -  2 0.00 0.00000 - -  3 0.00 0.00000 - -  4 0.00 0.00000 - -  5 0.00 0.00000 - -  6 0.00 0.00000 - -  7 a 23.49 0.14820 YES YES  8 a 29.04 0.45971 YES YES  9 a 35.66 0.12329 YES YES  10 a 80.21 0.02911 YES YES  11 a 83.55 0.10732 YES YES  12 a 86.84 0.47737 YES YES  13 a 104.50 0.20026 YES YES  14 a 116.44 0.55151 YES YES  15 a 131.22 1.90384 YES YES  16 a 148.40 1.74478 YES YES  17 a 174.20 16.69383 YES YES  18 a 192.75 8.96309 YES YES  19 a 200.55 0.52575 YES YES  20 a 225.16 0.26904 YES YES  21 a 232.25 9.44609 YES YES  22 a 257.39 8.68462 YES YES  23 a 259.12 27.75506 YES YES  24 a 367.09 0.53621 YES YES  25 a 384.94 3.94902 YES YES  26 a 424.52 1.76977 YES YES  27 a 434.38 0.12579 YES YES  28 a 445.16 7.46788 YES YES  29 a 445.29 12.22694 YES YES  30 a 448.14 2.12165 YES YES  31 a 464.81 3.33115 YES YES  32 a 473.28 24.74979 YES YES  33 a 485.89 1.49973 YES YES  34 a 562.49 0.37494 YES YES  35 a 590.22 0.04350 YES YES  36 a 644.82 16.63722 YES YES  37 a 664.06 10.71859 YES YES  38 a 679.40 0.54194 YES YES  39 a 682.54 13.12636 YES YES  40 a 687.66 17.99498 YES YES  41 a 689.73 10.46738 YES YES  42 a 722.37 14.38813 YES YES  43 a 768.81 0.17231 YES YES  44 a 788.69 83.47465 YES YES  45 a 793.14 15.52447 YES YES  46 a 793.69 20.64454 YES YES  47 a 798.13 0.51954 YES YES  48 a 809.72 2.29194 YES YES  49 a 818.52 0.35615 YES YES  50 a 819.35 74.10674 YES YES  51 a 882.79 0.20617 YES YES  52 a 895.68 0.71062 YES YES  53 a 897.64 1.21778 YES YES  54 a 961.68 0.56828 YES YES  55 a 965.03 0.44398 YES YES  56 a 997.63 216.92276 YES YES  57 a 1019.60 0.33473 YES YES  58 a 1024.43 0.26952 YES YES  59 a 1047.31 0.03195 YES YES  60 a 1048.78 0.05308 YES YES  61 a 1053.74 0.30579 YES YES  62 a 1059.32 0.07446 YES YES  63 a 1064.60 55.84750 YES YES  64 a 1069.86 17.02656 YES YES  65 a 1075.30 13.71917 YES YES  66 a 1076.68 33.39441 YES YES  67 a 1086.37 0.43743 YES YES  68 a 1089.16 0.11373 YES YES  69 a 1092.13 2.44660 YES YES  70 a 1092.64 0.68751 YES YES  71 a 1097.14 1.38837 YES YES  72 a 1127.69 5.25896 YES YES  73 a 1137.47 5.49955 YES YES  74 a 1167.69 46.77341 YES YES  75 a 1168.93 3.18672 YES YES  76 a 1183.05 1.39484 YES YES  77 a 1196.35 23.34755 YES YES  78 a 1209.23 9.00205 YES YES  79 a 1209.88 48.66999 YES YES  80 a 1226.59 6.19664 YES YES  81 a 1230.77 9.71541 YES YES  82 a 1291.54 45.00967 YES YES  83 a 1341.25 23.46488 YES YES  84 a 1346.85 5.86264 YES YES  85 a 1349.40 5.27744 YES YES  86 a 1367.31 0.20622 YES YES  87 a 1380.81 29.89306 YES YES  88 a 1388.48 20.79514 YES YES  89 a 1389.44 2.22615 YES YES  90 a 1391.57 35.02208 YES YES  91 a 1446.03 0.36521 YES YES  92 a 1514.78 13.30112 YES YES  93 a 1516.23 25.11332 YES YES  94 a 1523.28 6.48533 YES YES  95 a 1539.28 67.78485 YES YES  96 a 1553.01 68.93834 YES YES  97 a 1574.99 42.93060 YES YES  98 a 1600.13 15.21148 YES YES  99 a 1605.38 0.31604 YES YES  100 a 1613.78 612.71305 YES YES  101 a 1654.70 79.00007 YES YES  102 a 1685.75 131.62371 YES YES  103 a 1701.98 28.43355 YES YES  104 a 1709.53 13.94324 YES YES  105 a 1718.23 16.79359 YES YES  106 a 1730.84 51.52158 YES YES  107 a 1742.46 35.53930 YES YES  108 a 3269.61 10.25206 YES YES  109 a 3285.76 1.22628 YES YES  110 a 3288.02 9.69746 YES YES  111 a 3297.12 5.62420 YES YES  112 a 3299.32 9.06718 YES YES  113 a 3304.17 2.11825 YES YES  114 a 3305.56 0.05249 YES YES  115 a 3311.72 4.76237 YES YES  116 a 3317.27 0.00397 YES YES  117 a 3320.92 1.77258 YES YES  118 a 3322.58 1.40366 YES YES  119 a 3322.63 0.16181 YES YES  120 a 3325.12 0.46138 YES YES  121 a 3328.33 1.55692 YES YES  122 a 3335.70 1.87835 YES YES  123 a 3343.37 0.19889 YES YES |

Optimized atomic coordinates from the DFT calculations (B3LYP/SV(P) level) (Bohr Units):

| 8.76578186150091 7.87449207556256 43.73508188825718 In  11.69020746329019 6.78333738657231 40.84053336423177 N  7.95952842831951 3.88402941564367 42.79695631704660 N  11.20167331909300 7.63092370282535 47.11899483045765 N  6.12431543604997 8.24428660470586 47.05296579444826 N  13.44946845369136 8.37139706749538 39.88849085427380 C  11.33037108626838 4.45330158819889 39.76237113808427 C  9.33536766046579 2.87715831680604 40.83608785099417 C  6.11744724237305 2.49529775094614 43.89694713036336 C  13.72545985339692 7.23211399189489 47.04628570196027 C  10.03756152058928 8.10708249075822 49.39326802694283 C  7.30167797146588 8.46689420508593 49.35259899556398 c  3.60666188531713 8.63305692104760 46.86645461733891 C  14.98427055901249 7.74647900442688 37.86572935127973 c  13.60780934223800 10.21914421477345 40.81093324066393 H  12.83111583313638 3.72690865601073 37.67456417122866 C  8.75193635471469 0.42914400797930 39.94425955602797 C  5.50910858797529 0.07097492832142 43.13090931497815 C  5.11411163468264 3.39819015327450 45.46419814700503 H  14.52754648541205 6.83590175870452 45.18144582757128 H  15.24178897806676 7.29873697999193 49.17458792224581 C  11.50978665606197 8.25301418718659 51.61622635503290 C  5.85508077316746 9.07763363554760 51.51456425113744 c  2.09971301898338 9.20958551625988 48.92422977571278 C  2.79462196848281 8.46930671132316 44.96805404221607 H  16.39421145868591 9.08707185379322 37.17621448920767 H  14.65555765375121 5.35331951437515 36.73326398157371 C  12.55768570218053 1.88575732145829 36.78913823260525 H  9.80450090127369 -0.37486304209867 38.36518314830862 H  6.85532970832897 -0.97519282307290 41.07968426772094 C  4.02359106623167 -0.98248424622959 44.10234848249296 H  17.26716002464143 6.93359066626337 49.01771008796097 H  14.09542195099787 7.84922382345140 51.51940883537398 C  10.61369657455037 8.66800826324490 53.42535830048740 H  6.76683565122049 9.27521455072710 53.35264319329973 H  3.26885686449170 9.43781180077032 51.31157658698543 C  0.06880864846808 9.48690461418648 48.68133392092210 H  15.81605748764028 4.78698210833339 35.12010476890235 H  6.41644258959115 -2.87349879161301 40.39166535545445 H  15.22685935231965 7.95215993912744 53.24531650873767 H  2.15342272895189 9.90764635348993 52.98633643104095 H |
| --- |

List of calculated frequencies (B3LYP/SV(P) level):

| mode symmetry wave number IR intensity selection rules  cm**(-1) km/mol IR RAMAN |
| --- |
| 1 0.00 0.00000 - -  2 0.00 0.00000 - -  3 0.00 0.00000 - -  4 0.00 0.00000 - -  5 0.00 0.00000 - -  6 0.00 0.00000 - -  7 a 25.13 0.21302 YES YES  8 a 29.99 0.61189 YES YES  9 a 32.25 3.72732 YES YES  10 a 81.53 11.27782 YES YES  11 a 82.67 0.49006 YES YES  12 a 90.67 12.49744 YES YES  13 a 95.69 0.01054 YES YES  14 a 121.23 0.31353 YES YES  15 a 123.72 49.88874 YES YES  16 a 149.30 3.04178 YES YES  17 a 165.44 114.35242 YES YES  18 a 178.72 4.07574 YES YES  19 a 189.62 3.01907 YES YES  20 a 227.18 69.19591 YES YES  21 a 232.73 12.07656 YES YES  22 a 232.86 8.39457 YES YES  23 a 236.36 0.37316 YES YES  24 a 364.42 0.00142 YES YES  25 a 366.74 3.53837 YES YES  26 a 419.33 3.51732 YES YES  27 a 421.75 0.06882 YES YES  28 a 424.53 19.93325 YES YES  29 a 433.90 15.56412 YES YES  30 a 438.58 5.43614 YES YES  31 a 438.77 0.59879 YES YES  32 a 458.74 3.50168 YES YES  33 a 460.54 11.20809 YES YES  34 a 554.29 5.54859 YES YES  35 a 556.65 0.00403 YES YES  36 a 635.38 3.28126 YES YES  37 a 635.67 0.10311 YES YES  38 a 657.65 134.75116 YES YES  39 a 658.85 0.32221 YES YES  40 a 659.60 16.86676 YES YES  41 a 660.35 0.10978 YES YES  42 a 725.54 4.64437 YES YES  43 a 725.67 8.85075 YES YES  44 a 749.19 1.43151 YES YES  45 a 749.34 0.12024 YES YES  46 a 765.32 259.62291 YES YES  47 a 768.60 79.13497 YES YES  48 a 769.38 45.46849 YES YES  49 a 773.63 13.92425 YES YES  50 a 815.99 0.37939 YES YES  51 a 816.68 0.45796 YES YES  52 a 891.20 30.10015 YES YES  53 a 891.44 4.26795 YES YES  54 a 892.76 1.06359 YES YES  55 a 893.63 8.31662 YES YES  56 a 949.67 3588.68167 YES YES  57 a 981.93 0.03918 YES YES  58 a 982.32 0.48658 YES YES  59 a 987.41 0.39168 YES YES  60 a 987.63 0.16536 YES YES  61 a 1002.63 5.51289 YES YES  62 a 1004.26 25.03633 YES YES  63 a 1012.03 0.19671 YES YES  64 a 1012.27 0.04980 YES YES  65 a 1016.97 0.06202 YES YES  66 a 1017.22 0.10929 YES YES  67 a 1022.46 0.51479 YES YES  68 a 1047.78 20.05931 YES YES  69 a 1049.47 2.98091 YES YES  70 a 1068.98 27.90977 YES YES  71 a 1069.80 0.31979 YES YES  72 a 1071.17 2.16655 YES YES  73 a 1072.47 7.57550 YES YES  74 a 1125.70 27.79892 YES YES  75 a 1126.22 0.64285 YES YES  76 a 1147.45 2.52863 YES YES  77 a 1149.60 10.67182 YES YES  78 a 1169.92 918.86925 YES YES  79 a 1171.49 9.95196 YES YES  80 a 1172.32 218.62924 YES YES  81 a 1188.92 0.16904 YES YES  82 a 1262.29 554.90858 YES YES  83 a 1270.19 1.81940 YES YES  84 a 1304.91 2429.86376 YES YES  85 a 1310.87 1.44410 YES YES  86 a 1314.30 294.75134 YES YES  87 a 1343.02 0.86732 YES YES  88 a 1347.32 1.65416 YES YES  89 a 1347.84 0.18931 YES YES  90 a 1354.64 173.16238 YES YES  91 a 1361.29 0.69125 YES YES  92 a 1431.67 2834.04247 YES YES  93 a 1453.46 0.09980 YES YES  94 a 1458.06 217.23840 YES YES  95 a 1469.96 51.91621 YES YES  96 a 1470.48 18.37294 YES YES  97 a 1498.72 121.55634 YES YES  98 a 1499.96 28.93625 YES YES  99 a 1522.31 4.43694 YES YES  100 a 1573.71 193.29312 YES YES  101 a 1583.72 75.78925 YES YES  102 a 1587.70 253.69390 YES YES  103 a 1588.64 0.88188 YES YES  104 a 1612.58 6.34896 YES YES  105 a 1615.53 185.30110 YES YES  106 a 1633.80 2.42141 YES YES  107 a 1636.14 0.18034 YES YES  108 a 3170.35 19.37484 YES YES  109 a 3170.42 4.23813 YES YES  110 a 3190.05 5.52476 YES YES  111 a 3190.39 4.75189 YES YES  112 a 3197.43 1.08140 YES YES  113 a 3197.50 1.99266 YES YES  114 a 3199.16 0.67693 YES YES  115 a 3199.21 0.03803 YES YES  116 a 3213.95 0.32346 YES YES  117 a 3214.15 0.39662 YES YES  118 a 3216.94 14.11677 YES YES  119 a 3217.07 0.61952 YES YES  120 a 3218.89 0.18896 YES YES  121 a 3218.95 0.30386 YES YES  122 a 3229.39 1.54834 YES YES  123 a 3229.72 1.47755 YES YES |

### [Ga(bipy)_3_]^+^

Optimized atomic coordinates from the DFT calculations (BHLYP/SV(P) level) (Bohr Units):

| -6.83717358953923 8.13317018230287 11.82028662907442 Ga  -4.85558140191647 9.81285082291615 9.02612569856455 N  -8.94128032658606 11.28748532440878 11.40106467319715 N  -4.78880193561454 9.74613395000189 14.74961317111227 N  -8.80721410200512 6.85016286808033 15.02766885316284 N  -4.75086430748045 4.75395920708388 11.71540734770308 N  -8.86924241824103 5.99558226450934 9.24572978495636 N  -2.84774640492731 8.84690555238914 7.78853031831964 C  -5.90688150741624 12.04011910887494 8.19715157620339 C  -8.03230375467487 12.91555965082995 9.59132996415915 C  -10.93577684188268 11.99251325465669 12.82214328932088 C  -2.81107456914732 11.29089664482903 14.41687637626089 C  -5.85169343892869 9.53365963203875 17.07433554781344 C  -8.01381540959711 7.85437490859644 17.24003624430689 c  -10.74781837982587 5.24399123171694 14.99127879263378 C  -2.75905549528289 4.22589919927624 13.16707096954110 C  -5.51327881218362 3.08951877925012 9.92936335338917 C  -7.72930335604500 3.82145018430891 8.49428239687486 c  -10.90191039072343 6.83715157567933 7.99039259732149 C  -2.09493325661773 7.08996200780617 8.52446023942877 H  -1.76775407015812 9.98018238549908 5.73715834561992 C  -4.86918013831992 13.27074691115495 6.07032631662649 C  -9.20508941246414 15.28720681486780 9.25069039187791 C  -12.14022547827035 14.25842999324757 12.55002865748809 C  -11.57149920891921 10.63663691891065 14.21939821870855 H  -2.07089543589704 11.40378248784041 12.51087326808704 H  -1.75938102487385 12.66682248347214 16.35135647340534 C  -4.88874030764376 10.91606277309999 19.11345086777438 C  -9.27609430958112 7.25829009696323 19.49149215785527 C  -12.04565437611441 4.55048096075857 17.13786169221576 C  -11.26760962191531 4.50252194698539 13.15295542089192 H  -2.26524839632361 5.63951768574824 14.56607473094268 H  -1.38029995780020 2.03290049051556 12.92937975816755 C  -4.16739952914963 0.83595895471518 9.56136242394193 C  -8.67149361544101 2.46706896220544 6.42606292215658 C  -11.93360194236130 5.57201801331624 5.97268185824778 C  -11.70501550416183 8.59799744581144 8.65924237744053 H  -0.14152389587921 9.13004087818281 4.83046758033824 H  -2.82730468287772 12.26838072025029 4.84514238832687 C  -5.72004099336451 15.00240645776748 5.39041174980837 H  -8.46932264773444 16.58603416477522 7.85198142782546 H  -11.23263891114662 15.96501548470154 10.70124468845392 C  -13.74714977415378 14.71725620013206 13.73180299437803 H  -0.14215075955335 13.86923480638826 15.99226564082016 H  -2.83867327179740 12.47921015392660 18.76437810756989 C  -5.75825862499545 10.77093639920622 20.95863783341546 H  -8.66361292144333 8.06931544797188 21.26545277400672 H  -11.28813150826512 5.60943130977250 19.44110957033531 C  -13.60147702017343 3.22582960711190 17.02299289695684 H  0.21308968229607 1.67417396151115 14.16260485421649 H  -2.10700178863333 0.30744245092690 11.05746588835401 C  -4.75594115660797 -0.49480936612562 8.12517337881937 H  -7.74114172766873 0.75413357628697 5.80762932545386 H  -10.77592536793218 3.32827750439206 5.16354505677415 C  -13.59666500138306 6.31554598180583 5.03908526709994 H  -2.04184226036535 13.20703295972475 3.20172754474097 H  -12.12153601166661 17.79395891282379 10.44879326137209 H  -2.08280929957877 13.55814372662192 20.33392280795163 H  -12.26713188192739 5.13141425584702 21.17750608229815 H  -1.06473868877909 -1.43736220810604 10.79044126574940 H  -11.51473052898553 2.29119959968602 3.55812776753703 H |
| --- |

List of calculated frequencies (BHLYP/SV(P) level):

| mode symmetry wave number IR intensity selection rules  cm**(-1) km/mol IR RAMAN |
| --- |
| 1 -0.00 0.00000 - -  2 0.00 0.00000 - -  3 0.00 0.00000 - -  4 0.00 0.00000 - -  5 0.00 0.00000 - -  6 0.00 0.00000 - -  7 a 20.77 16.43916 YES YES  8 a 34.96 9.48346 YES YES  9 a 36.91 1.73309 YES YES  10 a 37.71 17.65876 YES YES  11 a 41.16 0.12353 YES YES  12 a 58.57 0.49456 YES YES  13 a 87.56 1.38349 YES YES  14 a 88.75 41.90257 YES YES  15 a 97.15 0.41081 YES YES  16 a 115.25 484.53814 YES YES  17 a 116.26 42.81387 YES YES  18 a 118.20 5.56686 YES YES  19 a 133.64 5.76110 YES YES  20 a 141.57 48.87511 YES YES  21 a 153.41 1.06264 YES YES  22 a 163.85 284.48501 YES YES  23 a 173.89 7.21846 YES YES  24 a 192.81 36.62130 YES YES  25 a 200.37 172.52875 YES YES  26 a 202.85 215.13248 YES YES  27 a 247.35 196.08409 YES YES  28 a 250.68 112.79831 YES YES  29 a 258.41 26.24284 YES YES  30 a 263.22 1.43555 YES YES  31 a 264.86 1.39032 YES YES  32 a 265.61 127.00349 YES YES  33 a 279.56 2.77299 YES YES  34 a 385.10 18.52721 YES YES  35 a 386.55 0.60994 YES YES  36 a 395.65 2.26505 YES YES  37 a 423.30 130.29707 YES YES  38 a 439.51 22.91388 YES YES  39 a 446.34 0.29444 YES YES  40 a 447.87 7.93485 YES YES  41 a 452.38 1.59517 YES YES  42 a 455.17 5.95435 YES YES  43 a 460.86 12.18318 YES YES  44 a 462.08 7.71725 YES YES  45 a 469.44 51.77360 YES YES  46 a 486.00 29.89310 YES YES  47 a 488.59 5.65193 YES YES  48 a 504.46 4.66080 YES YES  49 a 573.59 11.25644 YES YES  50 a 577.81 13.61940 YES YES  51 a 581.67 1.20823 YES YES  52 a 652.97 97.22754 YES YES  53 a 661.18 32.41201 YES YES  54 a 662.23 3.74867 YES YES  55 a 671.92 672.36097 YES YES  56 a 682.16 209.16426 YES YES  57 a 683.00 4.37755 YES YES  58 a 684.61 2.51300 YES YES  59 a 685.91 98.01027 YES YES  60 a 694.30 5.89038 YES YES  61 a 727.19 40.43642 YES YES  62 a 753.31 235.83782 YES YES  63 a 758.15 30.96864 YES YES  64 a 776.94 0.08410 YES YES  65 a 785.62 593.15503 YES YES  66 a 788.04 39.79783 YES YES  67 a 789.89 12.34383 YES YES  68 a 791.22 339.25960 YES YES  69 a 797.56 325.34894 YES YES  70 a 804.97 50.61856 YES YES  71 a 806.31 17.54330 YES YES  72 a 808.59 36.71881 YES YES  73 a 823.03 5.60155 YES YES  74 a 849.58 4.68809 YES YES  75 a 851.43 3.44595 YES YES  76 a 903.52 96.67488 YES YES  77 a 905.87 71.59789 YES YES  78 a 929.82 2479.27015 YES YES  79 a 933.86 284.15825 YES YES  80 a 934.21 34.72241 YES YES  81 a 936.01 56.05622 YES YES  82 a 941.98 3511.18051 YES YES  83 a 948.76 4341.01637 YES YES  84 a 1025.09 0.75017 YES YES  85 a 1025.72 0.09599 YES YES  86 a 1033.89 51.58913 YES YES  87 a 1041.65 2.28572 YES YES  88 a 1043.08 2.50454 YES YES  89 a 1044.48 0.62000 YES YES  90 a 1046.47 1.22165 YES YES  91 a 1047.62 1.15725 YES YES  92 a 1049.61 0.20179 YES YES  93 a 1057.15 14.55747 YES YES  94 a 1058.63 12.59880 YES YES  95 a 1059.32 9.68184 YES YES  96 a 1060.56 4.24735 YES YES  97 a 1066.76 0.30904 YES YES  98 a 1068.03 0.31654 YES YES  99 a 1084.77 11.87483 YES YES  100 a 1085.30 0.44794 YES YES  101 a 1090.34 6.04056 YES YES  102 a 1091.23 13.85083 YES YES  103 a 1093.30 9.79342 YES YES  104 a 1097.25 1.80989 YES YES  105 a 1108.04 6.55301 YES YES  106 a 1109.07 5.47944 YES YES  107 a 1113.97 6.07438 YES YES  108 a 1116.51 4.16447 YES YES  109 a 1166.73 76.25893 YES YES  110 a 1169.16 148.22794 YES YES  111 a 1173.82 11.94944 YES YES  112 a 1189.07 329.34739 YES YES  113 a 1190.21 26.73781 YES YES  114 a 1196.72 391.27363 YES YES  115 a 1201.10 373.91968 YES YES  116 a 1202.85 581.92590 YES YES  117 a 1207.54 95.50133 YES YES  118 a 1208.33 156.43178 YES YES  119 a 1208.66 47.04295 YES YES  120 a 1225.36 3.72449 YES YES  121 a 1293.62 198.47034 YES YES  122 a 1304.22 1615.14985 YES YES  123 a 1327.00 7.74651 YES YES  124 a 1342.54 1597.34683 YES YES  125 a 1347.98 1321.69579 YES YES  126 a 1351.78 191.14118 YES YES  127 a 1356.06 87.13362 YES YES  128 a 1360.23 1588.02324 YES YES  129 a 1387.80 25.03461 YES YES  130 a 1388.64 43.29878 YES YES  131 a 1393.33 11.54194 YES YES  132 a 1398.64 70.76210 YES YES  133 a 1400.80 191.19142 YES YES  134 a 1405.45 339.90468 YES YES  135 a 1434.00 639.91445 YES YES  136 a 1465.36 1581.07862 YES YES  137 a 1478.27 2372.86753 YES YES  138 a 1516.86 0.55309 YES YES  139 a 1518.31 3.06318 YES YES  140 a 1523.93 47.84610 YES YES  141 a 1527.07 61.44125 YES YES  142 a 1533.76 74.65735 YES YES  143 a 1534.74 104.38143 YES YES  144 a 1558.51 66.43815 YES YES  145 a 1561.14 28.74656 YES YES  146 a 1563.29 19.34219 YES YES  147 a 1607.04 1.73552 YES YES  148 a 1631.93 37.45061 YES YES  149 a 1639.76 228.60838 YES YES  150 a 1647.10 172.34611 YES YES  151 a 1662.20 12.09388 YES YES  152 a 1669.82 111.31174 YES YES  153 a 1672.47 152.53035 YES YES  154 a 1691.96 127.93647 YES YES  155 a 1695.21 162.16518 YES YES  156 a 1697.59 29.18205 YES YES  157 a 1715.41 77.34785 YES YES  158 a 1715.65 0.83557 YES YES  159 a 1721.70 60.03941 YES YES  160 a 3296.48 1.46975 YES YES  161 a 3298.47 4.95180 YES YES  162 a 3299.86 1.69340 YES YES  163 a 3300.11 1.73402 YES YES  164 a 3302.12 0.92578 YES YES  165 a 3302.28 0.83006 YES YES  166 a 3307.35 1.90968 YES YES  167 a 3308.84 17.85599 YES YES  168 a 3309.60 9.40587 YES YES  169 a 3309.99 9.00139 YES YES  170 a 3314.49 1.78072 YES YES  171 a 3314.66 13.49997 YES YES  172 a 3314.85 6.03417 YES YES  173 a 3319.66 1.30725 YES YES  174 a 3319.89 1.68431 YES YES  175 a 3322.58 0.39661 YES YES  176 a 3323.95 4.77575 YES YES  177 a 3325.61 2.68229 YES YES  178 a 3325.81 2.21292 YES YES  179 a 3327.16 8.46465 YES YES  180 a 3328.33 0.92810 YES YES  181 a 3328.48 1.37021 YES YES  182 a 3333.25 1.55310 YES YES  183 a 3333.63 1.12597 YES YES |

Optimized atomic coordinates from the DFT calculations (B3LYP/SV(P) level) (Bohr Units):

| 0.01693478537195 -0.13907721646635 0.03246909360099 Ga  2.03124007591731 2.66445925711037 -1.82091161654784 N  -1.98624997227982 3.04184685850732 1.13356516163942 N  2.17485457932003 -0.00575002924104 3.31784174898006 N  -1.99529747138509 -2.62470096723398 2.39829871916462 N  2.01844250394115 -3.13734470690759 -1.65396753207251 N  -2.13898656242083 -0.80294549073256 -3.19150019537573 N  3.98511154217043 2.29434446652355 -3.43244387790681 C  1.05444454442342 5.06491488324227 -1.52992293648117 C  -1.02880778631793 5.28986760788269 0.22241826823664 C  -3.93531950793663 3.09076701376313 2.79175261181920 C  4.24821561807695 1.44751386854942 3.65812768170500 C  1.14826830847130 -1.26500777006702 5.35089879264559 C  -1.09634022323903 -2.75392715691306 4.82667178991814 C  -4.02922154404047 -3.99103970329093 1.71816460320245 C  4.04364242960598 -4.30980071102617 -0.65796989409295 C  1.13517649135319 -3.83176504734120 -3.99070121061169 C  -1.10112189955237 -2.50418664958506 -4.86586285598519 C  -4.20661786432605 0.53165476530621 -3.87883893234305 C  4.68164674258999 0.35645373606551 -3.58480361531908 H  5.07993929934456 4.22170664135432 -4.81269053027879 C  2.08952705208484 7.09756465723742 -2.92146597842864 C  -2.08115130275509 7.60767186570469 1.03510708948610 C  -5.04501866493296 5.30349823088874 3.62298482984979 C  -4.61455720599963 1.25478816180359 3.44861671411848 H  4.95674913598531 2.41592045023195 1.97729238548560 H  5.43490847866213 1.71696588086463 5.97158922872391 C  2.26529039857010 -1.02656057615391 7.76439363062287 C  -2.36957193605509 -4.26909101895885 6.62465100721226 C  -5.32352824667607 -5.54468863718944 3.38513687420396 C  -4.62302003523647 -3.80668567734570 -0.25277201210952 H  4.62662827602107 -3.66818837771075 1.21808728471468 H  5.34263126005275 -6.21439610484385 -1.90373004485168 C  2.41559125917371 -5.72853601034173 -5.37408115258761 C  -2.19858193835428 -2.83448126584721 -7.27735406069683 C  -5.37477439014121 0.25406270042326 -6.20067031250929 C  -4.92699067899361 1.86556479319297 -2.47639865658732 H  6.66702240867618 3.81910693094918 -6.06945243984967 H  4.09121382384903 6.69552639275015 -4.55553050596330 C  1.28578772581803 8.98715427560756 -2.72698068850853 H  -1.29362935301696 9.38676358838377 0.35030283921904 H  -4.07840557113839 7.63139339496085 2.72286014429624 C  -6.62641908817730 5.23148092107269 4.94746241796289 H  7.12335013638286 2.89263021107592 6.13538639986268 H  4.40309214907913 0.44821418576461 8.08657800748743 C  1.43766249927432 -1.99923344894358 9.38360947823555 H  -1.68855972604156 -4.36202794972065 8.56857556351426 H  -4.46963427668106 -5.65989316874213 5.91166702971647 C  -6.95151480900038 -6.63607887596476 2.73982135179655 H  6.96164508097727 -7.12775564525792 -1.00789134467823 H  4.50547385110778 -6.91750857076809 -4.33934609225907 C  1.74742056067740 -6.27324129957347 -7.24712336704468 H  -1.36066932277266 -4.15861393012929 -8.61810751544514 H  -4.32988038691821 -1.47398188350785 -7.95268933890975 C  -7.05939424420440 1.35954809178709 -6.64777714760497 H  4.88410640675229 8.26441339199083 -5.64115509459160 H  -4.88427994972919 9.42344703907708 3.36145199586347 H  5.26984402653958 0.63442277664608 9.95279156503071 H  -5.44761341051160 -6.84237138209134 7.29624244592234 H  5.48866992247551 -8.39350592216717 -5.40070868475894 H  -5.18160652755062 -1.72804526250137 -9.81781219053071 H |
| --- |

List of calculated frequencies (B3LYP/SV(P) level):

| mode symmetry wave number IR intensity selection rules  cm**(-1) km/mol IR RAMAN |
| --- |
| 1 -0.00 0.00000 - -  2 -0.00 0.00000 - -  3 0.00 0.00000 - -  4 0.00 0.00000 - -  5 0.00 0.00000 - -  6 0.00 0.00000 - -  7 a 26.19 0.01320 YES YES  8 a 35.53 1.48548 YES YES  9 a 35.98 0.07767 YES YES  10 a 38.80 0.13843 YES YES  11 a 38.95 0.00022 YES YES  12 a 56.44 0.24939 YES YES  13 a 83.16 0.00315 YES YES  14 a 87.70 0.05014 YES YES  15 a 92.98 0.29506 YES YES  16 a 112.12 0.93326 YES YES  17 a 113.11 0.28283 YES YES  18 a 126.86 0.94821 YES YES  19 a 133.58 5.72980 YES YES  20 a 138.49 3.77662 YES YES  21 a 147.62 0.03035 YES YES  22 a 166.00 2.83559 YES YES  23 a 178.69 5.89922 YES YES  24 a 186.32 1.31259 YES YES  25 a 205.58 42.23255 YES YES  26 a 206.89 13.74767 YES YES  27 a 242.08 2.92458 YES YES  28 a 246.95 1.58747 YES YES  29 a 249.20 3.40693 YES YES  30 a 252.59 0.01226 YES YES  31 a 263.94 58.80844 YES YES  32 a 264.46 33.28488 YES YES  33 a 269.28 6.27791 YES YES  34 a 373.43 0.69187 YES YES  35 a 373.58 0.69527 YES YES  36 a 376.63 0.60225 YES YES  37 a 414.81 13.58464 YES YES  38 a 420.53 2.23946 YES YES  39 a 427.89 3.89523 YES YES  40 a 431.36 0.96199 YES YES  41 a 432.96 0.82642 YES YES  42 a 433.69 4.00636 YES YES  43 a 439.62 3.94278 YES YES  44 a 442.12 5.97390 YES YES  45 a 448.52 4.42245 YES YES  46 a 469.96 2.48804 YES YES  47 a 471.69 0.83585 YES YES  48 a 485.43 4.35564 YES YES  49 a 553.12 1.55998 YES YES  50 a 553.26 0.89794 YES YES  51 a 559.53 1.71670 YES YES  52 a 634.01 3.24813 YES YES  53 a 637.88 1.70992 YES YES  54 a 638.60 0.15350 YES YES  55 a 659.26 44.95101 YES YES  56 a 660.07 4.99967 YES YES  57 a 660.31 0.44532 YES YES  58 a 661.79 27.02033 YES YES  59 a 664.56 23.41263 YES YES  60 a 669.33 0.22121 YES YES  61 a 712.60 7.13851 YES YES  62 a 720.71 6.52230 YES YES  63 a 720.85 3.69841 YES YES  64 a 747.76 2.14969 YES YES  65 a 750.15 1.00643 YES YES  66 a 752.02 0.41823 YES YES  67 a 763.26 86.08078 YES YES  68 a 766.59 64.85856 YES YES  69 a 768.25 91.76744 YES YES  70 a 770.44 36.96987 YES YES  71 a 770.57 48.09524 YES YES  72 a 777.58 0.55094 YES YES  73 a 802.16 1.28072 YES YES  74 a 809.99 0.10776 YES YES  75 a 810.67 0.20580 YES YES  76 a 880.70 0.92321 YES YES  77 a 882.24 5.27061 YES YES  78 a 886.27 2.18308 YES YES  79 a 887.38 4.42036 YES YES  80 a 888.10 0.13233 YES YES  81 a 888.82 4.98523 YES YES  82 a 974.72 1234.29603 YES YES  83 a 978.46 115.95207 YES YES  84 a 978.87 12.97064 YES YES  85 a 980.36 945.67635 YES YES  86 a 985.83 11.30490 YES YES  87 a 986.46 26.15461 YES YES  88 a 989.75 0.68102 YES YES  89 a 990.49 38.63717 YES YES  90 a 1001.23 1.26937 YES YES  91 a 1003.89 19.31975 YES YES  92 a 1004.86 2.54818 YES YES  93 a 1004.94 2.51437 YES YES  94 a 1006.10 4.98499 YES YES  95 a 1011.12 26.09802 YES YES  96 a 1013.10 1.52144 YES YES  97 a 1013.26 1.58308 YES YES  98 a 1014.36 28.35698 YES YES  99 a 1040.06 0.26692 YES YES  100 a 1048.77 20.83334 YES YES  101 a 1051.30 7.03035 YES YES  102 a 1051.62 7.04809 YES YES  103 a 1062.88 11.43106 YES YES  104 a 1064.21 16.40269 YES YES  105 a 1065.49 4.05607 YES YES  106 a 1066.40 3.50197 YES YES  107 a 1069.42 3.33409 YES YES  108 a 1071.51 5.87027 YES YES  109 a 1125.37 4.14635 YES YES  110 a 1127.39 13.82027 YES YES  111 a 1129.83 1.45725 YES YES  112 a 1148.74 1.38193 YES YES  113 a 1149.04 9.94783 YES YES  114 a 1152.76 4.65777 YES YES  115 a 1168.12 2.17278 YES YES  116 a 1168.65 8.00481 YES YES  117 a 1169.40 37.28408 YES YES  118 a 1172.34 264.12616 YES YES  119 a 1172.61 345.45870 YES YES  120 a 1184.98 4.83213 YES YES  121 a 1253.04 55.62088 YES YES  122 a 1257.76 227.89307 YES YES  123 a 1273.28 17.47748 YES YES  124 a 1306.85 31.17013 YES YES  125 a 1310.44 54.42951 YES YES  126 a 1314.63 18.01542 YES YES  127 a 1333.25 981.28398 YES YES  128 a 1338.95 693.85130 YES YES  129 a 1350.27 7.72257 YES YES  130 a 1350.58 30.51948 YES YES  131 a 1351.14 39.81344 YES YES  132 a 1353.97 41.47838 YES YES  133 a 1360.96 190.09348 YES YES  134 a 1361.76 226.87320 YES YES  135 a 1374.35 55.37737 YES YES  136 a 1437.37 1525.37612 YES YES  137 a 1448.79 1617.79303 YES YES  138 a 1455.88 0.18148 YES YES  139 a 1459.85 265.39045 YES YES  140 a 1466.02 343.38044 YES YES  141 a 1470.05 93.98588 YES YES  142 a 1472.15 67.01417 YES YES  143 a 1473.44 101.80705 YES YES  144 a 1497.60 93.93236 YES YES  145 a 1501.00 16.97289 YES YES  146 a 1501.23 16.30746 YES YES  147 a 1538.75 9.31757 YES YES  148 a 1569.85 64.63719 YES YES  149 a 1573.32 157.30126 YES YES  150 a 1578.08 63.56040 YES YES  151 a 1583.03 26.96602 YES YES  152 a 1585.92 46.68661 YES YES  153 a 1586.33 204.53572 YES YES  154 a 1609.70 105.29123 YES YES  155 a 1613.04 154.29616 YES YES  156 a 1617.25 13.57108 YES YES  157 a 1634.54 43.60258 YES YES  158 a 1635.07 4.34093 YES YES  159 a 1639.82 34.36287 YES YES  160 a 3192.70 1.88427 YES YES  161 a 3192.76 2.10689 YES YES  162 a 3192.90 0.02642 YES YES  163 a 3194.68 2.14227 YES YES  164 a 3194.99 1.47682 YES YES  165 a 3195.05 1.41111 YES YES  166 a 3199.71 5.02323 YES YES  167 a 3199.75 5.00799 YES YES  168 a 3203.67 1.31496 YES YES  169 a 3205.02 6.04624 YES YES  170 a 3205.12 6.79769 YES YES  171 a 3205.39 1.34367 YES YES  172 a 3209.67 1.36450 YES YES  173 a 3210.71 0.63588 YES YES  174 a 3210.86 1.11514 YES YES  175 a 3216.95 2.42704 YES YES  176 a 3217.83 4.05482 YES YES  177 a 3218.03 1.73395 YES YES  178 a 3218.56 3.55602 YES YES  179 a 3218.95 0.48417 YES YES  180 a 3219.17 3.81019 YES YES  181 a 3220.81 4.13340 YES YES  182 a 3222.95 3.72674 YES YES  183 a 3223.14 2.28062 YES YES |

### [Ga(bipy)_3_]^2+^

Optimized atomic coordinates from the DFT calculations (BHLYP/SV(P) level) (Bohr Units):

| 41.87736831034179 22.07336654687887 40.26860047216300 Ga  38.81268084032506 22.75447542013013 42.32502651634828 N  39.52735929389768 19.90412536462370 38.30225966115972 N  38.57296090871954 24.31375843718834 44.34088756167001 C  40.27836269629245 25.27188134779482 44.95071136407089 H  36.34852758420323 24.68613180433498 45.57774806475712 C  36.25954272598325 25.94907160703994 47.18557132443376 H  34.17410405538563 23.38532752285631 44.70097238295588 C  32.37017726830233 23.63798796638423 45.63951058409056 H  34.36924428805683 21.82057647344794 42.66175143980211 C  32.71679633489705 20.83237382603567 41.97352325725105 H  36.72080874320396 21.48877883080900 41.42700146403975 C  37.11461225208998 19.92350773886285 39.28277123450011 C  35.22716424309123 18.41907627185544 38.12568508309923 C  33.32640095580177 18.42388102892087 38.87923140182869 H  35.79431899664974 16.95828125789676 36.07888301133571 C  34.34585860727643 15.80557229039260 35.20017149661718 H  38.29623154759717 16.95272419181540 35.11537708893582 C  38.81385405737885 15.80973453055335 33.49869507217512 H  40.05409177667653 18.42608775986924 36.28009719341732 C  41.98750531022424 18.47488097222324 35.60344473992060 H  43.22886942842423 19.06003434844669 42.52778565035025 N  45.22563912314652 20.87688594982094 38.32539503877609 N  41.99323275433667 18.19966019982600 44.54253535075225 C  40.36274258616734 19.28235904643440 45.14558482861852 H  42.73249675346734 16.03324053478215 45.81093060430304 C  41.67968446775692 15.39331776318345 47.44670677421500 H  44.82063239050099 14.72447349143562 44.92410627458836 C  45.45591327489066 13.01211603520451 45.85563828917808 H  46.09190650341186 15.61975696890926 42.80974408568051 C  47.70690223929078 14.59629242059461 42.08695526194786 H  45.24229280235393 17.80732053459253 41.64077718222777 C  46.43234917190992 18.90378855500375 39.35753520584620 C  48.65167361471711 17.97718250514832 38.30598667290587 C  49.63194313692644 16.39463065576181 39.15001995912753 H  49.62000379538586 19.10990522564445 36.14953514124898 C  51.35106152198865 18.40611511321089 35.30591326874449 H  48.34678227994793 21.14362716117316 35.09565225268048 C  49.03058981079923 22.08073411910323 33.40804825278951 H  46.15243336840209 21.96466687358591 36.25611157084081 C  45.09429445959054 23.54351842634202 35.49481715077054 H  41.42725185304294 25.33760793833028 37.99361446262213 N  44.20324834086628 24.82992044975586 42.11619238303874 N  39.86275538117548 25.42964551953855 36.02631765378348 C  39.00750571285528 23.65846421914475 35.45523578062217 H  39.33657484579997 27.66246999660400 34.76681015813759 C  38.05412115808709 27.66126380801978 33.17026549522966 H  40.47322941776500 29.86514155670961 35.61303153107846 C  40.10152648827195 31.65686836462940 34.68835705514710 H  42.09022775903904 29.76189583897846 37.67763518313745 C  42.96495760376283 31.47501494287559 38.36916978147880 H  42.53156472620370 27.45316259347445 38.83905943262483 C  44.19122632501225 27.13640185904990 41.06994392385231 C  45.65588938715894 29.07648451184846 42.06024176581946 C  45.65741608790860 30.93215167966427 41.20333449019601 H  47.14075923291421 28.61407052059512 44.17027142791540 C  48.29392205231139 30.11109105562161 44.96569786215038 H  47.12694462572983 26.22179139277208 45.24018117006271 C  48.25137727102472 25.77837808619709 46.89329518979194 H  45.62590205748303 24.38453051496531 44.14155106369495 C  45.55310750795721 22.49152263365707 44.91818757736966 H |
| --- |

List of calculated frequencies (BHLYP/SV(P) level):

| mode symmetry wave number IR intensity selection rules  cm**(-1) km/mol IR RAMAN |
| --- |
| 1 0.00 0.00000 - -  2 0.00 0.00000 - -  3 0.00 0.00000 - -  4 0.00 0.00000 - -  5 0.00 0.00000 - -  6 0.00 0.00000 - -  7 a 30.34 0.25114 YES YES  8 a 34.30 0.55500 YES YES  9 a 35.82 0.49340 YES YES  10 a 36.55 0.05341 YES YES  11 a 40.95 0.04079 YES YES  12 a 57.58 0.46927 YES YES  13 a 86.28 0.03945 YES YES  14 a 91.81 0.63015 YES YES  15 a 100.68 0.61540 YES YES  16 a 108.15 0.13944 YES YES  17 a 113.63 0.10493 YES YES  18 a 127.56 0.06777 YES YES  19 a 137.85 0.01851 YES YES  20 a 138.32 1.90232 YES YES  21 a 151.17 0.00304 YES YES  22 a 172.58 5.15964 YES YES  23 a 194.17 4.47967 YES YES  24 a 197.55 4.27593 YES YES  25 a 221.10 17.64087 YES YES  26 a 225.84 7.85310 YES YES  27 a 253.62 2.52306 YES YES  28 a 256.85 0.08758 YES YES  29 a 262.35 14.08436 YES YES  30 a 269.69 0.20714 YES YES  31 a 281.06 0.15712 YES YES  32 a 288.70 37.12515 YES YES  33 a 316.33 68.14313 YES YES  34 a 379.53 0.46516 YES YES  35 a 380.93 0.00430 YES YES  36 a 400.65 3.44608 YES YES  37 a 431.17 15.17403 YES YES  38 a 437.82 0.55554 YES YES  39 a 446.79 4.32428 YES YES  40 a 449.44 5.20786 YES YES  41 a 453.54 11.81880 YES YES  42 a 457.28 1.38617 YES YES  43 a 460.01 0.00138 YES YES  44 a 473.45 2.77511 YES YES  45 a 483.97 11.20947 YES YES  46 a 487.34 33.61142 YES YES  47 a 490.13 2.16567 YES YES  48 a 502.06 6.44857 YES YES  49 a 567.15 0.00035 YES YES  50 a 588.84 0.00453 YES YES  51 a 592.24 0.00748 YES YES  52 a 651.67 27.49929 YES YES  53 a 669.52 13.77874 YES YES  54 a 669.89 5.82513 YES YES  55 a 683.01 0.33730 YES YES  56 a 687.89 4.87818 YES YES  57 a 688.27 0.46775 YES YES  58 a 691.56 16.27521 YES YES  59 a 691.76 10.61921 YES YES  60 a 699.33 19.66480 YES YES  61 a 721.11 16.49929 YES YES  62 a 776.46 0.08238 YES YES  63 a 791.15 14.10792 YES YES  64 a 792.92 49.16242 YES YES  65 a 795.75 1.96107 YES YES  66 a 796.42 75.80576 YES YES  67 a 804.70 0.54238 YES YES  68 a 806.84 0.41258 YES YES  69 a 810.62 5.98402 YES YES  70 a 811.15 0.78945 YES YES  71 a 823.04 0.00989 YES YES  72 a 826.53 72.13701 YES YES  73 a 827.41 47.26968 YES YES  74 a 882.17 0.60319 YES YES  75 a 882.92 0.01658 YES YES  76 a 906.74 0.66605 YES YES  77 a 906.95 0.44377 YES YES  78 a 967.32 0.72419 YES YES  79 a 967.82 0.84277 YES YES  80 a 970.01 1.83972 YES YES  81 a 970.74 0.10867 YES YES  82 a 1005.17 252.40060 YES YES  83 a 1024.95 0.20100 YES YES  84 a 1025.20 0.12283 YES YES  85 a 1055.07 0.09499 YES YES  86 a 1055.28 0.22444 YES YES  87 a 1056.08 0.00010 YES YES  88 a 1061.38 0.23886 YES YES  89 a 1061.57 0.08266 YES YES  90 a 1061.79 0.08194 YES YES  91 a 1077.35 0.10440 YES YES  92 a 1077.95 14.27792 YES YES  93 a 1079.34 59.74741 YES YES  94 a 1081.17 7.89781 YES YES  95 a 1084.41 81.19643 YES YES  96 a 1086.12 8.26353 YES YES  97 a 1089.89 4.31802 YES YES  98 a 1093.47 0.98257 YES YES  99 a 1096.55 0.23499 YES YES  100 a 1096.61 0.04179 YES YES  101 a 1098.15 2.61058 YES YES  102 a 1099.13 0.19744 YES YES  103 a 1099.33 0.40033 YES YES  104 a 1100.32 0.29951 YES YES  105 a 1128.36 6.39966 YES YES  106 a 1129.07 9.96128 YES YES  107 a 1137.25 0.59727 YES YES  108 a 1138.50 6.67605 YES YES  109 a 1172.87 12.37470 YES YES  110 a 1173.28 15.24929 YES YES  111 a 1176.42 40.15927 YES YES  112 a 1184.90 0.01513 YES YES  113 a 1186.14 1.72451 YES YES  114 a 1203.85 14.72351 YES YES  115 a 1211.56 37.41225 YES YES  116 a 1212.32 12.78775 YES YES  117 a 1213.40 42.39661 YES YES  118 a 1227.62 13.45736 YES YES  119 a 1227.73 5.02998 YES YES  120 a 1230.63 2.84961 YES YES  121 a 1291.30 43.51464 YES YES  122 a 1338.21 12.87585 YES YES  123 a 1338.55 19.05926 YES YES  124 a 1341.75 7.08912 YES YES  125 a 1347.96 6.81710 YES YES  126 a 1353.30 0.19565 YES YES  127 a 1369.00 0.05939 YES YES  128 a 1369.87 0.04522 YES YES  129 a 1385.81 7.39015 YES YES  130 a 1391.06 17.50532 YES YES  131 a 1392.09 43.32460 YES YES  132 a 1394.61 32.96548 YES YES  133 a 1396.49 1.32866 YES YES  134 a 1396.94 2.23927 YES YES  135 a 1448.36 0.22543 YES YES  136 a 1519.19 2.20833 YES YES  137 a 1521.02 14.88011 YES YES  138 a 1525.48 2.98350 YES YES  139 a 1525.68 8.02878 YES YES  140 a 1541.56 51.47203 YES YES  141 a 1542.01 93.47941 YES YES  142 a 1552.57 80.17775 YES YES  143 a 1572.84 62.86360 YES YES  144 a 1574.68 19.30192 YES YES  145 a 1603.35 0.51369 YES YES  146 a 1604.13 31.95551 YES YES  147 a 1609.79 7.06902 YES YES  148 a 1621.41 604.59291 YES YES  149 a 1649.62 2.82711 YES YES  150 a 1690.88 185.04373 YES YES  151 a 1701.80 10.37943 YES YES  152 a 1701.93 52.42154 YES YES  153 a 1708.44 7.28307 YES YES  154 a 1714.27 10.68982 YES YES  155 a 1715.23 31.29891 YES YES  156 a 1729.99 29.75247 YES YES  157 a 1730.97 81.73919 YES YES  158 a 1743.16 39.99322 YES YES  159 a 1744.95 38.37900 YES YES  160 a 3301.72 0.32003 YES YES  161 a 3302.24 0.00150 YES YES  162 a 3306.46 24.88327 YES YES  163 a 3307.93 0.56179 YES YES  164 a 3307.95 0.89357 YES YES  165 a 3308.61 3.05320 YES YES  166 a 3308.64 1.15549 YES YES  167 a 3308.67 0.65068 YES YES  168 a 3319.72 6.11310 YES YES  169 a 3319.75 8.39629 YES YES  170 a 3320.31 0.34364 YES YES  171 a 3320.84 5.82417 YES YES  172 a 3321.01 4.85942 YES YES  173 a 3325.32 3.13007 YES YES  174 a 3326.56 0.25878 YES YES  175 a 3328.10 1.00028 YES YES  176 a 3328.17 1.34872 YES YES  177 a 3331.17 0.96294 YES YES  178 a 3331.23 0.03461 YES YES  179 a 3331.71 3.53387 YES YES  180 a 3332.01 2.73807 YES YES  181 a 3335.29 0.15771 YES YES  182 a 3343.04 4.46244 YES YES  183 a 3343.10 3.77669 YES YES |

Optimized atomic coordinates from the DFT calculations (B3LYP/SV(P) level) (Bohr Units):

| 42.00459205162536 22.11662480383663 40.27245974126797 Ga  38.64075260135026 22.79170320596856 42.30692777407606 N  39.42302762355338 19.88675262642867 38.27823094474491 N  38.38837783490145 24.39214411890305 44.27050918316294 C  40.09075870830249 25.40553561623468 44.85312767140517 H  36.10752434133877 24.75808913249740 45.51747374391076 C  35.99099429851780 26.08090168622252 47.09786779115644 H  33.98801828356419 23.39115646152123 44.68888120540846 C  32.15669391777890 23.62214029887074 45.61846238531491 H  34.23947680628975 21.73839650323134 42.66009015003741 C  32.60029028900264 20.68080847887100 41.99417652211490 H  36.59990782799076 21.45990146916491 41.46770911112717 C  37.05707771457463 19.77646480021322 39.30724224015456 C  35.23916999468605 18.11314308037764 38.31468503398622 C  33.35675662214697 18.00402346794268 39.14800836411361 H  35.84713057460241 16.58058315618804 36.26620078806671 C  34.44230097976633 15.28175915080122 35.48576747175166 H  38.28864332930318 16.72124527411106 35.22878836904158 C  38.84560925084547 15.55559413968686 33.61860385803193 H  40.01003866295258 18.38360738333472 36.30123014092143 C  41.93388459084926 18.54294097456136 35.56944961782292 H  43.15677202667110 19.10753762813616 42.46951177153824 N  45.14481353817251 20.96664768284758 38.25105450180914 N  41.95967793127859 18.20690711237489 44.54567143667742 C  40.33457998354692 19.29350759173081 45.20993131036573 H  42.72493952317853 16.04122577061636 45.79467701596472 C  41.70564521517878 15.40094811120017 47.47161941490165 H  44.82719151739867 14.70128285027405 44.84665688155410 C  45.47456909973636 12.96945203017632 45.76887438056779 H  46.05938081447689 15.60431120829912 42.71621132969683 C  47.67256120566837 14.57172465373676 41.95348419484826 H  45.21382634307186 17.83418022400749 41.52710715056763 C  46.37931592264790 18.93176157149108 39.28690482173160 C  48.63398511019027 18.03818257618695 38.18106876456233 C  49.63336260392342 16.43955836765268 39.01520738495146 H  49.60241641450902 19.20453229339390 36.04277121036694 C  51.35376489189859 18.52049567547815 35.18607018143064 H  48.30276445128163 21.28669475093006 34.99954208272151 C  48.99547151085699 22.25540119021299 33.31354069310052 H  46.10697543071788 22.09600178523473 36.16597402367975 C  45.04675892901848 23.70386446371360 35.42204368921522 H  41.37948194837911 25.43524546671074 38.07671661677953 N  44.15395016193879 24.85766707977438 42.23581972511705 N  39.84247505143207 25.57975433020235 36.05852359405601 C  38.89128112927258 23.83342356836056 35.50138953238694 H  39.46423593631997 27.81457823435875 34.72433187052356 C  38.19332998304324 27.84746016158794 33.09765647113286 H  40.74720270889566 29.98729824815347 35.52749058563660 C  40.50572163536831 31.78269346902740 34.53226604261710 H  42.33654667167949 29.84120588207956 37.62256723210461 C  43.32942773624485 31.52712611960869 38.27077210612924 H  42.62563201328422 27.52793483739674 38.89118797103234 C  44.24911966879000 27.18031007716205 41.13407866038710 C  45.82773181607109 29.07206650646368 42.11505283552671 C  45.92388218757500 30.92751677087190 41.22221057544176 H  47.29961215814215 28.57025416641071 44.24107974020590 C  48.53560101881661 30.03288779131762 45.01814226949060 H  47.17646077646812 26.16911079919571 45.35473546586516 C  48.29494235018038 25.69402885624392 47.02385987970157 H  45.58919453582527 24.37107733010757 44.28158348042526 C  45.44329385908696 22.46973153885394 45.07281361548823 H |
| --- |

List of calculated frequencies (B3LYP/SV(P) level):

| mode symmetry wave number IR intensity selection rules  cm**(-1) km/mol IR RAMAN |
| --- |
| 1 0.00 0.00000 - -  2 0.00 0.00000 - -  3 0.00 0.00000 - -  4 0.00 0.00000 - -  5 0.00 0.00000 - -  6 0.00 0.00000 - -  7 a 29.00 20.25137 YES YES  8 a 33.46 9.18945 YES YES  9 a 35.22 3.03938 YES YES  10 a 35.76 5.29113 YES YES  11 a 38.04 0.12120 YES YES  12 a 57.65 0.62701 YES YES  13 a 86.51 0.29484 YES YES  14 a 86.79 23.06445 YES YES  15 a 96.01 0.68816 YES YES  16 a 106.53 287.03351 YES YES  17 a 108.24 70.33324 YES YES  18 a 110.59 216.22576 YES YES  19 a 124.47 4.95867 YES YES  20 a 133.47 22.88069 YES YES  21 a 147.08 1.48732 YES YES  22 a 157.94 371.10117 YES YES  23 a 168.74 10.41719 YES YES  24 a 188.35 46.48210 YES YES  25 a 189.26 12.16695 YES YES  26 a 200.94 101.50178 YES YES  27 a 239.52 164.28416 YES YES  28 a 244.65 9.47805 YES YES  29 a 246.08 34.05917 YES YES  30 a 254.95 0.05399 YES YES  31 a 255.42 14.78514 YES YES  32 a 256.72 115.21326 YES YES  33 a 269.94 1.58736 YES YES  34 a 368.90 14.72519 YES YES  35 a 371.95 7.55004 YES YES  36 a 376.78 2.64934 YES YES  37 a 419.93 103.23370 YES YES  38 a 422.02 5.88975 YES YES  39 a 429.72 14.51637 YES YES  40 a 433.93 5.57785 YES YES  41 a 434.89 1.43816 YES YES  42 a 440.18 1.59877 YES YES  43 a 442.74 16.23858 YES YES  44 a 448.96 4.49982 YES YES  45 a 456.49 29.91879 YES YES  46 a 470.19 10.73497 YES YES  47 a 471.13 3.01845 YES YES  48 a 484.26 3.67221 YES YES  49 a 558.16 3.91590 YES YES  50 a 561.63 6.38635 YES YES  51 a 566.15 0.83610 YES YES  52 a 639.70 66.45489 YES YES  53 a 644.47 23.69479 YES YES  54 a 645.63 12.05885 YES YES  55 a 653.56 493.91158 YES YES  56 a 660.93 4.30373 YES YES  57 a 661.58 16.83140 YES YES  58 a 663.09 2.91073 YES YES  59 a 664.35 78.46124 YES YES  60 a 670.16 2.05819 YES YES  61 a 722.37 65.16859 YES YES  62 a 738.45 31.24369 YES YES  63 a 746.97 61.11493 YES YES  64 a 756.44 1.86205 YES YES  65 a 762.56 1.35076 YES YES  66 a 766.47 0.28192 YES YES  67 a 770.06 218.66223 YES YES  68 a 772.41 328.32174 YES YES  69 a 776.10 24.02122 YES YES  70 a 780.12 1.61553 YES YES  71 a 782.79 54.46764 YES YES  72 a 786.95 51.17233 YES YES  73 a 816.17 2.04861 YES YES  74 a 829.98 2.33955 YES YES  75 a 838.01 2.89005 YES YES  76 a 899.07 63.46890 YES YES  77 a 899.39 23.43516 YES YES  78 a 910.98 258.89960 YES YES  79 a 913.30 9.93244 YES YES  80 a 917.76 417.12511 YES YES  81 a 919.96 114.39670 YES YES  82 a 927.46 4680.04459 YES YES  83 a 958.68 2747.42193 YES YES  84 a 987.05 1.31965 YES YES  85 a 987.91 0.97898 YES YES  86 a 996.48 0.68004 YES YES  87 a 996.97 0.81011 YES YES  88 a 1001.42 0.48512 YES YES  89 a 1002.26 0.83785 YES YES  90 a 1010.36 15.36330 YES YES  91 a 1021.27 0.09732 YES YES  92 a 1023.16 2.43141 YES YES  93 a 1025.45 0.10412 YES YES  94 a 1029.03 0.70857 YES YES  95 a 1030.88 0.12548 YES YES  96 a 1036.55 0.10224 YES YES  97 a 1037.51 0.18538 YES YES  98 a 1042.55 0.09985 YES YES  99 a 1045.47 0.05661 YES YES  100 a 1052.97 9.12494 YES YES  101 a 1056.13 4.85361 YES YES  102 a 1057.85 4.50784 YES YES  103 a 1071.37 2.13969 YES YES  104 a 1073.03 3.74834 YES YES  105 a 1078.71 0.32785 YES YES  106 a 1082.22 2.26000 YES YES  107 a 1083.01 8.01109 YES YES  108 a 1088.77 7.64231 YES YES  109 a 1128.23 157.16649 YES YES  110 a 1129.01 71.68242 YES YES  111 a 1132.86 4.19756 YES YES  112 a 1146.70 29.41796 YES YES  113 a 1148.61 83.75928 YES YES  114 a 1152.96 40.57229 YES YES  115 a 1163.56 1321.42265 YES YES  116 a 1169.57 717.41679 YES YES  117 a 1174.33 49.47324 YES YES  118 a 1175.96 64.27608 YES YES  119 a 1176.63 52.74160 YES YES  120 a 1190.82 2.56065 YES YES  121 a 1256.61 1795.67318 YES YES  122 a 1260.92 790.82020 YES YES  123 a 1290.07 121.00674 YES YES  124 a 1304.40 431.39197 YES YES  125 a 1306.69 735.59686 YES YES  126 a 1308.04 616.56383 YES YES  127 a 1313.06 93.93278 YES YES  128 a 1315.69 894.69718 YES YES  129 a 1345.68 63.83140 YES YES  130 a 1350.03 6.33140 YES YES  131 a 1350.57 10.63556 YES YES  132 a 1351.69 0.86965 YES YES  133 a 1352.72 14.11409 YES YES  134 a 1354.15 23.43512 YES YES  135 a 1363.02 46.31304 YES YES  136 a 1425.85 2458.07471 YES YES  137 a 1429.33 2373.15029 YES YES  138 a 1458.36 20.57435 YES YES  139 a 1460.19 9.10979 YES YES  140 a 1462.34 12.80677 YES YES  141 a 1474.40 55.62915 YES YES  142 a 1478.25 71.76611 YES YES  143 a 1479.90 81.47447 YES YES  144 a 1498.59 113.07849 YES YES  145 a 1501.83 64.33716 YES YES  146 a 1505.11 35.88312 YES YES  147 a 1534.84 0.32231 YES YES  148 a 1576.33 118.49979 YES YES  149 a 1586.84 266.29872 YES YES  150 a 1588.78 65.60134 YES YES  151 a 1601.37 37.04858 YES YES  152 a 1606.89 63.52948 YES YES  153 a 1618.15 35.74889 YES YES  154 a 1620.65 59.75779 YES YES  155 a 1625.40 48.16978 YES YES  156 a 1632.20 80.93868 YES YES  157 a 1634.60 7.18422 YES YES  158 a 1641.90 29.80385 YES YES  159 a 1649.37 38.14700 YES YES  160 a 3201.94 1.79986 YES YES  161 a 3202.72 0.20239 YES YES  162 a 3202.80 0.29008 YES YES  163 a 3202.98 0.00824 YES YES  164 a 3203.99 4.87782 YES YES  165 a 3204.10 1.84186 YES YES  166 a 3209.31 7.34209 YES YES  167 a 3210.08 32.81203 YES YES  168 a 3210.43 11.15861 YES YES  169 a 3211.26 15.61287 YES YES  170 a 3212.95 28.86838 YES YES  171 a 3213.40 14.37364 YES YES  172 a 3217.67 0.70576 YES YES  173 a 3219.30 1.01227 YES YES  174 a 3220.17 0.94879 YES YES  175 a 3222.03 4.49453 YES YES  176 a 3222.90 10.32727 YES YES  177 a 3223.01 2.69353 YES YES  178 a 3223.13 5.30741 YES YES  179 a 3224.34 0.42427 YES YES  180 a 3224.44 4.30320 YES YES  181 a 3229.94 1.13300 YES YES  182 a 3232.02 3.29542 YES YES  183 a 3233.24 4.50005 YES YES |

### [In(phen)_2_]^+^

Optimized atomic coordinates from the DFT calculations (BHLYP/SV(P) level) (Bohr Units):

| 12.35607557141340 26.47753801500417 12.92969515849768 in  16.14903910132540 26.09825105310841 10.00780728390952 n  14.84567265950665 22.19289256685050 13.16565335567267 n  10.64273896926876 29.58268414757863 9.46651431672835 n  10.17161611704054 24.42443313870422 9.17413009719253 n  16.75231798724326 27.93891764747838 8.45482326484134 c  17.77553520145386 24.16522018756856 10.32508325423427 c  14.23564763119276 20.39656454217014 14.76246756449531 c  17.10618469257887 22.12783197687681 12.01597720337372 c  10.80700660637290 32.04524924904000 9.70584519932588 c  9.10048415203319 28.59785927376643 7.70949425388869 c  9.98074162505931 21.95524809834124 9.00214345766866 c  8.88089967233396 25.88156917677135 7.53312171881739 c  19.02302291050641 28.01155055243688 7.09909195494956 c  20.12568807628054 24.08229080175505 9.07627048175231 c  15.82512056495597 18.35118287293513 15.31023804469161 c  18.83855322373501 20.14874381185624 12.42723100409484 c  9.47177373979358 33.73686501049052 8.16767835570847 c  7.68385107167168 30.13341039238530 6.05968337606200 c  8.48672358406825 20.73549526125015 7.19103796529924 c  7.29814934847390 24.83675558782939 5.66429253704327 c  20.71723524546740 26.07811805710918 7.42423823223041 c  21.82519819920119 22.02266212867833 9.51865546454057 c  18.12960946435142 18.23127170530004 14.12770516065952 c  21.21529506342076 20.14254168458214 11.13489159012970 c  7.91411006372049 32.76828275874868 6.33494724870814 c  6.08641188728945 28.99841097463840 4.19448758700205 c  7.13458167049206 22.18787109265114 5.52478636508626 c  5.91232866437355 26.45862043290386 3.99838406910037 c  15.38548882746419 29.45502305129892 8.25432265693369 h  12.40232475127950 20.55772764241242 15.67913784973267 h  12.04723042101290 32.74949718441877 11.18682032754639 h  11.05551506669247 20.85531896403185 10.35928383930449 h  19.41264077508694 29.57895020118292 5.83969109283879 h  15.22690444175620 16.91325760777488 16.64047497955976 h  9.68026771795001 35.75615150347529 8.43975311549197 h  8.40925113538700 18.68954162117448 7.13881172904619 h  22.50975766152994 26.06805167562313 6.42732212902869 h  23.62423749335882 22.01283314075312 8.53615150178895 h  19.41939435863512 16.67891415174036 14.49354046055982 h  22.51582021321384 18.59570685812131 11.47829416429301 h  6.84735437242484 34.00942851142760 5.09849144669930 h  5.01898639290715 30.21965804593341 2.94103253440736 h  5.93667843548373 21.32219195273989 4.10257480142158 h  4.70444828266217 25.60092606214384 2.58156589235088 h |
| --- |

List of calculated frequencies (BHLYP/SV(P) level):

| mode symmetry wave number IR intensity selection rules  cm**(-1) km/mol IR RAMAN |
| --- |
| 1 0.00 0.00000 - -  2 0.00 0.00000 - -  3 0.00 0.00000 - -  4 0.00 0.00000 - -  5 0.00 0.00000 - -  6 0.00 0.00000 - -  7 a 18.05 0.56565 YES YES  8 a 25.55 0.06253 YES YES  9 a 31.70 0.57961 YES YES  10 a 58.52 0.20061 YES YES  11 a 71.92 0.39419 YES YES  12 a 80.78 0.10189 YES YES  13 a 104.74 21.76172 YES YES  14 a 111.41 0.39730 YES YES  15 a 112.01 2.73248 YES YES  16 a 123.84 1.90243 YES YES  17 a 142.84 0.67699 YES YES  18 a 146.82 1.77786 YES YES  19 a 165.94 26.70301 YES YES  20 a 249.09 3.77082 YES YES  21 a 250.92 1.91827 YES YES  22 a 265.79 0.41401 YES YES  23 a 268.61 0.04120 YES YES  24 a 275.77 1.02545 YES YES  25 a 276.20 5.65596 YES YES  26 a 432.77 6.59086 YES YES  27 a 433.23 14.14883 YES YES  28 a 442.46 0.48017 YES YES  29 a 445.05 0.04318 YES YES  30 a 470.67 2.95812 YES YES  31 a 475.72 1.11283 YES YES  32 a 491.60 5.46531 YES YES  33 a 493.69 0.50522 YES YES  34 a 537.67 0.31781 YES YES  35 a 537.95 2.93287 YES YES  36 a 542.05 3.72557 YES YES  37 a 545.99 0.31548 YES YES  38 a 581.96 0.25004 YES YES  39 a 582.24 0.20124 YES YES  40 a 590.87 0.00895 YES YES  41 a 592.82 0.00550 YES YES  42 a 654.86 0.12441 YES YES  43 a 656.28 0.02527 YES YES  44 a 667.20 19.64810 YES YES  45 a 668.65 3.02583 YES YES  46 a 757.74 11.88053 YES YES  47 a 758.20 45.92643 YES YES  48 a 762.77 16.15259 YES YES  49 a 762.81 4.43835 YES YES  50 a 791.69 28.41298 YES YES  51 a 793.40 58.16752 YES YES  52 a 828.16 3.68021 YES YES  53 a 828.87 10.04725 YES YES  54 a 862.98 0.08676 YES YES  55 a 863.96 0.10733 YES YES  56 a 905.37 12.91811 YES YES  57 a 905.42 25.28065 YES YES  58 a 915.32 45.27213 YES YES  59 a 915.59 44.81799 YES YES  60 a 929.25 0.07987 YES YES  61 a 929.75 0.02377 YES YES  62 a 940.74 3.17718 YES YES  63 a 943.00 0.44440 YES YES  64 a 1030.70 0.11463 YES YES  65 a 1031.49 2.73809 YES YES  66 a 1034.35 1.77901 YES YES  67 a 1035.38 0.74804 YES YES  68 a 1064.06 0.04241 YES YES  69 a 1064.47 0.02389 YES YES  70 a 1074.84 0.23116 YES YES  71 a 1075.30 0.23621 YES YES  72 a 1080.38 0.05648 YES YES  73 a 1080.61 0.00619 YES YES  74 a 1092.34 0.37018 YES YES  75 a 1092.69 0.07130 YES YES  76 a 1113.70 1.35724 YES YES  77 a 1115.22 1.29386 YES YES  78 a 1147.76 0.04301 YES YES  79 a 1149.62 0.65102 YES YES  80 a 1162.65 48.88334 YES YES  81 a 1163.37 9.87683 YES YES  82 a 1196.50 30.01963 YES YES  83 a 1196.62 5.97723 YES YES  84 a 1197.71 1.97179 YES YES  85 a 1197.88 6.32465 YES YES  86 a 1269.35 0.04538 YES YES  87 a 1269.44 0.60705 YES YES  88 a 1276.11 11.46871 YES YES  89 a 1278.91 0.15251 YES YES  90 a 1291.71 13.55370 YES YES  91 a 1291.81 4.36861 YES YES  92 a 1337.68 0.66352 YES YES  93 a 1338.26 3.36094 YES YES  94 a 1388.09 0.04395 YES YES  95 a 1388.23 0.07228 YES YES  96 a 1410.15 2.26183 YES YES  97 a 1410.28 6.81460 YES YES  98 a 1431.68 17.48462 YES YES  99 a 1432.97 0.94236 YES YES  100 a 1501.09 2.59601 YES YES  101 a 1501.17 1.99852 YES YES  102 a 1504.70 5.46209 YES YES  103 a 1504.90 1.72050 YES YES  104 a 1523.46 81.11350 YES YES  105 a 1524.60 17.01288 YES YES  106 a 1555.66 0.00533 YES YES  107 a 1556.31 0.58447 YES YES  108 a 1605.03 15.87358 YES YES  109 a 1605.09 6.37411 YES YES  110 a 1641.13 34.35443 YES YES  111 a 1641.88 122.32180 YES YES  112 a 1711.04 53.54703 YES YES  113 a 1712.73 10.45100 YES YES  114 a 1728.73 20.24865 YES YES  115 a 1728.81 14.42249 YES YES  116 a 1744.00 0.63839 YES YES  117 a 1744.59 0.23120 YES YES  118 a 1759.01 23.79886 YES YES  119 a 1759.05 8.76897 YES YES  120 a 3269.44 4.66754 YES YES  121 a 3269.47 1.89065 YES YES  122 a 3288.11 0.12338 YES YES  123 a 3288.11 0.12822 YES YES  124 a 3292.69 1.09113 YES YES  125 a 3292.72 0.76794 YES YES  126 a 3293.27 0.10236 YES YES  127 a 3293.30 0.79050 YES YES  128 a 3301.99 0.91377 YES YES  129 a 3302.23 1.49107 YES YES  130 a 3304.79 4.42384 YES YES  131 a 3304.81 1.80490 YES YES  132 a 3316.85 0.47613 YES YES  133 a 3316.87 0.18800 YES YES  134 a 3318.40 1.13854 YES YES  135 a 3318.44 0.85841 YES YES |

Optimized atomic coordinates from the DFT calculations (B3LYP/SV(P) level) (Bohr Units):

| 12.40067003684563 26.41264670849550 12.75209286248820 In  16.24891403142484 26.04817259723285 9.91319065818196 N  14.86865377410406 22.11649872191644 13.06660416048281 N  10.67855273426734 29.57887794191704 9.33854396733936 N  10.13116219362612 24.39081155742029 9.03735649503655 N  16.89563854402421 27.90108273176444 8.34843083889333 C  17.91166449674350 24.13598068338018 10.33077364416676 C  14.22369296809913 20.29870227885287 14.66780962595562 C  17.20309016562989 22.09238841924982 12.01795793271174 C  10.87784799685939 32.06608087257904 9.57906975549639 C  9.01847145517616 28.61210216879362 7.64783196917352 C  9.90473228042449 21.89842305344643 8.84792785740664 C  8.76027642665282 25.88729986072967 7.46376422167461 C  19.23784826249484 28.00132709970853 7.08946133590556 C  20.34612497049839 24.09363670655692 9.18143654927764 C  15.84390087363801 18.28562268032221 15.31270257665039 C  18.97762064400061 20.13698780573770 12.54138116133695 C  9.46882340199915 33.78668479925298 8.11351796922976 C  7.51202471711947 30.19081031076873 6.07152373927217 C  8.29952174345395 20.70518314308387 7.09265438330464 C  7.05148727280738 24.85861038610721 5.65521555973479 C  20.97223078928357 26.09465104640629 7.52168859990365 C  22.07213288210436 22.07000488432582 9.73364953576297 C  18.22565443736564 18.20759310612122 14.23595758923327 C  21.42152700415862 20.17608939518641 11.35872621139799 C  7.78743928689301 32.83781701948578 6.34982513289597 C  5.79822142455400 29.07697105913556 4.28409347823744 C  6.85968801645858 22.19583248470934 5.50050559662647 C  5.58804932615298 26.51499411242818 4.07599481541124 C  15.49516981428746 29.39748159391711 8.06746007326354 H  12.32995627154293 20.42755041683402 15.50258052183908 H  12.21376515122586 32.75512496891412 11.00747079417100 H  11.04844194673530 20.77007080379411 10.15103299364554 H  19.65531062545670 29.57142282052216 5.81428264220709 H  15.21028046062525 16.83167657625194 16.63580297494661 H  9.71692293730834 35.81944649037207 8.38250232151540 H  8.20324966215279 18.64334776630830 7.02093104725574 H  22.82396351287840 26.11339060188932 6.60071213561691 H  23.93180065365746 22.08767763455121 8.83129600252141 H  19.54154109587280 16.67581659004694 14.68287275909606 H  22.75214132915140 18.65256001276986 11.78474678764796 H  6.65895658213234 34.10798063512561 5.17047233972646 H  4.66429274831952 30.32563752699339 3.08903590898731 H  5.57097058675271 21.34506618703482 4.12471204179795 H  4.28548757650966 25.67537611262160 2.70804451928160 H |
| --- |

List of calculated frequencies (B3LYP/SV(P) level):

| mode symmetry wave number IR intensity selection rules  cm**(-1) km/mol IR RAMAN |
| --- |
| 1 -0.00 0.00000 - -  2 -0.00 0.00000 - -  3 0.00 0.00000 - -  4 0.00 0.00000 - -  5 0.00 0.00000 - -  6 0.00 0.00000 - -  7 a 17.57 0.65012 YES YES  8 a 24.27 0.08248 YES YES  9 a 30.40 0.60903 YES YES  10 a 56.62 0.41689 YES YES  11 a 67.99 0.28664 YES YES  12 a 76.95 0.05909 YES YES  13 a 103.48 17.31812 YES YES  14 a 104.60 0.01181 YES YES  15 a 109.83 1.12095 YES YES  16 a 120.74 4.93622 YES YES  17 a 137.05 0.74313 YES YES  18 a 142.51 0.91253 YES YES  19 a 162.36 22.89349 YES YES  20 a 238.15 4.06245 YES YES  21 a 239.98 1.35237 YES YES  22 a 255.45 0.38607 YES YES  23 a 258.15 0.06868 YES YES  24 a 267.69 1.62425 YES YES  25 a 267.73 4.73388 YES YES  26 a 418.07 4.01745 YES YES  27 a 419.04 11.09587 YES YES  28 a 424.53 0.57037 YES YES  29 a 427.06 0.04670 YES YES  30 a 452.06 4.03537 YES YES  31 a 457.05 1.06249 YES YES  32 a 475.18 3.99922 YES YES  33 a 476.91 0.43713 YES YES  34 a 520.96 0.43155 YES YES  35 a 521.32 2.79011 YES YES  36 a 521.73 6.56586 YES YES  37 a 526.64 0.47243 YES YES  38 a 563.49 0.13441 YES YES  39 a 563.73 0.05192 YES YES  40 a 569.97 0.02960 YES YES  41 a 571.88 0.01645 YES YES  42 a 629.84 0.07901 YES YES  43 a 631.04 0.02834 YES YES  44 a 643.65 15.54757 YES YES  45 a 645.15 2.45883 YES YES  46 a 727.04 6.85674 YES YES  47 a 728.35 35.56551 YES YES  48 a 738.16 13.15997 YES YES  49 a 738.23 3.20841 YES YES  50 a 763.46 21.84540 YES YES  51 a 764.64 49.14225 YES YES  52 a 795.58 3.39508 YES YES  53 a 796.26 11.49935 YES YES  54 a 827.78 0.02113 YES YES  55 a 828.88 0.11636 YES YES  56 a 874.12 3.95956 YES YES  57 a 874.26 21.03635 YES YES  58 a 882.34 42.77377 YES YES  59 a 882.72 36.13162 YES YES  60 a 899.50 0.07728 YES YES  61 a 899.95 0.04052 YES YES  62 a 905.38 2.04303 YES YES  63 a 907.24 0.35890 YES YES  64 a 979.81 0.13553 YES YES  65 a 980.21 3.43656 YES YES  66 a 983.87 1.68186 YES YES  67 a 984.62 0.86728 YES YES  68 a 1017.13 0.03259 YES YES  69 a 1017.57 0.02891 YES YES  70 a 1025.71 0.66685 YES YES  71 a 1026.13 0.25480 YES YES  72 a 1033.00 0.05217 YES YES  73 a 1033.17 0.00104 YES YES  74 a 1053.46 0.31322 YES YES  75 a 1053.97 0.02870 YES YES  76 a 1076.89 1.91483 YES YES  77 a 1078.13 1.12459 YES YES  78 a 1106.34 0.00709 YES YES  79 a 1108.31 0.71671 YES YES  80 a 1119.80 41.72375 YES YES  81 a 1120.32 6.04303 YES YES  82 a 1156.33 24.12722 YES YES  83 a 1156.40 4.22540 YES YES  84 a 1164.54 0.48004 YES YES  85 a 1164.56 2.16912 YES YES  86 a 1226.96 3.58619 YES YES  87 a 1228.32 0.19699 YES YES  88 a 1242.12 2.77744 YES YES  89 a 1243.03 0.09181 YES YES  90 a 1248.34 3.99381 YES YES  91 a 1248.41 9.30059 YES YES  92 a 1288.82 0.50426 YES YES  93 a 1289.64 2.22694 YES YES  94 a 1349.34 5.00016 YES YES  95 a 1349.56 0.53991 YES YES  96 a 1373.54 9.13615 YES YES  97 a 1373.88 0.45621 YES YES  98 a 1379.40 9.11397 YES YES  99 a 1380.18 0.79197 YES YES  100 a 1440.08 1.97710 YES YES  101 a 1440.25 0.49059 YES YES  102 a 1446.80 2.42962 YES YES  103 a 1446.91 0.79065 YES YES  104 a 1460.28 77.30783 YES YES  105 a 1461.27 16.66321 YES YES  106 a 1489.10 0.01989 YES YES  107 a 1490.31 0.33389 YES YES  108 a 1540.67 17.58444 YES YES  109 a 1540.83 4.59573 YES YES  110 a 1563.03 15.56229 YES YES  111 a 1564.23 72.84455 YES YES  112 a 1622.87 31.14685 YES YES  113 a 1624.36 5.95175 YES YES  114 a 1645.28 7.53064 YES YES  115 a 1645.46 1.94608 YES YES  116 a 1658.95 0.15529 YES YES  117 a 1659.47 0.19204 YES YES  118 a 1674.18 26.35583 YES YES  119 a 1674.26 7.41749 YES YES  120 a 3159.58 6.04413 YES YES  121 a 3159.67 2.72562 YES YES  122 a 3181.69 0.02088 YES YES  123 a 3181.73 0.02049 YES YES  124 a 3185.80 1.55116 YES YES  125 a 3185.87 1.92980 YES YES  126 a 3186.62 0.66148 YES YES  127 a 3186.65 0.77192 YES YES  128 a 3191.00 0.63684 YES YES  129 a 3191.16 1.14142 YES YES  130 a 3197.47 6.94145 YES YES  131 a 3197.51 3.65856 YES YES  132 a 3209.44 2.10202 YES YES  133 a 3209.46 0.43044 YES YES  134 a 3210.55 2.76526 YES YES  135 a 3210.57 0.87646 YES YES |

### [Ag(C_6_H_5_F)]^+^

Optimized atomic coordinates from the DFT calculations (BHLYP/SV(P) level) (Bohr Units):

| 0.22076026874804 -3.03201697165817 0.22994621618120 Ag  2.05072265842924 1.21246221564249 -2.39229566849202 C  -0.43870938901301 -1.24993844183347 -4.52597672987881 H  -2.50130581806181 0.97070645503784 -1.76180935792339 C  -0.29152415778624 0.06494612243591 -2.95205327732447 C  -4.32869543241157 0.18383377566428 -2.26340274757327 H  -2.36871770559452 2.96219808359218 -0.06125423823078 C  -0.03706086972638 4.06510711901145 0.42445653132098 C  -4.04420373641165 3.71418953304716 0.84735152338642 H  0.08052523292055 5.96595462246270 1.99467109710487 F  2.17637430401431 3.20696810711563 -0.69817338861002 C  3.74676602451511 0.60318240099364 -3.37231651103089 H  3.94716210856578 4.14429482537050 -0.26949224693648 h |
| --- |

List of calculated frequencies (BHLYP/SV(P) level):

| mode symmetry wave number IR intensity selection rules  cm**(-1) km/mol IR RAMAN |
| --- |
| 1 -0.00 0.00000 - -  2 0.00 0.00000 - -  3 0.00 0.00000 - -  4 0.00 0.00000 - -  5 0.00 0.00000 - -  6 0.00 0.00000 - -  7 a 37.05 0.25189 YES YES  8 a 52.61 0.49048 YES YES  9 a 175.76 1.47560 YES YES  10 a 296.25 6.24025 YES YES  11 a 433.37 3.86863 YES YES  12 a 447.27 0.28947 YES YES  13 a 537.01 7.31698 YES YES  14 a 554.49 7.03193 YES YES  15 a 638.85 0.17814 YES YES  16 a 730.96 5.88579 YES YES  17 a 860.14 80.87568 YES YES  18 a 869.88 55.90202 YES YES  19 a 903.90 0.61820 YES YES  20 a 979.44 16.86339 YES YES  21 a 1029.69 20.56503 YES YES  22 a 1055.86 1.58356 YES YES  23 a 1058.32 6.30664 YES YES  24 a 1066.62 0.37194 YES YES  25 a 1117.79 5.30740 YES YES  26 a 1199.26 0.29641 YES YES  27 a 1210.29 12.93621 YES YES  28 a 1368.62 1.69641 YES YES  29 a 1375.07 96.27728 YES YES  30 a 1411.44 0.13448 YES YES  31 a 1539.49 9.83372 YES YES  32 a 1590.58 98.79584 YES YES  33 a 1687.75 5.89859 YES YES  34 a 1724.50 197.22938 YES YES  35 a 3261.31 3.94008 YES YES  36 a 3297.36 1.80564 YES YES  37 a 3299.77 1.94012 YES YES  38 a 3318.10 10.12176 YES YES  39 a 3318.80 4.06268 YES YES |

Optimized atomic coordinates from the DFT calculations (B3LYP/SV(P) level) (Bohr Units):

| 0.24949274056844 -3.21399788718756 0.00626478116151 Ag  2.07619538349175 1.19410776544729 -2.35126967580789 C  -0.45037905959974 -1.25952569434830 -4.52082479334383 H  -2.52637400684111 0.96103346371689 -1.71454341093318 C  -0.28945568933887 0.01314157480575 -2.88972482591706 C  -4.36718866024809 0.15892097677815 -2.21120520927255 H  -2.39401818096015 2.99928674108186 -0.05127233429394 C  -0.03947618676320 4.11712272097528 0.42201505896807 C  -4.08305275807209 3.78047523052350 0.84523215339075 H  0.07476937813601 6.07025454522452 1.96178279076791 F  2.19747292502538 3.23231933010816 -0.68815736002131 C  3.78417440315726 0.56915425370857 -3.33629518831224 H  3.97993319963228 4.18959482604807 -0.27235078439285 H |
| --- |

List of calculated frequencies (B3LYP/SV(P) level):

| mode symmetry wave number IR intensity selection rules  cm**(-1) km/mol IR RAMAN |
| --- |
| 1 -0.00 0.00000 - -  2 -0.00 0.00000 - -  3 -0.00 0.00000 - -  4 0.00 0.00000 - -  5 0.00 0.00000 - -  6 0.00 0.00000 - -  7 a 50.33 0.21445 YES YES  8 a 56.94 0.10148 YES YES  9 a 172.12 0.37301 YES YES  10 a 299.81 5.87991 YES YES  11 a 419.08 3.57920 YES YES  12 a 427.38 0.23625 YES YES  13 a 517.10 11.49966 YES YES  14 a 532.48 4.92037 YES YES  15 a 614.88 0.21009 YES YES  16 a 705.94 6.06986 YES YES  17 a 828.97 46.46862 YES YES  18 a 839.51 66.80095 YES YES  19 a 859.59 0.36588 YES YES  20 a 934.50 26.68837 YES YES  21 a 985.74 24.64804 YES YES  22 a 1007.38 1.20602 YES YES  23 a 1015.84 2.49816 YES YES  24 a 1021.39 0.74790 YES YES  25 a 1076.78 5.15067 YES YES  26 a 1162.70 0.50970 YES YES  27 a 1171.44 19.21368 YES YES  28 a 1322.33 98.80018 YES YES  29 a 1324.45 1.62917 YES YES  30 a 1389.13 0.38831 YES YES  31 a 1482.80 11.31697 YES YES  32 a 1522.59 87.40891 YES YES  33 a 1599.84 5.45061 YES YES  34 a 1646.88 225.25466 YES YES  35 a 3145.54 5.31358 YES YES  36 a 3194.65 1.91725 YES YES  37 a 3195.43 1.42840 YES YES  38 a 3215.53 9.22232 YES YES  39 a 3216.08 3.34033 YES YES |

### [(phen)_2_In–Ag(C_6_H_5_F)]^2+^

Optimized atomic coordinates from the DFT calculations (BHLYP/SV(P) level) (Bohr Units):

| 0.11192569129215 0.33921194842718 -0.36497266097806 In  0.81452146526946 1.38198665443384 4.45298434384997 Ag  3.81962677055720 -1.82513857460126 -1.68474104410113 N  1.55359412516590 2.14524852497913 -4.07232804163355 N  -3.94487877768562 1.99036082795538 -1.37435178097122 N  -2.18459088251006 -2.73813821478123 -2.44838627414077 N  4.93984863464159 -3.65159579776895 -0.41891156519410 C  4.94253111194844 -0.86554917303825 -3.75485617967069 C  0.45464910401198 4.06208213242732 -5.22937577854467 C  3.73541481344421 1.21287200090189 -5.02691316798705 C  -4.78137182234827 4.24104870457559 -0.71975762427131 C  -5.54294999647611 0.32328605898452 -2.43987111437657 C  -1.32841261345882 -5.01936108677828 -2.98120075830944 C  -4.60767555465638 -2.16152906312286 -3.02856648714444 C  3.99142147985867 -4.32808236369164 1.27392903285904 H  7.24398911361213 -4.70687201111987 -1.17053268998817 C  7.25541628140144 -1.80267206551349 -4.66886243391982 C  1.43588083148465 5.18894565572427 -7.40601406119468 C  -1.29057875597044 4.75990848258709 -4.41621804328443 H  4.87501615684057 2.23345611174858 -7.19937892092794 C  -3.43900629121765 5.50585092052340 0.18643959113884 H  -7.27152765263864 5.02042408415105 -1.14277517924688 C  -8.08033342996149 0.92768190897171 -2.94990612871903 C  -2.80704378195819 -6.88672426766439 -4.11979052069221 C  0.62063582136710 -5.41600940076004 -2.49380978386692 H  -6.24532344585472 -3.92341107307428 -4.15730734850229 C  8.08409045995962 -6.21696431155361 -0.07134608579588 H  8.39301751159636 -3.78633708287057 -3.30866958467939 C  8.35733178535300 -0.72376988545660 -6.89097790273403 C  3.65305864070138 4.27273487595366 -8.39100862434112 C  0.44685632696124 6.75937054680737 -8.27110567757618 H  7.20834338080959 1.20637240435957 -8.10881837042218 C  -7.86460257819411 6.89835659440847 -0.58060089045710 H  -8.91737338392970 3.36040049463114 -2.27172456076091 C  -9.69367585803873 -0.91178936557798 -4.10303826411872 C  -5.27313581451076 -6.33732245926879 -4.70446480477021 C  -1.99657114721283 -8.72580627541375 -4.51170596845316 H  -8.80808084389873 -3.23526765982767 -4.69087394311133 C  10.17938095992499 -4.56132043601064 -3.95311852445947 H  10.13470523217140 -1.47997287482271 -7.57585964698996 H  4.47786999438592 5.10623768811426 -10.07375107741203 H  8.04695585760232 2.02434980699747 -9.79034588561278 H  -10.86319477753595 3.89866300530642 -2.63361868812923 H  -11.64160651117423 -0.40770190847460 -4.49213288882640 H  -6.48325346148072 -7.74370991404043 -5.57868601288020 H  -10.03261932362752 -4.62754420216319 -5.56376209752295 H  3.45210044631143 0.85846469506571 9.17889031271246 C  2.24920961668456 4.68070643061902 8.38346269111004 H  -0.88678680665810 2.28500534545783 9.44724406704759 C  1.65060221404340 2.77576766473756 8.86146807291131 C  -2.28056395649332 3.78218271652595 9.29930032252359 H  -1.61611680908796 -0.08089604190204 10.32166871609804 C  0.20862662671751 -1.93703354762717 10.62063638914455 C  -3.55956809718828 -0.49294402537823 10.82168301295394 H  -0.47287598161751 -4.17192126052719 11.45610584215781 F  2.73215338468469 -1.50761334947015 10.05571702321821 C  5.43288602888633 1.24599743457600 8.81590645315119 H  4.09621663942606 -3.00608309944069 10.35769435848889 H |
| --- |

List of calculated frequencies (BHLYP/SV(P) level):

| mode symmetry wave number IR intensity selection rules  cm**(-1) km/mol IR RAMAN |
| --- |
| 1 -0.00 0.00000 - -  2 -0.00 0.00000 - -  3 0.00 0.00000 - -  4 0.00 0.00000 - -  5 0.00 0.00000 - -  6 0.00 0.00000 - -  7 a 8.25 0.09365 YES YES  8 a 14.19 0.26655 YES YES  9 a 19.34 0.27971 YES YES  10 a 20.60 0.34409 YES YES  11 a 29.58 0.17380 YES YES  12 a 31.46 0.00768 YES YES  13 a 36.63 0.82858 YES YES  14 a 41.03 0.10013 YES YES  15 a 51.47 0.23908 YES YES  16 a 58.63 0.27908 YES YES  17 a 84.05 0.05358 YES YES  18 a 85.29 0.00973 YES YES  19 a 106.08 2.50418 YES YES  20 a 110.81 0.15707 YES YES  21 a 146.01 1.96134 YES YES  22 a 149.20 6.54544 YES YES  23 a 155.86 14.71132 YES YES  24 a 159.19 0.65683 YES YES  25 a 163.36 4.17414 YES YES  26 a 165.91 24.20067 YES YES  27 a 166.39 14.91030 YES YES  28 a 222.40 24.85640 YES YES  29 a 249.09 4.10344 YES YES  30 a 251.37 2.11057 YES YES  31 a 272.30 0.13150 YES YES  32 a 274.24 1.78491 YES YES  33 a 274.60 2.85708 YES YES  34 a 299.43 8.35010 YES YES  35 a 300.88 1.69759 YES YES  36 a 431.40 3.06016 YES YES  37 a 440.16 12.03332 YES YES  38 a 440.34 9.15073 YES YES  39 a 443.85 1.79019 YES YES  40 a 446.07 1.22305 YES YES  41 a 446.73 0.09811 YES YES  42 a 472.36 0.29622 YES YES  43 a 476.66 3.06089 YES YES  44 a 500.05 0.08368 YES YES  45 a 501.23 1.72004 YES YES  46 a 538.08 0.17190 YES YES  47 a 538.73 3.15853 YES YES  48 a 540.49 4.62794 YES YES  49 a 547.51 0.54199 YES YES  50 a 549.50 15.52646 YES YES  51 a 550.95 0.50819 YES YES  52 a 584.39 0.05298 YES YES  53 a 584.45 0.09135 YES YES  54 a 586.10 0.02352 YES YES  55 a 588.53 0.00210 YES YES  56 a 643.04 0.16468 YES YES  57 a 658.03 0.24682 YES YES  58 a 659.08 0.05069 YES YES  59 a 677.30 3.98067 YES YES  60 a 677.40 10.67068 YES YES  61 a 733.15 8.32841 YES YES  62 a 761.79 32.22232 YES YES  63 a 762.05 2.20853 YES YES  64 a 766.84 53.52874 YES YES  65 a 767.89 26.46476 YES YES  66 a 785.74 32.72308 YES YES  67 a 786.97 76.80419 YES YES  68 a 834.75 1.51671 YES YES  69 a 835.53 6.26576 YES YES  70 a 843.42 161.91123 YES YES  71 a 864.47 0.34903 YES YES  72 a 865.77 0.34660 YES YES  73 a 866.50 18.10906 YES YES  74 a 899.77 0.17688 YES YES  75 a 911.14 37.99495 YES YES  76 a 911.39 14.12397 YES YES  77 a 919.53 46.38763 YES YES  78 a 919.93 46.29867 YES YES  79 a 924.46 0.03645 YES YES  80 a 924.99 0.21168 YES YES  81 a 950.92 1.26105 YES YES  82 a 951.02 5.89449 YES YES  83 a 977.12 1.62558 YES YES  84 a 1033.55 0.23978 YES YES  85 a 1035.77 5.01438 YES YES  86 a 1037.33 0.13523 YES YES  87 a 1039.58 0.82179 YES YES  88 a 1040.42 14.37292 YES YES  89 a 1051.92 0.19044 YES YES  90 a 1065.97 10.43727 YES YES  91 a 1067.44 0.66249 YES YES  92 a 1070.50 0.04729 YES YES  93 a 1070.94 0.02508 YES YES  94 a 1083.26 0.34985 YES YES  95 a 1083.64 0.22777 YES YES  96 a 1088.78 0.04541 YES YES  97 a 1088.98 0.01354 YES YES  98 a 1094.11 0.48934 YES YES  99 a 1094.37 0.65980 YES YES  100 a 1121.67 3.49114 YES YES  101 a 1123.77 2.63448 YES YES  102 a 1126.59 2.67628 YES YES  103 a 1154.84 0.30653 YES YES  104 a 1157.82 0.38512 YES YES  105 a 1167.22 50.21342 YES YES  106 a 1168.24 16.23498 YES YES  107 a 1201.29 0.28946 YES YES  108 a 1202.30 29.26009 YES YES  109 a 1202.33 7.92354 YES YES  110 a 1204.22 6.54162 YES YES  111 a 1204.36 7.72452 YES YES  112 a 1209.06 7.57031 YES YES  113 a 1273.08 2.59956 YES YES  114 a 1273.59 0.55202 YES YES  115 a 1279.42 8.63573 YES YES  116 a 1282.45 1.90387 YES YES  117 a 1293.48 1.79796 YES YES  118 a 1293.74 18.42253 YES YES  119 a 1331.20 0.07634 YES YES  120 a 1333.23 3.16800 YES YES  121 a 1363.20 107.09667 YES YES  122 a 1366.88 1.64510 YES YES  123 a 1396.37 0.47185 YES YES  124 a 1396.65 1.96773 YES YES  125 a 1402.31 0.04060 YES YES  126 a 1409.47 6.24439 YES YES  127 a 1409.69 0.39818 YES YES  128 a 1427.23 20.31779 YES YES  129 a 1429.41 4.25895 YES YES  130 a 1502.69 0.61684 YES YES  131 a 1502.81 0.89744 YES YES  132 a 1511.25 18.09701 YES YES  133 a 1511.56 7.42852 YES YES  134 a 1522.86 32.47856 YES YES  135 a 1523.11 101.98572 YES YES  136 a 1543.59 6.34877 YES YES  137 a 1555.33 2.53161 YES YES  138 a 1555.44 1.12566 YES YES  139 a 1596.45 96.13417 YES YES  140 a 1601.88 4.43780 YES YES  141 a 1602.14 18.99369 YES YES  142 a 1646.12 147.33998 YES YES  143 a 1646.52 57.93368 YES YES  144 a 1707.77 6.78703 YES YES  145 a 1714.77 59.45016 YES YES  146 a 1716.51 10.77960 YES YES  147 a 1723.12 36.96382 YES YES  148 a 1723.14 9.61558 YES YES  149 a 1732.52 122.09564 YES YES  150 a 1738.13 0.05209 YES YES  151 a 1738.83 4.70251 YES YES  152 a 1758.94 31.21014 YES YES  153 a 1758.99 22.03887 YES YES  154 a 3274.85 1.11440 YES YES  155 a 3277.79 0.87489 YES YES  156 a 3278.03 0.79494 YES YES  157 a 3297.04 0.76549 YES YES  158 a 3297.06 0.88375 YES YES  159 a 3298.79 0.08462 YES YES  160 a 3300.31 0.48180 YES YES  161 a 3300.40 0.43326 YES YES  162 a 3300.43 0.25304 YES YES  163 a 3301.14 0.15323 YES YES  164 a 3301.23 0.15069 YES YES  165 a 3312.04 0.33944 YES YES  166 a 3312.06 0.29948 YES YES  167 a 3316.55 2.92046 YES YES  168 a 3317.50 1.05596 YES YES  169 a 3318.65 4.03367 YES YES  170 a 3318.87 4.17781 YES YES  171 a 3321.52 3.55388 YES YES  172 a 3321.54 2.12191 YES YES  173 a 3330.52 0.39584 YES YES  174 a 3330.90 0.46334 YES YES |

Optimized atomic coordinates from the DFT calculations (B3LYP/SV(P) level) (Bohr Units):

| 0.08684054749177 0.30869614149306 -0.43782686784244 In  0.78501479895553 1.35201960320950 4.35514750886730 Ag  3.82424018102438 -1.85305712549057 -1.71634033671933 N  1.58516021060743 2.17863816588394 -4.10574128788013 N  -3.98255804232671 1.98398522932543 -1.40407463812032 N  -2.23598741574573 -2.78784940696164 -2.48428439134899 N  4.93863877069167 -3.71536404211436 -0.44488070117496 C  4.99365073377675 -0.85275933456715 -3.76760998748017 C  0.49906180678089 4.13444947565279 -5.26765955620617 C  3.80370524589310 1.25207634543935 -5.03819798685653 C  -4.81945450400277 4.26229918903020 -0.74238495923140 C  -5.61242959376706 0.29820385505003 -2.44378281856507 C  -1.38566796640244 -5.09885689788858 -3.02531339655594 C  -4.68656718516525 -2.20259078090336 -3.03604923059314 C  3.95205250134080 -4.41978151707670 1.23548109859327 H  7.27295452604530 -4.75926656425075 -1.17724425510301 C  7.35078942837925 -1.78482126592201 -4.65893490408591 C  1.53251125646168 5.29773499058520 -7.42056506191671 C  -1.27582688645543 4.82438336214269 -4.46825194637699 H  4.99565817265890 2.31992928272433 -7.19620811293746 C  -3.45062165340069 5.53808376405704 0.14752846865518 H  -7.33213196654243 5.04133110130357 -1.13529417101031 C  -8.18459739050736 0.91149587389182 -2.92145797654108 C  -2.89853815816896 -6.97631717536848 -4.13924129537708 C  0.58597464230200 -5.49811859989286 -2.55896152855415 H  -6.36051898717590 -3.98920026876190 -4.14266028101288 C  8.10289419708682 -6.29667161653830 -0.07620692537920 H  8.47108524761619 -3.79975455559716 -3.29763282403668 C  8.49447569919751 -0.66539956420108 -6.85236018974290 C  3.78720467237957 4.38970860503865 -8.38582438060847 C  0.55295896775395 6.89440951431995 -8.28908455688598 H  7.35589183879559 1.30128198220376 -8.07592737533737 C  -7.92076995719665 6.93830474295798 -0.57002984733773 H  -9.01194434925714 3.36448981431548 -2.23893007069358 C  -9.82061524105137 -0.94157801817008 -4.04473383604706 C  -5.39323820823724 -6.42197097386876 -4.69435015378779 C  -2.09236564722729 -8.83468884817833 -4.53837018021318 H  -8.93899497039199 -3.29284254527176 -4.64069738302672 C  10.28451566432916 -4.57127507489348 -3.92468539469201 H  10.30064512874030 -1.41371885602284 -7.52190686139556 H  4.65010498940602 5.25737422832578 -10.05292835299501 H  8.23587282589977 2.14971194478346 -9.74208953477314 H  -10.97826720418364 3.90890350978385 -2.57605706972652 H  -11.79041826289961 -0.43544914764175 -4.41069200092544 H  -6.62790533220604 -7.84348599858448 -5.54925247241273 H  -10.19180840081897 -4.69681590619151 -5.49465814961339 H  3.48073566819546 0.83215302974394 9.11255568640962 C  2.25087777758504 4.64463728683544 8.15779848711522 H  -0.89492073708643 2.27385150985704 9.36695272940482 C  1.65378450236288 2.74075733610600 8.71093484694916 C  -2.30450072548419 3.77338332810266 9.16611287003453 H  -1.60953643540121 -0.05938303967074 10.38372645637599 C  0.23986883373683 -1.90748627119392 10.75912630857879 C  -3.56011357950626 -0.45146279038661 10.93793782926808 H  -0.42854982958948 -4.11580742917981 11.73915848487343 F  2.77517728501808 -1.49949274297411 10.13272332219200 C  5.47187781282967 1.20919260916574 8.70251019279682 H  4.15878284858685 -2.98832653535521 10.50038210365338 H |
| --- |

List of calculated frequencies (B3LYP/SV(P) level):

| mode symmetry wave number IR intensity selection rules  cm**(-1) km/mol IR RAMAN |
| --- |
| 1 -0.00 0.00000 - -  2 -0.00 0.00000 - -  3 -0.00 0.00000 - -  4 0.00 0.00000 - -  5 0.00 0.00000 - -  6 0.00 0.00000 - -  7 a 7.72 0.06099 YES YES  8 a 14.03 0.21571 YES YES  9 a 19.54 0.42797 YES YES  10 a 20.61 0.23137 YES YES  11 a 29.22 0.10150 YES YES  12 a 30.14 0.00401 YES YES  13 a 37.01 0.79662 YES YES  14 a 40.03 0.16865 YES YES  15 a 52.02 0.38359 YES YES  16 a 59.85 0.47511 YES YES  17 a 81.68 0.02002 YES YES  18 a 82.98 0.00245 YES YES  19 a 104.27 2.34558 YES YES  20 a 108.61 0.11005 YES YES  21 a 141.77 1.65524 YES YES  22 a 144.73 4.81977 YES YES  23 a 150.87 9.96520 YES YES  24 a 154.08 0.33721 YES YES  25 a 159.00 6.87675 YES YES  26 a 161.42 27.76851 YES YES  27 a 162.19 14.32659 YES YES  28 a 217.65 23.28587 YES YES  29 a 238.63 4.61259 YES YES  30 a 240.95 1.81362 YES YES  31 a 262.18 0.10803 YES YES  32 a 264.02 0.20272 YES YES  33 a 267.10 6.28257 YES YES  34 a 290.58 8.12350 YES YES  35 a 292.23 1.39886 YES YES  36 a 416.12 2.59252 YES YES  37 a 425.13 4.81483 YES YES  38 a 425.26 5.26693 YES YES  39 a 427.04 7.40088 YES YES  40 a 428.24 0.09133 YES YES  41 a 428.80 2.91348 YES YES  42 a 454.54 0.24426 YES YES  43 a 458.69 3.52544 YES YES  44 a 483.00 0.12731 YES YES  45 a 484.35 1.18438 YES YES  46 a 521.45 5.95422 YES YES  47 a 521.49 2.26174 YES YES  48 a 522.14 2.74099 YES YES  49 a 526.76 12.51997 YES YES  50 a 527.79 0.76283 YES YES  51 a 531.35 2.44970 YES YES  52 a 565.57 0.08561 YES YES  53 a 566.05 0.01315 YES YES  54 a 566.46 0.13397 YES YES  55 a 568.43 0.00162 YES YES  56 a 620.91 0.19303 YES YES  57 a 633.04 0.24653 YES YES  58 a 633.93 0.06303 YES YES  59 a 653.77 11.11480 YES YES  60 a 653.94 0.75308 YES YES  61 a 705.82 6.51263 YES YES  62 a 736.30 49.65487 YES YES  63 a 737.18 2.05816 YES YES  64 a 738.43 24.91984 YES YES  65 a 738.52 23.82957 YES YES  66 a 757.69 26.89255 YES YES  67 a 758.51 64.58975 YES YES  68 a 802.25 1.55697 YES YES  69 a 803.13 7.47776 YES YES  70 a 810.17 156.12575 YES YES  71 a 829.55 0.28133 YES YES  72 a 831.05 0.03790 YES YES  73 a 834.06 15.97814 YES YES  74 a 858.60 0.19433 YES YES  75 a 880.02 29.83914 YES YES  76 a 880.14 10.77278 YES YES  77 a 886.56 41.75476 YES YES  78 a 887.12 39.51162 YES YES  79 a 895.39 0.01873 YES YES  80 a 895.92 0.12729 YES YES  81 a 915.32 0.53637 YES YES  82 a 915.49 4.55858 YES YES  83 a 931.69 2.94060 YES YES  84 a 981.74 0.20949 YES YES  85 a 983.66 5.50109 YES YES  86 a 985.69 0.01493 YES YES  87 a 987.61 0.82486 YES YES  88 a 998.41 16.40202 YES YES  89 a 1002.14 1.20706 YES YES  90 a 1021.38 0.33829 YES YES  91 a 1024.82 0.04720 YES YES  92 a 1025.23 0.03685 YES YES  93 a 1026.44 11.50131 YES YES  94 a 1035.54 0.85534 YES YES  95 a 1035.85 0.41017 YES YES  96 a 1042.81 0.03860 YES YES  97 a 1042.95 0.00137 YES YES  98 a 1054.70 0.33270 YES YES  99 a 1054.80 0.43182 YES YES  100 a 1083.06 3.53836 YES YES  101 a 1085.63 3.67499 YES YES  102 a 1087.92 2.92028 YES YES  103 a 1113.47 0.34725 YES YES  104 a 1116.43 0.37610 YES YES  105 a 1125.19 48.31199 YES YES  106 a 1126.16 14.21872 YES YES  107 a 1162.48 17.86424 YES YES  108 a 1162.53 12.70429 YES YES  109 a 1165.39 0.56067 YES YES  110 a 1169.20 10.43009 YES YES  111 a 1170.36 3.70952 YES YES  112 a 1170.46 0.86364 YES YES  113 a 1231.72 4.94481 YES YES  114 a 1234.21 0.39429 YES YES  115 a 1244.75 2.59777 YES YES  116 a 1245.52 1.54263 YES YES  117 a 1250.56 0.94946 YES YES  118 a 1250.95 13.92248 YES YES  119 a 1284.42 0.06753 YES YES  120 a 1286.50 1.84644 YES YES  121 a 1305.97 113.80311 YES YES  122 a 1324.26 0.89499 YES YES  123 a 1359.11 0.14472 YES YES  124 a 1359.41 0.23256 YES YES  125 a 1370.37 16.92769 YES YES  126 a 1372.05 1.19992 YES YES  127 a 1377.58 6.33160 YES YES  128 a 1377.95 0.29258 YES YES  129 a 1378.72 3.55675 YES YES  130 a 1448.35 5.71496 YES YES  131 a 1448.40 5.65426 YES YES  132 a 1448.94 3.73895 YES YES  133 a 1449.17 1.40145 YES YES  134 a 1459.99 33.92319 YES YES  135 a 1460.61 93.97417 YES YES  136 a 1484.25 5.36341 YES YES  137 a 1490.38 1.66670 YES YES  138 a 1490.56 2.29639 YES YES  139 a 1529.93 80.23781 YES YES  140 a 1538.08 5.07767 YES YES  141 a 1538.22 16.04139 YES YES  142 a 1570.69 78.36168 YES YES  143 a 1570.85 65.00372 YES YES  144 a 1628.04 5.98996 YES YES  145 a 1629.62 35.94803 YES YES  146 a 1631.19 6.34890 YES YES  147 a 1641.31 6.23182 YES YES  148 a 1641.37 13.53066 YES YES  149 a 1653.31 119.29707 YES YES  150 a 1653.60 5.18807 YES YES  151 a 1654.14 4.58018 YES YES  152 a 1674.55 33.65295 YES YES  153 a 1674.63 19.57367 YES YES  154 a 3166.67 1.21979 YES YES  155 a 3171.29 0.94637 YES YES  156 a 3171.54 0.67418 YES YES  157 a 3192.06 0.58564 YES YES  158 a 3192.43 0.58907 YES YES  159 a 3193.20 0.19642 YES YES  160 a 3194.89 0.01403 YES YES  161 a 3194.90 0.29229 YES YES  162 a 3195.44 0.22591 YES YES  163 a 3195.64 0.06798 YES YES  164 a 3196.35 0.07634 YES YES  165 a 3206.06 0.05064 YES YES  166 a 3206.43 0.05027 YES YES  167 a 3210.91 3.43594 YES YES  168 a 3211.88 3.37983 YES YES  169 a 3212.40 1.59586 YES YES  170 a 3213.27 0.69616 YES YES  171 a 3215.79 1.74536 YES YES  172 a 3216.05 1.77813 YES YES  173 a 3221.49 0.39641 YES YES  174 a 3222.40 0.42610 YES YES |

### [(bipy)In−In(bipy)]^2+^ (triplet)

Optimized atomic coordinates from the DFT calculations (BHLYP/SV(P) level) (Bohr Units):

| -1.15383037268822 2.46600715075111 2.23706300306729 In  1.15211168340997 -2.45934090639303 2.28828755580981 In  -2.31115241749139 5.11045413282405 -0.84919668175841 N  1.69049098784303 5.59478868957462 2.24888296798414 N  -1.70514167485518 -5.57750406208505 2.28797792342118 N  2.35463437556767 -5.14862053559130 -0.74190161041528 N  -4.33139382785568 4.74363669163579 -2.31488534452238 C  -0.92716991294653 7.22780854803645 -1.09133210207500 C  1.26629159700116 7.50326829249958 0.62680621546428 C  3.61959824037265 5.71600592482074 3.87005879826851 C  -3.66359645980185 -5.67056285661795 3.87538344651827 C  -1.26316062664358 -7.50522298610344 0.69375740726616 C  0.96367256513175 -7.26121679598661 -0.98584911274641 C  4.40481211806607 -4.81014214683278 -2.17240624022944 C  -5.08543314678694 6.46793784460731 -4.12115657928101 C  -1.58110923808146 9.04422149147265 -2.87412015942776 C  2.83067722245068 9.61429604431218 0.61978418255046 C  5.25189734091092 7.74986118066497 3.95718473958605 C  -5.30969568127342 -7.69364701653661 3.95377246570574 C  -2.84048732758866 -9.60660072509009 0.67956564638372 C  1.64168378797548 -9.10212565472732 -2.73415476720042 C  5.18394215706623 -6.55989588433845 -3.94325176401697 C  -3.67457667546388 8.66244104486522 -4.40119335728784 C  4.83955304443205 9.73898148631167 2.29670616490446 C  -4.88010061347597 -9.70190470435158 2.32094631103938 C  3.76640259949444 -8.74985948068305 -4.22476419079992 C  -5.38360364189560 3.00625062097926 -2.02367312133564 H  3.85550353336929 4.12850229502927 5.14926602025424 H  -3.91264531370470 -4.06855471069464 5.13386148984987 H  5.46117659570451 -3.07525719628094 -1.88125152040789 H  -6.73921617015976 6.09847769044827 -5.27116792788654 H  -0.48016280407368 10.75285423411126 -3.08299476153555 H  2.49853201873085 11.16361356310984 -0.67039083840821 H  6.79607795935729 7.77403531181034 5.30231633159197 H  -6.87780711231117 -7.69488525999928 5.27115161518620 H  -2.49487659382041 -11.17096342402426 -0.58872955516163 H  0.53571676448812 -10.80741296211205 -2.94384697093221 H  6.86174613338930 -6.21309293903849 -5.06518079701231 H  -4.19957552738753 10.07238612186994 -5.79371785181072 H  6.06601366158743 11.38164673511100 2.30798508479671 H  -6.11693461446751 -11.33680721307284 2.32643811319468 H  4.31033385946743 -10.17916342844279 -5.59002305636119 H |
| --- |

List of calculated frequencies (BHLYP/SV(P) level):

| mode symmetry wave number IR intensity selection rules  cm**(-1) km/mol IR RAMAN |
| --- |
| 1 -0.00 0.00000 - -  2 -0.00 0.00000 - -  3 0.00 0.00000 - -  4 0.00 0.00000 - -  5 0.00 0.00000 - -  6 0.00 0.00000 - -  7 a 12.92 0.77054 YES YES  8 a 16.21 0.58194 YES YES  9 a 16.66 0.98825 YES YES  10 a 38.52 0.11311 YES YES  11 a 39.30 0.33346 YES YES  12 a 76.87 0.00023 YES YES  13 a 79.86 0.57245 YES YES  14 a 84.37 0.09809 YES YES  15 a 87.77 0.72353 YES YES  16 a 104.37 0.77659 YES YES  17 a 144.70 10.42839 YES YES  18 a 146.85 5.09995 YES YES  19 a 164.05 2.94152 YES YES  20 a 176.68 0.23301 YES YES  21 a 177.09 1.16090 YES YES  22 a 192.81 0.01720 YES YES  23 a 242.42 12.26308 YES YES  24 a 251.59 0.33934 YES YES  25 a 253.80 0.29279 YES YES  26 a 255.93 0.62004 YES YES  27 a 374.56 2.03124 YES YES  28 a 375.17 0.01974 YES YES  29 a 442.79 1.47343 YES YES  30 a 443.21 0.64393 YES YES  31 a 448.60 8.27501 YES YES  32 a 448.61 9.43177 YES YES  33 a 472.01 0.63577 YES YES  34 a 472.26 0.44212 YES YES  35 a 475.47 2.32903 YES YES  36 a 476.93 0.42383 YES YES  37 a 585.90 0.48190 YES YES  38 a 586.04 0.00170 YES YES  39 a 669.53 13.28131 YES YES  40 a 669.75 7.92331 YES YES  41 a 684.93 1.46382 YES YES  42 a 686.59 0.62712 YES YES  43 a 686.65 1.08336 YES YES  44 a 686.95 0.84052 YES YES  45 a 786.19 24.43953 YES YES  46 a 786.36 17.61636 YES YES  47 a 798.68 0.68251 YES YES  48 a 798.81 0.00252 YES YES  49 a 805.85 2.20472 YES YES  50 a 806.27 0.75843 YES YES  51 a 820.56 78.24666 YES YES  52 a 820.74 72.52968 YES YES  53 a 878.91 0.61068 YES YES  54 a 879.09 0.00954 YES YES  55 a 960.90 0.34047 YES YES  56 a 960.93 0.17618 YES YES  57 a 962.61 0.77815 YES YES  58 a 962.65 1.27068 YES YES  59 a 1047.10 0.05094 YES YES  60 a 1047.15 0.01688 YES YES  61 a 1048.60 0.17554 YES YES  62 a 1048.63 0.03306 YES YES  63 a 1071.56 3.37410 YES YES  64 a 1073.06 27.88704 YES YES  65 a 1073.35 16.21695 YES YES  66 a 1074.08 0.63098 YES YES  67 a 1091.84 0.25737 YES YES  68 a 1091.86 0.01376 YES YES  69 a 1094.98 0.44385 YES YES  70 a 1095.00 0.56319 YES YES  71 a 1095.40 1.38084 YES YES  72 a 1095.48 0.48546 YES YES  73 a 1127.12 2.38625 YES YES  74 a 1127.25 0.12317 YES YES  75 a 1133.91 10.03342 YES YES  76 a 1134.22 5.61381 YES YES  77 a 1172.44 1.07100 YES YES  78 a 1172.47 4.17111 YES YES  79 a 1185.48 0.90780 YES YES  80 a 1185.58 0.42500 YES YES  81 a 1214.54 21.71028 YES YES  82 a 1214.64 12.86566 YES YES  83 a 1229.80 7.98012 YES YES  84 a 1230.33 0.11973 YES YES  85 a 1340.62 19.07376 YES YES  86 a 1340.64 30.89816 YES YES  87 a 1347.64 7.02969 YES YES  88 a 1348.76 2.39379 YES YES  89 a 1369.32 67.77639 YES YES  90 a 1370.40 3.72323 YES YES  91 a 1380.74 33.67152 YES YES  92 a 1381.46 3.33023 YES YES  93 a 1392.63 26.44203 YES YES  94 a 1392.71 44.52064 YES YES  95 a 1522.31 34.05498 YES YES  96 a 1522.36 4.82988 YES YES  97 a 1539.84 65.30500 YES YES  98 a 1539.97 108.88826 YES YES  99 a 1571.70 63.44941 YES YES  100 a 1571.94 38.67096 YES YES  101 a 1586.71 87.08569 YES YES  102 a 1590.02 1.30423 YES YES  103 a 1690.03 3.45231 YES YES  104 a 1690.63 0.45533 YES YES  105 a 1707.55 16.00499 YES YES  106 a 1707.61 10.51485 YES YES  107 a 1727.40 121.60385 YES YES  108 a 1727.46 16.42855 YES YES  109 a 1737.83 26.73159 YES YES  110 a 1737.91 17.66059 YES YES  111 a 3293.74 2.07749 YES YES  112 a 3293.79 2.20648 YES YES  113 a 3295.69 4.55316 YES YES  114 a 3295.75 2.03943 YES YES  115 a 3309.80 0.08331 YES YES  116 a 3309.82 0.07628 YES YES  117 a 3310.53 7.64160 YES YES  118 a 3310.55 2.05034 YES YES  119 a 3325.30 8.61960 YES YES  120 a 3325.31 6.43408 YES YES  121 a 3325.85 2.99792 YES YES  122 a 3325.86 0.81043 YES YES  123 a 3331.80 2.87326 YES YES  124 a 3331.80 2.69578 YES YES  125 a 3348.08 5.53663 YES YES  126 a 3348.08 2.01031 YES YES |

Optimized atomic coordinates from the DFT calculations (B3LYP/SV(P) level) (Bohr Units):

| -1.14377339285991 2.50799341274619 2.10031190354299 In  1.13822699860953 -2.50293751504156 2.13672727574527 In  -2.39243039038898 5.21080058740288 -0.87479054438258 N  1.68869813720881 5.61479383051951 2.18551716165797 N  -1.69699897188987 -5.60703003228498 2.21916978701589 N  2.42148362247844 -5.23645056278865 -0.79513823949774 N  -4.46575868286728 4.88670027890604 -2.32454311620811 C  -1.00850985030313 7.37265914991528 -1.06880512577082 C  1.22099516771859 7.59814582237265 0.61280026756875 C  3.67543407393560 5.69557495870170 3.78388812181840 C  -3.70403496776696 -5.66989174234789 3.79282757227653 C  -1.21075633590535 -7.60702548160700 0.67340658468719 C  1.03989354350252 -7.40039304657272 -0.98214166667173 C  4.51281395911376 -4.92870394933258 -2.22246077850269 C  -5.27208680352454 6.68875140334892 -4.05429962025054 C  -1.73214896423422 9.26205797999212 -2.78371785527632 C  2.80272884540501 9.72651306378876 0.65048970291751 C  5.31392923115119 7.74358430775775 3.89532225271691 C  -5.34531012424047 -7.71564285476896 3.90474861156014 C  -2.79422874081164 -9.73404955740735 0.71335388075429 C  1.78472629758926 -9.30906139237185 -2.66634057270484 C  5.34048050520550 -6.75013087362797 -3.92158081007742 C  -3.87086394394346 8.92257589335947 -4.28461437875058 C  4.85993731919219 9.80276406323760 2.29527660489038 C  -4.87229299349092 -9.79186269229487 2.33244060205253 C  3.94210090109086 -8.98655422356261 -4.14407889291621 C  -5.50752478714618 3.11681907464730 -2.07247372907855 H  3.94217923539654 4.05514993268013 5.01697441601872 H  -3.98519056687584 -4.01653967165697 5.00526897115594 H  5.55154312676948 -3.15607016557015 -1.97735251978200 H  -6.96318580790617 6.34965544712261 -5.18974240275122 H  -0.64002126726704 11.00120614197267 -2.95365542535911 H  2.43902255986349 11.32928610368345 -0.59251397154319 H  6.90184666403278 7.72771730124168 5.21538959263801 H  -6.95001757449253 -7.68496567309659 5.20407835008940 H  -2.41587935189767 -11.34993934378692 -0.50810764600723 H  0.69464661955816 -11.05007957706708 -2.83017679911456 H  7.04565437398586 -6.42378940670230 -5.03956712960228 H  -4.44292460168427 10.39274716896470 -5.61979530130814 H  6.09479491961311 11.45958080377913 2.33316833330340 H  -6.10869814434487 -11.44748266034002 2.37192772767150 H  4.53069865546392 -10.47178209806898 -5.45518402229545 H |
| --- |

List of calculated frequencies (B3LYP/SV(P) level):

| mode symmetry wave number IR intensity selection rules  cm**(-1) km/mol IR RAMAN |
| --- |
| 1 -0.00 0.00000 - -  2 -0.00 0.00000 - -  3 -0.00 0.00000 - -  4 0.00 0.00000 - -  5 0.00 0.00000 - -  6 0.00 0.00000 - -  7 a 11.76 0.68305 YES YES  8 a 15.17 0.60959 YES YES  9 a 15.86 0.79849 YES YES  10 a 36.13 1.07932 YES YES  11 a 37.56 0.17713 YES YES  12 a 74.18 0.00154 YES YES  13 a 80.79 0.07015 YES YES  14 a 84.93 0.10788 YES YES  15 a 86.57 2.68854 YES YES  16 a 97.29 0.96369 YES YES  17 a 141.16 18.83942 YES YES  18 a 144.26 4.44817 YES YES  19 a 156.46 3.74051 YES YES  20 a 175.37 0.36593 YES YES  21 a 176.74 0.61955 YES YES  22 a 184.95 0.11580 YES YES  23 a 234.29 0.03743 YES YES  24 a 241.11 0.02034 YES YES  25 a 241.73 0.39478 YES YES  26 a 246.45 0.09297 YES YES  27 a 363.20 14.08110 YES YES  28 a 364.46 0.11683 YES YES  29 a 423.99 1.59338 YES YES  30 a 424.26 0.62199 YES YES  31 a 430.05 7.45220 YES YES  32 a 430.20 15.13769 YES YES  33 a 452.60 2.23886 YES YES  34 a 452.70 0.36798 YES YES  35 a 459.81 1.00474 YES YES  36 a 461.19 0.15433 YES YES  37 a 564.31 0.46216 YES YES  38 a 564.41 0.00404 YES YES  39 a 646.39 7.47438 YES YES  40 a 646.61 4.36307 YES YES  41 a 660.80 15.59929 YES YES  42 a 661.69 0.19672 YES YES  43 a 661.73 0.40112 YES YES  44 a 664.22 0.07459 YES YES  45 a 750.56 17.78801 YES YES  46 a 750.82 9.73587 YES YES  47 a 763.45 1.13312 YES YES  48 a 763.57 0.00275 YES YES  49 a 775.19 2.28265 YES YES  50 a 776.08 0.37246 YES YES  51 a 784.79 64.54534 YES YES  52 a 784.95 78.59733 YES YES  53 a 841.18 0.91697 YES YES  54 a 841.35 0.00825 YES YES  55 a 917.04 0.26135 YES YES  56 a 917.07 1.08438 YES YES  57 a 917.86 1.03640 YES YES  58 a 917.92 0.41419 YES YES  59 a 995.93 0.04636 YES YES  60 a 995.95 0.05219 YES YES  61 a 996.71 0.97561 YES YES  62 a 996.77 0.05440 YES YES  63 a 1018.51 152.43681 YES YES  64 a 1025.15 0.07930 YES YES  65 a 1026.22 16.03258 YES YES  66 a 1026.98 8.76450 YES YES  67 a 1040.94 0.21176 YES YES  68 a 1040.95 0.04012 YES YES  69 a 1044.22 0.18585 YES YES  70 a 1044.23 0.18433 YES YES  71 a 1054.16 1.38202 YES YES  72 a 1054.23 0.80963 YES YES  73 a 1084.43 0.88852 YES YES  74 a 1084.50 0.04821 YES YES  75 a 1088.57 6.55031 YES YES  76 a 1088.87 3.42422 YES YES  77 a 1129.96 2.16257 YES YES  78 a 1130.18 0.17426 YES YES  79 a 1146.17 0.25347 YES YES  80 a 1146.29 0.09708 YES YES  81 a 1179.05 13.21426 YES YES  82 a 1179.10 7.36235 YES YES  83 a 1190.17 81.56738 YES YES  84 a 1191.50 1.79903 YES YES  85 a 1292.09 102.37029 YES YES  86 a 1292.81 11.21837 YES YES  87 a 1313.04 1.76049 YES YES  88 a 1313.11 1.03056 YES YES  89 a 1326.04 225.07660 YES YES  90 a 1328.43 5.03270 YES YES  91 a 1341.87 148.91147 YES YES  92 a 1344.15 4.61517 YES YES  93 a 1348.14 17.01351 YES YES  94 a 1348.23 29.38929 YES YES  95 a 1459.65 137.68829 YES YES  96 a 1460.51 4.97958 YES YES  97 a 1479.34 43.77616 YES YES  98 a 1479.48 78.01459 YES YES  99 a 1499.68 726.97568 YES YES  100 a 1504.94 87.05635 YES YES  101 a 1505.16 49.83296 YES YES  102 a 1510.92 11.19347 YES YES  103 a 1603.43 102.48090 YES YES  104 a 1604.98 1.77139 YES YES  105 a 1620.09 17.55995 YES YES  106 a 1620.12 10.86408 YES YES  107 a 1643.73 58.18114 YES YES  108 a 1643.75 11.09726 YES YES  109 a 1653.06 4.90301 YES YES  110 a 1653.12 2.72550 YES YES  111 a 3186.44 2.29928 YES YES  112 a 3186.45 2.36017 YES YES  113 a 3188.22 5.44869 YES YES  114 a 3188.30 2.26563 YES YES  115 a 3205.42 0.22684 YES YES  116 a 3205.44 0.22709 YES YES  117 a 3206.22 12.41972 YES YES  118 a 3206.26 1.61847 YES YES  119 a 3219.61 5.32479 YES YES  120 a 3219.63 4.87095 YES YES  121 a 3220.31 7.04160 YES YES  122 a 3220.35 1.92331 YES YES  123 a 3224.83 3.42224 YES YES  124 a 3224.83 3.07055 YES YES  125 a 3237.79 3.83649 YES YES  126 a 3237.79 1.25235 YES YES |

### [(bipy)_2_In−In(bipy)_2_]^2+^ (triplet)

Optimized atomic coordinates from the DFT calculations (BHLYP/SV(P) level) (Bohr Units):

| 1.65178018777448 2.43096810284637 0.67093060423072 In  -0.97270696135655 -2.44698409115227 0.00766517997034 In  0.19581437431439 4.40566666263241 4.42362281606470 N  3.70242497407408 6.35023496840346 1.31175305181468 N  -1.24331446455201 5.02657525406295 -1.63548641037788 N  2.68921763887568 2.60750329523533 -3.76381060114634 N  0.16713142445008 -4.64839390737228 3.78420878587616 N  -3.88004803507680 -5.66109092634199 0.88542036524323 N  1.52058444327182 -4.88678077550528 -2.23086958315497 N  -2.46690619922797 -2.22294882516506 -3.91118836461461 N  -1.52229706248176 3.30446649704987 5.89786549936103 C  1.50678457364084 6.39244849703234 5.28634715322271 C  3.41402389097176 7.48764329193410 3.55560265348046 C  5.39207173414408 7.24345744060126 -0.32382850838682 C  -3.07353313282912 6.30146524455341 -0.47918161367607 C  -0.97571497015904 5.23932445924617 -4.14238966083128 C  1.08564614579799 3.75685104292715 -5.33562119325703 C  4.56987522924773 1.22077559796011 -4.67473136714641 C  2.14562511431355 -3.96849139024717 5.17375631634309 C  -1.38784143604885 -6.45888050558532 4.60642766300630 C  -3.56174723841218 -7.09061742890090 2.95076626373818 C  -5.78570459326480 -6.14578742623130 -0.68004847674439 C  3.54951598592308 -6.13636700281448 -1.33621390117551 C  0.86324644820326 -5.15497054507949 -4.73299392212314 C  -1.24920332986686 -3.75045364885282 -5.62582672922536 C  -4.39540948868046 -0.75504428462013 -4.67731419112148 C  -2.05383639385759 4.13047318337394 8.31662456168763 C  1.08807435576972 7.32295204171894 7.71543023989990 C  4.87577628875822 9.57969413519376 4.20440376136594 C  6.89153859206802 9.32286516884611 0.17339594167269 C  -4.73886621471057 7.87366843628880 -1.74168146655007 C  -2.56989545976508 6.79173618281552 -5.54554683694925 C  1.35977968018198 3.53154237768194 -7.94007422233283 C  4.98370664042232 0.91784983956860 -7.24101667443392 C  2.70948537533458 -5.07934189900374 7.47295009788296 C  -0.96171338312158 -7.65429383977478 6.91224776002365 C  -5.20579293843662 -9.06899749144842 3.48664728468770 C  -7.49958223038886 -8.07984381029580 -0.27172055377853 C  5.02669479722100 -7.69846059379965 -2.76422503998920 C  2.32357907935126 -6.78351988349291 -6.28808811220005 C  -2.14276895369968 -3.81284463085853 -8.15910328543639 C  -5.29654572055732 -0.71579088352199 -7.09603774461165 C  -0.71269308058978 6.19097166331171 9.23796635559818 C  6.62730168450440 10.50921466009618 2.49813813299866 C  -4.46891393799420 8.12556748324794 -4.33478989160922 C  3.33586528609213 2.10054681179184 -8.89841752055109 C  1.11363386206197 -6.96429056305577 8.35215881159770 C  -7.19693763012842 -9.56930816000548 1.85887704610532 C  4.36511658135714 -8.03399065396911 -5.33320215920093 C  -4.12548003463684 -2.33558115543082 -8.88644779478646 C  -2.50706901843589 1.69366674717492 5.08918504883003 H  5.54927142542129 6.24289936346001 -2.10622212426443 H  -3.21106513783789 6.06067853501347 1.55028670098841 H  5.78715676207841 0.30922141348728 -3.29386080291122 H  3.32501318725499 -2.47146052857591 4.40712679007421 H  -5.93411010780518 -4.94586042590929 -2.33287829633508 H  3.97913975727264 -5.84899121427231 0.64827100963031 H  -5.25003012710104 0.42481805810288 -3.22501506668468 H  -3.48118869647870 3.18865408748808 9.44331884093616 H  2.14971395894082 8.91635273606233 8.42839892946309 H  4.65567532680295 10.49510815012143 6.01780197340887 H  8.23081713585920 9.98830311227591 -1.22562323793408 H  -6.19590233034788 8.87606479819431 -0.70905999123687 H  -2.32915523487297 6.98488148468237 -7.56529465207459 H  0.04580412792135 4.42602162186202 -9.22391038152867 H  6.54526014674229 -0.22634752709426 -7.90852454833476 H  4.35526988069518 -4.48293304675034 8.53586113758945 H  -2.22457842521073 -9.10723451710966 7.59695051364536 H  -4.94068217244734 -10.23706201719497 5.14178333662305 H  -9.01857914547551 -8.40439832852263 -1.60591881462315 H  6.63305495254473 -8.65606203614549 -1.93303600004499 H  1.81006464724904 -7.03856361174252 -8.25039527301665 H  -1.25131769549698 -5.04770375763767 -9.52357124535194 H  -6.87282469774503 0.48680209083038 -7.60504836927305 H  -1.06197919836738 6.91040135117603 11.12554938496923 H  7.77038694645813 12.14088095987397 2.98061053028466 H  -5.71588285828164 9.34887643558824 -5.40796765546479 H  3.57173404360178 1.90638315876265 -10.92554863336562 H  1.47379538062559 -7.89142730178508 10.14486726056516 H  -8.48562762563661 -11.11391711925042 2.25392994216362 H  5.46630260888178 -9.27805685128824 -6.53392090295506 H  -4.80621725129472 -2.40645529266919 -10.81885879271093 H |
| --- |

List of calculated frequencies (BHLYP/SV(P) level):

| mode symmetry wave number IR intensity selection rules  cm**(-1) km/mol IR RAMAN |
| --- |
| 1 -0.00 0.00000 - -  2 -0.00 0.00000 - -  3 -0.00 0.00000 - -  4 -0.00 0.00000 - -  5 0.00 0.00000 - -  6 0.00 0.00000 - -  7 a 11.37 0.17260 YES YES  8 a 15.09 0.51403 YES YES  9 a 18.42 0.16364 YES YES  10 a 25.05 0.34117 YES YES  11 a 25.57 0.04776 YES YES  12 a 26.97 0.44616 YES YES  13 a 29.63 0.79928 YES YES  14 a 32.48 1.46283 YES YES  15 a 35.68 2.01786 YES YES  16 a 41.92 0.22991 YES YES  17 a 50.29 2.22389 YES YES  18 a 60.46 2.74780 YES YES  19 a 76.91 1.66868 YES YES  20 a 79.77 0.16269 YES YES  21 a 82.66 0.27660 YES YES  22 a 86.95 0.51792 YES YES  23 a 87.84 0.16641 YES YES  24 a 92.73 0.07195 YES YES  25 a 95.40 0.39995 YES YES  26 a 104.37 0.88781 YES YES  27 a 112.89 0.57588 YES YES  28 a 114.44 1.74539 YES YES  29 a 133.03 4.38774 YES YES  30 a 136.17 2.28491 YES YES  31 a 143.15 2.62146 YES YES  32 a 143.97 3.92415 YES YES  33 a 159.15 1.89224 YES YES  34 a 159.47 5.01107 YES YES  35 a 167.59 65.55150 YES YES  36 a 177.78 9.19117 YES YES  37 a 186.64 3.37530 YES YES  38 a 198.87 10.21332 YES YES  39 a 227.61 8.62994 YES YES  40 a 230.06 0.82734 YES YES  41 a 234.34 6.86956 YES YES  42 a 238.16 10.14165 YES YES  43 a 252.30 39.29341 YES YES  44 a 255.16 0.61163 YES YES  45 a 256.25 6.41832 YES YES  46 a 261.06 4.11779 YES YES  47 a 365.47 2.43740 YES YES  48 a 365.90 0.28245 YES YES  49 a 367.97 0.30392 YES YES  50 a 380.92 2.73568 YES YES  51 a 425.19 0.47974 YES YES  52 a 440.41 0.46231 YES YES  53 a 443.28 1.89688 YES YES  54 a 444.43 0.49609 YES YES  55 a 445.90 0.33056 YES YES  56 a 447.62 14.11701 YES YES  57 a 448.52 36.95074 YES YES  58 a 449.32 5.98452 YES YES  59 a 451.38 3.89172 YES YES  60 a 463.14 5.78430 YES YES  61 a 468.60 3.24896 YES YES  62 a 472.43 4.29684 YES YES  63 a 473.77 31.57571 YES YES  64 a 475.80 1.65900 YES YES  65 a 482.70 5.70957 YES YES  66 a 487.56 1.54875 YES YES  67 a 563.13 0.37114 YES YES  68 a 589.76 0.12458 YES YES  69 a 591.83 0.21690 YES YES  70 a 592.44 0.13525 YES YES  71 a 641.57 15.65113 YES YES  72 a 658.51 14.37528 YES YES  73 a 661.29 8.02141 YES YES  74 a 662.19 11.09001 YES YES  75 a 675.94 0.23869 YES YES  76 a 679.19 2.29943 YES YES  77 a 680.49 19.29673 YES YES  78 a 681.17 11.55932 YES YES  79 a 684.78 25.17350 YES YES  80 a 688.87 0.65936 YES YES  81 a 689.98 3.11932 YES YES  82 a 690.68 4.80632 YES YES  83 a 724.91 15.98005 YES YES  84 a 771.64 0.61182 YES YES  85 a 789.58 59.05052 YES YES  86 a 790.20 17.94933 YES YES  87 a 793.40 45.54167 YES YES  88 a 793.76 15.17466 YES YES  89 a 794.53 9.24711 YES YES  90 a 796.77 0.64641 YES YES  91 a 798.52 8.02097 YES YES  92 a 798.68 1.41820 YES YES  93 a 808.73 0.34202 YES YES  94 a 810.28 4.16458 YES YES  95 a 810.65 4.11114 YES YES  96 a 818.63 59.42334 YES YES  97 a 818.79 58.36952 YES YES  98 a 819.80 9.70114 YES YES  99 a 820.73 43.72810 YES YES  100 a 882.60 1.12467 YES YES  101 a 883.25 0.52282 YES YES  102 a 884.61 0.28359 YES YES  103 a 894.68 1.43909 YES YES  104 a 899.11 0.53576 YES YES  105 a 959.99 2.96304 YES YES  106 a 961.82 1.86313 YES YES  107 a 962.25 8.68749 YES YES  108 a 963.77 0.31125 YES YES  109 a 963.86 0.75662 YES YES  110 a 965.88 3.00347 YES YES  111 a 1001.65 171.39222 YES YES  112 a 1021.90 0.26957 YES YES  113 a 1025.85 0.05164 YES YES  114 a 1047.44 0.14209 YES YES  115 a 1049.24 1.49623 YES YES  116 a 1049.93 0.47499 YES YES  117 a 1051.31 0.32317 YES YES  118 a 1051.90 0.03006 YES YES  119 a 1053.67 1.90515 YES YES  120 a 1055.86 1.48574 YES YES  121 a 1057.89 16.10180 YES YES  122 a 1058.27 9.72546 YES YES  123 a 1061.23 35.00131 YES YES  124 a 1065.45 17.59956 YES YES  125 a 1069.59 23.20150 YES YES  126 a 1069.91 21.63230 YES YES  127 a 1073.27 42.20881 YES YES  128 a 1073.54 21.40103 YES YES  129 a 1074.24 33.82704 YES YES  130 a 1086.41 0.35104 YES YES  131 a 1086.99 1.70559 YES YES  132 a 1089.11 0.54940 YES YES  133 a 1089.63 0.30101 YES YES  134 a 1090.11 0.25320 YES YES  135 a 1092.31 0.24255 YES YES  136 a 1093.30 0.25066 YES YES  137 a 1094.78 2.18918 YES YES  138 a 1095.74 2.38489 YES YES  139 a 1097.85 1.59584 YES YES  140 a 1098.24 0.58004 YES YES  141 a 1125.93 4.29639 YES YES  142 a 1127.87 4.03921 YES YES  143 a 1128.98 5.65493 YES YES  144 a 1134.43 4.45569 YES YES  145 a 1137.92 5.26592 YES YES  146 a 1137.97 5.88842 YES YES  147 a 1165.87 37.04817 YES YES  148 a 1168.84 6.39884 YES YES  149 a 1169.09 5.05360 YES YES  150 a 1169.27 6.04607 YES YES  151 a 1182.11 0.75161 YES YES  152 a 1183.36 2.49311 YES YES  153 a 1183.99 0.01482 YES YES  154 a 1193.54 27.54264 YES YES  155 a 1208.68 32.66005 YES YES  156 a 1208.94 1.12682 YES YES  157 a 1209.61 31.78669 YES YES  158 a 1209.83 17.64884 YES YES  159 a 1225.88 22.19692 YES YES  160 a 1227.50 5.46170 YES YES  161 a 1228.33 7.97696 YES YES  162 a 1229.84 13.84470 YES YES  163 a 1288.35 54.06290 YES YES  164 a 1338.63 9.31346 YES YES  165 a 1340.23 21.91694 YES YES  166 a 1342.58 15.46588 YES YES  167 a 1344.56 4.09450 YES YES  168 a 1348.49 11.06384 YES YES  169 a 1349.20 16.53769 YES YES  170 a 1350.47 5.60398 YES YES  171 a 1366.85 100.30168 YES YES  172 a 1367.36 5.27976 YES YES  173 a 1368.63 0.15122 YES YES  174 a 1377.86 39.57704 YES YES  175 a 1379.15 29.89054 YES YES  176 a 1387.99 9.70605 YES YES  177 a 1388.22 5.31779 YES YES  178 a 1389.42 33.61321 YES YES  179 a 1390.49 16.07436 YES YES  180 a 1391.86 36.64609 YES YES  181 a 1393.17 12.25093 YES YES  182 a 1445.81 0.12512 YES YES  183 a 1511.11 19.31682 YES YES  184 a 1517.95 26.65718 YES YES  185 a 1519.52 28.64782 YES YES  186 a 1522.65 6.33893 YES YES  187 a 1523.50 4.34429 YES YES  188 a 1536.97 47.31640 YES YES  189 a 1537.98 55.68348 YES YES  190 a 1538.37 68.16169 YES YES  191 a 1553.99 43.59052 YES YES  192 a 1571.00 60.40985 YES YES  193 a 1574.97 23.68838 YES YES  194 a 1575.69 24.55765 YES YES  195 a 1578.50 185.83978 YES YES  196 a 1598.80 80.05265 YES YES  197 a 1599.95 109.47547 YES YES  198 a 1603.42 45.58762 YES YES  199 a 1614.04 270.52179 YES YES  200 a 1646.52 94.06105 YES YES  201 a 1682.37 88.75856 YES YES  202 a 1687.15 23.00724 YES YES  203 a 1700.18 17.28754 YES YES  204 a 1702.47 39.72529 YES YES  205 a 1710.79 14.97575 YES YES  206 a 1711.17 39.52271 YES YES  207 a 1717.18 13.66711 YES YES  208 a 1718.76 11.09880 YES YES  209 a 1726.52 55.60889 YES YES  210 a 1730.27 56.20340 YES YES  211 a 1731.02 45.35847 YES YES  212 a 1735.23 20.89649 YES YES  213 a 1741.32 38.70006 YES YES  214 a 1742.29 37.81919 YES YES  215 a 3251.76 13.14978 YES YES  216 a 3276.27 2.67376 YES YES  217 a 3278.81 3.63000 YES YES  218 a 3282.36 2.75166 YES YES  219 a 3293.70 1.85670 YES YES  220 a 3294.51 7.53323 YES YES  221 a 3299.35 10.22722 YES YES  222 a 3303.80 1.47648 YES YES  223 a 3303.85 0.85782 YES YES  224 a 3304.47 0.56424 YES YES  225 a 3305.26 0.75709 YES YES  226 a 3305.32 0.19393 YES YES  227 a 3305.64 0.34794 YES YES  228 a 3309.93 4.20609 YES YES  229 a 3313.19 0.22007 YES YES  230 a 3315.65 2.26958 YES YES  231 a 3317.10 0.56927 YES YES  232 a 3319.69 4.20625 YES YES  233 a 3320.80 0.36914 YES YES  234 a 3320.88 0.77101 YES YES  235 a 3321.73 1.77270 YES YES  236 a 3322.79 0.31498 YES YES  237 a 3323.27 0.45185 YES YES  238 a 3326.84 0.41350 YES YES  239 a 3328.97 1.74862 YES YES  240 a 3328.99 0.87427 YES YES  241 a 3329.73 0.84911 YES YES  242 a 3332.50 4.01952 YES YES  243 a 3332.62 1.34389 YES YES  244 a 3345.27 1.15781 YES YES  245 a 3346.08 0.32167 YES YES  246 a 3346.09 0.35864 YES YES |

Optimized atomic coordinates from the DFT calculations (B3LYP/SV(P) level) (Bohr Units):

| 0.66334995370145 2.67128413672729 -0.37027609425608 In  -0.68206278138458 -2.57697085208824 -0.45174132871203 In  -0.29315561484153 3.70823463833563 3.71029849960071 N  4.10664213700501 4.51525127078675 1.24932912840583 N  -1.54277448545528 5.90384678826249 -1.86575835695578 N  2.05632152144202 3.28024054727456 -4.36311782543705 N  0.47798378775903 -3.89662137518996 3.44079249421187 N  -4.06615466566932 -4.42986862874212 1.17621614113969 N  1.37807305506508 -5.77608697564748 -2.29743327776070 N  -2.24944787030126 -2.90780274168808 -4.46408650322217 N  -2.47439455514481 3.09738351481035 4.87268727340468 C  1.52237766510493 5.01596835589650 4.99542423372156 C  3.87668142004573 5.52606944914114 3.63100253273052 C  6.26245822017129 4.91478843664989 -0.06548997748671 C  -3.32438732141106 7.18261152020444 -0.54254516574450 C  -0.84924938614442 6.77713689256960 -4.22300161288388 C  1.04863637611793 5.32497578344870 -5.59403935279144 C  3.75415196113443 1.79588605751293 -5.55522432750985 C  2.73659053510904 -3.42129635258455 4.52713004233473 C  -1.30054619449978 -5.27610841845132 4.72356737488880 C  -3.73307032424276 -5.61551065174610 3.47483640101151 C  -6.31472977351352 -4.66574625874273 -0.02777393295153 C  3.15554823357185 -7.19123403628865 -1.12553106432847 C  0.56666609589170 -6.47581928822992 -4.66371194560532 C  -1.33755247646006 -4.86931482997428 -5.87068871497317 C  -3.94582837487431 -1.30401725726373 -5.47987406527495 C  -3.02250975102371 3.78353992179849 7.34219657327635 C  1.07653130993692 5.77701924599264 7.51412590245453 C  5.85594918239639 6.99001183333092 4.65191056900459 C  8.28212755559878 6.29550737109245 0.86511388784421 C  -4.50986330383252 9.33574286772261 -1.42861631923180 C  -1.97108698288775 8.99808989884186 -5.19378013597964 C  1.85913589598223 5.90048416522719 -8.07697042719638 C  4.60827837889561 2.25830268309548 -7.98456803176938 C  3.40748713324991 -4.31737860797691 6.89200078712198 C  -0.71686227522413 -6.25645802981283 7.14220088052220 C  -5.71225743692635 -7.08238296424265 4.50641114196575 C  -8.32350864213126 -6.03776547122949 0.92520521277679 C  4.22726221967569 -9.32866915714771 -2.19077134125058 C  1.55833354990643 -8.66457611283236 -5.81653523141597 C  -2.22497623740871 -5.24172447467783 -8.36002859505040 C  -4.88675550356442 -1.56495373596293 -7.91211945351143 C  -1.19107778670036 5.17245812659261 8.68591263799951 C  8.06074746989670 7.37684874624126 3.28644958491615 C  -3.79570041953989 10.27712948189716 -3.82086010433751 C  3.63328981469160 4.38704425278412 -9.26743528667685 C  1.62286131375672 -5.79149595868112 8.22203862136281 C  -8.00101292994618 -7.29727452736785 3.25508096221020 C  3.38870945723856 -10.09283980690718 -4.59796834531138 C  -4.00054475578754 -3.60232889850916 -9.37953090698244 C  -3.83520648007180 2.00333440767941 3.75537248275870 H  6.34709393637868 4.09088658524398 -1.95694631459608 H  -3.79609155539201 6.42709582738828 1.32090007096842 H  4.44946642044164 0.16091919363873 -4.48656619210977 H  4.05619287210925 -2.26280836315428 3.42481374221607 H  -6.48083970617975 -3.70617633025793 -1.84902440854787 H  3.72274605852629 -6.57063762463035 0.76057609377403 H  -4.56712871065134 0.26157406783773 -4.27161768874092 H  -4.82142636645181 3.24842871455868 8.20170533014946 H  2.51057090369399 6.82352518289949 8.56090521140282 H  5.65975630275200 7.83752041880476 6.52010990334500 H  9.98324561794871 6.53724575429937 -0.27858926347770 H  -5.94620945976630 10.27281590129436 -0.28041405343641 H  -1.39566895100287 9.72263796411727 -7.03548490275415 H  1.08172555667440 7.52741995781352 -9.07548372393810 H  5.98933103140670 1.00264586221278 -8.86562030629492 H  5.26170177140604 -3.88380532372295 7.68835385723611 H  -2.10878302989485 -7.36808689690318 8.17910595968154 H  -5.44170394456120 -8.06230814860744 6.29930278066632 H  -10.09675025963417 -6.14126204061171 -0.12571275625287 H  5.67085232020149 -10.38418572480380 -1.16008961134229 H  0.88200038150380 -9.25442313595490 -7.67170853304236 H  -1.51105407144325 -6.80059485748867 -9.50351698396268 H  -6.26603296046738 -0.21814438201594 -8.65024149234556 H  -1.53812174868088 5.75866287268089 10.63696942750859 H  9.59328190759827 8.51166407389807 4.08193444370298 H  -4.65918535982426 11.99343782611285 -4.58074559203216 H  4.25754911233747 4.83370651901349 -11.18603331413321 H  2.07108772045283 -6.54922803502735 10.09153817602036 H  -9.52723045311593 -8.43260801946676 4.06152290773857 H  4.15542013899566 -11.78856685262678 -5.49571916483513 H  -4.68818132592174 -3.88736742437029 -11.30741211210256 H |
| --- |

List of calculated frequencies (B3LYP/SV(P) level):

| mode symmetry wave number IR intensity selection rules  cm**(-1) km/mol IR RAMAN |
| --- |
| 1 -0.00 0.00000 - -  2 -0.00 0.00000 - -  3 0.00 0.00000 - -  4 0.00 0.00000 - -  5 0.00 0.00000 - -  6 0.00 0.00000 - -  7 a 7.02 0.00840 YES YES  8 a 13.18 0.81243 YES YES  9 a 14.03 0.42649 YES YES  10 a 23.10 0.01936 YES YES  11 a 24.90 0.02275 YES YES  12 a 25.85 0.21255 YES YES  13 a 27.70 0.26018 YES YES  14 a 29.52 0.45810 YES YES  15 a 33.72 7.76480 YES YES  16 a 50.23 0.00024 YES YES  17 a 52.30 5.95121 YES YES  18 a 73.58 0.01408 YES YES  19 a 76.82 0.11557 YES YES  20 a 77.21 0.06189 YES YES  21 a 87.07 0.09495 YES YES  22 a 97.11 0.57723 YES YES  23 a 98.84 1.52108 YES YES  24 a 99.35 1.34172 YES YES  25 a 102.49 0.55630 YES YES  26 a 104.79 5.92835 YES YES  27 a 109.87 76.69269 YES YES  28 a 119.78 11.85135 YES YES  29 a 124.88 1.25826 YES YES  30 a 138.12 77.15092 YES YES  31 a 140.46 5.61881 YES YES  32 a 142.82 281.26196 YES YES  33 a 159.72 26.29564 YES YES  34 a 160.81 0.07133 YES YES  35 a 172.47 44.94220 YES YES  36 a 187.69 0.10188 YES YES  37 a 191.79 1.10489 YES YES  38 a 211.91 0.00816 YES YES  39 a 223.98 21.19703 YES YES  40 a 226.07 121.96422 YES YES  41 a 234.26 9.05805 YES YES  42 a 234.96 0.22060 YES YES  43 a 235.95 4.71867 YES YES  44 a 237.76 0.04261 YES YES  45 a 242.46 20.55078 YES YES  46 a 259.09 0.07397 YES YES  47 a 364.66 2.38310 YES YES  48 a 364.77 8.05712 YES YES  49 a 366.94 3.15249 YES YES  50 a 367.48 0.15143 YES YES  51 a 418.82 0.87018 YES YES  52 a 419.15 4.28104 YES YES  53 a 424.80 34.74170 YES YES  54 a 425.81 0.57422 YES YES  55 a 425.87 0.18577 YES YES  56 a 426.70 0.19447 YES YES  57 a 428.40 21.78086 YES YES  58 a 429.73 0.94189 YES YES  59 a 435.72 1.04157 YES YES  60 a 435.93 0.73273 YES YES  61 a 439.27 3.13055 YES YES  62 a 439.74 9.19062 YES YES  63 a 461.10 19.78954 YES YES  64 a 462.40 0.55654 YES YES  65 a 465.78 12.28208 YES YES  66 a 467.38 0.22697 YES YES  67 a 554.06 1.67109 YES YES  68 a 554.10 3.15269 YES YES  69 a 558.09 1.58789 YES YES  70 a 558.31 0.07445 YES YES  71 a 634.72 1.23924 YES YES  72 a 634.99 0.81907 YES YES  73 a 636.98 0.92118 YES YES  74 a 637.12 1.45205 YES YES  75 a 647.04 133.53998 YES YES  76 a 653.94 331.96091 YES YES  77 a 659.80 2.16122 YES YES  78 a 659.85 0.88221 YES YES  79 a 661.12 7.66407 YES YES  80 a 661.27 0.29925 YES YES  81 a 662.22 25.41997 YES YES  82 a 664.12 0.01910 YES YES  83 a 722.34 53.07360 YES YES  84 a 722.52 5.89050 YES YES  85 a 731.28 57.85652 YES YES  86 a 731.71 3.24733 YES YES  87 a 749.59 5.18850 YES YES  88 a 749.76 10.08776 YES YES  89 a 753.62 8.29346 YES YES  90 a 753.98 0.80113 YES YES  91 a 756.67 221.80833 YES YES  92 a 764.66 321.34913 YES YES  93 a 769.61 42.66583 YES YES  94 a 769.64 121.49359 YES YES  95 a 773.48 22.48971 YES YES  96 a 773.56 49.49449 YES YES  97 a 775.66 67.96333 YES YES  98 a 776.39 3.15533 YES YES  99 a 813.74 8.80541 YES YES  100 a 814.07 1.00124 YES YES  101 a 821.49 6.90019 YES YES  102 a 821.79 1.25601 YES YES  103 a 884.17 786.70731 YES YES  104 a 891.14 4.84665 YES YES  105 a 892.09 88.92149 YES YES  106 a 893.37 13.83192 YES YES  107 a 893.51 14.59636 YES YES  108 a 899.59 27.19321 YES YES  109 a 900.66 34.98576 YES YES  110 a 901.57 37.67232 YES YES  111 a 902.00 13.39085 YES YES  112 a 947.15 4925.46050 YES YES  113 a 979.92 0.09443 YES YES  114 a 980.28 0.06902 YES YES  115 a 982.17 3.45004 YES YES  116 a 982.28 7.78909 YES YES  117 a 986.71 9.04221 YES YES  118 a 987.11 0.47436 YES YES  119 a 989.66 6.65973 YES YES  120 a 989.88 1.55307 YES YES  121 a 1003.04 21.17512 YES YES  122 a 1003.35 17.16234 YES YES  123 a 1010.81 9.72229 YES YES  124 a 1011.14 5.94626 YES YES  125 a 1014.32 0.24998 YES YES  126 a 1014.40 0.32725 YES YES  127 a 1019.77 0.05652 YES YES  128 a 1019.88 0.10409 YES YES  129 a 1020.02 0.35386 YES YES  130 a 1020.07 0.13649 YES YES  131 a 1024.99 0.02076 YES YES  132 a 1025.06 0.02291 YES YES  133 a 1029.78 79.95320 YES YES  134 a 1033.19 0.03583 YES YES  135 a 1050.19 12.61390 YES YES  136 a 1050.28 6.49763 YES YES  137 a 1052.57 5.67150 YES YES  138 a 1052.75 4.22672 YES YES  139 a 1069.57 0.89044 YES YES  140 a 1069.80 11.74692 YES YES  141 a 1071.72 1.68768 YES YES  142 a 1071.79 5.11273 YES YES  143 a 1075.24 5.44586 YES YES  144 a 1075.29 1.52897 YES YES  145 a 1077.91 5.68542 YES YES  146 a 1078.04 2.26469 YES YES  147 a 1124.23 26.21133 YES YES  148 a 1125.29 35.98798 YES YES  149 a 1128.62 20.89304 YES YES  150 a 1128.87 0.26361 YES YES  151 a 1147.84 13.98642 YES YES  152 a 1148.13 0.45227 YES YES  153 a 1150.63 11.38782 YES YES  154 a 1150.87 3.39026 YES YES  155 a 1160.48 255.64236 YES YES  156 a 1171.95 1434.37172 YES YES  157 a 1172.74 36.96654 YES YES  158 a 1172.78 22.38813 YES YES  159 a 1173.66 45.91351 YES YES  160 a 1173.80 54.45736 YES YES  161 a 1191.77 8.49144 YES YES  162 a 1192.06 0.27575 YES YES  163 a 1254.08 207.93890 YES YES  164 a 1259.59 759.90936 YES YES  165 a 1275.74 129.72342 YES YES  166 a 1277.65 45.95933 YES YES  167 a 1287.43 213.32045 YES YES  168 a 1306.85 2320.97487 YES YES  169 a 1310.00 140.02064 YES YES  170 a 1310.16 3.81957 YES YES  171 a 1314.83 30.59937 YES YES  172 a 1316.94 898.89820 YES YES  173 a 1343.05 17.03813 YES YES  174 a 1343.55 3.90003 YES YES  175 a 1345.82 3.15961 YES YES  176 a 1346.14 34.86605 YES YES  177 a 1351.72 15.75111 YES YES  178 a 1352.05 0.14504 YES YES  179 a 1354.80 9.30666 YES YES  180 a 1354.92 71.38219 YES YES  181 a 1362.98 85.87140 YES YES  182 a 1363.05 13.74488 YES YES  183 a 1413.29 314.11840 YES YES  184 a 1433.13 4201.07130 YES YES  185 a 1456.17 30.51915 YES YES  186 a 1456.21 18.27755 YES YES  187 a 1457.72 6.32516 YES YES  188 a 1458.00 171.56857 YES YES  189 a 1469.40 29.41388 YES YES  190 a 1469.45 26.94029 YES YES  191 a 1472.36 47.41264 YES YES  192 a 1472.44 73.34448 YES YES  193 a 1500.80 46.58702 YES YES  194 a 1500.91 105.74481 YES YES  195 a 1502.59 51.01274 YES YES  196 a 1502.75 59.56212 YES YES  197 a 1534.86 17.82560 YES YES  198 a 1536.19 0.06766 YES YES  199 a 1574.27 20.02504 YES YES  200 a 1575.53 233.06999 YES YES  201 a 1582.24 125.58744 YES YES  202 a 1582.45 121.69768 YES YES  203 a 1592.78 18.55459 YES YES  204 a 1593.64 116.01089 YES YES  205 a 1594.84 77.68221 YES YES  206 a 1594.91 70.38729 YES YES  207 a 1611.77 193.70314 YES YES  208 a 1612.21 22.13322 YES YES  209 a 1615.62 172.53941 YES YES  210 a 1616.22 1.68520 YES YES  211 a 1634.12 4.13534 YES YES  212 a 1634.14 0.22710 YES YES  213 a 1637.16 8.40652 YES YES  214 a 1637.21 2.85614 YES YES  215 a 3153.55 3.07156 YES YES  216 a 3153.80 13.91731 YES YES  217 a 3157.30 1.71208 YES YES  218 a 3157.51 12.55209 YES YES  219 a 3198.61 0.87169 YES YES  220 a 3198.82 0.87255 YES YES  221 a 3199.35 0.56208 YES YES  222 a 3199.55 0.54937 YES YES  223 a 3200.08 0.52240 YES YES  224 a 3200.26 0.44716 YES YES  225 a 3200.96 0.35453 YES YES  226 a 3201.10 0.35205 YES YES  227 a 3206.99 8.50572 YES YES  228 a 3207.41 9.71216 YES YES  229 a 3209.61 8.54353 YES YES  230 a 3210.19 9.00108 YES YES  231 a 3214.93 3.53460 YES YES  232 a 3215.00 2.68791 YES YES  233 a 3215.70 6.33496 YES YES  234 a 3215.75 3.65655 YES YES  235 a 3218.00 3.65516 YES YES  236 a 3218.13 2.92988 YES YES  237 a 3219.10 2.93537 YES YES  238 a 3219.29 2.42470 YES YES  239 a 3221.33 0.63215 YES YES  240 a 3221.49 0.49617 YES YES  241 a 3222.25 0.78550 YES YES  242 a 3222.58 0.65107 YES YES  243 a 3230.80 0.12563 YES YES  244 a 3230.89 0.12137 YES YES  245 a 3232.47 0.01189 YES YES  246 a 3232.61 0.01403 YES YES |

### [(bipy)_2_In−In(bipy)]^2+^ (triplet)

Optimized atomic coordinates from the DFT calculations (BHLYP/SV(P) level) (Bohr Units):

| -0.22487964988786 2.77844162037907 3.22595118382309 In  0.99956727686487 -1.58673091685673 -0.02504606256004 In  0.83272006378476 6.34490774532473 1.00442922375465 N  -3.82984706775996 5.10928434031552 2.58853791262317 N  2.62021536493882 0.27942077402269 -3.30187974225376 N  4.80492514755330 -2.96491032672311 -0.03767805841955 N  -0.50950020628574 -4.76105859509054 2.63183686567474 N  -1.10663480982850 -4.49040473557509 -2.40155736094311 N  3.18347826269440 6.80044217678113 0.23240312260201 C  -0.98674706012761 8.07043422542614 0.63077293114185 C  -3.55181161047682 7.40492041873240 1.54608503953459 C  -6.07700377612773 4.38878265131399 3.46542455214938 C  1.46570866980135 1.96936283851688 -4.83209325082362 C  5.10010953134508 -0.38144829328476 -3.74987184303836 C  6.25469724529334 -2.08387260343393 -2.01434881535291 C  5.83495654996905 -4.59024948812822 1.64518324727385 C  -0.20888645537503 -4.71372096256315 5.12706270123436 C  -1.85527617133234 -6.60965420223571 1.55448272672880 C  -2.10048584762556 -6.51270997667589 -1.23916632647060 C  -1.21974366800724 -4.30326424615635 -4.90843904052593 C  3.86497768003278 9.02161734317157 -0.96344782013990 C  -0.44012805949504 10.35027665639269 -0.55396710153571 C  -5.59655030436450 9.04653232538637 1.37079201206169 C  -8.19376390612101 5.91366125287361 3.34441578728192 C  2.60116727749579 3.04965054052750 -6.87314124315166 C  6.35121909417005 0.68169825191363 -5.87188566603490 C  8.79907674664149 -2.89938554814920 -2.21120409319289 C  8.26900935720413 -5.41982948074451 1.52274785029578 C  -1.22390163096963 -6.53397138402532 6.70568306811968 C  -2.95346470563590 -8.49762540465836 3.01718255738545 C  -3.27184767081576 -8.41124300353326 -2.62114873670533 C  -2.36032421132640 -6.11866565737211 -6.40668272881795 C  2.00920587046439 10.82966868835369 -1.35581738411738 C  -7.93707020026645 8.29439823665826 2.27662787974454 C  5.13902788487773 2.35555825447690 -7.41130145177149 C  9.79785369361551 -4.53045302263626 -0.48268676084125 C  -2.62719291564983 -8.46174541703035 5.61699348141477 C  -3.40670771624953 -8.21070114960401 -5.23243974433992 C  -6.18378350489028 2.51979165254236 4.30756994519816 H  4.57485948490788 5.33288023284415 0.58314600718997 H  -0.47977409076766 2.43301801594042 -4.35265397638864 H  4.59955317213831 -5.23703047721540 3.14820688222217 H  0.88829724255557 -3.15799219923870 5.89658703288545 H  -0.34854167543563 -2.64723348308247 -5.74273895847693 H  5.80064043758896 9.31805563065793 -1.56218145248231 H  -1.89367478410702 11.75336797347185 -0.86095402253781 H  -5.38436997368298 10.90511651735967 0.54852274146794 H  -9.98690542968930 5.25261353762506 4.08037333746670 H  1.58448732003053 4.38287693639262 -8.04733815563105 H  8.28518124137555 0.15789723243891 -6.27791252841122 H  9.96315173310807 -2.22073442436308 -3.74790072648558 H  8.99110994757701 -6.72279878794888 2.92586186792095 H  -0.91932391928126 -6.43501265781490 8.72850048639076 H  -4.06057823782332 -9.98618228093595 2.16059898144698 H  -4.05869814603926 -10.05359032794640 -1.69478966823548 H  -2.40621424846930 -5.89009403240274 -8.44106144009480 H  2.45650938798937 12.60478614749492 -2.27872619763783 H  -9.54146937542607 9.56471344533321 2.15417418689518 H  6.10673044603834 3.14846330779529 -9.03446542674725 H  11.74715359922570 -5.14078902913415 -0.64881001830427 H  -3.46838073203869 -9.92778366226919 6.77737666977682 H  -4.31073100324140 -9.68742828497206 -6.33024313472956 H |
| --- |

List of calculated frequencies (BHLYP/SV(P) level):

| mode symmetry wave number IR intensity selection rules  cm**(-1) km/mol IR RAMAN |
| --- |
| 1 -0.00 0.00000 - -  2 0.00 0.00000 - -  3 0.00 0.00000 - -  4 0.00 0.00000 - -  5 0.00 0.00000 - -  6 0.00 0.00000 - -  7 a 9.53 0.83742 YES YES  8 a 14.12 0.25884 YES YES  9 a 19.58 1.32152 YES YES  10 a 26.03 0.45598 YES YES  11 a 27.99 0.07836 YES YES  12 a 32.98 0.17521 YES YES  13 a 35.02 0.24994 YES YES  14 a 50.73 0.01973 YES YES  15 a 72.11 0.44077 YES YES  16 a 78.41 0.17824 YES YES  17 a 80.58 0.05695 YES YES  18 a 85.11 0.15115 YES YES  19 a 95.08 0.06301 YES YES  20 a 97.49 0.39464 YES YES  21 a 110.01 0.47981 YES YES  22 a 124.72 4.44486 YES YES  23 a 138.05 4.70356 YES YES  24 a 142.43 3.12014 YES YES  25 a 159.52 1.60416 YES YES  26 a 161.75 34.27783 YES YES  27 a 163.51 1.66536 YES YES  28 a 185.45 13.25856 YES YES  29 a 198.57 11.99968 YES YES  30 a 206.91 3.94639 YES YES  31 a 228.94 0.28960 YES YES  32 a 239.72 18.41340 YES YES  33 a 245.68 8.24168 YES YES  34 a 252.46 0.15882 YES YES  35 a 256.57 5.79893 YES YES  36 a 260.98 28.40841 YES YES  37 a 370.36 0.10432 YES YES  38 a 372.58 0.74813 YES YES  39 a 386.75 2.72175 YES YES  40 a 421.84 7.82466 YES YES  41 a 434.71 0.77943 YES YES  42 a 442.70 1.29949 YES YES  43 a 444.57 3.04001 YES YES  44 a 447.08 2.64349 YES YES  45 a 447.40 19.95436 YES YES  46 a 450.47 4.55004 YES YES  47 a 469.64 0.80985 YES YES  48 a 470.64 3.43539 YES YES  49 a 474.71 41.41339 YES YES  50 a 476.26 4.50662 YES YES  51 a 484.52 1.49431 YES YES  52 a 565.33 0.17435 YES YES  53 a 586.65 0.26017 YES YES  54 a 590.24 0.04028 YES YES  55 a 644.20 24.51508 YES YES  56 a 666.98 10.42314 YES YES  57 a 667.49 12.76993 YES YES  58 a 679.67 0.92687 YES YES  59 a 684.01 11.04120 YES YES  60 a 685.64 19.33860 YES YES  61 a 687.77 4.76465 YES YES  62 a 688.15 9.79993 YES YES  63 a 689.40 12.27567 YES YES  64 a 720.98 18.90598 YES YES  65 a 771.62 0.57097 YES YES  66 a 789.65 22.28753 YES YES  67 a 791.93 38.95220 YES YES  68 a 792.68 68.61130 YES YES  69 a 793.59 19.40706 YES YES  70 a 798.20 0.38025 YES YES  71 a 800.51 0.07845 YES YES  72 a 807.71 3.88543 YES YES  73 a 809.25 5.53769 YES YES  74 a 819.71 73.43460 YES YES  75 a 821.88 2.00066 YES YES  76 a 823.05 61.62611 YES YES  77 a 881.34 0.82674 YES YES  78 a 883.36 0.44141 YES YES  79 a 898.99 0.72370 YES YES  80 a 904.67 0.77312 YES YES  81 a 961.86 0.39358 YES YES  82 a 963.32 1.75160 YES YES  83 a 964.79 0.58862 YES YES  84 a 967.76 1.01512 YES YES  85 a 998.20 246.09428 YES YES  86 a 1019.97 0.17014 YES YES  87 a 1021.65 0.17012 YES YES  88 a 1046.62 0.06556 YES YES  89 a 1047.35 0.01837 YES YES  90 a 1050.25 0.27597 YES YES  91 a 1050.70 0.15967 YES YES  92 a 1059.23 0.70682 YES YES  93 a 1060.14 0.25918 YES YES  94 a 1065.25 41.85751 YES YES  95 a 1072.39 28.00461 YES YES  96 a 1073.62 25.59252 YES YES  97 a 1074.55 9.25900 YES YES  98 a 1075.39 29.16966 YES YES  99 a 1079.05 63.51488 YES YES  100 a 1086.98 1.22599 YES YES  101 a 1091.77 0.05285 YES YES  102 a 1093.70 1.84869 YES YES  103 a 1094.23 0.19003 YES YES  104 a 1094.84 0.45097 YES YES  105 a 1096.45 0.34689 YES YES  106 a 1096.91 0.44496 YES YES  107 a 1098.20 1.81717 YES YES  108 a 1128.01 1.52591 YES YES  109 a 1129.60 5.41254 YES YES  110 a 1135.69 7.63697 YES YES  111 a 1138.18 4.71062 YES YES  112 a 1167.51 54.25302 YES YES  113 a 1170.83 7.93459 YES YES  114 a 1171.38 8.51498 YES YES  115 a 1183.99 0.24912 YES YES  116 a 1184.52 2.17961 YES YES  117 a 1197.68 30.04140 YES YES  118 a 1211.48 24.56947 YES YES  119 a 1212.29 33.58238 YES YES  120 a 1212.58 17.86885 YES YES  121 a 1229.86 1.44878 YES YES  122 a 1230.43 11.52683 YES YES  123 a 1232.47 7.06626 YES YES  124 a 1289.86 49.49456 YES YES  125 a 1339.60 20.83242 YES YES  126 a 1340.79 25.81449 YES YES  127 a 1344.74 5.74711 YES YES  128 a 1349.08 6.05770 YES YES  129 a 1351.54 5.67400 YES YES  130 a 1368.78 0.27160 YES YES  131 a 1369.39 2.38060 YES YES  132 a 1378.30 17.23779 YES YES  133 a 1386.68 0.42077 YES YES  134 a 1390.42 29.78796 YES YES  135 a 1391.79 11.21723 YES YES  136 a 1392.28 30.58025 YES YES  137 a 1393.14 34.25625 YES YES  138 a 1447.29 2.45025 YES YES  139 a 1510.12 13.24246 YES YES  140 a 1519.79 12.25700 YES YES  141 a 1523.53 5.38195 YES YES  142 a 1524.15 5.46943 YES YES  143 a 1539.25 57.86472 YES YES  144 a 1539.64 85.97424 YES YES  145 a 1553.51 57.86741 YES YES  146 a 1573.74 39.40999 YES YES  147 a 1575.29 44.86591 YES YES  148 a 1597.56 2.35946 YES YES  149 a 1599.73 161.03049 YES YES  150 a 1604.20 22.89465 YES YES  151 a 1620.51 452.63734 YES YES  152 a 1650.60 17.87021 YES YES  153 a 1683.36 175.71855 YES YES  154 a 1697.24 14.01231 YES YES  155 a 1701.84 42.87038 YES YES  156 a 1711.71 20.44483 YES YES  157 a 1712.50 12.51155 YES YES  158 a 1715.90 16.80831 YES YES  159 a 1729.36 77.03579 YES YES  160 a 1730.02 55.17099 YES YES  161 a 1740.38 34.06883 YES YES  162 a 1742.58 34.17581 YES YES  163 a 3253.71 12.01196 YES YES  164 a 3285.80 1.62416 YES YES  165 a 3289.80 3.77694 YES YES  166 a 3293.49 2.84456 YES YES  167 a 3298.11 4.67047 YES YES  168 a 3300.54 10.97837 YES YES  169 a 3304.89 7.63942 YES YES  170 a 3307.33 0.59296 YES YES  171 a 3307.99 0.42889 YES YES  172 a 3308.22 0.03470 YES YES  173 a 3309.04 1.34211 YES YES  174 a 3317.24 0.63696 YES YES  175 a 3319.58 8.27933 YES YES  176 a 3321.81 0.79627 YES YES  177 a 3323.46 3.23231 YES YES  178 a 3324.57 3.04206 YES YES  179 a 3325.33 1.00251 YES YES  180 a 3325.83 1.72006 YES YES  181 a 3329.36 1.69490 YES YES  182 a 3330.48 2.59267 YES YES  183 a 3331.20 2.74580 YES YES  184 a 3338.50 0.13390 YES YES  185 a 3346.42 2.41932 YES YES  186 a 3347.25 2.05388 YES YES |

Optimized atomic coordinates from the DFT calculations (B3LYP/SV(P) level) (Bohr Units):

| 0.24650364174291 2.87659262924049 2.83483080402110 In  1.20552435274971 -1.66719047230253 -0.28994824733899 In  0.70332476728867 6.68962752728689 0.89190360196409 N  -3.66299548887718 4.68056394133259 2.61587848195148 N  3.18945989309635 0.15516292288374 -3.58184989610677 N  5.06846145491304 -3.23536946277204 -0.23705333913755 N  -0.41158885103128 -4.50198563816746 2.46327255082130 N  -0.95388068093739 -4.40007581893323 -2.62085945896580 N  2.94709847773566 7.56806792236874 0.07227292139135 C  -1.37273555673902 8.20334852983836 0.81008987552196 C  -3.76573690916538 7.10525706181539 1.77031339684439 C  -5.76278502551853 3.56426156726529 3.52271075527931 C  2.15082547503996 1.87416774463160 -5.16137748799709 C  5.66004534142358 -0.56658595456852 -3.90650989292470 C  6.68218025233749 -2.36828615856873 -2.08062130857192 C  5.90827127357444 -4.94263520345225 1.47130054883839 C  -0.09978139484860 -4.41610153571290 4.99656835571392 C  -1.96887398047372 -6.28487992293127 1.42236767237834 C  -2.27187611212858 -6.22281105191163 -1.33155106578665 C  -1.11849274113747 -4.29136012410893 -5.16908783481111 C  3.25664311370661 9.99576838339891 -0.88420426339770 C  -1.18322070439616 10.67912246702872 -0.12504419302509 C  -6.05045495925452 8.45237592814793 1.85243303940812 C  -8.08557281943822 4.78719183233035 3.63472442521703 C  3.45287965836314 2.94985437021167 -7.15780949716941 C  7.07970856120136 0.46901917710202 -5.92038847148525 C  9.21033343214997 -3.21990759071109 -2.12381432617341 C  8.35719313232105 -5.86316150283433 1.48915544990084 C  -1.26763168303426 -6.09046583056148 6.63654536667325 C  -3.22243514633503 -8.03158072363909 3.00174270213737 C  -3.84434610292796 -7.90995691248658 -2.66567253855757 C  -2.59407869932718 -5.93346724758675 -6.57562406293051 C  1.14059222972607 11.58219049762164 -0.97784825504721 C  -8.22251878650648 7.29000471869641 2.78623163257300 c  5.98960179778511 2.20944965340842 -7.54592475227261 C  10.05418165407895 -4.95739006599150 -0.35223100637915 C  -2.86910132572847 -7.94874374008325 5.59958901022274 C  -4.01026813511838 -7.77576671705875 -5.28067737672853 C  -5.55898096402142 1.61436382314036 4.18726843023232 H  4.55208127444421 6.26734072810604 0.20194614995409 H  0.17694093451901 2.38205107544843 -4.78656471767648 H  4.53119435469247 -5.59207984842535 2.86821556909699 H  1.15530801046792 -2.93462570657529 5.71900422618016 H  0.00354320527932 -2.82342205162859 -6.09454729630734 H  5.11381571417594 10.62977663610685 -1.52653329844268 H  -2.83455712837683 11.91026734975235 -0.18995938734804 H  -6.14754309259007 10.40359026362611 1.19667944746828 H  -9.74247880397590 3.80455808503122 4.37827832675975 H  2.52398141148018 4.31612029618979 -8.39535320669434 H  9.03807054548917 -0.10243838685631 -6.21391351201892 H  10.51622622477392 -2.52025209159545 -3.55628845315413 H  8.93251348834206 -7.25316306881786 2.90224392826952 H  -0.93403035179282 -5.96061138237853 8.66969544930365 H  -4.46890649650370 -9.46044321889046 2.19447723987076 H  -4.92781237979618 -9.34203392219633 -1.65486369988782 H  -2.63668455954406 -5.78381999965003 -8.63387758493400 H  1.29910822867752 13.51087535673452 -1.70340635480014 H  -10.00615005268581 8.33169992105891 2.85610005150894 H  7.08642218213454 2.99521153238309 -9.11097507098194 H  12.01228800497543 -5.61581367058080 -0.39509356304203 H  -3.82725506882782 -9.31257531980221 6.82107009103120 H  -5.22017112298610 -9.09455669174469 -6.31348473593320 H |
| --- |

List of calculated frequencies (B3LYP/SV(P) level):

| mode symmetry wave number IR intensity selection rules  cm**(-1) km/mol IR RAMAN |
| --- |
| 1 -0.00 0.00000 - -  2 0.00 0.00000 - -  3 0.00 0.00000 - -  4 0.00 0.00000 - -  5 0.00 0.00000 - -  6 0.00 0.00000 - -  7 a 8.24 0.06487 YES YES  8 a 13.07 0.69034 YES YES  9 a 16.89 1.30680 YES YES  10 a 26.64 1.15563 YES YES  11 a 28.39 3.55246 YES YES  12 a 31.31 0.62673 YES YES  13 a 33.73 4.87732 YES YES  14 a 48.66 4.17792 YES YES  15 a 68.71 1.86731 YES YES  16 a 78.32 24.42848 YES YES  17 a 80.24 4.90109 YES YES  18 a 84.52 1.00224 YES YES  19 a 89.41 11.14839 YES YES  20 a 98.63 5.25608 YES YES  21 a 100.58 5.62718 YES YES  22 a 116.24 109.47422 YES YES  23 a 126.72 26.96017 YES YES  24 a 138.60 60.81725 YES YES  25 a 142.00 209.47303 YES YES  26 a 153.97 14.77459 YES YES  27 a 160.60 47.38628 YES YES  28 a 163.67 0.44464 YES YES  29 a 187.64 1.12021 YES YES  30 a 194.78 0.95306 YES YES  31 a 222.30 105.03177 YES YES  32 a 231.53 6.07307 YES YES  33 a 236.09 11.20261 YES YES  34 a 238.22 14.31336 YES YES  35 a 239.94 0.38399 YES YES  36 a 241.34 0.70692 YES YES  37 a 359.10 9.98955 YES YES  38 a 366.23 8.93117 YES YES  39 a 367.26 5.86378 YES YES  40 a 418.61 30.42887 YES YES  41 a 420.14 1.38322 YES YES  42 a 423.58 25.09637 YES YES  43 a 424.30 0.11476 YES YES  44 a 429.12 5.35989 YES YES  45 a 432.86 12.51525 YES YES  46 a 439.28 7.31875 YES YES  47 a 443.45 1.78414 YES YES  48 a 451.01 5.79002 YES YES  49 a 458.51 0.79482 YES YES  50 a 459.29 2.74516 YES YES  51 a 464.33 8.04434 YES YES  52 a 555.15 0.70019 YES YES  53 a 560.03 1.02165 YES YES  54 a 564.97 1.65702 YES YES  55 a 636.56 16.21584 YES YES  56 a 638.60 0.69702 YES YES  57 a 643.64 1.37858 YES YES  58 a 652.23 462.93604 YES YES  59 a 659.17 17.56354 YES YES  60 a 659.61 2.31667 YES YES  61 a 660.47 3.03809 YES YES  62 a 662.94 0.17928 YES YES  63 a 663.03 1.23610 YES YES  64 a 725.58 9.87585 YES YES  65 a 732.36 3.52992 YES YES  66 a 751.47 8.20294 YES YES  67 a 753.05 17.85279 YES YES  68 a 754.61 2.82634 YES YES  69 a 761.69 364.41542 YES YES  70 a 763.30 196.54546 YES YES  71 a 771.43 27.17122 YES YES  72 a 774.04 13.71919 YES YES  73 a 775.65 94.28533 YES YES  74 a 777.14 5.35576 YES YES  75 a 783.46 56.40183 YES YES  76 a 817.39 0.34495 YES YES  77 a 824.29 0.36722 YES YES  78 a 842.83 5.44102 YES YES  79 a 894.37 43.65821 YES YES  80 a 897.76 19.00263 YES YES  81 a 901.19 9.63847 YES YES  82 a 902.76 62.82852 YES YES  83 a 916.40 757.47129 YES YES  84 a 917.80 32.39245 YES YES  85 a 924.39 4406.06055 YES YES  86 a 981.63 1.36341 YES YES  87 a 983.94 1.01724 YES YES  88 a 988.71 0.59795 YES YES  89 a 991.14 3.67008 YES YES  90 a 994.76 0.47634 YES YES  91 a 995.67 3.07207 YES YES  92 a 1003.09 242.68281 YES YES  93 a 1010.17 500.15536 YES YES  94 a 1011.13 7.79661 YES YES  95 a 1017.43 0.12440 YES YES  96 a 1022.92 0.10165 YES YES  97 a 1024.91 0.33958 YES YES  98 a 1025.27 8.07088 YES YES  99 a 1027.30 0.11598 YES YES  100 a 1028.13 2.75892 YES YES  101 a 1038.54 0.31467 YES YES  102 a 1041.97 0.08041 YES YES  103 a 1049.51 12.45498 YES YES  104 a 1051.76 3.37688 YES YES  105 a 1055.05 1.72652 YES YES  106 a 1069.04 7.86128 YES YES  107 a 1074.80 5.43278 YES YES  108 a 1075.37 11.48498 YES YES  109 a 1078.27 4.54237 YES YES  110 a 1084.15 4.53551 YES YES  111 a 1089.40 3.26672 YES YES  112 a 1125.75 92.41694 YES YES  113 a 1128.33 1.93336 YES YES  114 a 1128.50 2.52315 YES YES  115 a 1144.49 1.62256 YES YES  116 a 1147.80 4.16446 YES YES  117 a 1150.51 5.69829 YES YES  118 a 1167.06 1485.38530 YES YES  119 a 1174.02 18.95969 YES YES  120 a 1174.85 35.15826 YES YES  121 a 1177.21 11.99495 YES YES  122 a 1185.61 379.48891 YES YES  123 a 1192.07 0.80990 YES YES  124 a 1258.06 1266.93172 YES YES  125 a 1275.50 109.34627 YES YES  126 a 1291.60 486.99740 YES YES  127 a 1298.86 1696.40535 YES YES  128 a 1309.85 5.17464 YES YES  129 a 1313.65 133.93403 YES YES  130 a 1315.57 10.03228 YES YES  131 a 1320.21 854.51383 YES YES  132 a 1342.78 164.96698 YES YES  133 a 1345.54 12.20462 YES YES  134 a 1346.87 15.23682 YES YES  135 a 1347.40 3.88916 YES YES  136 a 1348.37 3.08905 YES YES  137 a 1353.59 47.74628 YES YES  138 a 1357.75 98.63186 YES YES  139 a 1426.15 2785.44816 YES YES  140 a 1454.61 28.43990 YES YES  141 a 1456.17 133.28588 YES YES  142 a 1460.43 140.16789 YES YES  143 a 1470.26 173.79072 YES YES  144 a 1471.93 199.85826 YES YES  145 a 1477.20 2253.55048 YES YES  146 a 1478.53 76.10630 YES YES  147 a 1499.77 134.20942 YES YES  148 a 1501.16 59.52747 YES YES  149 a 1506.18 49.80861 YES YES  150 a 1524.88 33.16387 YES YES  151 a 1574.37 169.78862 YES YES  152 a 1584.16 90.29798 YES YES  153 a 1587.87 276.12234 YES YES  154 a 1595.29 130.17736 YES YES  155 a 1603.97 131.27124 YES YES  156 a 1612.62 81.95876 YES YES  157 a 1619.31 101.06778 YES YES  158 a 1621.43 11.34141 YES YES  159 a 1634.98 8.31847 YES YES  160 a 1638.04 2.00082 YES YES  161 a 1640.58 93.15333 YES YES  162 a 1652.57 8.36256 YES YES  163 a 3160.99 11.73030 YES YES  164 a 3166.35 7.90910 YES YES  165 a 3181.17 5.99703 YES YES  166 a 3182.31 4.03266 YES YES  167 a 3200.15 1.94590 YES YES  168 a 3200.47 0.82833 YES YES  169 a 3201.07 1.08526 YES YES  170 a 3201.85 2.68138 YES YES  171 a 3203.34 0.62820 YES YES  172 a 3204.24 6.80466 YES YES  173 a 3205.77 10.75658 YES YES  174 a 3205.97 12.93096 YES YES  175 a 3215.46 5.49767 YES YES  176 a 3216.73 6.36764 YES YES  177 a 3218.07 2.51867 YES YES  178 a 3218.88 18.84695 YES YES  179 a 3219.25 5.05393 YES YES  180 a 3219.57 6.36814 YES YES  181 a 3222.06 1.82920 YES YES  182 a 3222.23 3.69586 YES YES  183 a 3223.11 2.28498 YES YES  184 a 3233.03 0.03981 YES YES  185 a 3234.22 0.21218 YES YES  186 a 3235.73 1.46822 YES YES |

### (In_3_)^3+^

Atomic coordinates from the single point DFT calculations (BHLYP/SV(P) and B3LYP/SV(P) level) (Bohr Units):

| -2.68117317895056 1.21992945153091 0.00000000000000 In  0.28419222232750 -2.93213878360879 0.00000000000000 In  2.39698095662306 1.71220933207788 0.00000000000000 In |
| --- |

### [In_3_(bipy)_6_]^3+^ (from XRD: 4^3+^)

Atomic coordinates from the single point DFT calculations (BHLYP/SV(P) and B3LYP/SV(P) level) (Bohr Units):

| -0.52719393936946 2.58089610977747 -0.08890086155250 In  -2.05579364903593 -2.20538190252863 -0.54637691741017 In  2.93387064167864 -1.00351660796811 0.58521338467385 In  1.62912450954115 6.45595478848003 -0.25500340345413 N  -0.41748706714952 4.17433596770352 -4.28593068093450 N  -1.45714814155295 4.07476549496506 4.12080071653518 N  -4.35897777508624 4.77936959609270 0.01000851846500 N  -5.83343319130897 -1.02181206930907 2.34046867201517 N  -5.96218179396687 -1.78190958175374 -2.74885450072385 N  -2.89927888931869 -6.48860429595747 0.35996108272532 N  -0.11670524877367 -5.11177051429288 -3.78681318865069 N  6.01697183953655 1.79542710118088 -2.26854379533994 N  6.24887038339420 1.12109018629000 2.82635783901328 N  5.45045955124982 -4.59053514750772 -0.26725365951245 N  2.47911331143227 -4.34027679666808 3.97752611448830 N  2.83835069630826 7.43017318311207 1.75277599818093 C  1.57953778560193 7.80514466321520 -2.41569915891017 C  0.43484684164759 6.54984972710988 -4.64889774255504 C  -1.20233673956958 2.82322173635407 -6.25967304009256 C  0.08872271690233 3.67623809699655 6.08275400227869 C  -3.27362827515931 5.82124410760630 4.32280986693996 C  -5.07835791672100 5.93169754900947 2.16000899013351 C  -5.98253788271822 4.74386377199163 -1.94018574977695 C  -5.70370553404500 -0.58204530301075 4.82415506823286 C  -8.00593610353497 -0.49162681502522 1.14858510067441 C  -8.13447507455496 -1.11549174375227 -1.58468079417407 C  -6.05877778675232 -2.42816020187015 -5.15696417713209 C  -4.70904133822547 -7.16611580735481 2.00771196806079 C  -1.88531653538429 -8.29680414471298 -1.06956219521962 C  0.01620547773919 -7.49719881957116 -2.96179483676615 C  1.50227437058141 -4.40698881342392 -5.56140841563359 C  5.72889056489407 2.17433983554438 -4.75310331767679 C  7.27285615614623 3.50263833230869 -0.94514007515301 C  7.66071268939951 2.96368026248172 1.77728388194178 C  6.72256723854227 0.41780407169569 5.18334331626282 C  7.26861593115373 -4.52964442499799 -1.98674285247825 C  5.23574900571337 -6.63655372176374 1.21587814538761 C  3.20656749202221 -6.62951899593624 3.13071183177962 C  0.67667197790355 -4.27307759738919 5.75498648146847 C  4.00562020149861 9.75533364946141 1.69355107997032 C  2.66734493491004 10.20429757110508 -2.53175887744508 C  0.38616434915593 7.63637569156649 -7.03100463917246 C  -1.21466891590890 3.75201507274227 -8.69084529378765 C  -0.03503676778021 5.07395188438734 8.28613290231889 C  -3.47434514887536 7.35958959510273 6.43119029894567 C  -7.44241420242191 7.03099742185182 2.41117651114338 C  -8.36284586872829 5.79334463871184 -1.79647955423255 C  -7.66539320502498 0.39722544056406 6.23075383918315 C  -10.04243371868645 0.58289092035376 2.42035284975135 C  -10.39997338311408 -1.03439185439560 -2.94080517013914 C  -8.23518936175647 -2.35825640814053 -6.63523773463919 C  -5.51809980457349 -9.62975638770177 2.33487325464446 C  -2.61027220128516 -10.82820433516656 -0.83980559100794 C  1.84258470139693 -9.17910762036515 -3.84932760535879 C  3.33667287495530 -5.97812185499443 -6.57293038415544 C  6.69155375552343 4.24833016962295 -6.00216475704995 C  8.21822616417428 5.69698475307975 -2.00879873316908 C  9.50988948978332 4.20374737856893 3.20395053155678 C  8.57319922795800 1.50157249480828 6.66151377750136 C  8.95771435260566 -6.50061196612750 -2.35223745724392 C  6.88488627299269 -8.68650374383813 0.99378333191540 C  2.11140847478416 -8.86584711506209 4.04680386813016 C  -0.45120498437990 -6.39578501737724 6.78302777176363 C  3.87352317380010 11.15799487588215 -0.44947136114094 C  -0.46530305720580 6.22956432103964 -9.07225472584212 C  -1.81066427006358 6.99975496385949 8.41366319601066 C  -9.10763212886999 6.95432239460604 0.40298635221622 C  -9.87127302458262 1.02352833761094 4.96427803402125 C  -10.44179337261803 -1.60695519548546 -5.46238887837687 C  -4.44446210001273 -11.48488984204428 0.85264397414025 C  3.49765590743694 -8.38846682794791 -5.66199110402451 C  7.90107949301627 6.07340599346582 -4.58595624482069 C  9.96957289703834 3.44782481213562 5.65258448407614 C  8.74440740292639 -8.60328323063888 -0.82613477045134 C  0.25884003523689 -8.73039818758440 5.87577261567572 C  2.89048092605628 6.47767576113831 3.27357270301111 H  -1.77849212907820 1.14641903817353 -5.97803719987414 H  1.32261199740399 2.37846019047519 5.95569062318959 H  -5.47187455839335 3.96675914931256 -3.47580671983965 H  -4.16809161174669 -0.96719415456746 5.67057804610378 H  -4.53784462166941 -2.97923923679668 -5.93543972558954 H  -5.47462941567165 -5.88250628772179 3.00227153969329 H  1.39945447900725 -2.72382165570349 -6.17730731815153 H  4.81620732366389 0.95416350882286 -5.70235408119588 H  5.73420485354475 -0.90313853063716 5.89128022489460 H  7.42288388018866 -3.06806565201582 -3.01767180562576 H  0.13919873289322 -2.66563639502507 6.34677804755620 H  4.88976933676746 10.36810068132457 3.13081575228907 H  2.57220637455337 11.16869531195123 -4.04296043189576 H  0.93140724091389 9.33083062476641 -7.26415734824105 H  -1.72374162566123 2.72043385880897 -10.06909519276413 H  1.05882752610675 4.72782795653238 9.66692288513163 H  -4.73176109458182 8.63822811289782 6.51354582435799 H  -7.91254668755168 7.82908958107637 3.94903368524441 H  -9.47575368713438 5.71451291132250 -3.20293439024624 H  -7.51118236671276 0.63420030071901 8.00360049859501 H  -11.54169472822591 1.00783591796284 1.52890320453165 H  -11.92224510606928 -0.57829972896387 -2.10554216927202 H  -8.20308599496015 -2.80748480085388 -8.37307465594406 H  -6.77898462026697 -10.03295307314977 3.54755328699808 H  -1.83007702725848 -12.08523722365795 -1.85668171479948 H  1.93258764419165 -10.85080222508730 -3.20098568574616 H  4.44689645203612 -5.39780167918567 -7.85877074676570 H  6.52726598656243 4.41565953537122 -7.78203307728347 H  9.06423537874771 6.91689943522186 -0.99936077367564 H  10.44340460871954 5.55976013230736 2.48789882680749 H  8.87617925611653 0.91973412933332 8.33263001474888 h  10.22651587715236 -6.40506899030554 -3.61867142667118 H  6.72692376321120 -10.10972288654900 2.07650631914565 H  2.64189167750760 -10.45920410377870 3.41227228498666 H  -1.68301753936193 -6.24742738200361 8.08047590691806 H  4.61367642819297 12.79261552498912 -0.50517489983420 H  -0.53338584360363 6.95721573866093 -10.71199598022492 H  -1.88280547183866 8.06578875201550 9.85631072596215 H  -10.73891097815221 7.69120554611067 0.54010709163124 H  -11.25093639157753 1.75144791637662 5.85283376335797 H  -11.97112756505977 -1.48885551106929 -6.39512713997147 H  -4.97280382714430 -13.19371122458697 1.00653969330397 H  4.75453710358059 -9.50766460410077 -6.28684934155828 H  8.51019645514485 7.57122400099880 -5.36588896118938 H  11.23545300583202 4.26082142715442 6.63206207193651 H  9.87258233154806 -9.98494833929826 -1.02886890097044 H  -0.51574846045647 -10.22459741259541 6.50036162963406 H |
| --- |

### [In(bipy)_2_]^+^ (1^st^ cut out of 4^3+^)

Atomic coordinates from the single point DFT calculations (BHLYP/SV(P) and B3LYP/SV(P) level) (Bohr Units):

| 0.14300700265331 -0.21759179889414 2.82271360503840 In  0.57839544721337 3.02935133355199 -0.17080877970017 N  4.26498926123606 -0.22394103228630 1.04075753639198 N  -4.19388502299790 0.02647912884359 1.42634514550146 N  -0.63452874349167 -3.06836399441461 -0.46273494148627 N  -1.22302829975420 4.77444163723375 -0.56073171143160 C  2.67146482897519 3.09865360883373 -1.62188977905776 C  4.71191904374658 1.28899937594188 -0.96237722984099 C  6.11106088467722 -1.68989134280804 1.92402806289801 C  -5.86475149551664 1.63365445975910 2.43777689312555 C  -4.73073887941203 -1.07324030836455 -0.78566760611798 C  -2.89827865776524 -3.04564795402665 -1.62152303020748 C  1.02135375892225 -4.88721388865707 -1.08747806544711 C  -1.02254662843485 6.61050319122106 -2.39405709237001 C  2.91326991325093 4.87603169308951 -3.55455280571668 C  7.02916763867209 1.24087749713953 -2.18114101233624 C  8.48611524376765 -1.76256996724322 0.86231139619970 C  -8.08818960743723 2.30030253858645 1.23956901462184 C  -6.87625660405656 -0.44700037112086 -2.14825315667865 C  -3.55989456780827 -4.85953149418785 -3.39138131445331 C  0.46410139598120 -6.74847294267394 -2.82330991397098 C  1.03832004400388 6.62272753940120 -3.92053422868102 C  8.93202373229244 -0.31544095193105 -1.27129021977551 C  -8.54875857947192 1.28583189170181 -1.13457504844321 C  -1.85280404409901 -6.73930241256911 -3.99467291480030 C  -2.69332124009393 4.74082256990552 0.46878271053719 H  5.77471730627714 -2.73023699351483 3.34792939104733 H  -5.50767378560363 2.34601395184354 4.04647040545718 H  2.63670189760485 -4.89147074405972 -0.30425864591465 H  -2.30719652355123 7.84956268758498 -2.58729031538919 H  4.36674662066934 4.87477263356085 -4.60823604187035 H  7.31222016131693 2.26530967029072 -3.62794698047477 H  9.78355984176569 -2.77841673302676 1.57475821692120 H  -9.26171679579145 3.41561684891809 2.01534589676866 H  -7.19271600638095 -1.19421418858320 -3.74960218482519 H  -5.17121613320922 -4.81027238255282 -4.18144250760151 H  1.66902587620181 -8.02331999250613 -3.20516781392609 H  1.17985808262011 7.83625988099505 -5.23591274083203 H  10.52185527120585 -0.38743493641439 -2.10202229256990 H  -10.00635119804748 1.77958910925229 -2.05896233446015 H  -2.27318198327347 -8.00337470848133 -5.19811513577614 H |
| --- |

### [In(bipy)_2_]^+^ (2^nd^ cut out of 4^3+^)

Atomic coordinates from the single point DFT calculations (BHLYP/SV(P) and B3LYP/SV(P) level) (Bohr Units):

| -0.11258431232455 -0.15032468002893 -2.27601745688063 In  1.51925778748962 4.46530787581804 -1.04453201385763 N  -2.57529122849717 2.00070168600783 0.85991398379988 N  2.67349449851953 -1.90644361336723 0.73735821766405 N  -1.57916652859392 -4.45174589866738 -0.79430542482570 N  3.46705524055220 5.64433538948178 -2.14706587046431 C  -0.24880764033899 5.85616544448192 0.04297563099241 C  -2.31473846893866 4.48677959905505 1.35034432855580 C  -4.23324156587896 0.66691484903337 2.18150165089309 C  4.41012413372408 -0.51017642227434 1.87779621589374 C  2.30419556454245 -4.28785116833960 1.52760433739842 C  0.33466885967218 -5.78132321192078 0.23349423263169 C  -3.42387823153592 -5.75676657954723 -1.93738705134522 C  3.72836697546768 8.23628725623119 -2.18963935308795 C  -0.20792066906941 8.47099067354491 0.00737107737390 C  -3.86807078618356 5.66427402860321 3.13731511768131 C  -5.75828553302033 1.69317264833558 4.02687871136610 C  5.89597061620342 -1.40129324498602 3.84490675433187 C  3.69603187934533 -5.31619145292999 3.52174145840792 C  0.42021123506798 -8.42979721041219 0.09922089267850 C  -3.49380756440085 -8.36519719358689 -2.09882356400430 C  1.81755231177613 9.67314357394851 -1.14861482609490 C  -5.58341339967699 4.24525211554763 4.48731975596256 C  5.51585851451034 -3.84664919659242 4.66433629499326 C  -1.50861418151369 -9.71596966769661 -1.09278407255081 C  4.73147905161599 4.64597625194407 -2.93915768185078 H  -4.37806456472848 -1.08932635180899 1.83862066161691 H  4.64916461280448 1.18020982842440 1.32256690382626 h  -4.76915719559285 -4.83199505472469 -2.68437038556065 H  5.17848099472985 9.00880199238026 -2.91304182655253 H  -1.53444770412858 9.42092467073707 0.75626348830552 H  -3.74055735645863 7.43284275208444 3.41812840978460 H  -6.89972457200987 0.66516017044013 4.95600169835745 H  7.14108816372727 -0.35632306818606 4.60685189034041 H  3.39336734970040 -6.99577272638546 4.07869287289975 H  1.79516622398670 -9.32866597942489 0.82335000161659 H  -4.87447605156812 -9.20291165291804 -2.88287256449566 H  1.88718600549880 11.46549745246172 -1.22207244777069 H  -6.63246969803090 5.01995433654040 5.72105783297289 H  6.50264653245156 -4.51291872310366 6.00791829674258 H  -1.46862177982349 -11.50634145244019 -1.21808203040573 H |
| --- |

### [In(bipy)_2_]^+^ (3^rd^ cut out of 4^3+^)

Atomic coordinates from the single point DFT calculations (BHLYP/SV(P) and B3LYP/SV(P) level) (Bohr Units):

| 0.22029540360019 2.40601857955871 -0.26729953401895 In  -4.35298835869598 1.46889419719434 1.22002996549796 N  -1.89229272918413 -0.70895389863165 -2.74197577378331 N  1.63243546546354 -0.68192917651036 2.62222449150901 N  4.58846751892264 0.83957428910196 -1.35030944644380 N  -5.54444552421634 2.62278600607228 3.12473091438497 C  -5.70645145410934 -0.06128245065659 -0.27935285114636 C  -4.30685526214087 -1.41237873024775 -2.30225707799710 C  -0.59325986985754 -1.95070111793021 -4.47302757462236 C  0.02668589056471 -1.85822212338648 4.19932567897749 C  3.96709168716039 -1.60584482004455 2.42986191576433 C  5.67248413324000 -0.36319046762314 0.59052894498791 C  6.09541752003386 1.86198760550211 -3.06809091624604 C  -8.08452053413781 2.34458576564530 3.63102468319329 C  -8.29696832503057 -0.37142351637810 0.05581720804672 C  -5.40712126336946 -3.35067270262221 -3.72020076673156 C  -1.57241549865205 -3.87294689146712 -5.98073180149647 C  0.68821422840611 -3.91911038657916 5.66433586440566 C  4.76744387209738 -3.68320698531511 3.85476140460486 C  8.29154385635706 -0.47343018325508 0.87437057330555 C  8.71011874899536 1.79058074522375 -2.94605754565220 C  -9.48443554224662 0.83054847881511 2.01545941790654 C  -4.06263124900343 -4.54211302089636 -5.58051758329610 C  3.11387268380271 -4.84883054770826 5.45691558137545 c  9.79224119645015 0.61791313905012 -0.91643923384577 C  -4.58360355186822 3.69884892400076 4.19325930170706 H  1.12922676065142 -1.50173299999099 -4.70630297350278 H  -1.65497073702849 -1.24034957293574 4.31418099238280 H  5.33817749370020 2.69383991794327 -4.46722076792503 H  -8.84785463342597 3.16074253374177 5.03606294870083 H  -9.23521056771510 -1.41054476001615 -1.06800343188787 H  -7.10119754313872 -3.84462260830385 -3.38987584334909 H  -0.57165171255170 -4.68751092827969 -7.22887891397622 H  -0.49605977377246 -4.67357896493833 6.78293463005153 H  6.45402840948599 -4.28066678335209 3.70865311794005 H  9.01962856977035 -1.29651372601613 2.29403136294005 H  9.71808155567737 2.53328658593064 -4.23258478990321 H  -11.25068887225643 0.62886428014460 2.26554312739421 H  -4.83356888782031 -5.81473244372724 -6.58496082906074 H  3.62675999241152 -6.27846709645024 6.41398681049560 H  11.57912146575274 0.56255060031015 -0.75274527286931 H |
| --- |
